# CITF1 interacts with FIT and regulates copper–iron crosstalk in Arabidopsis

**DOI:** 10.1093/plcell/koag114

**Published:** 2026-04-16

**Authors:** J C Chia, X Liu, S Dey, J Yan, Y Niu, O K Vatamaniuk

**Affiliations:** Plant Biology Section, School of Integrative Plant Science, Cornell University, Ithaca, NY, United States; Plant Biology Section, School of Integrative Plant Science, Cornell University, Ithaca, NY, United States; Plant Biology Section, School of Integrative Plant Science, Cornell University, Ithaca, NY, United States; Department of Botany, University of Calcutta, Kolkata 19, India; Plant Biology Section, School of Integrative Plant Science, Cornell University, Ithaca, NY, United States; Plant Biology Section, School of Integrative Plant Science, Cornell University, Ithaca, NY, United States; Plant Biology Section, School of Integrative Plant Science, Cornell University, Ithaca, NY, United States

## Abstract

Iron (Fe) and copper (Cu) are essential yet potentially toxic metals with interconnected metabolic pathways; however, the mechanisms underlying Fe–Cu crosstalk remain poorly defined. Here, we show that CITF1 (COPPER DEFICIENCY INDUCED TRANSCRIPTION FACTOR 1), a Cu homeostasis regulator in *Arabidopsis thaliana*, physically interacts with FIT (FER-LIKE IRON DEFICIENCY-INDUCED TRANSCRIPTION FACTOR), the central Fe homeostasis regulator, forming a nutrient-responsive transcriptional module. Under Cu deficiency, the CITF1–FIT complex accumulates and promotes expression of the Cu uptake genes *COPT2 (COPPER TRANSPORTER 2), FRO4 (FERRIC REDUCTION OXIDASE 4),* and *FRO5 (FERRIC REDUCTION OXIDASE 5)*. Proteasome-dependent degradation regulates CITF1 and FIT stability, with Cu deficiency delaying their turnover in a CITF1-dependent manner. Under Fe deficiency, *CITF1* expression is downregulated, allowing FIT to interact with bHLH38/39/100/101 partners and activate Fe uptake genes, as CITF1 disrupts these interactions. Thus, CITF1 negatively regulates Fe acquisition. Consistent with this, *citf1-*1 and *citf1-*2 mutants show reduced sensitivity to Fe deficiency. Under Cu deficiency, the *citf1-*2 and *fit-*2 mutants have additive effects and under Fe deficiency, the double mutant shows partial suppression of the *fit-2* slow growth phenotype, supporting the positive and negative roles of CITF1 in Cu and Fe homeostasis, respectively. Complete loss of CITF1 function in the homozygous *citf1-*1 *fit-*2 double mutant causes embryo lethality, revealing roles for CITF1 and FIT in embryo development. These findings establish CITF1 as a nutrient-responsive regulator of Cu/Fe crosstalk, functioning through interactions with FIT to prioritize Cu or Fe acquisition and balance micronutrient homeostasis.

## Teaser

Plants use a nutrient switch where CITF1 partners with FIT to drive copper uptake and, when iron is scarce, releases FIT to cooperate with other factors that activate iron uptake.

## Introduction

Copper (Cu) and iron (Fe) are essential micronutrients that, due to similar redox properties, have been employed by nature as cofactors of redox-active enzymes involved in essential physiological processes, including photosynthesis, respiration, lignin synthesis, and scavenging of reactive oxygen species (ROS) (Ravet and Pilon 2013). At the same time, ionic forms of Cu and Fe cause toxicity either due to their direct involvement in ROS production and/or due to mis-metalation of proteins if these transition metals accumulate in cells in excess; the latter is especially attributed to Cu, considering the tightness of its binding to cellular ligands based on the Irving–Williams series (Irving and Williams 1953; Martin 1987; Osman and Robinson 2023). Thus, the cellular concentrations of Cu and Fe must be tightly controlled to prevent deficiency while avoiding toxicity. This control involves multiple mechanisms, including transcriptional and post-translational regulation of uptake and internal transport, and intracellular trafficking *via* chaperone proteins, ensuring correct protein metallation while preventing cytoplasmic exposure to metal ions in transit (Harrison et al. 1999; Meiser et al. 2011; Riaz and Guerinot 2021; Ishka et al. 2022). The latter mechanism has been documented for Cu in various organisms, including plants, while Fe chaperones operate mainly in animals (Harrison et al. 1999; Riaz and Guerinot 2021; Ishka et al. 2022; Philpott et al. 2023).

Based on studies in *Arabidopsis thaliana*, Cu uptake and internal transport are regulated by a conserved transcription factor (TF) SQUAMOSA PROMOTER BINDING PROTEIN-LIKE 7 (SPL7) and COPPER DEFICIENCY-INDUCED TRANSCRIPTION FACTOR 1 (CITF1, *alias* bHLH160); CITF1 is a member of the clade Ib of basic helix-loop-helix (bHLH) TFs (Yamasaki et al. 2009; Bernal et al. 2012; Yan et al. 2017). Among the SPL7 and CITF1 co-regulated genes are those that encode members of the high-affinity Cu uptake system, including IRON (FE)/CU REDUCTASE OXIDASES, FRO4 and FRO5, and a Cu transporter, COPT2, that is a member of the CTR/COPT/SLC31 (COPPER TRANSPORTER/COPPER TRANSPORTER/SOLUTE CARRIER 31) family (Yamasaki et al. 2009; Bernal et al. 2012; Yan et al. 2017). The increased expression of *FRO4*, *FRO5,* and *COPT2,* as well as other SPL7- and CITF1-regulated genes, and the increased expression of *CITF1* constitute a signature of Cu deficiency response (Chia et al. 2023).

The transcriptional regulation of Fe uptake in *A. thaliana* engages TFs mainly from the bHLH family. Among them, FIT (FER-LIKE FE DEFICIENCY-INDUCED TRANSCRIPTION FACTOR, alias bHLH29) acts as an essential regulatory hub orchestrating Fe acquisition into the *A. thaliana* root (Colangelo and Guerinot 2004; Jakoby et al. 2004). *FIT* is upregulated under Fe deficiency by a cascade of TFs from the bHLH family; still, FIT protein must hetero-oligomerize with the subgroup Ib bHLH TFs such as bHLH038, bHLH039, bHLH100, and bHLH101 to form a transcription-activating DNA-interacting complex (Yuan et al. 2008; Wang et al. 2013; Kim et al. 2019; Gao et al. 2020; Gao et al. 2024). These FIT-bHLH complexes regulate the expression of Fe acquisition genes, including *FE-REGULATED TRANSPORTER* (*IRT1*) and *FRO2* (Colangelo and Guerinot 2004; Yuan et al. 2008; Wang et al. 2013). The increased expression of *FIT, bHLH038/039/100/101,* and their downstream targets (eg*, IRT1* and *FRO2*) in the root is used as a signature of Fe deficiency response in *A. thaliana* (Schwarz and Bauer 2020; Riaz and Guerinot 2021). FIT is recycled under Fe sufficiency *via* BRUTUS-LIKE (BTSL) E3 ligases and the 26S proteasome degradation pathway, and this process is important for switching off Fe uptake to prevent Fe overload (Meiser et al. 2011; Sivitz et al. 2011; Rodríguez-Celma et al. 2019).

Cu and Fe uptake into plant cells is tightly linked to each other, and the existence of the cross-talk between Cu and Fe homeostasis is now well-documented (Bernal et al. 2012; Waters and Armbrust 2013; Kastoori Ramamurthy et al. 2018; Cai et al. 2021; Sheng et al. 2021; Chia et al. 2023). The signature of this cross-talk is the increased Cu uptake under Fe deficiency and vice versa (Waters and Armbrust 2013; Kastoori Ramamurthy et al. 2018; Cai et al. 2021; Sheng et al. 2021; Chia et al. 2023). This cross-talk is regulated by the differential expression of Cu and Fe uptake systems in response to the availability of these elements in the local root environment and the needs of the developing shoot. The cross-talk between Cu and Fe in the systemic, shoot-to-root signaling is, in part, mediated by the phloem companion cell-localized Fe/Cu transporter, OLIGOPEPTIDE TRANSPORTER 3 (AtOPT3) and IRON MAN/FE UPTAKE-INDUCING PEPTIDES (IMA/FEP) (Chia et al. 2023; Cai et al. 2024; Chia and Vatamaniuk 2024). Concerning events regulating Cu/Fe cross-talk locally, recent studies have shown that FIT, via its interactions with bHLH38 and bHLH39, controls the upregulation of the Cu transport system under Fe deficiency (Cai et al. 2021). Whether FIT operates under Cu deficiency is unknown. The molecular players mediating Cu/Fe cross-talk locally under Cu deficiency are also unknown. In this regard, it is noteworthy that *CITF1,* while being upregulated by Cu deficiency, is downregulated by Fe deficiency in *A. thaliana* roots (Yan et al. 2017). This finding suggests that CITF1 might also be involved in Cu/Fe cross-talk.

The data presented in this study identify FIT as an interacting partner of CITF1 and CITF1 as a regulator of Fe/Cu cross-talk in *A. thaliana*. CITF1 promotes Cu deficiency responses by stabilizing FIT and forming a CITF1-FIT complex that activates Cu uptake genes. Conversely, under Fe deficiency, reduced *CITF1* expression allows FIT to partner with subgroup Ib bHLHs to induce Fe acquisition genes. This partner-dependent toggling ensures that plants can precisely prioritize and balance micronutrient uptake according to prevailing deficiencies.

## Results

### CITF1 interacts with FIT in yeast-2-hybrid assays

**Figure 1:**
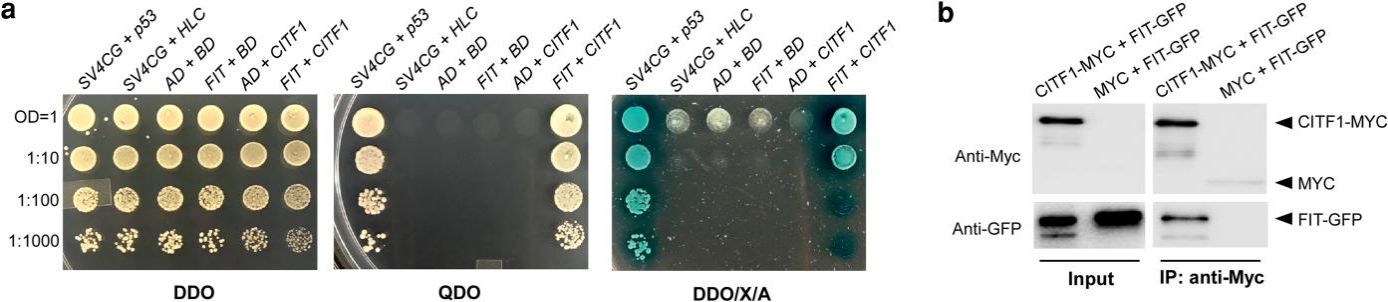
FIT is a CITF1 interacting partner. a) The yeast-2-hybrid assay used the indicated combinations of bait and prey vectors containing *CITF1* or *FIT* cDNAs, respectively, and bait and prey vectors lacking cDNA inserts (BD and AD, respectively). The prey vector expressing SV40 large T antigen (*SV4CG*) and probed with the bait vector expressing *p53* or human lamin C (*HLC*) were used as positive and negative controls, respectively. Cells co-expressing the indicated vector combinations were grown overnight to an OD_600nm_ = 1.0 in a liquid double dropout medium lacking Leu/Trp (DDO), serially 10-time diluted as indicated on the left and spotted either on the solid DDO medium (control) or on a quadruple dropout medium lacking Leu/Trp/His/Ade (QDO) to select for protein–protein interactions. Protein–protein interactions were also visualized on DDO plates supplemented with 40 *µ*g/mL X-a-Gal and 0.2 *µ*g/mL Aureobasidin A (DDO/X/A). The data shown are representative of 3 independent experiments. b) FIT co-immunoprecipitates with CITF1. The *citf1;35S_pro_:CITF1-MYC* (CITF1-MYC) overexpression line was crossed with the *citf1* mutant expressing *35S_pro_:FIT-GFP*, and the resultant FIT-GFP and CITF1-MYC co-expressing plants (CITF1-MYC + FIT-GFP) were used for co-IP. The *citf1* mutant expressing the MYC-expressing empty vector (MYC) was crossed with *citf1; 35S_pro_:FIT-GFP*-expressing plants to be used as a negative control (MYC + FIT-GFP). FIT and CITF1 were co-IP’d from total soluble fractions of proteins using the anti-MYC antibody; immunoblots were probed with anti-MYC or anti-GFP antibodies, as indicated.

CITF1 belongs to the basic helix-loop-helix (bHLH) transcription factor family, whose members function as homo- or heterodimers to bind to DNA (Heim et al. 2003; Gao and Dubos 2024). To identify potential CITF1-interacting partners, we conducted a genome-wide yeast 2-hybrid (Y2H) cDNA library screen using CITF1 as the bait. This screen yielded 17 clones displaying strong interaction signals with CITF1 (Figure S1). Sequencing of the corresponding cDNAs revealed that 14 of these clones encoded the transcription factor FIT (Table S1). The ability of CITF1 to interact with FIT in Y2H was further validated by retransforming yeast cells with CITF1-bait and FIT-prey vectors (Fig. 1a). Notably, this interaction was maintained when the N-terminal region and the bHLH domain of FIT were removed to prevent autoactivation (Lingam et al. 2011; Gratz et al. 2020), and the resulting C-terminal FIT peptide, used as bait, was co-expressed with CITF1 prey (Figure S2a). Since CITF1 is a member of the subgroup Ib of the bHLH TF family and FIT interacts with other subgroup Ib members, including bHLH38, bHLH39, bHLH100, and bHLH101, to regulate genes involved in Cu uptake (Cai et al. 2021), we tested whether CITF1 interacts with these bHLHs as well. Unlike FIT, CITF1 did not interact with bHLH38, bHLH39, bHLH100 or bHLH101 in the Y2H assay (Figure S2b, c).

### CITF1 Co-immunoprecipitates with FIT

To further validate the interaction between CITF1 and FIT in planta, we utilized the *citf1-*1 mutant previously described by Yan et al. (2017) and introduced a construct expressing *CITF1* fused to a 9×MYC epitope tag under the control of the CaMV 35S promoter (*35S_pro_:CITF1-MYC*). We intentionally expressed *CITF1-MYC* under the strong constitutive promoter, given CITF1's low transcript abundance in control conditions (Yan et al. 2017). In parallel, we generated *citf1-*1 plants expressing *FIT* fused to *GFP* under the same promoter (*35S_pro_:FIT-GFP*). These transgenic lines were subsequently crossed to obtain a co-expression line (*35S_pro_:CITF1-MYC + 35S_pro_:FIT-GFP*) in the *citf1-*1 background, which was then used for co-immunoprecipitation (co-IP) assays. Consistent with results from Y2H assays, FIT-GFP was specifically co-immunoprecipitated with CITF1-MYC using an anti-MYC monoclonal antibody (Fig. 1b). In contrast, no FIT-GFP signal was detected in eluates from *citf1-*1 plants co-expressing FIT-GFP and a MYC-tagged empty vector control (*35S_pro_:FIT-GFP* + *35S_pro_:MYC*), confirming the specificity of the CITF1-FIT interaction (Fig. 1b).

### Copper deficiency increases the abundance of the CITF1-FIT complex

**Figure 2:**
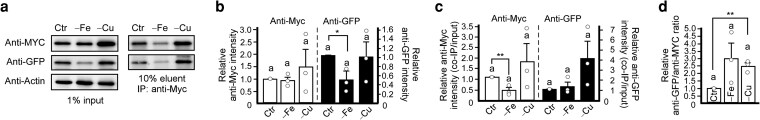
Copper deficiency favors CITF1-FIT complex formation. a) Representative western blot image showing results of co-immunoprecipitation of the CITF1-FIT complex. The CITF1-FIT protein complex was co-immunoprecipitated from 5-d-old *citf1-*1 mutant seedlings co-expressing CITF1-MYC and FIT-GFP constructs. Plants were grown in control or iron-, or copper-depleted hydroponic solutions for 5 d before tissue collection, protein extractions, co-IP with anti-MYC antibody, and western blot analysis using the anti-GFP and anti-MYC antibodies. An anti-actin antibody was used to detect actin, serving as a loading control. CITF1-MYC, FIT-GFP, and Actin migrated on SDS-PAGE as expected for their predicted molecular weights (∼63 kDa for CITF1-MYC (TAP), 63 to 72 kDa for FIT-GFP, and ∼42 kDa for Actin), as estimated using a pre-stained protein size standard (BioRad). (b) Quantification of the relative MYC/Actin signal ratio and GFP/Actin signal ratio of the input samples of the co-IP experiments. For each experiment, MYC or GFP intensities were normalized to Actin, and the MYC/Actin or GFP/Actin ratio of the control condition was set to 1, and the relative MYC/Actin or GFP/Actin values, compared with the control, were calculated. Values are mean ± S.E. (*n* = 3 independent experiments). c) The quantified intensity of anti-MYC and anti-GFP after anti-MYC pull-down and western blot analysis. The anti-MYC and anti-GFP intensities under −Fe or −Cu conditions were normalized to the same antibody intensities under control conditions, which were set to 1. Values are mean ± S.E. (*n* = 3 independent experiments). The relative signal intensities in (b) and (c) were determined using the Bio-Rad Image Lab software package. (d) The relative GFP-to-MYC ratios were calculated using the results shown in (c). Values are mean ± S.E. (*n* = 3 independent experiments). In (b) to (d), different lowercase letters indicate significant differences (*P* < 0.05; ANOVA followed by Tukey's HSD, JMP Pro 14 software package; asterisks indicate statistically significant differences of planned comparisons by ANOVA followed by Student's *t*-test (*P* < 0.05).

Given the established roles of CITF1 and FIT in Cu and Fe homeostasis, respectively, we next investigated whether the steady-state levels of the CITF1-FIT protein complex are influenced by Cu and/or Fe availability. We performed co-IP assays using *citf1*;35S*_pro_*:FIT-GFP + 35S*_pro_*:CITF1-MYC seedlings grown under control, Cu-deficient (−Cu), or Fe-deficient (−Fe) conditions (Fig. 2 and Figure S3a).

To directly account for changes in protein abundance across nutrient treatments (Fig. 2a), we quantified FIT-GFP and CITF1-MYC levels in the input fractions (Fig. 2b) and calculated input-normalized co-IP values (co-IP signal ÷ input signal) for both proteins, as well as their ratios (Fig. 2c, d, respectively) for each biological replicate (*n* = 3, Figure S3a). Although pairwise comparisons across all treatments did not reach statistical significance (ANOVA followed by Tukey HSD, *P* > 0.05), largely due to between-experiment variability in Western blot signal intensities (Figure S3a), the input-normalized anti-MYC and anti-GFP signals tended to increase under −Cu relative to the control, consistent with the direction of changes observed in the raw co-IP data (Fig. 2a, c and Figure S3a). Under Fe deficiency, input-normalized anti-MYC co-IP signal showed a downward trend compared with control, consistent with the raw co-IP data, and this difference was statistically significant (Fig. 2a, c, and Figure S3a). A planned comparison using a Student's *t*-test (*P* < 0.01) revealed that the relative abundance of the input-normalized FIT-CITF1 complex increased under −Cu but not under −Fe conditions relative to control (Fig. 2d). Taken together, these results support the cautious interpretation that Cu deficiency increases the abundance of the CITF1–FIT complex. The effect of Fe deficiency on the CITF1 protein stability and complex formation will require further investigation.

### The steady-state accumulation of native FIT protein is increased by copper deficiency in the presence of CITF1

**Figure 3:**
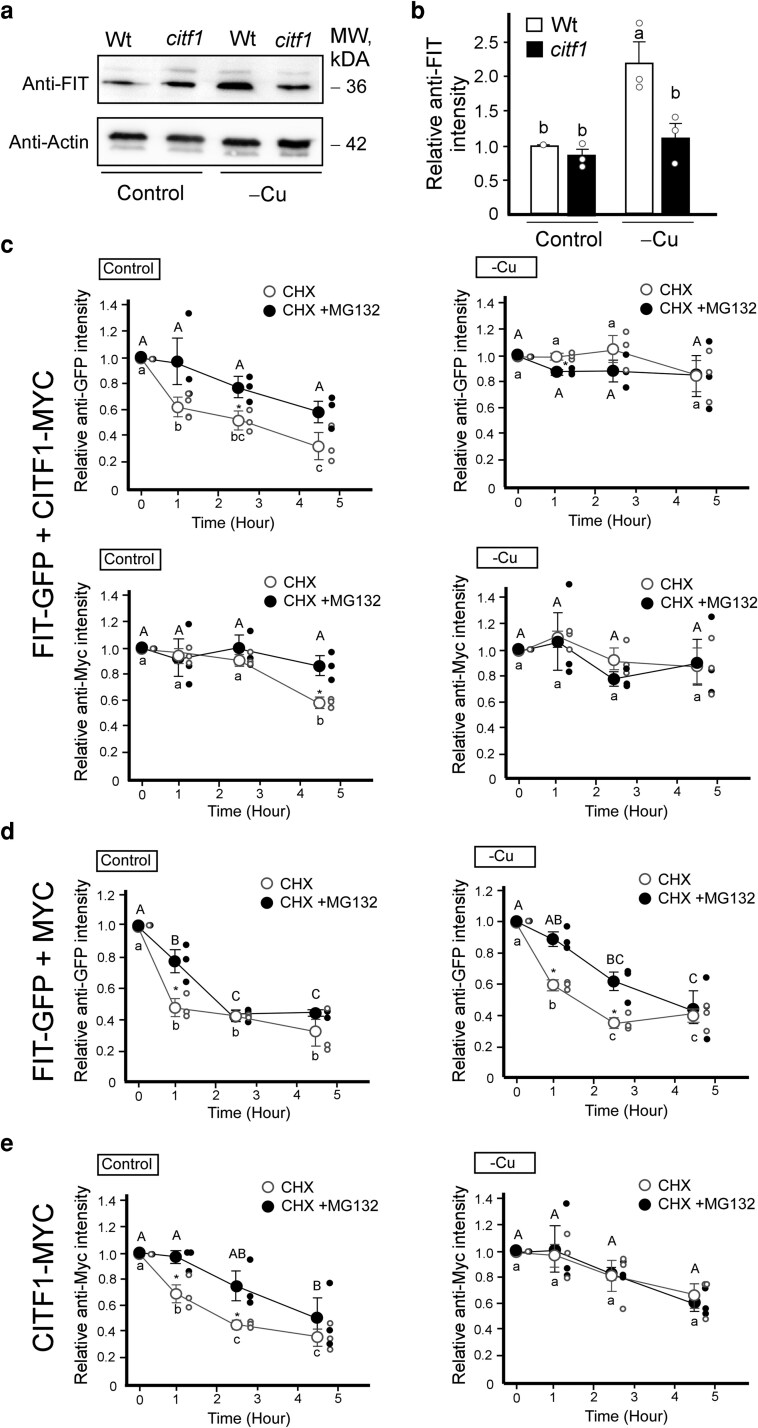
Copper deficiency and CITF1 delay FIT protein turnover. a) Immunoblot showing FIT protein levels in roots of 4-wk-old wild-type and *citf1* mutant plants grown hydroponically with or without 250 nm CuSO_4_ (control and − Cu, respectively). Molecular weights (kDa) for anti-FIT and anti-Actin immunoreactive bands are indicated on the right. b) Quantification of FIT signal intensity normalized to actin, based on 3 independent experiments. Signal intensities were quantified using Bio-Rad Image Lab. Values are mean ± SE. c to e) Quantification of the anti-MYC or anti-GFP antibodies signal intensities normalized to actin, based on 3 independent Western blot analyses of FIT-GFP and CITF1-MYC in *citf1*;*35S_pro_:FIT-GFP + 35S_pro_:CITF1-MYC* (c), *citf1*;*35S_pro_:FIT-GFP + 35S_pro_:MYC* (d), and *citf1*;*35S*_pro_*:CITF1-MYC* (e) seedlings. In (c) to (e), plants were grown hydroponically for 5 d with or without 250 nm CuSO_4_ (control and **−**Cu, respectively) before being treated with 355 *µ*m CHX or 355 *µ*m CHX + 100 *µ*m MG-132 (CHX or CHX + MG132, respectively) for the indicated time. In (b) to (e) different lowercase letters indicate significant differences (*P* < 0.05; ANOVA followed by Tukey's HSD, JMP Pro 17 software package; asterisks indicate statistically significant differences of planned comparisons by ANOVA followed by Student's *t*-test (*P* < 0.05).

Previous studies, together with our observations, indicate that the transcript abundance of *FIT* in *A. thaliana* roots is not significantly affected by Cu deficiency (Figure S3b) (Bernal et al. 2012; Kastoori Ramamurthy et al. 2018). In contrast, the expression of *CITF1* is strongly upregulated in the roots of Cu-deficient plants (Figure S3b) and (Yan et al. 2017). Given that Cu deficiency enhances the abundance of the CITF1-FIT complex (Fig. 2), we hypothesized that Cu deficiency may also influence the accumulation of FIT protein. Supporting this hypothesis, levels of endogenous FIT protein were markedly elevated in the roots of wild-type plants grown under Cu-deficient conditions (Fig. 3a, b). However, this increase was not observed in the *citf1-*1 mutant under the same conditions (Fig. 3a, b), suggesting that CITF1 contributes to FIT protein stability during Cu deficiency.

### CITF1 stabilizes the FIT-GFP recombinant protein under copper deficiency

To investigate the role of CITF1 in FIT protein stability and to elucidate the mechanism regulating FIT degradation under varying Cu conditions, we compared the turnover of FIT-GFP expressed either with or without CITF1 in *35S_pro_:FIT-GFP + 35S_pro_:CITF1-MYC* and *35S_pro_:FIT-GFP + 35S_pro_:MYC* plants. To monitor protein stability independently of de novo synthesis, we treated seedlings with the translation inhibitor cycloheximide (CHX). In control conditions, CITF1 protein levels showed a moderate decrease after 4.5 h of CHX treatment in *35S_pro_:FIT-GFP + 35S_pro_:CITF1-MYC* plants, whereas CITF1 remained stable under Cu-deficient conditions (Fig. 3c and Figure S4a). The stability of CITF1 was further enhanced in the presence of the proteasome inhibitor MG132, suggesting proteasome-mediated degradation.

FIT-GFP abundance declined after 1 h of CHX treatment and continued to decrease over time in control-grown plants. This degradation was mitigated by MG132, consistent with previous reports (Fig. 3c and Figure S4a; (Lingam et al. 2011)). Under control conditions, FIT turnover was similar in the presence or absence of CITF1, as indicated by comparable FIT-GFP decline in *35S_pro_:FIT-GFP + 35S_pro_:CITF1-MYC* and *35S_pro_:FIT-GFP + 35S_pro_:MYC* lines (Fig. 3c, d and Figure S4a). In contrast, under Cu deficiency, FIT-GFP exhibited differential stability depending on CITF1 co-expression. FIT-GFP levels remained more stable in Cu-deficient *35S_pro_:FIT-GFP + 35S_pro_:CITF1-MYC* plants following CHX treatment, whereas a marked decline was observed in *35S_pro_:FIT-GFP + 35S:*MYC plants (Fig. 3c, d and Figure S4a). In both genotypes, FIT-GFP degradation was delayed by MG132, indicating a proteasome-dependent pathway.

Taken together, these results suggest that both CITF1 and FIT are stabilized under Cu deficiency and that CITF1 is required for FIT protein stability in these conditions. Moreover, our data demonstrate that CITF1-MYC and FIT-GFP turnover under control conditions, as well as FIT-GFP degradation triggered by Cu deficiency in the absence of CITF1, are mediated by the proteasome.

### The CITF1 protein is stabilized by copper deficiency

We next examined CITF1 protein turnover using transgenic *citf1-*1 mutant plants expressing *35S_pro_:CITF1-MYC.* Upon CHX treatment, CITF1-MYC levels declined within 2.5 h under control conditions (Fig. 3e and Figure S4b, c). In contrast, under Cu-deficient conditions, CITF1-MYC remained relatively stable, with no notable degradation observed until 4.5 h after CHX treatment. In both conditions, CITF1-MYC degradation was inhibited by MG132, indicating the involvement of the proteasome pathway (Fig. 3e and Figure S4b, c). We note that in the *35S_pro_:CITF1-MYC* + *35S_pro_:FIT-GFP* co-expression experiments (Fig. 3c and Figure S4a), CITF1 protein decay appears less pronounced than in the *citf1* mutant background with endogenous *FIT* levels (Fig. 3e and Figure S4). *FIT* overexpression elevates *CITF1* transcript levels (Figure S5) and may also stabilize the protein, resulting in higher overall CITF1 abundance that masks degradation dynamics. By contrast, when *CITF1* is expressed in the presence of endogenous FIT, CITF1 degradation is more readily detectable (Fig. 3e and Figure S4b, c). Together, these results demonstrate that Cu deficiency delays CITF1 turnover and that, like FIT, CITF1 stability is regulated by proteasome-mediated degradation.

### FIT positively regulates Cu uptake genes under Cu but not Fe deficiency through interacting with CITF1

**Figure 4:**
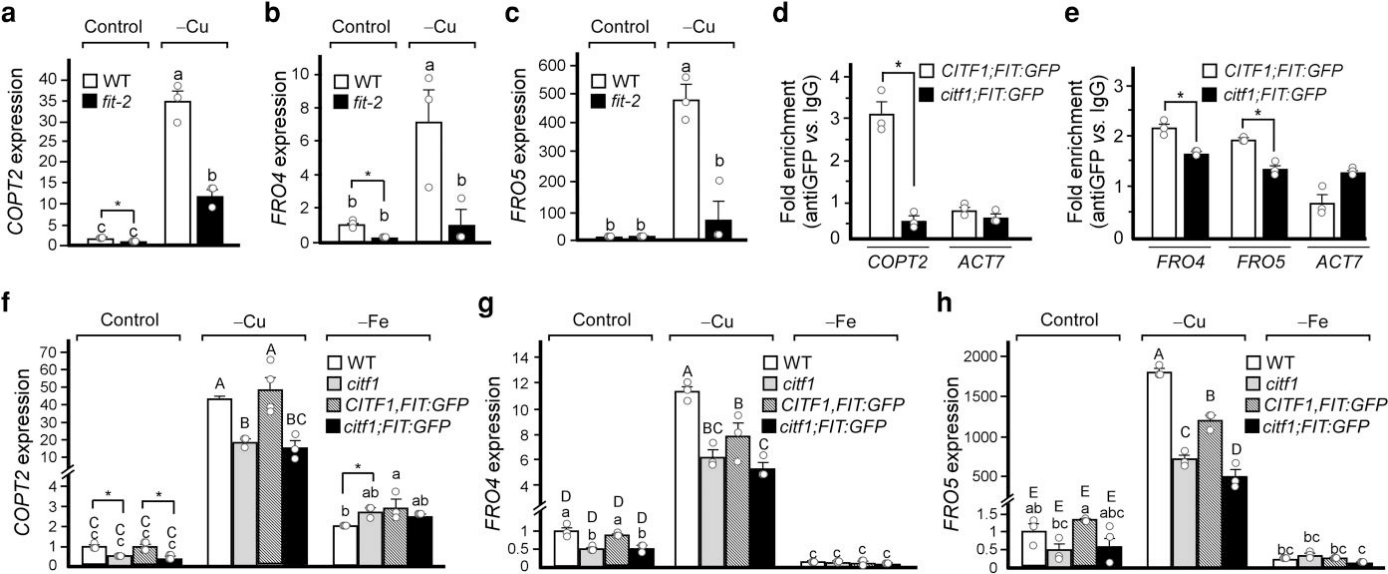
CITF1 and FIT coregulate the expression of copper uptake genes. (a to c) Transcript levels of *COPT2*, *FRO4,* and *FRO5* in roots of wild-type and the *fit-2* mutant grown hydroponically with or without 250 nm CuSO_4_ (**control**, **−Cu**, respectively). d, e) ChIP-qPCR showing FIT-GFP binds to *COPT2*, *FRO4*, and *FRO5* promoters in CITF1;35S*pro*:FIT-GFP but not 35S*pro*:FIT-GFP plants grown under Cu deficiency. ACT7 served as a negative control. Values are mean ± SD (*n* = 3 technical replicates) of fold enrichment using the anti-MYC antibody *vs.* mouse IgG. Asterisks indicate statistically significant differences between groups (*P* < 0.05, Student's *t*-test). f to h) The transcript abundance of *COPT2*, *FRO4,* and *FRO5* in the wild-type, *citf1* mutant, CITF1;35S*pro*:FIT-GFP, and *citf1*;35S*pro*:FIT-GFP plants grown hydroponically for 4 wk with 250 nm CuSO_4_ and 10 *µ*m Fe-HBED (**control**) or 10 *µ*m Fe-HBED and 0 nm CuSO_4_ (**−Cu**), or 250 nm CuSO_4_ and 1 *µ*m Fe-HBED (**−Fe**). In (a to c) and (f to h), values are means ± SE (*n* = 3 to 4 independent experiments with the roots of 4 plants pooled together per experiment). Different lowercase letters indicate significant differences (*P* < 0.05; ANOVA, followed by Tukey HSD, JMP Pro 15 software package). Asterisks indicate planned Student's *t*-test comparison of gene expression within the same conditions (*P* < 0.05).

Since CITF1 regulates the expression of *COPT2*, *FRO4,* and *FRO5* under Cu deficiency (Yan et al. 2017), we next asked whether this CITF1 function requires FIT. As reported previously, *COPT2*, *FRO4*, and *FRO5* transcripts were significantly upregulated in the roots of Cu-deficient wild-type plants (Fig. 4a to c) and (Bernal et al. 2012; Yan et al. 2017). However, their expression was markedly reduced in the *fit-*2 mutant under Cu deficiency, indicating that FIT is required for the full activation of these CITF1 targets (Fig. 4a to c). Despite this reduction, transcript level of *COPT2* in *fit-*2 remained elevated under Cu deficiency compared with control conditions, suggesting that additional FIT-independent mechanisms contribute to their regulation. Notably, *COPT2* and *FRO4* expression was also lower in *fit-*2 roots under control conditions, indicating a role for FIT in maintaining basal expression of Cu uptake genes (Fig. 4a to c).

To test whether FIT directly binds to the promoters of *COPT2*, *FRO4*, and *FRO5* under Cu deficiency, and whether this binding depends on CITF1, we performed chromatin immunoprecipitation followed by qPCR (ChIP-qPCR) using transgenic plants expressing *35S_pro_:FIT-GFP* in the *citf1-*1 mutant or wild-type backgrounds (*citf1;35S_pro_:FIT-GFP* and *CITF1;35S_pro_:FIT-GFP*, respectively). Notably, *CITF1* expression was elevated in *CITF1;35S_pro_:FIT-GFP* plants (Figure S5). Chromatin from Cu-deficient seedlings was immunoprecipitated using an anti-GFP antibody. FIT-GFP was enriched at the promoters of *COPT2*, *FRO4*, and *FRO5* in *CITF1;35S:FIT-GFP* plants, but not in *citf1;35S_pro_:FIT-GFP* plants, suggesting that CITF1 is required for FIT promoter binding (Fig. 4d, e).

Consistent with these results, the expression of *COPT2* was strongly induced in roots of wild-type and *CITF1;35S_pro_:FIT-GFP* plants, and was significantly reduced, though still above control levels, in *citf1-*1 or *citf1*;*35S_pro_:FIT-GFP* plants under Cu deficiency (Fig. 4f). Similarly, *FRO4* and *FRO5* transcript levels were higher in wild-type and *CITF1;35S_pro_:FIT-GFP* roots compared with the *citf1-*1 mutant and *citf1*;*35S_pro_:FIT-GFP* lines (Fig. 4g, h). By contrast, CITF1 was not required for FIT-mediated activation of *COPT2* under Fe deficiency; in fact, *COPT2* expression was slightly elevated in *citf1-*1 under these conditions (Fig. 4f). Fe deficiency did not significantly affect *FRO4* and *FRO5* expression under our experimental conditions. Together, these results show that FIT cooperates with CITF1 to regulate the expression of Cu uptake genes specifically under Cu deficiency, but not under Fe deficiency.

### CITF1 represses the transcriptional iron deficiency response

**Figure 5:**
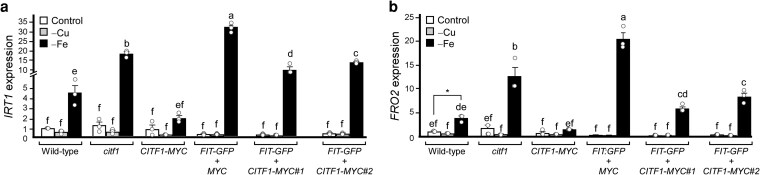
*CITF1* overexpression attenuates Fe deficiency responses. Transcript levels of *IRT1* (a) and *FRO2* (b) in seedlings of the indicated genotypes grown under control, −Cu, or −Fe conditions for 5 d. Data are means ± SD (*n* = 3). Different letters indicate significant differences (ANOVA + Tukey HSD). Asterisks indicate significant differences (*P* < 0.05, Student's *t*-test).

We next tested whether CITF1 is required for FIT-mediated regulation of the Fe deficiency response. We measured *IRT1* and *FRO2* expression in seedlings of wild-type, the *citf1-*1 mutant, and *citf1-*1 mutant expressing *35S_pro_:CITF1-MYC* or *35S_pro_:FIT-GFP*, or both, all grown under control, −Cu, or −Fe conditions. As expected, *IRT1* and *FRO2* were induced only by Fe deficiency in all genotypes (Fig. 5). Notably, *IRT1* and *FRO2* expression was significantly higher in Fe-deficient *citf1-*1 and *35S_pro_:FIT-GFP* seedlings compared with wild-type (Fig. 5). In contrast, *IRT1* and *FRO2* expression was reduced in Fe-deficient seedlings overexpressing CITF1, either alone or together with FIT. These results suggest that CITF1 negatively regulates Fe deficiency responses.

### CITF1 interferes with the formation of FIT-bHLH038, FIT-bHLH039, FIT-bHLH100, and FIT-bHLH101 heterodimers

We then used the yeast 3-hybrid (Y3H) system to test the hypothesis that CITF1 negatively regulates Fe deficiency responses by sequestering FIT and preventing it from binding to its other partners, *eg,* bHLH038, bHLH039, or bHLH100, and/or bHLH101. The Y3H system uses the Y2H pGADT7 vector with the GAL4 TF activation domain (AD) fused to the prey protein, the pBridge vector with GAL4 DNA binding domain (BD) fused to the bait protein, and an additional expression cassette for the third, conditionally expressed protein, which can either facilitate or disrupt prey and bait protein interactions (Tirode et al. 1997; Glass and Takenaka 2018).

We expressed FIT in the pGADT7 while bHLH38, bHLH39, bHLH100, or bHLH101 were in the pBridge vector. pBridge also carried *CITF1* under the control of the *MET25* promoter, which is repressed by methionine. We expected that if CITF1 disrupts interactions between FIT and bHLH38/39/100 or bHLH101, the presence of methionine would repress *CITF1* expression, so CITF1 would not interfere with FIT's ability to bind to group Ib bHLHs. By contrast, weak or lost interactions will be manifested in the methionine-free medium, as *CITF1* would be expressed. We also generated a pBridge vector that, instead of *CITF1,* contained *MBP* (maltose-binding protein) under the control of *MET25pro*. This would allow us to test the specificity of CITF1-mediated disruption of the FIT-HLH38/39/100/101 interaction relative to MBP.

**Figure 6:**
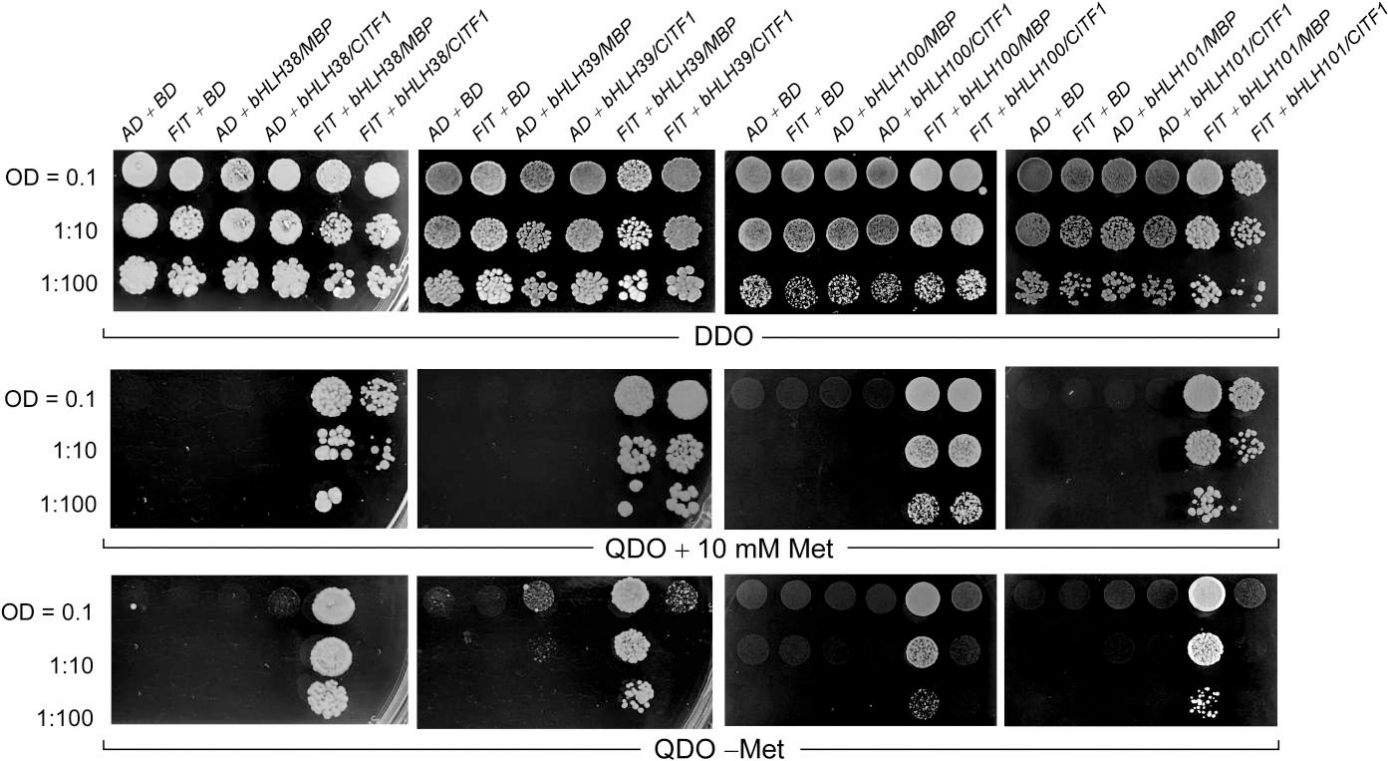
CITF1 disrupts FIT-bHLH38/39/100/101 interactions. Serial dilutions of yeast co-expressing AD-FIT and BD-bHLH38 or BD-bHLH39, or BD-bHLH100 or BD-bHLH101 with MET25p-driven *CITF1* or *MBP* were spotted on double dropout medium (lacking Leu/Trp, DDO, control). Cells were also spotted onto quadruple dropout medium lacking Leu/Trp/His/Ade (QDO) and either containing 10 mm methionine (to repress CITF1 or MBP) or lacking methionine (to induce *CITF1* or *MBP*). FIT–bHLH38/39/100/101 interactions are maintained in + Met and −Met when *MBP* is expressed but are abolished in −Met when *CITF1* is expressed, indicating that CITF1 specifically disrupts these heterodimerizations. Bait and prey vectors lacking cDNA inserts (BD and AD, respectively) were used as additional controls.

Consistent with our expectations, FIT interacted with bHLH38/30/100/101 in a quadruple dropout medium containing methionine (Fig. 6 and Figure S6). The expression of *CITF1* but not *MBP* in the medium without methionine eliminated FIT-bHLH38, FIT-bHLH39, FIT-bHLH100, and FIT-bHLH101 interactions (Fig. 6). Thus, we concluded that CITF1 could negatively regulate Fe-deficiency responses by disrupting the interactions between FIT-bHLH38/39/100/101.

### CITF1 co-localizes with FIT in the nucleus in *A. thaliana* protoplasts

**Figure 7:**
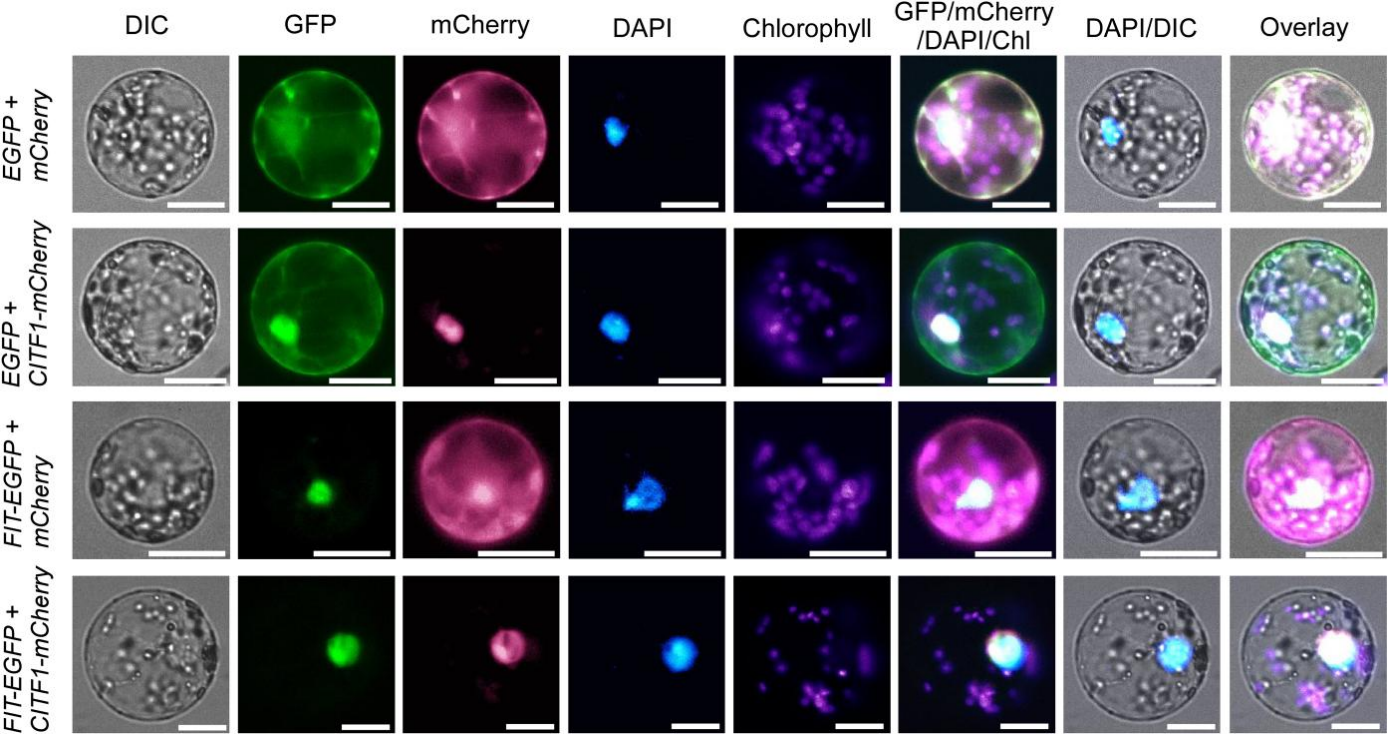
CITF1 and FIT co-localize to the nucleus in *Arabidopsis thaliana* protoplasts. Protoplasts isolated from the wild-type leaves were co-transfected with the following construct combinations: (1) empty vectors expressing EGFP and mCherry (EGFP + mCherry), (2) EGFP + CITF1, fused at the C-terminus with mCherry (EGFP + CITF1-mCherry), (3) mCherry + FIT, fused at the C-terminus with EGFP (mCherry + FIT–EGFP), and (4) FIT-EGFP + CITF1-mCherry. Protoplasts were also stained with DAPI (4′,6-diamidino-2-phenylindole) to assess the protein overlap with the nuclear marker. GFP, mCherry, DAPI, and chlorophyll autofluorescence signals were imaged separately. Overlay images confirm that FIT-EGFP and CITF1-mCherry co-localize in the nucleus. Bar = 20 *µ*m. All transfected protoplasts (transfection efficiency ∼80%) exhibited the same localization pattern. A representative image from the 18 documented protoplasts is shown. In addition, Figure S7 presents the nuclear localization of FIT-EGFP in protoplasts prepared from transgenic plants overexpressing *CITF1*.

CITF1 localizes to the nucleus in *A. thaliana* protoplasts (Yan et al. 2017), whereas FIT partitions between the cytoplasm and nucleus and can influence the nuclear localization of bHLH039 (Gratz et al. 2019; Trofimov et al. 2019). To determine whether CITF1 affects the subcellular localization of FIT, we co-expressed *35S_pro_:FIT-EGFP* of the pSAT vector with either *35S_pro_:CITF1-mCherry* or empty vectors in mesophyll protoplasts isolated from *A. thaliana* wild-type. FIT localized predominantly to the nucleus, and its distribution was not altered by the presence of CITF1 (Fig. 7). These results suggest that while CITF1 can interfere with FIT interactions with other bHLH proteins, it does not affect FIT subcellular localization. Similar results were obtained when *35S_pro_:FIT-EGFP* was co-expressed with *35S_pro_:CITF1-MYC* in protoplasts isolated from the *citf1-*1 mutant (Figure S7).

### The *citf1-*1 mutant is less sensitive to Fe deficiency than the wild-type

**Figure 8:**
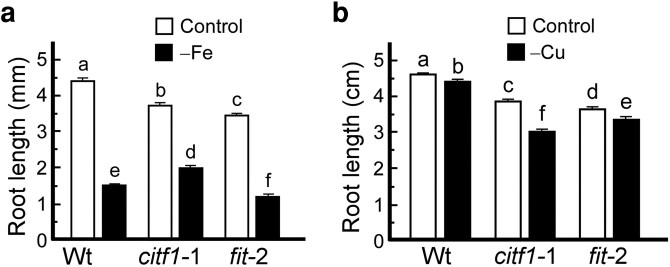
The *citf1-*1 and *fit* mutants display distinct nutrient-deficiency responses. a) shows the root length of wild-type, *citf1-*1, and *fit-*2 mutants grown on the 1/2 MS medium containing 50 *µ*m FeSO_4_-EDTA and 5 *µ*m CuSO_4_ (control). To achieve Fe deficiency, Fe was not added to the medium. b) Plants were grown as described in (a). To induce Cu deficiency, 100 *µ*m of the Cu chelator BCS was added instead of Cu. In (a) and (b), values are mean values ± SE (*n* = 50 roots of seedlings from 10 plates per genotype per treatment). Different lowercase letters indicate significant differences (*P* < 0.05; ANOVA followed by Tukey HSD).

Given that CITF1 appears to act as a negative regulator of the Fe-deficiency response, we asked whether its loss affects basal tolerance to Fe limitation. To test this, we compared root growth of wild-type, *citf1-*1, and *fit-*2 plants grown on ½ MS solid medium with or without Fe supplementation. Under control conditions, *citf1-*1 roots were shorter than wild-type roots, consistent with their lower Cu levels, as we reported previously (Yan *et al* 2017) and (Fig. 8a). In contrast, under Fe-deficient conditions, *citf1-*1 roots were longer than those of wild-type or *fit-*2 plants (Fig. 8a), consistent with CITF1 acting as a negative regulator of Fe responses. These findings further support a role for CITF1 in modulating the Fe-deficiency response.

### The *fit-*2 mutant is more sensitive to copper deficiency than wild-type

Considering that FIT positively regulates Cu uptake genes under Cu deficiency through interacting with CITF1 (Fig. 4a to c), we next tested the sensitivity of the *fit-*2 mutant to Cu deficiency. We compared the root growth of *fit-*2 plants with wild-type and the *citf1* mutant grown on ½ MS solid medium with or without the Cu chelator, BCS (bathocuproine disulfonate). We found that the roots of the Cu-deficient *fit-*2 mutant were shorter than the roots of the wild-type. As evidenced by the smaller rosette size of the *fit-*2 mutant, it was also more sensitive to Cu deficiency than wild-type when grown hydroponically (Figure S8). As expected, *citf1-*1 mutant roots were shorter than wild-type and *fit-*2 roots under Cu deficiency (Fig. 8b). Together, these results further support the conclusion that both CITF1 and FIT are positive regulators of Cu deficiency response, with CITF1 playing a more dominant role.

### Loss of both CITF1 and FIT in the c*itf1*-1 *fit-*2 double mutant causes embryolethality

**Figure 9:**
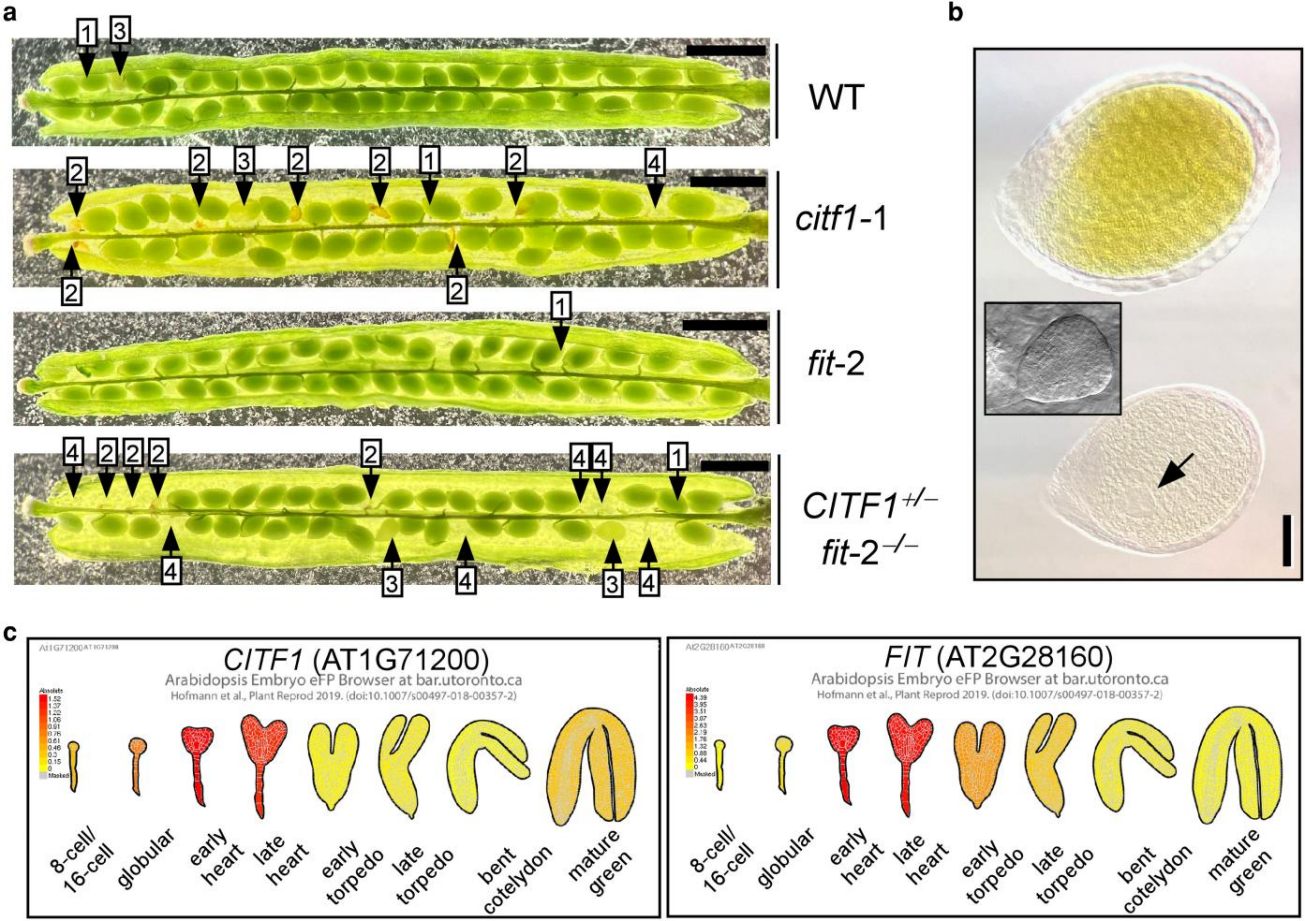
The *citf1*-1 *fit*-2 double mutant is embryo-lethal. a) shows representative images of siliques collected from soil-grown wild-type, homozygous *citf1-*1, *fit-2* single mutants, and *CITF1^+/−^ fit^−/−^* double mutant. Arrows indicate healthy green developing seeds (1), aborted shrunken and brown seeds (2), pale green or milky-white seeds that later turn brown (3), and unfertilized ovules (4). b) A representative DIC image of a green seed and a milky-white seed from *CITF1^+/−^ fit^−/−^* mutant. Developing embryos from at least 10 siliques of the *CITF1^+/−^ fit^−/−^* mutant were analyzed. The arrow indicates an aborted embryo, as further magnified in a DIC inset. Scale bars = 50 *µ*m. c) In silico analyses of *CITF1* and *FIT* expression in developing embryos using the electronic fluorescent pictograph (eFP) browser (Winter et al. 2007).

We next crossed the *citf1-*1 knockout allele with the *fit-*2 mutant to investigate how this protein complex influences the response to changes in Fe and Cu status. To ensure Fe- and Cu-sufficient growth conditions, 0.5 g/L Sequestrene and 50 *µ*m CuSO_4_ were added to the soil to rescue potential deficiency phenotypes caused by *FIT* and *CITF1* knockouts (Colangelo and Guerinot 2004; Yan et al. 2017). The PCR-based genotyping of at least 50 F2 plants did not identify the double mutant homozygous for both *citf1-*1 and *fit-*2 alleles, suggesting that the double mutation was lethal at some point either during gametogenesis or embryogenesis. To establish the developmental stage affected by the simultaneous loss of CITF1 and FIT function, we analyzed the segregation pattern of the *citf1-*1 allele from plants homozygous for the *fit-*2 mutation and heterozygous for the *citf1-*1 mutation (*CITF1^+/-^ fit-2 ^−/-^)*. In addition to green, developing seeds found in all genotypes, we observed defective seeds, randomly distributed along the siliques in the *CITF1^+/-^ fit-2 ^−/-^* mutant (Fig. 9a). Some ovules failed to develop or were unfertilized, whereas others were arrested at early developmental stages and subsequently shriveled. By the greening stage, more defective seeds were readily distinguishable as milky-white, in contrast to the green seeds observed in wild-type. Differential interference contrast microscopy comparison of green *vs.* milky-white seeds in *CITF1^+/-^ fit-2^−/-^* plants revealed that milky-white embryos were at the early heart stage of development (Fig. 9b and Figure S9). These abnormal seeds eventually desiccated and became brown, resembling previously described embryo-lethal phenotypes (Meinke and Sussex 1979; Stacey et al. 2002). *In silico* expression analyses identified both *CITF1* and *FIT* as strongly expressed during early to late heart stages of embryogenesis (Fig. 9c and (Winter et al. 2007; Hofmann et al. 2019)), supporting the function of these genes in embryo development.

Quantification revealed that defective seeds comprised ∼28% of developing seeds in *CITF1^+/-^ fit-2 ^−/-^* siliques, consistent with a 3:1 segregation ratio of normal to defective seeds (Table 1). Interestingly, *citf1-*1 single mutants also displayed a mild increase in defective seeds (10%) compared with wild-type or *fit-*2 mutants (both ∼4%) (Table 1). Consistent with embryonic lethality, none of the 88 PCR-genotyped progeny from *CITF1^+/-^ fit-2 ^−/-^* plants were double homozygous for both *citf1-*1 and *fit-*2. Together, these data support a previously unrecognized role for CITF1 and FIT in embryo development.

**Table 1:** Number of green seeds *vs.* defective seeds per silique in wild type, *fit* and *citf1* mutants, and *citf1^+/−^ fit^−/−^* double mutant.

Genotype	Total siliques analyzed	Number of green seeds per silique	Number of defective seeds per silique	Ratio of defective seeds (%)
Wild type	14	40.6 ± 0.92^a^	1.9 ± 0.36^c^	4.3 ± 0.83^c^
*fit*	13	43.8 ± 1.92^a^	1.7 ± 0.44^c^	3.8 ± 0.94^c^
*citf1*	23	35.5 ± 0.76^b^	4.9 ± 0.66^b^	11.9 ± 1.48^b^
*citf1^+/−^ fit^−/−^*	31	33.1 ± 1.00^b^	12.9 ± 0.66^a^	28.1 ± 1.37^a^

The plants were grown in soil supplemented with 0.5 g/L Sequestrene weekly. Different lowercase letters (a to c) indicate significant differences (*P* < 0.05; Tukey HSD, JMP Pro 15).

**Figure 10:**
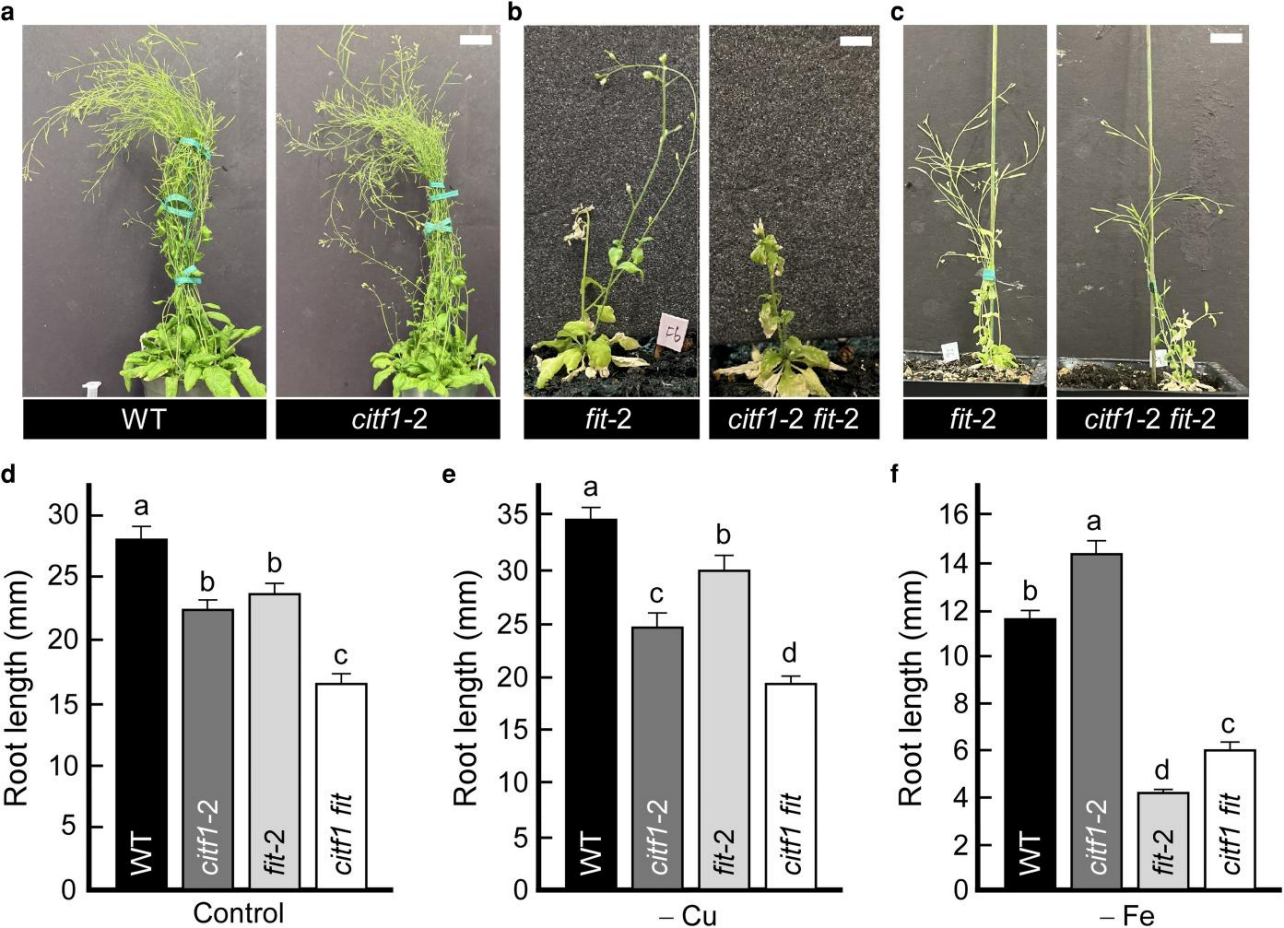
The *citf1-*2 *fit-*2 double mutant shows reduced growth under control conditions and altered sensitivity to copper and iron deficiency. a) Representative images of 10-wk-old wild-type and *citf1-*2 mutant plants grown in soil and fertilized with NPK. b, c) Representative images of 8-wk-old (b) and 11-wk-old (c) *fit-2* single and *citf1-*2 *fit-*2 double mutants grown in soil with NPK plus 50 mg/L Sequestrene 330 applied biweekly. d to f) Primary root length of 10-d-old seedlings grown vertically on 1/2 MS agar plates under control conditions containing 50 *µ*m FeSO_4_-EDTA and 5 *µ*m CuSO_4_ (d), under Cu deficiency induced by replacing Cu with 100 *µ*m BCS (e), or under Fe deficiency by omitting Fe (f). After 10 d, plates were photographed, and root length was measured using ImageJ. Values are means ± SE (*n* = 45 to 50 roots per genotype per treatment, pooled from 10 plates). Different lowercase letters indicate significant differences (*P* < 0.05; ANOVA with Tukey's HSD).

To further assess whether *CITF1* dosage affects embryo viability, we crossed *fit-*2 with a hypomorphic *CITF1* knockdown allele, *citf1*-2 (Yan et al. 2017). The resulting *citf1-*2 *fit-*2 double mutant (Figure S10a) did not show seed development defects but exhibited delayed inflorescence emergence and smaller stature compared with the *citf1-*2 and *fit-*2 single mutants (Fig. 10a to c). These findings indicate that CITF1 is broadly important for reproductive growth, and residual *CITF1* expression in *fit-2 citf1-2* double mutants is sufficient to support normal embryogenesis.

### Loss of CITF1 and FIT in the *citf1-*2 *fit-*2 double mutant differentially affects root growth under control, Cu- and Fe-deficient conditions

We next used the *citf1-*2 *fit-*2 double mutant to investigate genetic interactions between *CITF1* and *FIT* under varying metal availabilities using in-root growth regulation assays. As we showed for the *citf1-*1 allele (Fig. 8a), under control conditions, wild-type roots were significantly longer than those of *citf1-*2 or *fit-*2 single mutants, which displayed similar moderately reduced root lengths (Fig. 10d and Figure S10b). The *citf1-*2 *fit-*2 double mutant exhibited the shortest roots among all genotypes, suggesting additive effects of the mutations under nutrient sufficiency. Consistent with our findings for the *citf1-*1 mutant allele in this manuscript (Fig. 8a) and Yan et al. (2017), the *citf1-*2 mutant was also more sensitive to Cu deficiency than both the wild-type and the *fit-*2 mutant (Fig. 10e, Figure S10b). Likewise, the *fit-*2 mutant was more sensitive to Cu deficiency than wild-type but less sensitive than *citf1-*2. The *citf1-*2 *fit-*2 double mutant was most sensitive to Cu deficiency, as evidenced by its shorter roots, even compared with *citf1-*2 alone (Fig. 10e and Figure S10b). These results suggest that *CITF1* and *FIT* may contribute, in part, independently to root growth under Cu deficiency. While *CITF1* appears to play a more prominent role, the enhanced sensitivity of the double mutant suggests a partially additive interaction or a parallel pathway to maintain root growth under Cu deficiency. These data are consistent with both CITF1 and FIT acting as positive regulators of Cu homeostasis.

Under Fe deficiency, all genotypes showed reduced root lengths compared with the control. The *fit-*2 mutant exhibited the most severe reduction, consistent with its essential role in Fe acquisition. Consistent with our previous results for the *citf1-*1 allele, *citf1-*2 mutant roots were slightly longer than wild type, supporting a role for CITF1 as a negative regulator of Fe deficiency responses (Figs 8a, 10f, and Figure S10b). The *citf1-*2 *fit*-2 double mutant root lengths were significantly shorter than *citf1-*2 alone but longer than *fit*-2 mutant, indicating that *CITF1* loss partially alleviates the Fe deficiency growth defect of *fit-*2 mutation. We interpret these data to mean that while *FIT* functions as a positive regulator of Fe acquisition, *CITF1* acts as a negative regulator, and the 2 genes interact genetically such that *CITF1* knockdown can mitigate the phenotypic consequences of *FIT* deficiency.

### CITF1 contributes to Fe accumulation under Cu deficiency

**Figure 11:**
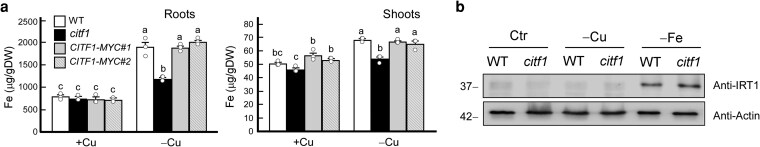
The *citf1-*1 mutant accumulates less iron under copper deficiency. a) Iron concentration in roots and shoots of wild-type and the *citf1-*1 (citf1) plants, grown hydroponically either with 10 *µ*m Fe-HBED and 250 nm Cu (**control**) or with 10 *µ*m Fe-HBED and no added copper (−Cu) for 4 wk before roots and leaves were collected for ICP-MS analysis. Values are mean ± SE (*n* = 3 independent experiments with samples pooled from 4 plants per experiment). Levels not connected by the same letter are statistically different (ANOVA followed by Tukey HSD, JMP Pro 15 software package). Asterisks indicate statistically significant differences between groups (*P* < 0.05, Student's *t*-test). b) Western blot analysis of IRT1 protein levels in wild-type and the *citf1-*1 mutant, grown as described in (a). For Fe-deficiency treatment (−Fe), plants were grown in a control hydroponic solution for 3 wk before being transferred to a new hydroponic medium lacking Fe. Roots were collected after 1 wk of growth for the Western blot analysis. Nitrocellulose filters, probed with the anti-IRT antibody, confirmed the accumulation of IRT1 under iron deficiency. The anti-Actin antibody was used as an internal control. The molecular weights of IRT1 and Actin (kDa, indicated on the left) were estimated based on their migration relative to the molecular weight ladder (Bio-Rad) and are consistent with their predicted sizes.

Our previous work demonstrated that the increased Cu-deficiency sensitivity of the *citf1-*1 mutant stems from its impaired Cu uptake (Yan et al. 2017). Here, we also tested if the loss of *CITF1* would affect Fe levels in plant tissues. Consistent with previous reports, Fe concentration in both roots and shoots of wild-type plants was higher under Cu-deficiency compared with control conditions (Fig. 11a and (Waters and Armbrust 2013; Kastoori Ramamurthy et al. 2018; Chia et al. 2023)). In contrast, Fe concentration did not increase in the shoots of the *citf1-*1 mutant under Cu deficiency and was significantly lower in its roots compared with wild-type and *35S_pro_:CITF1-MYC* plants grown under the same conditions (Fig. 11a). These data suggested that CITF1, in addition to regulating Cu uptake, also contributes to Fe uptake under Cu deficiency.

Because *IRT1* and *FRO2* are not transcriptionally induced by Cu deprivation (Fig. 5 and Chia et al. 2023), Fe transport under Cu deficiency appears independent of this system. Consistent with this, IRT1 protein accumulated only under Fe deficiency in roots of both wild-type and *citf1-*1 plants and remained weakly detectable under control or Cu-deficient conditions (Fig. 11b).

## Discussion

The dual, essential, and toxic nature of Cu and Fe requires the precise regulation of their cognate uptake systems to ensure normal plant growth. Because of similar physical and chemical properties, it is not surprising that notable physiologic interactions between Cu and Fe have been described in both plant and animal cells (Gulec and Collins 2014). For example, in *A. thaliana*, Cu deprivation enhances Fe uptake and vice versa, even though these metals cannot substitute each other fully for the completion of the plant life cycle (Waters and Armbrust 2013; Kastoori Ramamurthy et al. 2018; Cai et al. 2021; Sheng et al. 2021; Chia et al. 2023). Fe limitation upregulates Cu acquisition via COPT2, controlled by FIT in partnership with bHLH38/39/100/101 (Cai et al. 2021). Yet, the molecular basis for increased Fe uptake under Cu deficiency or Cu-deficiency transcriptional networks remained largely uncharacterized. Indeed, SPL7 and CITF1 are the only 2 TFs with a documented role in Cu homeostasis (Yamasaki et al. 2009; Bernal et al. 2012; Yan et al. 2017). Recently, *bHLH23* (alias *CITF2*) was identified as a direct SPL7 target, but its role in Cu homeostasis has not yet been established (Schulten et al. 2022).

**Figure 12:**
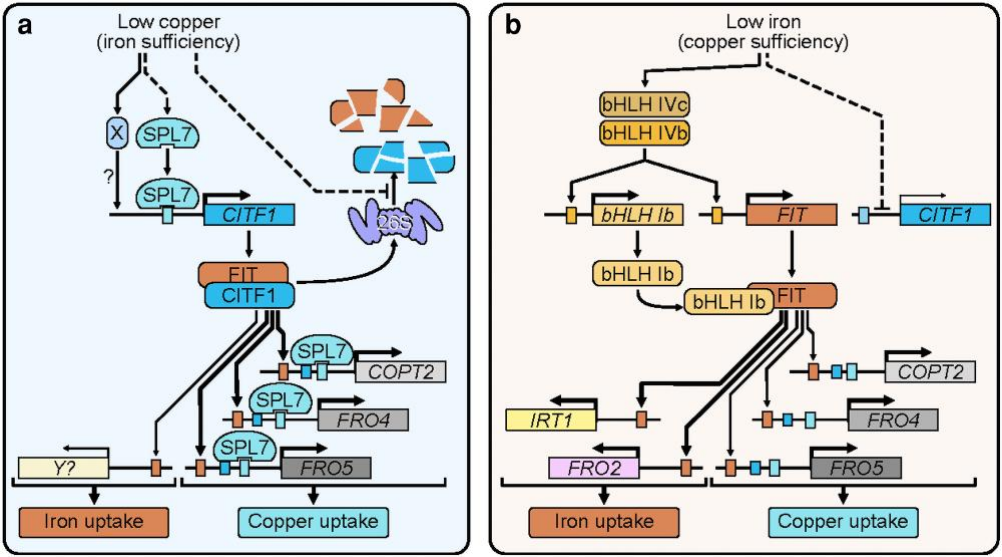
A proposed model for the CITF1-FIT-mediated crosstalk between Cu and Fe homeostasis. Under Cu deficiency, *CITF1* is upregulated by SPL7 and other unknown regulatory pathways (Yan et al. 2017). At the post-translational level, CITF1 physically interacts with FIT to facilitate Cu uptake by upregulating the expression of *COPT2*, *FRO4*, and *FRO*5. Copper deficiency slows proteasome-mediated degradation of CITF1 and FIT, leading to accumulation of the CITF1-FIT complex. Under Fe deficiency, *CITF1* is transcriptionally downregulated, releasing FIT for subsequent interaction with clade Ib bHLH members. FIT-bHLH Ib interaction promotes *IRT1*/*FRO2* and *COPT2* expression for proper iron deficiency response.

In this study, we used molecular, biochemical, and genetic analyses to uncover a partner-switching mechanism by which CITF1 dynamically modulates FIT function to coordinate Cu and Fe homeostasis in *A. thaliana* (Fig. 12).

### FIT interacts with CITF1 to positively regulate Cu deficiency responses in *A. thaliana*

The discovery of the role of FIT in Cu homeostasis stems from our attempts to identify CITF1 protein-interacting partners. CITF1 clusters together with the Ib bHLH subfamily members, containing regulators of Fe homeostasis, bHLH38/39/100/101 (Gao and Dubos 2024). The expression of *bHLH38/39/100/101* is upregulated under Fe deficiency, but they do not respond transcriptionally to Cu deficiency. Encoded proteins heterodimerize with FIT to positively regulate Fe deficiency response and mediate Fe and Cu uptake under Fe deficiency (Yuan et al. 2008; Wang et al. 2013; Cai et al. 2021). Distinct from other bHLH Ib members, *CITF1* expression is upregulated under Cu deficiency but is downregulated under Fe deficiency, and CITF1 is a positive regulator of Cu deficiency response (Yan et al. 2017). We show here that CITF1 acts in Cu deficiency via interacting with FIT but not bHLH38, bHLH39, bHLH100, or bHLH101 (Fig. 1 and Figure S2).

Notably, CITF1-FIT complex abundance increases under Cu deficiency (Fig. 2d). This effect is most likely CITF1-dependent, as evidenced by the decreased stability of FIT in the *citf1-1* mutant genetic background (Fig. 3a, b). The proteasome-dependent pathway is likely involved in FIT depletion under Cu sufficiency since the proteasome inhibitor, M132, delays FIT degradation (Fig. 3c, d). Past findings also showed that proteasomal-dependent degradation pathways mediate FIT turnover (Lingam et al. 2011; Meiser et al. 2011; Sivitz et al. 2011; Rodríguez-Celma et al. 2019; Riaz and Guerinot 2021). Furthermore, CITF1 undergoes proteasomal-dependent degradation, and Cu deficiency stabilizes CITF1 protein abundance, thereby influencing FIT stability (Fig. 3c to e). Likewise, it has recently been suggested that bHLH Ib TFs (*eg,* bHLH38) stabilize FIT, preventing its degradation (Cui et al. 2018).

We also show that CITF1-FIT protein interactions are required for FIT binding to the *COPT2*, *FRO4*, and *FRO5* promoters, thereby upregulating their expression under Cu deficiency (Fig. 4). Thus, FIT emerges as a positive regulator of Cu deficiency response in *A. thaliana*. Consistent with this remark, the *fit-*2 mutant allele was more sensitive to Cu deficiency than the wild-type (Figs 8b, 10e, and Figure S10b).

Notably, while the expression of *COPT2* was downregulated in Cu-deficient *fit-2* relative to wild-type, it was still somewhat higher compared with control conditions (Fig. 4a). Likely, other FIT-independent regulators (eg, SPL7 and/or, perhaps, CITF2 (Yan et al. 2017 ; Schulten et al. 2022)) operate concurrently to ensure Cu uptake into cells (Fig. 4a).

### CITF1 disrupts protein–protein interactions of FIT with bHLH38/38/100/101 and emerges as a negative regulator of Fe homeostasis

The transcript abundance of *CITF1* decreases under Fe deficiency (Figure S3b and Yan et al. 2017 ), and *citf1-*1 mutants show elevated *FRO2* and *IRT1* expression under these conditions, whereas these genes are significantly downregulated in *CITF1* overexpression plants (Fig. 5). These findings, together with our observation that CITF1 disrupts FIT interactions with bHLH38/39/100/101 (Fig. 6 and Figure S6), support a role for CITF1 as a negative regulator of Fe homeostasis. We propose that reduced *CITF1* expression under Fe deficiency allows FIT to engage with its canonical partners and activate Fe-deficiency response (Fig. 12), whereas the CITF1-FIT complex functions primarily under Cu deficiency to ensure proper Cu/Fe cellular uptake.

In addition to CITF1, other transcription factors negatively regulate Fe homeostasis by interacting with FIT. For example, a bHLH family member MYC1 interacts with FIT and, similar to CITF1, suppresses FIT binding to bHLH38/39, thereby negatively regulating Fe homeostasis (Song et al. 2023). BRUTUS-LIKE E3 ligases (BTSL1 and BTSL2) interact with FIT, leading to its polyubiquitination and subsequent ubiquitin-mediated proteasomal degradation (Rodríguez-Celma et al. 2019). Mutants lacking BTSL1 and BTSL2 exhibit impaired FIT regulation and accumulate excessive Fe, underscoring the importance of this degradation pathway. Other regulators interact with FIT in repressing Fe deficiency response, including jasmonic acid-regulating bHLH IVa members (bHLH18/19/20/25) and an oxidative stress-induced transcription factor, Zinc Finger of *Arabidopsis thaliana* 12 (ZAT12) (Le et al. 2016; Cui et al. 2018). By contrast, recent studies have shown that the R2R3-MYB transcription factor MYB30 regulates FIT stability and acts as a positive regulator of the Fe-deficiency response (Zhao et al. 2025). These protein–protein interactions between FIT and other TFs may be required for dynamic, context-specific regulation of gene expression, ensuring that Fe and other mineral uptake are coordinated with overall plant nutrient status at the tissue- and cell-specific levels.

### *CITF1*—*FIT* genetic interactions reveal functional interplay and developmental roles

Genetic analyses support our conclusion based on molecular evidence that while CITF1 and FIT are positive regulators of Cu deficiency responses, CITF1 suppresses Fe deficiency responses. Under Cu deficiency, the *citf1-*2 *fit-*2 double mutant exhibits reduced root growth, compared with *citf1-*2 and *fit-*2 single mutants, a result that is consistent with partially additive functions in Cu homeostasis (Fig. 10e, Figure S10b). Consistent with the proposed role of the negative regulator of Fe deficiency, the *citf1-*1 and *citf1-*2 mutant alleles were more tolerant to Fe deficiency compared with the *fit-*2 mutant (Figs 8a, 10f, Figures S8, and S10b). Furthermore, the *citf1-*2 mutant partially suppresses *fit-*2 root growth defects under Fe deficiency, reinforcing the role of CITF1 as a negative regulator of Fe deficiency responses (Fig. 10f, Figure S10b).

Beyond nutrient stress, CITF1 and FIT are jointly essential for embryonic viability. The homozygous *citf1-*1 *fit-*2 double mutant is embryo-lethal, as inferred from ∼3:1 segregation of viable to aborted seeds (Table 1). This essential developmental role is supported by their co-expression during embryogenesis, and FIT has been confirmed as a phase-specific marker with high expression in the heart stages of embryonic development (Fig. 9c and Winter et al. 2007; Hofmann et al. 2019). Our past data and data presented in this manuscript also show that the seed set is partially compromised in the *citf1-*1 single mutant (Table 1 and Yan et al. 2017). Thus, the severity of the *citf1-*1 *fit-*2 phenotype likely reflects a simultaneous disruption of Cu and Fe delivery to developing embryos. In contrast, the *citf1-*2 *fit-*2 hypomorphic double mutant is viable but displays delayed inflorescence development and reduced stature (Fig. 10a to c), indicating that residual CITF1 activity supports minimal developmental progression. These results parallel findings in *opt3* mutant alleles, where knockdown *opt3-*2 and *opt3-*3 alleles of the Fe/Cu transporter, AtOPT3, supports embryo development, whereas a null *opt3-*1 allele is embryo lethal (Stacey et al. 2002; Stacey et al. 2008; Mendoza-Cózatl et al. 2014; Zhai et al. 2014; Chia et al. 2023). The role of Fe and Cu in embryo development is yet unknown.

### CITF1 functions as a central nutrient-responsive regulator of metal homeostasis

Our data identify CITF1 as an important regulator that orchestrates Cu-Fe cross-talk by modulating the activity of FIT, a master regulator of Fe acquisition (Fig. 12). FIT forms heterodimers with subgroup Ib bHLH transcription factors, including bHLH38, bHLH39, bHLH100, and bHLH101, to activate Fe uptake genes (Wu and Ling 2019). CITF1 interacts directly with FIT, establishing a CITF1-FIT module that contributes to Cu homeostasis (Figs. 1 to 5 and 12).

The choice of FIT binding partners appears to be nutritionally regulated (Fig. 12). Under Fe deficiency, *CITF1* expression is downregulated, allowing FIT to dimerize with bHLH38/39/100/101 to induce Fe uptake genes such as *IRT1* and *FRO2* (Wu and Ling 2019). FIT does not require CITF1 to regulate *COPT2* expression under Fe deficiency (Fig. 4f), and it has been shown that under Fe deficiency, FIT directly regulates *COPT2*, *FRO4* and *FRO5* through interactions with bHLH38/39 (Cai et al. 2021) and Fig. 12.

In contrast, under Cu deficiency, *CITF1* expression is strongly upregulated. CITF1 binding to FIT outcompetes or weakens FIT interaction with bHLH38/39/100/101 (Fig. 6 and Figure S6), forming a CITF1-FIT complex that specifically upregulates Cu uptake genes such as *COPT2*, *FRO4*, and *FRO5* (Fig. 12). This CITF1-mediated partner switch reprograms FIT activity to prioritize Cu uptake over Fe uptake under Cu limitation. Thus, CITF1 emerges as a nutrient-responsive transcriptional switch that redirects FIT function according to metal status.

Recent work shows that in addition to CITF1, IMA peptides play opposing roles in Fe and Cu homeostasis (Cai et al. 2024; Chia and Vatamaniuk 2024 ). *IMA*s are strongly induced by Fe-deficiency but repressed by Cu deficiency. Consistently, they act as positive regulators of Fe-deficiency signaling. Specifically, IMAs compete with IVc bHLH transcription factors for binding to BTS/BTSL proteins, thereby preventing their degradation and promoting the induction of *FRO2* and *IRT1* (Grillet et al. 2018; Hirayama et al. 2018; Li et al. 2021). In contrast, IMAs physically interact with CITF1 and inhibit its ability to bind the promoters of its target genes. Consistent with this, the *ima8x* mutant is more tolerant to Cu deficiency and shows elevated expression of the Cu uptake genes *COPT2*, *FRO4*, and *FRO5* (Cai et al. 2024). Although our study focuses on the CITF1-FIT module, the emerging connections between CITF1 and IMA peptides suggest that IMA-dependent signaling may contribute to the modulation of CITF1 activity under Fe limitation. Future work will be required to determine how these interactions shape transcriptional outputs and metal-nutrient crosstalk.

Interestingly, CITF1 is also required for Fe acquisition under Cu deficiency: the Cu-deficient *citf1-*1 mutant shows reduced shoot and root Fe content, even though canonical Fe uptake genes (*IRT1*, *FRO2*) are not induced (Fig. 11). This suggests that CITF1 contributes to a noncanonical Fe uptake pathway that operates specifically during Cu scarcity. Such increased Fe accumulation under Cu deficiency aligns with the well-established reciprocal relationship between Cu and Fe homeostasis in plants, in which Cu deficiency enhances Fe uptake and Fe deficiency enhances Cu uptake, as reviewed by (Chia et al. 2025; Wairich et al. 2025). Rather than being a secondary consequence of altered Cu uptake, this response is likely part of an adaptive Cu-homeostasis strategy described by the metal-switch model (Ravet and Pilon 2013; Chia et al. 2025). Under Cu deficiency, nonessential Cu-dependent enzymes are degraded and replaced by Fe-containing isoforms, for example, the substitution of Cu/Zn SODs with Fe-SODs. Elevated Fe uptake during Cu deficiency may therefore help supply Fe cofactors needed to maintain metabolic activity when Cu availability is limited.

Together, these findings position CITF1 as a coordinator of Cu/Fe cross-talk that functions through its interactions with FIT. By acting as both a positive regulator of Cu deficiency responses and a negative regulator of Fe acquisition, CITF1 integrates nutrient signals to fine-tune metal uptake strategies through selective modulation of FIT activity.

## Materials and methods

### Plant material and growth conditions

*Arabidopsis thaliana* (cv. Col-0) and *citf1-*1 and *citf1-*2 T-DNA insertion alleles (SALK_073160 and SAIL_711_B07, respectively) were obtained from the Arabidopsis Biological Resource Centre (Alonso et al. 2003) and described previously (Yan et al. 2017). The *fit-*2 mutant (SALK_126020) was a generous gift from the Mary-Lou Guerinot lab (Dartmouth College, USA) and was described in (Colangelo and Guerinot 2004). For soil-grown plants, seeds were stratified at 4°C in the dark for 2 d before being sown onto Lambert 111 all-purpose soil mix (lambertpeatmoss.com; product code: 64980 2512). NPK was used to irrigate plants weekly after seedlings were established. For growing plants hydroponically, a medium containing 1.25 mm KNO_3_, 0.625 mm KH_2_PO_4_, 0.5 mm MgSO_4_, 0.5 mm Ca(NO_3_) and the following micronutrients: 17.5 μm H_3_BO_3_, 3.5 μm MnCl_2_, 0.25 μm ZnSO_4_, 0.05 μm NaMoO_4_, 2.5 μm NaCl, and 2.5 nm CoCl_2_, 250 nm CuSO_4_ and 10 μm Fe(III) HBED (*N*, *N*′-Di (2-hydroxybenzyl) ethylenediamine-*N*, *N*′-diacetic acid) was considered as a standard solution unless indicated otherwise. Seeds were surface-sterilized as described in Jung et al. (2011) before sowing onto 0.7% (w/v) agar and aliquoted into 10-μL pipette tips. Pipette tips were cut 7 mm short before being placed on foam board floats. Seedling roots were immersed in the hydroponic solution after 1 wk of growth. The solution was replaced weekly, and 24 h before samples were collected for various analyses. All plants were grown in a growth chamber at 22 °C and 70% humidity under a 14-h light/10-h dark photoperiod with a photon flux density of 110 μmol m^−2^ s^−1^, provided by Philips F32T8/TL741 fluorescent light bulbs.

For immunoprecipitation and protein degradation assays, seeds were sterilized and stratified at 4 °C in the dark before being grown directly in a hydroponic solution with the indicated concentration of Fe(III) HBED and CuSO_4_. Approximately 50 to 60 seeds were mixed with 1.5 mL hydroponic solution and cultured in 24-well plates for 5 d with shaking at 100 rpm. The hydroponic solutions were changed every 2 d until plants were harvested for subsequent experiments.

### Plasmid construction and plant transformation

For yeast 2-hybrid assays, coding sequences of the full length of *CITF1* and *FIT*, and the C-terminal domain of *FIT* (*FIT-c* (Gratz et al. 2020)) were amplified from the cDNA prepared from *A. thaliana* roots. The amplicons were subcloned into the pGBKT7 DNA-BD or pGADT7-AD vectors to obtain the bait and prey constructs. For yeast 3-hybrid assays, *bHLH38*, *bHLH39*, *bHLH100,* and *bHLH101* coding sequences were subcloned into the multiple cloning site I of the pBridge vector (Clontech) as DNA-BD fusions. *CITF1* and MBP coding sequences were subcloned into the multiple cloning site II downstream of the MET25 promoter to provide methionine (Met) repression.

For plant transformation, the *CITF1* and *FIT* coding sequences were amplified using primers containing *attB* sites, and the resulting PCR products were introduced into the DONR222 entry vector (Invitrogen). For *CITF1* overexpression, the entry vector carrying *CITF1* CDS was cloned by recombination into the pYL436 destination vector to fuse the C-terminus of CITF1 recombinant protein with a tandem affinity tag (TAP) under the control of CaMV 35S promoter. TAP includes 9× MYC epitope, a His tag, and a 2× IgG BD (Rubio et al. 2005). Since we used the MYC epitope for immunodetection, we designated the resulting construct *35S_pro_:CITF1-MYC*. For *FIT* overexpression, the entry vector was recombined with the pEarleyGate 103 destination vector to fuse the C-terminus of the FIT recombinant protein with eGFP (Earley et al. 2006). The resulting constructs and empty binary vectors were introduced into the *Agrobacterium tumefaciens* strain GV3101 and transformed into the *citf1*-1 mutant via floral dipping (Clough and Bent 1998). The homozygous lines carrying *pYL436-CITF1* were selected based on segregation ratios on solid half-strength Murashige and Skoog (MS) medium containing gentamycin, and the transgenic lines carrying pEarleyGate103-FIT were selected on half-strength MS medium containing BASTA. To create transgenic lines co-expressing CITF1 and FIT fusions, the homozygous lines carrying pYL436-CITF1 were crossed with the homozygous lines carrying pEarleyGate103-FIT, followed by subsequent selections. The primer sequences used for cloning are listed in Table S2.

For transient co-expression of FIT and CITF1 in the protoplasts, full-length *FIT* cDNA without the stop codon was fused with the modified green fluorescent protein (EGFP) using the *pSAT6-N1-EGFP-Gate* vector (Jung et al. 2012). The resulting construct expresses *FIT:EGFP* fusion protein under the control of the cauliflower mosaic virus 35S promoter. For transient expression of CITF1 in the protoplasts, full-length *CITF1* cDNA without the stop codon was cloned into *pUC35S-mCherry* (NovoPro). The resulting construct expresses *CITF1:mCherry* fusion protein under the control of the cauliflower mosaic virus 35S promoter. The primer sequences used for cloning are listed in Supplemental Dataset S1.

### Yeast 2-hybrid assays

The Y2H library screening was carried out using Matchmaker Gold Yeast 2-Hybrid System (Takara PT4084-1), and the screen was performed following the manufacturer's instructions. Briefly, full-length *CITF1* CDS was expressed as a fusion protein with the Gal4 DNA-BD in yeast strain Y2HGold, and the cDNA library expressing fusions with Gal4-AD was provided in yeast strain Y187. The yeast strain carrying the CITF1 bait protein was mated with the library to create diploids, followed by the selection on solid SD/–Ade/–His/–Leu/–Trp dropout medium containing 40 *µ*g/mL X-α-Gal (Clontech 630463) and 200 ng/mL Aureobasidin A (Clontech 630466). The prey plasmids of confirmed positive interactions were then rescued and sequenced. For the subsequent confirmation of CITF1-FIT interaction, the *pGBKT7 DNA-BD-CITF1* and *pGADT7 AD-FIT* were co-transformed into the yeast strain Y2HGold. Interactions were selected on solid SD/–Ade/–His/–Leu/–Trp dropout medium or SD/–Leu/–Trp medium, supplemented with 40 *µ*g/mL X-α-Gal and 200 ng/mL Aureobasidin A. For all experiments, the prey vector expressing either SV40 large T antigen (SV40T) was used as a positive control, and the bait vector expressing either p53 or human lamin C was used as a negative control. To confirm CITF1-FIT interaction, yeast 2-hybrid experiments were performed using CITF1 or bHLH38/39/100/100 as baits and FIT as a prey. We also used FIT-c (lacking the N-terminal autoactivation domain (Lingam et al. 2011; Gratz et al. 2020)) as bait while CITF1 was used as prey.

### Yeast 3-hybrid assays

The Y3H assay was performed using FIT as prey in the pGADT7-AD vector. bHLH38/39/100/ were used as baits in the pBridge vector. cDNAs encoding CITF1 or MBP were cloned into the same pBridge vector under the control of *MET25* promoter to provide methionine repression. Different bait-prey vector combinations were co-transformed into the Y2HGold yeast strain. The transformed cells were selected on SD/–Leu/–Trp plates and then replated 3 times on SD/–Leu/–Trp/–Met plates to ensure methionine depletion in yeast cells. To induce *CITF1* or *MBP* expression, the yeast strains were grown in liquid SD/–Leu/–Trp/–Met overnight before being dropped on solid SD/–Ade/–His/–Leu/–Trp/–Met medium. To repress *CITF1* or *MBP* expression, the yeast strains were grown overnight in liquid SD/–Leu/–Trp medium before being spotted onto solid SD/–Ade/–His/–Leu/–Trp plates supplemented with 10 mM methionine.

### RT-qPCR analysis

The tissues were collected from plants at Zeitgeber time 7 (ZT, ZT 0 being defined as lights-on) and flash-frozen in liquid nitrogen before homogenization. Total RNA was isolated using TRIzol reagent (Invitrogen) according to the manufacturer's instructions. First-strand cDNA templates used for qPCR analysis were synthesized with AffinityScript QPCR cDNA Synthesis Kit (Agilent Technologies). To eliminate genomic DNA contamination before first-strand cDNA synthesis, 1 μg of total RNA was treated with DNase I (New England Biolabs). Real-time qPCR analysis was performed in a total volume of 15 μL containing 1 × iTaq Universal SYBR Green Supermix (Bio-Rad), 500 nm of each PCR primer, and 2 μL of 15 × diluted cDNA templates using the CFX96 real-time PCR system (Bio-Rad) as described (Chia et al. 2023). Data were normalized to the expression of *ACT2* (At3g18780), whose expression was stable under the investigated conditions and in the studied genotypes (Chia et al. 2023). The fold-difference (2−ΔΔCt) and the statistical parameters were calculated using the CFX Maestro Software (Bio-Rad). Sequences of the primer sets used in this study are listed in Table S2.

### Protein extraction

Protein extraction procedures for co-IP and protein turnover assays were modified after (Rubio et al. 2005; Meiser et al. 2011). The plant tissues were weighted and frozen in liquid nitrogen, followed by homogenization in an extraction buffer containing 50 mM Tris–HCl (pH 7.5), 150 mM NaCl, 10% glycerol, 0.1% Nonidet P-40, 1 mm DTT, 1 mm phenylmethylsulfonyl fluoride (PMSF), 1× protease inhibitor cocktail (Sigma-Aldrich, P9599), 20 *µ*m MG-132 (Sigma-Aldrich), and 2 mm PMSF. Twenty *µ*g of proteins were run on 10% SDS-PAGE gels after boiling in a buffer containing 1% SDS, 1% β-mercaptoethanol, and 50 mm Tris–HCl (pH 7.4). Protein extraction for FIT and IRT1 western blot analyses was performed as previously described (Sivitz et al. 2011). Briefly, 100 mg of fresh root samples were cryo-homogenized. Total proteins were extracted in 300 *µ*L protein extraction buffer containing 5% SDS, 5% β-mercaptoethanol, 50 mm Tris–HCl (pH 7.4), 1× protease inhibitor cocktail (Sigma-Aldrich, P9599), 20 *µ*m MG-132 (Sigma-Aldrich), and 2 mm PMSF (Millipore-Sigma). The protein extracts were centrifuged at 14,000 × *g* for 15 min to separate the total proteins from cell debris. The samples were then boiled at 95 °C for 10 min. before electrophoresis.

### Co-immunoprecipitation

Proteins (1 mg), extracted from transgenic lines as described above, were first incubated with 10 *µ*g of mouse-monoclonal anti-Myc antibody (Invitrogen MA1-21316) for 4 h at 4 °C with gentle rotation. The protein solutions were then incubated with 20 *µ*L Dynabeads Protein G (Invitrogen 10003D) for 1 h at 4 °C. The beads were washed 3 times with a buffer containing 50 mm Tris–HCl pH 7.5, 300 mm NaCl, 10% glycerol, and 0.1% Nonidet P-40. The proteins were eluted with 100 *µ*L 1× Laemmli SDS sample buffer before Western blot analysis.

### CHX and MG132 treatments

For copper or iron deficiency treatments, seedlings were grown hydroponically for 5 d in 24-well plates either in a standard hydroponic solution containing 250 nm CuSO_4_ and 10 *µ*m Fe-HBED (control) or in a solution without copper (−Cu) and with 1 *µ*m Fe-HBED (−Fe).CHX (355 *µ*m) or CHX and MG132 (355 *µ*m and 100 *µ*m, respectively) were added to 1.5 mL aliquot of hydroponic solutions containing 50 to 60 seedlings per condition and incubated for the indicated time. Plant samples were collected and frozen in liquid nitrogen for protein extraction.

### Western blot analysis

Proteins were separated on a 10% sodium dodecyl sulfate-polyacrylamide gel electrophoresis (SDS-PAGE) and were transferred onto a nitrocellulose membrane (BIO-RAD) by electroblotting. The Western blotting procedures were described previously with slight modifications (Kim et al. 2010). The membranes were first blocked in EveryBlot Blocking Buffer (Bio-Rad) and then probed overnight at 4 °C with the primary antibodies. The membranes were then washed 3 times with the PBST buffer (137 mm NaCl, 2.7 mm KCl, 10 mm Na_2_HPO_4_, 1.8 mm KH_2_PO_4_, and 0.1% [w/v] Tween-20) and incubated with HRP-conjugated secondary antibodies for 1 h, followed by additional washes. The immunoreactive bands were visualized with Clarity Max ECL blotting substrates (BIO-RAD). The antibodies used to detect the accumulation of proteins in plant tissues were rabbit polyclonal anti-IRT1 antibody (Agrisera AS11-1780), rabbit polyclonal anti-FIT antibody (PhytoAB PHY0940A), mouse monoclonal anti-actin antibody (Sigma-Adrich A0480), mouse monoclonal anti-GFP antibody (Clontech JL-8), mouse-monoclonal anti-MYC antibody (Invitrogen MA1-21316), HRP-conjugated goat anti-mouse IgG and anti-rabbit IgG antibodies (Rockland Immunochemicals). For the immunodetection of the MYC epitope in co-IP experiments, a mouse monoclonal anti-MYC antibody conjugated with HRP (Cell Signaling Technology 2,040) was used. In all cases, the dilution factors suggested by the manufacturers were used for each antibody.

### Chromatin-immunoprecipitation

The procedures of chromatin-immunoprecipitation (ChIP) were adapted from Yamaguchi *et al.* (2014) with slight modifications. In brief, 500 mg of 5-d-old seedlings were fixed with 50 mL of 1% formaldehyde solution in phosphate saline buffer, pH 7.0, using vacuum infiltration for 10 minutes, followed by 5 minutes of incubation. The cross-linking reaction was stopped by adding glycine to a final concentration of 0.125 M, followed by vacuum infiltration for 5 min. The samples were then cryo-homogenized in the extraction buffer A (10 mm Tris–HCl pH 8.0, 400 mm sucrose, 10 mm MgCl_2_, 1 mm PMSF, 1× protease inhibitor cocktail (Sigma-Aldrich, P9599), 20 *µ*m MG-132 (Sigma-Aldrich), and 2 mm PMSF (Millipore-Sigma)) followed by centrifugation at 3,000 × *g*. The pellets were resuspended in 1 mL extraction buffer B (10 mm Tris–HCl pH 8.0, 250 mm sucrose, 10 mm MgCl_2_ and 1% Triton X-100) followed by centrifugation at 10,000*×g*. The pellets were then resuspended again in the same buffer containing 0.5% Triton X-100 and then layered carefully on top of 500 *µ*L extraction buffer C (10 mm Tris–HCl, pH 8.0, 1.7 M sucrose, 2 mm MgCl_2_ and 0.15% Triton X-100) followed by centrifugation at 16,000 × *g* to isolate nuclei. The resulting pellet from the centrifugation, containing nuclei, was used for chromatin extraction in a lysis buffer containing 50 mm Tris–HCl, pH 8.0, 10 mm EDTA, 1% SDS, 2 mm PMSF, 1× protease inhibitor cocktail, and 20 *µ*m MG-132. Extracted chromatin was sonicated using a probe sonicator to achieve an average chromatin fragment size of 200 to 500 bp. The chromatin was then separated by centrifugation at 16,000 × *g* to remove cellular debris. Supernatant from the centrifugation was 10-fold diluted with the ChIP dilution buffer containing 16.7 mm Tris–HCl, 1.2 mm EDTA, 167 mm NaCl, and 1.1% Triton X-100, 2 mm PMSF, 1× protease inhibitor cocktail and 20 *µ*m MG-132. The diluted chromatin solution was precleared by incubating at 4 °C for 1 h with 20 *µ*L Dynabeads Protein G (Invitrogen 10003D). An aliquot, corresponding to 0.5% of the starting chromatin volume, was then taken to be used as the input DNA control before adding 10 *µ*g anti-GFP antibody (Clontech JL-8). Next, the chromatin-antibody solution was incubated at 4 °C overnight on a rotary shaker with gentle agitation. The solution was then incubated at 4 °C for 2 h with 20 *µ*L Dynabeads Protein G (Invitrogen 10003D). The beads were then washed sequentially as follows: 1 wash step with low salt buffer (20 mm Tris–HCl pH 8.0, 2 mm EDTA 150 mm NaCl, 1% Triton X-100, 0.1% SDS), 1 wash step with high salt buffer (20 mm Tris–HCl pH 8.0, 2 mm EDTA, 500 mm NaCl, 1% Triton X-100, 0.1% SDS), 1 wash step with LiCl buffer (20 mm Tris–HCl pH 8.0, 1 mm EDTA, 250 mm LiCl, 1% NP-40, 0.1% sodium deoxycholate) and 2 wash steps with TE buffer (10 mm Tris–HCl pH 8.0 and 1 mm EDTA). The protein-DNA complexes were eluted in a buffer containing 50 mm Tris–HCl pH 8.0, 10 mm DETA, 1% SDS and 300 mm NaCl at 65 °C for 30 min. Reverse cross-linking was performed by adding NaCl to the eluates and the input DNA aliquots to a final concentration of 0.2 M before further incubating at 65 °C for 12 h. The RNA and protein in the samples were then digested by RNase A at 37 °C for 1.5 h and proteinase K at 45 °C for 2 h, respectively. The DNA was isolated using MinElute PCR purification kit (QUIAGEN). ChIP samples were analyzed by qPCR as described above.

### ICP-MS analysis

Root and shoot tissues were collected from 4-wk-old plants grown hydroponically with the indicated concentration of Fe and Cu. Roots and shoots were collected separately. Shoots were rinsed with deionized water 3 times before collecting samples. Iron and other elements from root cell walls were desorbed by washing roots in 10 mm EDTA for 10 min and with a solution containing 0.3 mm bathophenanthroline disulfonate and 5.7 mm sodium dithionite for 3 min, followed by 3 consecutive washes in deionized water. All samples were dried in an 80 °C oven for 3 d before measuring their weight. Elemental analysis was performed using inductively coupled plasma mass spectroscopy (ICP-MS; Agilent 7,500).

### Subcellular localization and fluorescent microscopy

The protoplasts were isolated from wild-type *A. thaliana* leaf mesophyll cells and co-transfected with equimolar amounts of *pSAT6-N1-FIT:EGFP* and *pUC35S-CITF1:mCherry* using previously established procedures (Zhai et al. 2009a,2009b; Jung et al. 2011). To visualize nuclei, protoplasts were incubated for 15 min with 50 *µ*g/mL 4′,6-diamidino-2-phenylindole dihydrochloride (DAPI). DAPI excess was eliminated by washing protoplasts in W5 medium as described (Yan et al. 2017). EGFP, mCherry, DAPI fluorescence, and chlorophyll autofluorescence were visualized using FITC, Texas Red, DAPI, and rhodamine filter sets, respectively, on an Axio Imager M2 microscope (Zeiss). Images were processed using ImageJ (Schneider et al. 2012).

### Plant growth on plates

Seeds were surface-sterilized, stratified at 4 °C for 2 d in darkness, and sown on half-strength Murashige and Skoog (MS) plates (Murashige and Skoog 1962). Plants were grown vertically for 10 d before the plates were photographed. A half-strength MS medium containing 50 *µ*m FeSO_4_•EDTA and 5 *µ*m CuSO_4_ was used as a control condition. For Fe-deficient treatment, a medium without FeSO_4_•EDTA was used. To achieve Cu-deficient conditions, 100  μM of Cu(I)-specific chelator, BCS, was added to the medium without CuSO_4_. The growth media were supplemented with 1% (w/v) glucose and 0.7% agar and adjusted to pH 5.8. The root length of the seedlings was analyzed using ImageJ (Schneider et al. 2012).

### Statistical analysis

Statistical analyses of experimental data were performed using the ANOVA single-factor analysis and Tukey HSD analysis or Student's *t*-test for planned comparisons with specific conditions, using JMP® Pro 15 (SAS Institute Inc., Cary, NC, 1989 to 2007). Results of the statistical analyses are provided in a Supplemental Dataset S2.

### Accession Numbers

Sequence data of the genes from this article can be found in the Arabidopsis Genome Initiative or GenBank/EMBL databases under the following accession numbers: *CITF1* (AT1G71200), *FIT* (AT2G28160), *FRO4* (AT5G23980), *FRO5* (AT5G23990), *COPT2* (AT3G46900), *IRT1* (AT4G19690), *FRO2* (AT1G01580), *bHLH38* (AT3G56970), *bHLH39* (AT3G56980), *bHLH100* (AT2G41240), *bHLH101* (AT5G04150).

## Supplementary Material

koag114_Supplementary_Data

## Data Availability

The data supporting the ﬁndings of this study are openly available. All data, including accession numbers, are provided in the article and the supporting information.

## Dive Curated Terms

The following phenotypic, genotypic, and functional terms are of significance to the work described in this paper:

SPL7 Gramene: At5g18830
SPL7 Araport: At5g18830

MYB30 Gramene: AT3G28910
MYB30 Araport: AT3G28910

bHLH100 Gramene: AT2G41240
bHLH100 Araport: AT2G41240

bHLH101 Gramene: AT5G04150
bHLH101 Araport: AT5G04150

BTSL2 Gramene: AT1G18910
BTSL2 Araport: AT1G18910

CITF1 Gramene: At1g71200
CITF1 Araport: At1g71200

CITF2 Gramene: At4g28790
CITF2 Araport: At4g28790

OPT3 Gramene: AT4G16370
OPT3 Araport: AT4G16370
