# Supplementary material for: CITF1 interacts with FIT and regulates copper–iron crosstalk in Arabidopsis
Source: Plant Cell. 2026 Apr 16;38(5):koag114. doi: 10.1093/plcell/koag114 (PMC13143221; doi:10.1093/plcell/koag114)
Supplement: koag114_Supplementary_Data [file koag114_supplementary_data.zip › Supplemental Dataset 2.pdf]

Fit Group

Oneway Analysis of MYC/ACT normalized by ctr By Treatment

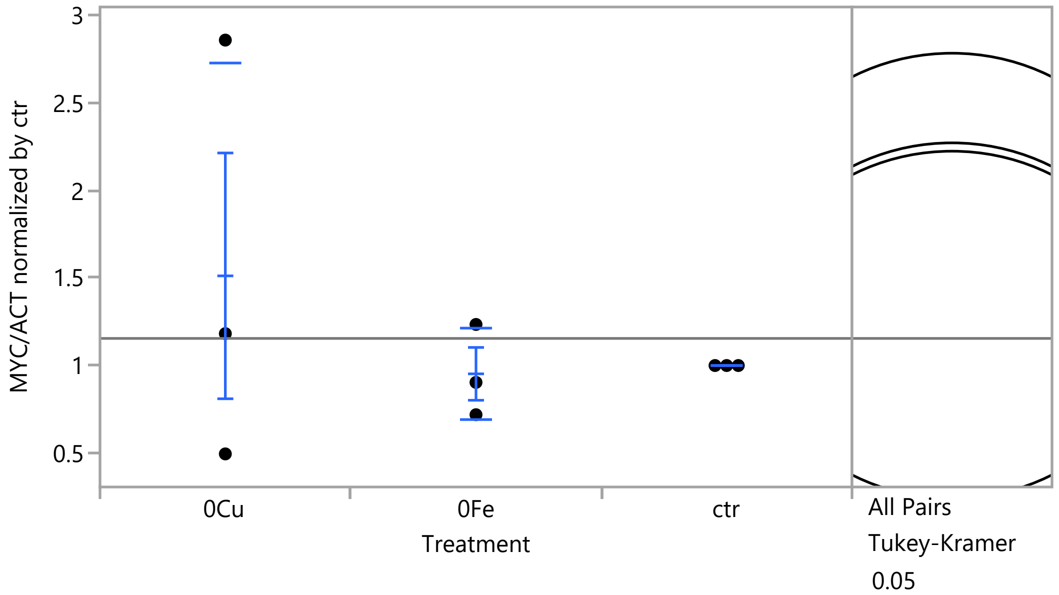

Means and Std Deviations

Level	Number	Mean	Std Dev	Std Err Mean	Lower 95%	Upper 95%
0Cu	3	1.5114435	1.214354	0.7011076	-1.505179	4.5280659
0Fe	3	0.9525162	0.260887	0.1506232	0.3044371	1.6005954
ctr	3	1	0	0	1	1

Fit Group

Oneway Analysis of MYC/ACT normalized by ctr By Treatment

Means Comparisons

Comparisons for all pairs using Tukey-Kramer HSD

Confidence Quantile

q*	Alpha
3.06815	0.05

HSD Threshold Matrix

Abs(Dif)-HSD

	0Cu	ctr	0Fe
0Cu	-1.7964	-1.2850	-1.2375
ctr	-1.2850	-1.7964	-1.7490
0Fe	-1.2375	-1.7490	-1.7964

Positive values show pairs of means that are significantly different.

Connecting Letters Report

Level		Mean	Std Error
0Cu	A	1.5114	0.41402
ctr	A	1.0000	0.41402
0Fe	A	0.9525	0.41402

Levels not connected by same letter are significantly different.

Ordered Differences Report

Level	- Level	Difference	Std Err Dif	Lower CL	Upper CL	p-Value
0Cu	0Fe	0.5589273	0.5855136	-1.23752	2.355370	0.6290
0Cu	ctr	0.5114435	0.5855136	-1.28500	2.307886	0.6750
ctr	0Fe	0.0474838	0.5855136	-1.74896	1.843927	0.9964

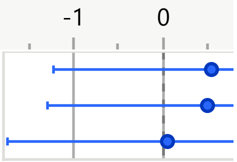

Oneway Analysis of GFP/ACT normalized by ctr By Treatment

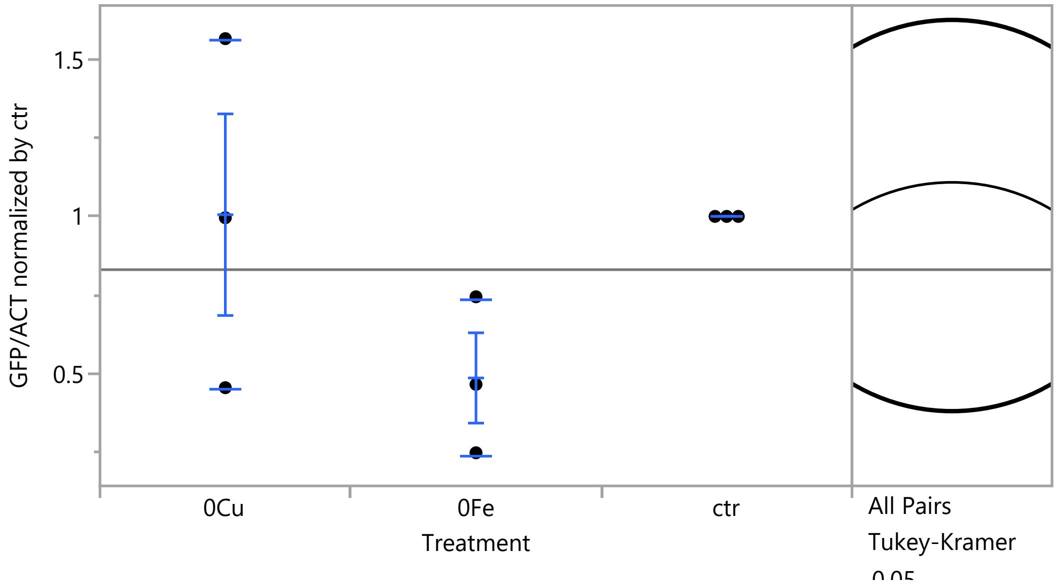

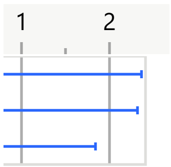
1
2

Fit Group

Oneway Analysis of GFP/ACT normalized by ctr By Treatment

Means and Std Deviations

Level	Number	Mean	Std Dev	Std Err Mean	Lower 95%	Upper 95%
0Cu	3	1.0054181	0.5539402	0.3198175	-0.370646	2.3814818
0Fe	3	0.4872154	0.2481888	0.1432919	-0.12932	1.1037506
ctr	3	1	0	0	1	1

Means Comparisons

Comparisons for all pairs using Tukey-Kramer HSD

Confidence Quantile

q*	Alpha
3.06815	0.05

HSD Threshold Matrix

Abs(Dif)-HSD

	0Cu	ctr	0Fe
0Cu	-0.87793	-0.87251	-0.35972
ctr	-0.87251	-0.87793	-0.36514
0Fe	-0.35972	-0.36514	-0.87793

Positive values show pairs of means that are significantly different.

Connecting Letters Report

Level		Mean	Std Error
0Cu	A	1.0054	0.20233
ctr	A	1.0000	0.20233
0Fe	A	0.4872	0.20233

Levels not connected by same letter are significantly different.

Ordered Differences Report

Level	- Level	Difference	Std Err Dif	Lower CL	Upper CL	p-Value
0Cu	0Fe	0.5182027	0.2861419	-0.359724	1.396129	0.2444
ctr	0Fe	0.5127846	0.2861419	-0.365142	1.390711	0.2502
0Cu	ctr	0.0054181	0.2861419	-0.872508	0.883344	0.9998

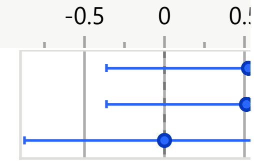

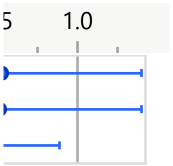
1.0

Oneway Analysis of GFP/ACT normalized by ctr By Treatment

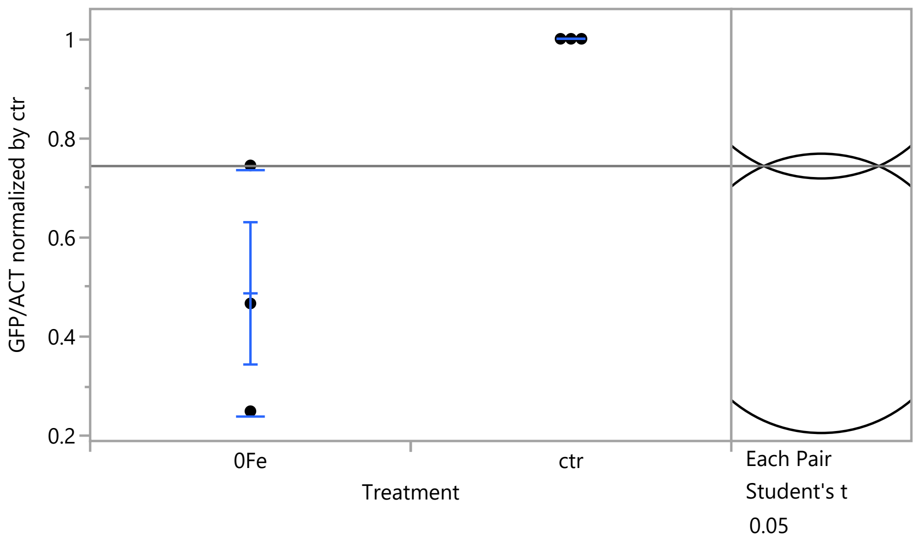

Means and Std Deviations

Level	Number	Mean	Std Dev	Std Err Mean	Lower 95%	Upper 95%
0Fe	3	0.4872154	0.2481888	0.1432919	-0.12932	1.1037506
ctr	3	1	0	0	1	1

Means Comparisons

Comparisons for each pair using Student's t

Confidence Quantile

t	Alpha
2.77645	0.05

LSD Threshold Matrix

Abs(Dif)-LSD

	ctr	0Fe
ctr	-0.39784	0.11494
0Fe	0.11494	-0.39784

Positive values show pairs of means that are significantly different.

Connecting Letters Report

Level			Mean	Std Error
ctr	A		1.0000	0.10132
0Fe		B	0.4872	0.10132

Levels not connected by same letter are significantly different.

Ordered Differences Report

Level	- Level	Difference	Std Err Dif	Lower CL	Upper CL	p-Value
ctr	0Fe	0.5127846	0.1432919	0.1149426	0.9106266	0.0232*

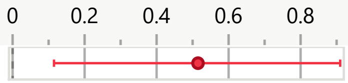

Fit Group

Oneway Analysis of MYC (co-IP/input) By Column 1

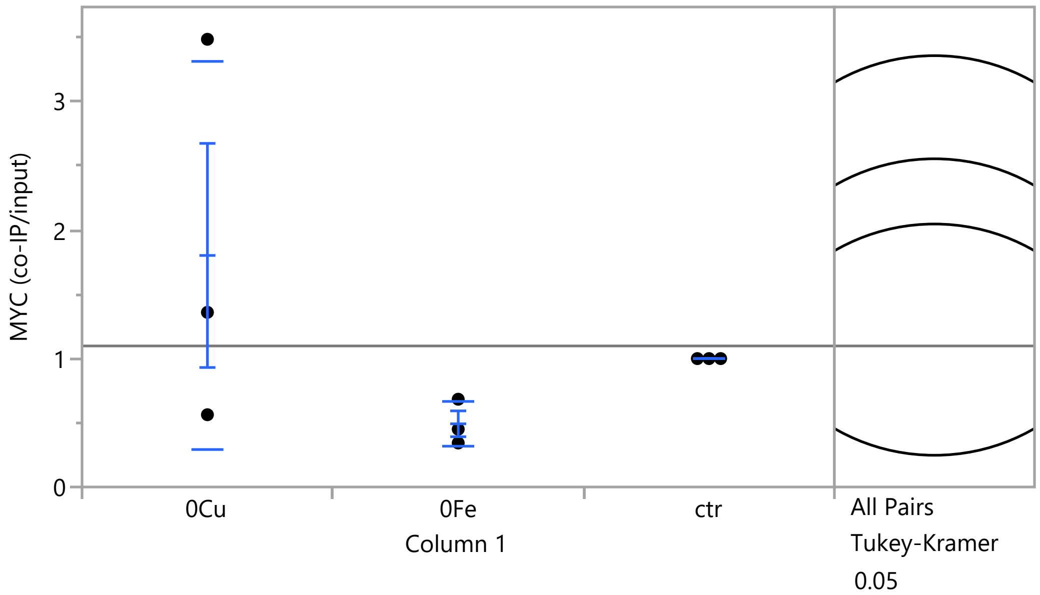

Means and Std Deviations

Level	Number	Mean	Std Dev	Std Err Mean	Lower 95%	Upper 95%
0Cu	3	1.803194	1.5113492	0.8725779	-1.951206	5.5575936
0Fe	3	0.4920736	0.1742487	0.1006026	0.0592157	0.9249314
ctr	3	1	0	0	1	1

Fit Group

Oneway Analysis of MYC (co-IP/input) By Column 1

Means Comparisons

Comparisons for all pairs using Tukey-Kramer HSD

Confidence Quantile

q*	Alpha
3.06815	0.05

HSD Threshold Matrix

Abs(Dif)-HSD

	0Cu	ctr	0Fe
0Cu	-2.2004	-1.3972	-0.8893
ctr	-1.3972	-2.2004	-1.6925
0Fe	-0.8893	-1.6925	-2.2004

Positive values show pairs of means that are significantly different.

Connecting Letters Report

Level		Mean	Std Error
0Cu	A	1.8032	0.50712
ctr	A	1.0000	0.50712
0Fe	A	0.4921	0.50712

Levels not connected by same letter are significantly different.

Ordered Differences Report

Level	- Level	Difference	Std Err Dif	Lower CL	Upper CL	p-Value
0Cu	0Fe	1.311120	0.7171764	-0.88928	3.511525	0.2393
0Cu	ctr	0.803194	0.7171764	-1.39721	3.003598	0.5376
ctr	0Fe	0.507926	0.7171764	-1.69248	2.708331	0.7678

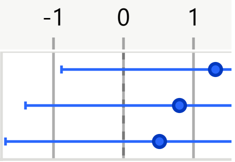

Missing Rows 23

Oneway Analysis of GFP(co-IP/input) By Column 1

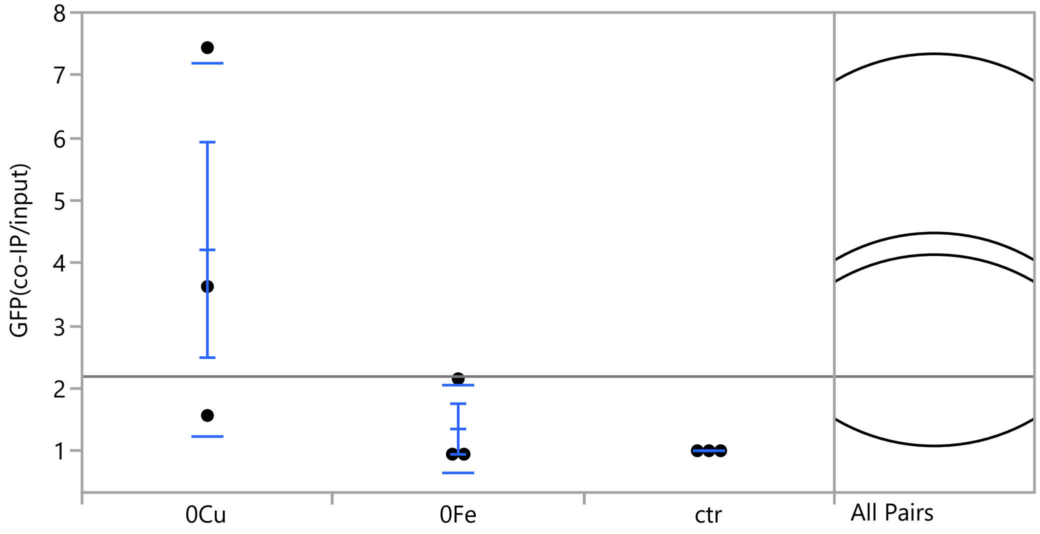

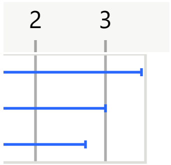
2
3

Fit Group

Oneway Analysis of GFP(co-IP/input) By Column 1

0.05

Means and Std Deviations

Level	Number	Mean	Std Dev	Std Err Mean	Lower 95%	Upper 95%
0Cu	3	4.2082597	2.9810078	1.7210857	-3.196974	11.613494
0Fe	3	1.3465695	0.7010373	0.4047441	-0.394904	3.0880427
ctr	3	1	0	0	1	1

Means Comparisons

Comparisons for all pairs using Tukey-Kramer HSD

Confidence Quantile

q*	Alpha
3.06815	0.05

HSD Threshold Matrix

Abs(Dif)-HSD

	0Cu	0Fe	ctr
0Cu	-4.4292	-1.5675	-1.2209
0Fe	-1.5675	-4.4292	-4.0826
ctr	-1.2209	-4.0826	-4.4292

Positive values show pairs of means that are significantly different.

Connecting Letters Report

Level		Mean	Std Error
0Cu	A	4.2083	1.0208
0Fe	A	1.3466	1.0208
ctr	A	1.0000	1.0208

Levels not connected by same letter are significantly different.

Ordered Differences Report

Level	- Level	Difference	Std Err Dif	Lower CL	Upper CL	p-Value
0Cu	ctr	3.208260	1.443596	-1.22091	7.637427	0.1455
0Cu	0Fe	2.861690	1.443596	-1.56748	7.290858	0.1973
0Fe	ctr	0.346569	1.443596	-4.08260	4.775737	0.9689

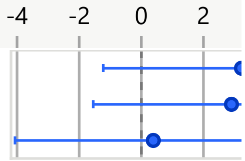

Missing Rows 23

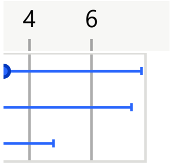
4
6

Oneway Analysis of MYC (co-IP/input) By Column 1

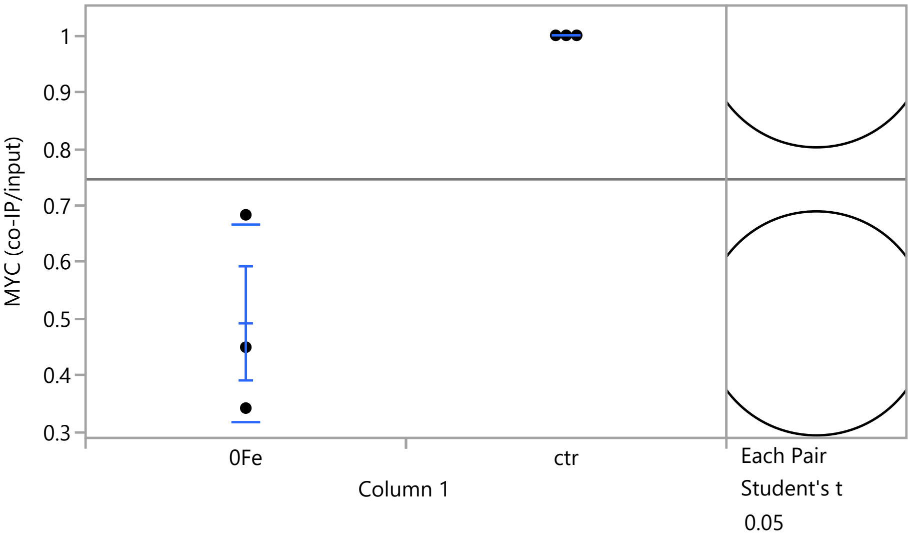

Means and Std Deviations

Level	Number	Mean	Std Dev	Std Err Mean	Lower 95%	Upper 95%
0Fe	3	0.4920736	0.1742487	0.1006026	0.0592157	0.9249314
ctr	3	1	0	0	1	1

Means Comparisons

Comparisons for each pair using Student's t

Confidence Quantile

t	Alpha
2.77645	0.05

LSD Threshold Matrix

Abs(Dif)-LSD

	ctr	0Fe
ctr	-0.27932	0.22861
0Fe	0.22861	-0.27932

Positive values show pairs of means that are significantly different.

Connecting Letters Report

Level			Mean	Std Error
ctr	A		1.0000	0.07114
0Fe		B	0.4921	0.07114

Levels not connected by same letter are significantly different.

Ordered Differences Report

Level	- Level	Difference	Std Err Dif	Lower CL	Upper CL	p-Value
ctr	0Fe	0.5079264	0.1006026	0.2286090	0.7872439	0.0072*

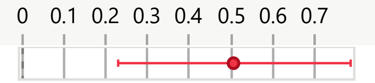

Oneway Analysis of GFP/MYC By Column 1

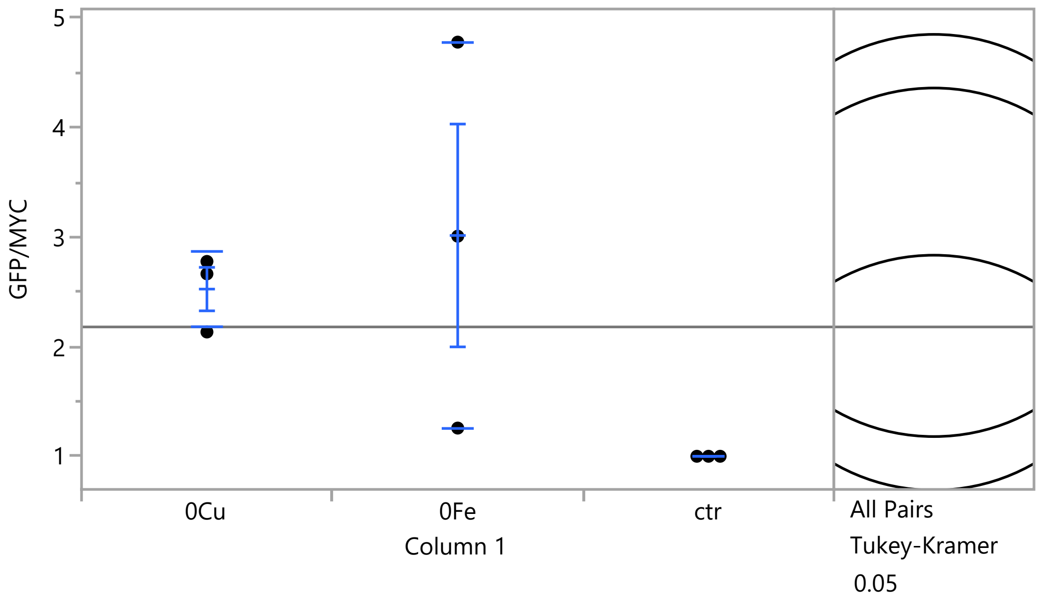

Means and Std Deviations

Level	Number	Mean	Std Dev	Std Err Mean	Lower 95%	Upper 95%
0Cu	3	2.5247548	0.3435561	0.1983522	1.6713143	3.3781954
0Fe	3	3.0137883	1.7599491	1.0161071	-1.358168	7.3857443
ctr	3	1	0	0	1	1

Oneway Analysis of GFP/MYC By Column 1

Means Comparisons

Comparisons for all pairs using Tukey-Kramer HSD

Confidence Quantile

q*	Alpha
3.06815	0.05

HSD Threshold Matrix

Abs(Dif)-HSD

	0Fe	0Cu	ctr
0Fe	-2.5935	-2.1045	-0.5797
0Cu	-2.1045	-2.5935	-1.0688
ctr	-0.5797	-1.0688	-2.5935

Positive values show pairs of means that are significantly different.

Connecting Letters Report

Level		Mean	Std Error
0Fe	A	3.0138	0.59772
0Cu	A	2.5248	0.59772
ctr	A	1.0000	0.59772

Levels not connected by same letter are significantly different.

Ordered Differences Report

Level	- Level	Difference	Std Err Dif	Lower CL	Upper CL	p-Value
0Fe	ctr	2.013788	0.8453075	-0.57974	4.607318	0.1186
0Cu	ctr	1.524755	0.8453075	-1.06877	4.118285	0.2466
0Fe	0Cu	0.489034	0.8453075	-2.10450	3.082563	0.8362

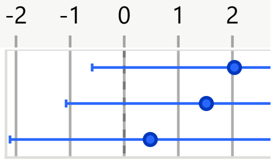

Missing Rows 23

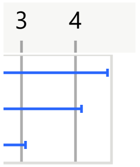
3
4

Oneway Analysis of GFP/MYC By Column 2

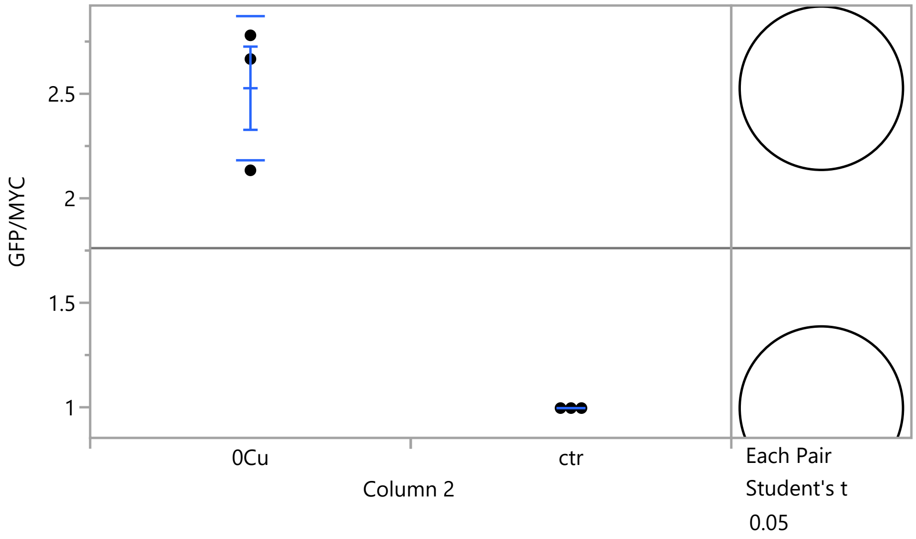

Means and Std Deviations

Level	Number	Mean	Std Dev	Std Err Mean	Lower 95%	Upper 95%
0Cu	3	2.5247548	0.3435561	0.1983522	1.6713143	3.3781954
ctr	3	1	0	0	1	1

Means Comparisons

Comparisons for each pair using Student's t

Confidence Quantile

t	Alpha
2.77645	0.05

LSD Threshold Matrix

Abs(Dif)-LSD

	0Cu	ctr
0Cu	-0.55071	0.97404
ctr	0.97404	-0.55071

Positive values show pairs of means that are significantly different.

Connecting Letters Report

Level			Mean	Std Error
0Cu	A		2.5248	0.14026
ctr		B	1.0000	0.14026

Levels not connected by same letter are significantly different.

Ordered Differences Report

Level	- Level	Difference	Std Err Dif	Lower CL	Upper CL	p-Value
0Cu	ctr	1.524755	0.1983522	0.9740409	2.075469	0.0015*

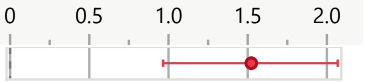

Oneway Analysis of Intensity By Treatment

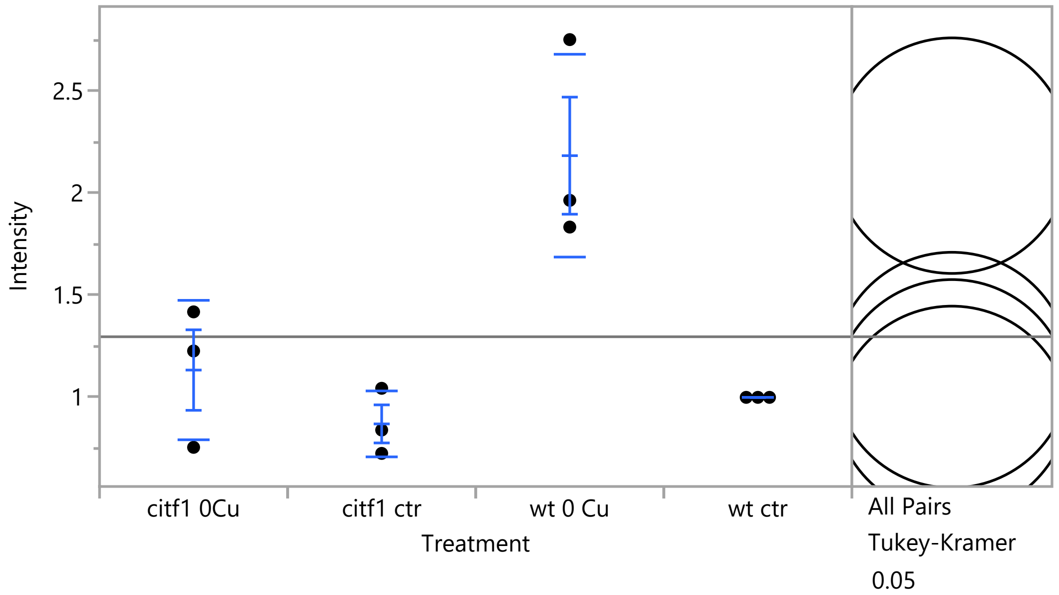

Means and Std Deviations

Level	Number	Mean	Std Dev	Std Err Mean	Lower 95%	Upper 95%
citf1 0Cu	3	1.1337597	0.3417314	0.1972987	0.2848519	1.9826676
citf1 ctr	3	0.8698213	0.1616071	0.0933039	0.468367	1.2712756
wt 0 Cu	3	2.1846137	0.4970044	0.2869457	0.9499862	3.4192411
wt ctr	3	1	0	0	1	1

Oneway Analysis of Intensity By Treatment

Means Comparisons

Comparisons for all pairs using Tukey-Kramer HSD

Confidence Quantile

q*	Alpha
3.20234	0.05

HSD Threshold Matrix

Abs(Dif)-HSD

	wt 0 Cu	citf1 0Cu	wt ctr	citf1 ctr
wt 0 Cu	-0.81635	0.23451	0.36827	0.49845
citf1 0Cu	0.23451	-0.81635	-0.68259	-0.55241
wt ctr	0.36827	-0.68259	-0.81635	-0.68617
citf1 ctr	0.49845	-0.55241	-0.68617	-0.81635

Positive values show pairs of means that are significantly different.

Connecting Letters Report

Level			Mean	Std Error
wt 0 Cu	A		2.1846	0.18026
citf1 0Cu		B	1.1338	0.18026
wt ctr		B	1.0000	0.18026
citf1 ctr		B	0.8698	0.18026

Levels not connected by same letter are significantly different.

Ordered Differences Report

Level	- Level	Difference	Std Err Dif	Lower CL	Upper CL	p-Value
wt 0 Cu	citf1 ctr	1.314792	0.2549218	0.498445	2.131140	0.0038*
wt 0 Cu	wt ctr	1.184614	0.2549218	0.368266	2.000961	0.0072*
wt 0 Cu	citf1 0Cu	1.050854	0.2549218	0.234507	1.867201	0.0141*
citf1 0Cu	citf1 ctr	0.263938	0.2549218	-0.552409	1.080286	0.7349
citf1 0Cu	wt ctr	0.133760	0.2549218	-0.682587	0.950107	0.9507
wt ctr	citf1 ctr	0.130179	0.2549218	-0.686168	0.946526	0.9542

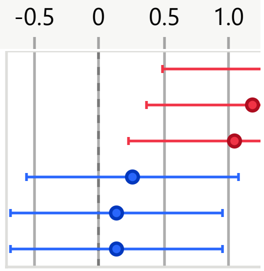

Missing Rows 5

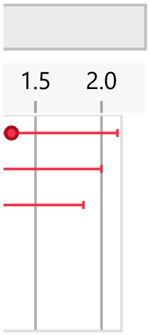
1.5
2.0

Oneway Analysis of GFP By Treatment

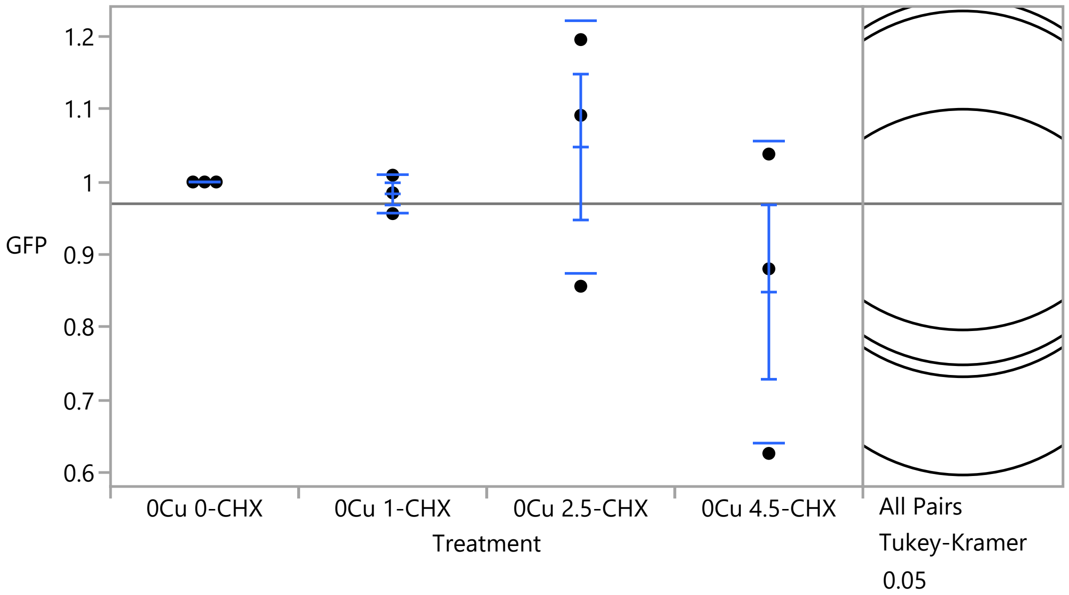

Means and Std Deviations

Level	Number	Mean	Std Dev	Std Err Mean	Lower 95%	Upper 95%
0Cu 0-CHX	3	1	0	0	1	1
0Cu 1-CHX	3	0.9835937	0.0265186	0.0153105	0.917718	1.0494695
0Cu 2.5-CHX	3	1.0481306	0.1741426	0.1005413	0.6155364	1.4807248
0Cu 4.5-CHX	3	0.8482107	0.2081563	0.1201791	0.3311219	1.3652995

Oneway Analysis of GFP By Treatment

Means Comparisons

Comparisons for all pairs using Tukey-Kramer HSD

Confidence Quantile

q*	Alpha
3.20234	0.05

HSD Threshold Matrix

Abs(Dif)-HSD

	0Cu 2.5-CHX	0Cu 0-CHX	0Cu 1-CHX	0Cu 4.5-CHX
0Cu 2.5-CHX	-0.35650	-0.30837	-0.29196	-0.15658
0Cu 0-CHX	-0.30837	-0.35650	-0.34009	-0.20471
0Cu 1-CHX	-0.29196	-0.34009	-0.35650	-0.22111
0Cu 4.5-CHX	-0.15658	-0.20471	-0.22111	-0.35650

Positive values show pairs of means that are significantly different.

Connecting Letters Report

Level		Mean	Std Error
0Cu 2.5-CHX	A	1.0481	0.07872
0Cu 0-CHX	A	1.0000	0.07872
0Cu 1-CHX	A	0.9836	0.07872
0Cu 4.5-CHX	A	0.8482	0.07872

Levels not connected by same letter are significantly different.

Ordered Differences Report

Level	- Level	Difference	Std Err Dif	Lower CL	Upper CL	p-Value
0Cu 2.5-CHX	0Cu 4.5-CHX	0.1999199	0.1113238	-0.156577	0.5564170	0.3414
0Cu 0-CHX	0Cu 4.5-CHX	0.1517893	0.1113238	-0.204708	0.5082863	0.5527
0Cu 1-CHX	0Cu 4.5-CHX	0.1353830	0.1113238	-0.221114	0.4918801	0.6346
0Cu 2.5-CHX	0Cu 1-CHX	0.0645369	0.1113238	-0.291960	0.4210339	0.9354
0Cu 2.5-CHX	0Cu 0-CHX	0.0481306	0.1113238	-0.308366	0.4046277	0.9712
0Cu 0-CHX	0Cu 1-CHX	0.0164063	0.1113238	-0.340091	0.3729033	0.9988

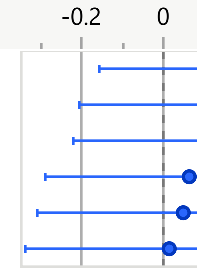

Missing Rows 8

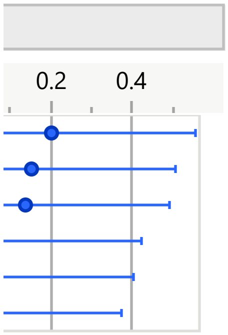
0.2
0.4

Oneway Analysis of GFP By Treatment

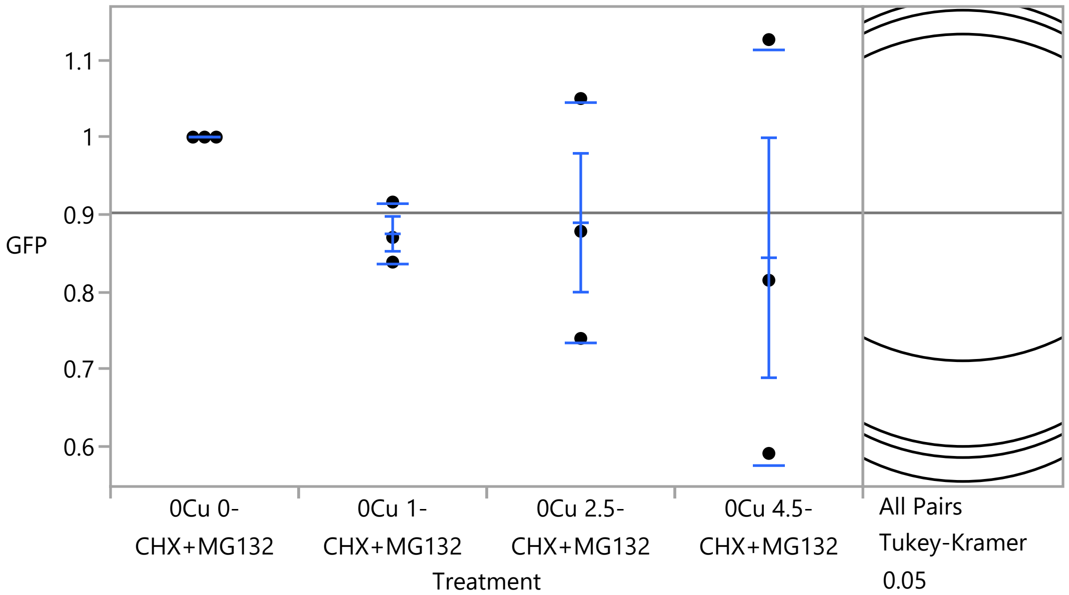

Means and Std Deviations

Level	Number	Mean	Std Dev	Std Err Mean	Lower 95%	Upper 95%
0Cu 0-CHX+MG132	3	1	0	0	1	1
0Cu 1-CHX+MG132	3	0.8747889	0.0390648	0.022554	0.7777466	0.9718311
0Cu 2.5-CHX+MG132	3	0.8891647	0.1555277	0.089794	0.5028125	1.2755169
0Cu 4.5-CHX+MG132	3	0.8438697	0.2689392	0.1552721	0.1757876	1.5119517

Oneway Analysis of GFP By Treatment

Means Comparisons

Comparisons for all pairs using Tukey-Kramer HSD

Confidence Quantile

q*	Alpha
3.20234	0.05

HSD Threshold Matrix

Abs(Dif)-HSD

	0Cu 0-CHX+MG132	0Cu 2.5-CHX+MG132	0Cu 1-CHX+MG132	0Cu 4.5-CHX+MG132
0Cu 0-CHX+MG132	-0.40936	-0.29852	-0.28414	-0.25323
0Cu 2.5-CHX+MG132	-0.29852	-0.40936	-0.39498	-0.36406
0Cu 1-CHX+MG132	-0.28414	-0.39498	-0.40936	-0.37844
0Cu 4.5-CHX+MG132	-0.25323	-0.36406	-0.37844	-0.40936

Positive values show pairs of means that are significantly different.

Connecting Letters Report

Level		Mean	Std Error
0Cu 0-CHX+MG132	A	1.0000	0.09039
0Cu 2.5-CHX+MG132	A	0.8892	0.09039
0Cu 1-CHX+MG132	A	0.8748	0.09039
0Cu 4.5-CHX+MG132	A	0.8439	0.09039

Levels not connected by same letter are significantly different.

Ordered Differences Report

Level	- Level	Difference	Std Err Dif	Lower CL	Upper CL	p-Value
0Cu 0-CHX+MG132	0Cu 4.5-CHX+MG132	0.1561303	0.1278301	-0.253226	0.5654863	0.6316
0Cu 0-CHX+MG132	0Cu 1-CHX+MG132	0.1252111	0.1278301	-0.284145	0.5345671	0.7647
0Cu 0-CHX+MG132	0Cu 2.5-CHX+MG132	0.1108353	0.1278301	-0.298521	0.5201913	0.8215
0Cu 2.5-CHX+MG132	0Cu 4.5-CHX+MG132	0.0452951	0.1278301	-0.364061	0.4546510	0.9837
0Cu 1-CHX+MG132	0Cu 4.5-CHX+MG132	0.0309192	0.1278301	-0.378437	0.4402752	0.9946
0Cu 2.5-CHX+MG132	0Cu 1-CHX+MG132	0.0143758	0.1278301	-0.394980	0.4237318	0.9994

Missing Rows 8

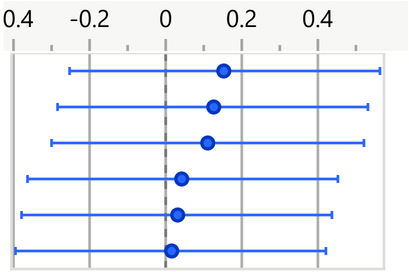
0.4
-0.2
0
0.2
0.4

Oneway Analysis of GFP By Treatment

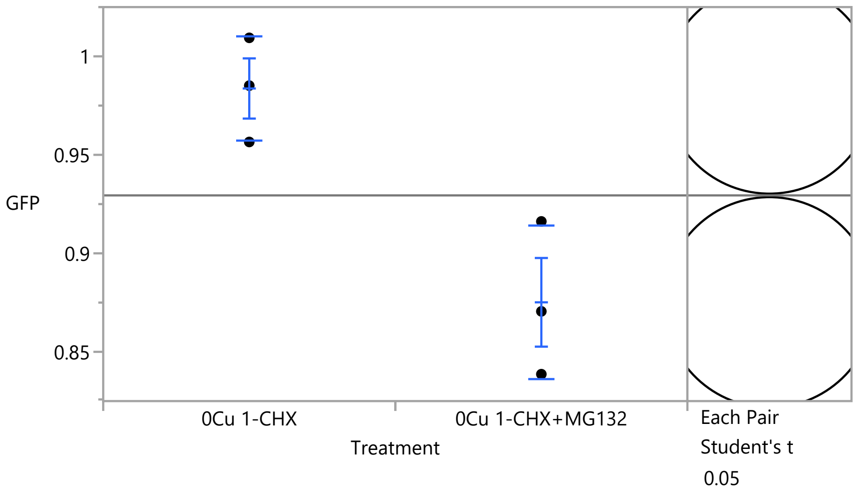

Means and Std Deviations

Level	Number	Mean	Std Dev	Std Err Mean	Lower 95%	Upper 95%
0Cu 1-CHX	3	0.9835937	0.0265186	0.0153105	0.917718	1.0494695
0Cu 1-CHX+MG132	3	0.8747889	0.0390648	0.022554	0.7777466	0.9718311

Means Comparisons

Comparisons for each pair using Student's t

Confidence Quantile

t	Alpha
2.77645	0.05

LSD Threshold Matrix

Abs(Dif)-LSD

	0Cu 1-CHX	0Cu 1-CHX+MG132
0Cu 1-CHX	-0.07569	0.03312
0Cu 1-CHX+MG132	0.03312	-0.07569

Positive values show pairs of means that are significantly different.

Connecting Letters Report

Level			Mean	Std Error
0Cu 1-CHX	A		0.98359	0.01928
0Cu 1-CHX+MG132		B	0.87479	0.01928

Levels not connected by same letter are significantly different.

Ordered Differences Report

Level	- Level	Difference	Std Err Dif	Lower CL	Upper CL	p-Value
0Cu 1-CHX	0Cu 1-CHX+MG132	0.1088049	0.0272598	0.0331195	0.1844902	0.0162*

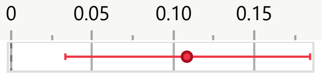

Oneway Analysis of GFP By Treatment

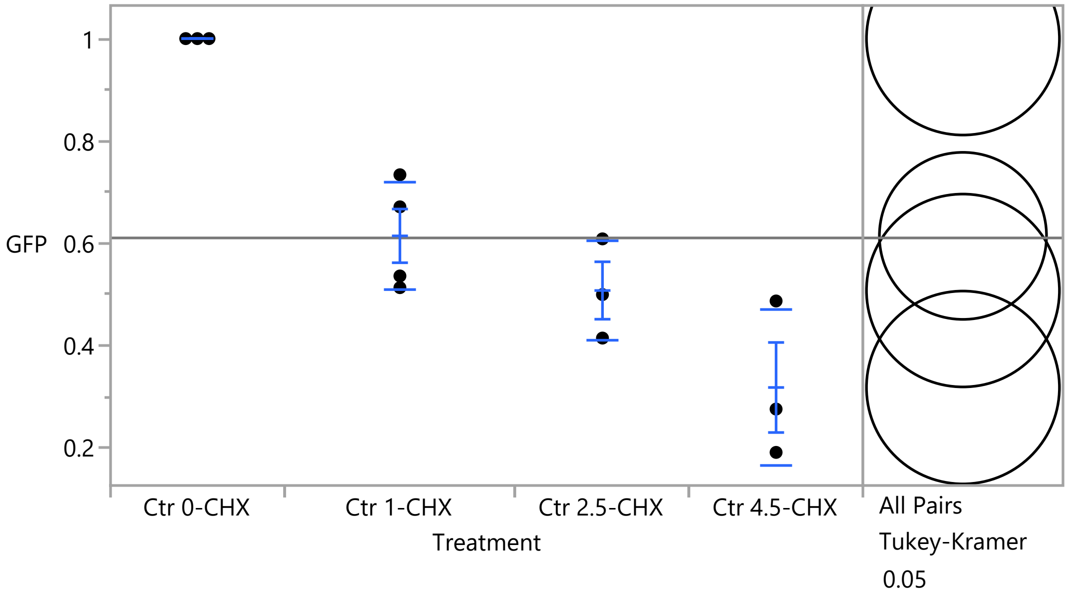

Means and Std Deviations

Level	Number	Mean	Std Dev	Std Err Mean	Lower 95%	Upper 95%
Ctr 0-CHX	3	1	0	0	1	1
Ctr 1-CHX	4	0.6144214	0.1049613	0.0524807	0.4474046	0.7814383
Ctr 2.5-CHX	3	0.5076671	0.0971941	0.056115	0.2662236	0.7491107
Ctr 4.5-CHX	3	0.3181134	0.152414	0.0879962	-0.060504	0.6967306

Oneway Analysis of GFP By Treatment

Means Comparisons

Comparisons for all pairs using Tukey-Kramer HSD

Confidence Quantile

q*	Alpha
3.12180	0.05

HSD Threshold Matrix

Abs(Dif)-HSD

	Ctr 0-CHX	Ctr 1-CHX	Ctr 2.5-CHX	Ctr 4.5-CHX
Ctr 0-CHX	-0.26653	0.13626	0.22580	0.41536
Ctr 1-CHX	0.13626	-0.23082	-0.14256	0.04699
Ctr 2.5-CHX	0.22580	-0.14256	-0.26653	-0.07698
Ctr 4.5-CHX	0.41536	0.04699	-0.07698	-0.26653

Positive values show pairs of means that are significantly different.

Connecting Letters Report

Level				Mean	Std Error
Ctr 0-CHX	A			1.0000	0.06037
Ctr 1-CHX		B		0.6144	0.05228
Ctr 2.5-CHX		B	C	0.5077	0.06037
Ctr 4.5-CHX			C	0.3181	0.06037

Levels not connected by same letter are significantly different.

Ordered Differences Report

Level	- Level	Difference	Std Err Dif	Lower CL	Upper CL	p-Value
Ctr 0-CHX	Ctr 4.5-CHX	0.6818866	0.0853767	0.415357	0.9484161	0.0001*
Ctr 0-CHX	Ctr 2.5-CHX	0.4923329	0.0853767	0.225803	0.7588624	0.0013*
Ctr 0-CHX	Ctr 1-CHX	0.3855786	0.0798626	0.136263	0.6348941	0.0042*
Ctr 1-CHX	Ctr 4.5-CHX	0.2963080	0.0798626	0.046992	0.5456236	0.0207*
Ctr 2.5-CHX	Ctr 4.5-CHX	0.1895537	0.0853767	-0.076976	0.4560832	0.1894
Ctr 1-CHX	Ctr 2.5-CHX	0.1067543	0.0798626	-0.142561	0.3560698	0.5646

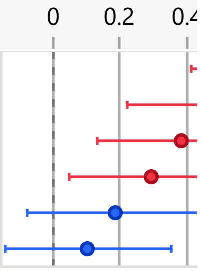

Missing Rows 7

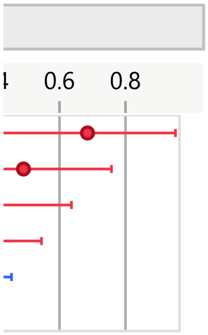
0.6
0.8

Oneway Analysis of GFP By Treatment

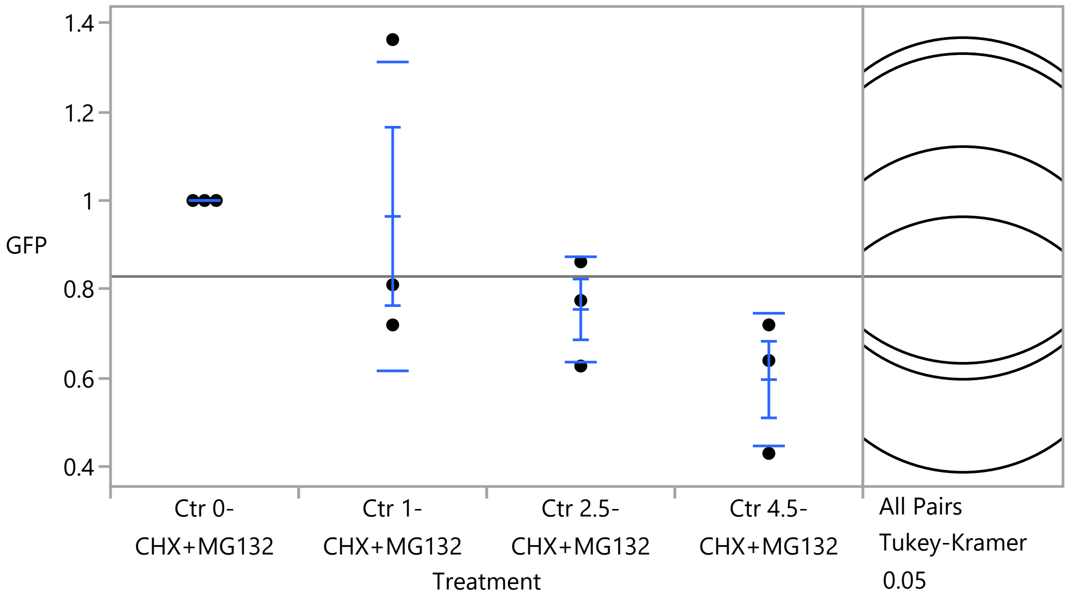

Means and Std Deviations

Level	Number	Mean	Std Dev	Std Err Mean	Lower 95%	Upper 95%
Ctr 0-CHX+MG132	3	1	0	0	1	1
Ctr 1-CHX+MG132	3	0.9641081	0.3476569	0.2007198	0.1004805	1.8277357
Ctr 2.5-CHX+MG132	3	0.7547394	0.1185691	0.0684559	0.4601973	1.0492815
Ctr 4.5-CHX+MG132	3	0.5968061	0.1493471	0.0862256	0.2258073	0.9678048

Oneway Analysis of GFP By Treatment

Means Comparisons

Comparisons for all pairs using Tukey-Kramer HSD

Confidence Quantile

q*	Alpha
3.20234	0.05

HSD Threshold Matrix

Abs(Dif)-HSD

	Ctr 0-CHX+MG132	Ctr 1-CHX+MG132	Ctr 2.5-CHX+MG132	Ctr 4.5-CHX+MG132
Ctr 0-CHX+MG132	-0.51839	-0.48250	-0.27313	-0.11520
Ctr 1-CHX+MG132	-0.48250	-0.51839	-0.30902	-0.15109
Ctr 2.5-CHX+MG132	-0.27313	-0.30902	-0.51839	-0.36046
Ctr 4.5-CHX+MG132	-0.11520	-0.15109	-0.36046	-0.51839

Positive values show pairs of means that are significantly different.

Connecting Letters Report

Level		Mean	Std Error
Ctr 0-CHX+MG132	A	1.0000	0.11447
Ctr 1-CHX+MG132	A	0.9641	0.11447
Ctr 2.5-CHX+MG132	A	0.7547	0.11447
Ctr 4.5-CHX+MG132	A	0.5968	0.11447

Levels not connected by same letter are significantly different.

Ordered Differences Report

Level	- Level	Difference	Std Err Dif	Lower CL	Upper CL	p-Value
Ctr 0-CHX+MG132	Ctr 4.5-CHX+MG132	0.4031939	0.1618788	-0.115198	0.9215856	0.1362
Ctr 1-CHX+MG132	Ctr 4.5-CHX+MG132	0.3673021	0.1618788	-0.151090	0.8856937	0.1847
Ctr 0-CHX+MG132	Ctr 2.5-CHX+MG132	0.2452606	0.1618788	-0.273131	0.7636523	0.4723
Ctr 1-CHX+MG132	Ctr 2.5-CHX+MG132	0.2093688	0.1618788	-0.309023	0.7277605	0.5914
Ctr 2.5-CHX+MG132	Ctr 4.5-CHX+MG132	0.1579333	0.1618788	-0.360458	0.6763250	0.7667
Ctr 0-CHX+MG132	Ctr 1-CHX+MG132	0.0358919	0.1618788	-0.482500	0.5542836	0.9958

Missing Rows 8

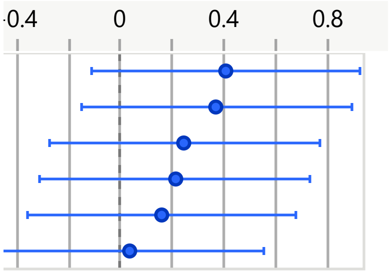
-0.4
0
0.4
0.8

Oneway Analysis of GFP By Treatment

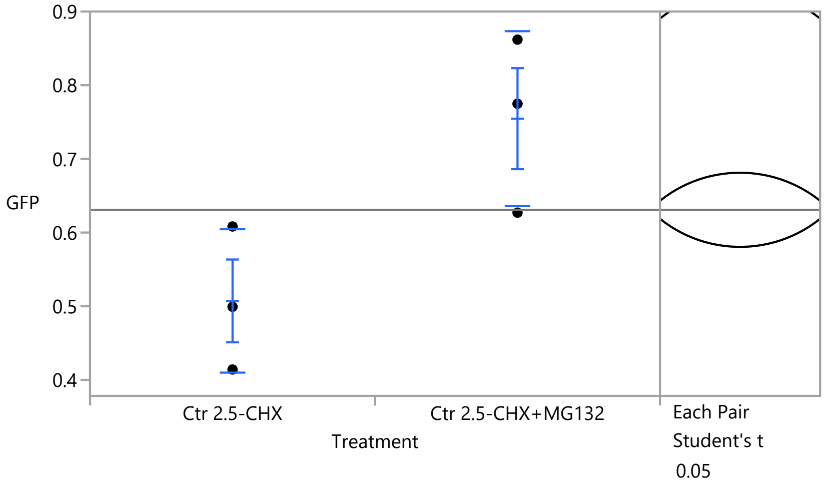

Means and Std Deviations

Level	Number	Mean	Std Dev	Std Err Mean	Lower 95%	Upper 95%
Ctr 2.5-CHX	3	0.5076671	0.0971941	0.056115	0.2662236	0.7491107
Ctr 2.5-CHX+MG132	3	0.7547394	0.1185691	0.0684559	0.4601973	1.0492815

Means Comparisons

Comparisons for each pair using Student's t

Confidence Quantile

t	Alpha
2.77645	0.05

LSD Threshold Matrix

Abs(Dif)-LSD

	Ctr 2.5-CHX+MG132	Ctr 2.5-CHX
Ctr 2.5-CHX+MG132	-0.24576	0.00131
Ctr 2.5-CHX	0.00131	-0.24576

Positive values show pairs of means that are significantly different.

Connecting Letters Report

Level			Mean	Std Error
Ctr 2.5-CHX+MG132	A		0.75474	0.06259
Ctr 2.5-CHX		B	0.50767	0.06259

Levels not connected by same letter are significantly different.

Ordered Differences Report

Level	- Level	Difference	Std Err Dif	Lower CL	Upper CL	p-Value	0 0.1 0.2 0.3 0.4
Ctr 2.5-CHX+MG132	Ctr 2.5-CHX	0.2470722	0.0885162	0.0013120	0.4928325	0.0492*	

Oneway Analysis of Myc-new blots By Treatment

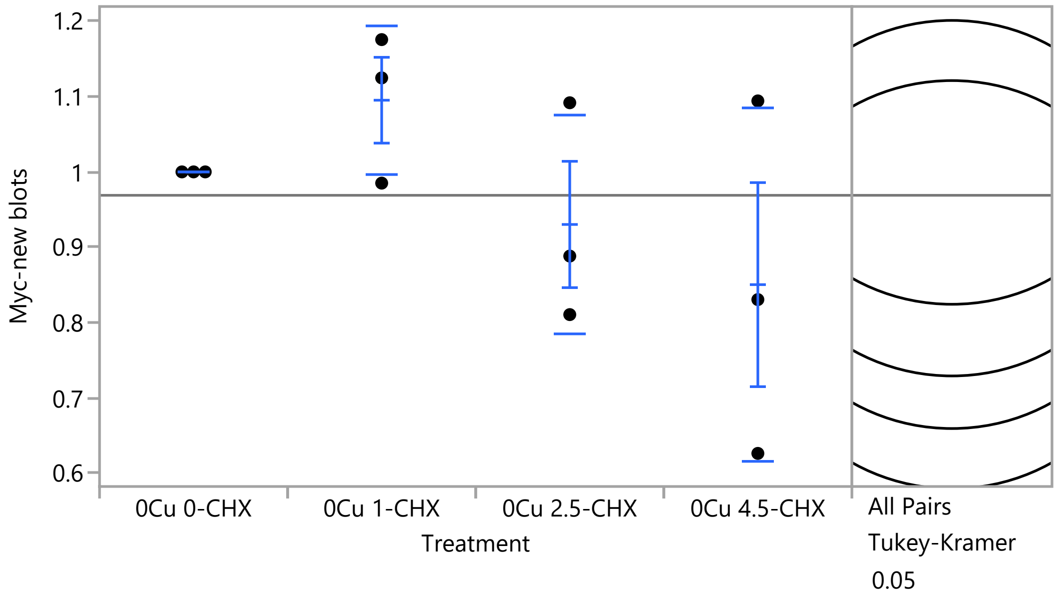

Means and Std Deviations

Level	Number	Mean	Std Dev	Std Err Mean	Lower 95%	Upper 95%
0Cu 0-CHX	3	1	0	0	1	1
0Cu 1-CHX	3	1.0952431	0.0987667	0.057023	0.849893	1.3405933
0Cu 2.5-CHX	3	0.9301094	0.1453883	0.08394	0.5689449	1.2912739
0Cu 4.5-CHX	3	0.8501648	0.2348526	0.1355922	0.2667585	1.4335711

Oneway Analysis of Myc-new blots By Treatment

Means Comparisons

Comparisons for all pairs using Tukey-Kramer HSD

Confidence Quantile

q*	Alpha
3.20234	0.05

HSD Threshold Matrix

Abs(Dif)-HSD

	0Cu 1-CHX	0Cu 0-CHX	0Cu 2.5-CHX	0Cu 4.5-CHX
0Cu 1-CHX	-0.38350	-0.28826	-0.21836	-0.13842
0Cu 0-CHX	-0.28826	-0.38350	-0.31361	-0.23366
0Cu 2.5-CHX	-0.21836	-0.31361	-0.38350	-0.30355
0Cu 4.5-CHX	-0.13842	-0.23366	-0.30355	-0.38350

Positive values show pairs of means that are significantly different.

Connecting Letters Report

Level		Mean	Std Error
0Cu 1-CHX	A	1.0952	0.08468
0Cu 0-CHX	A	1.0000	0.08468
0Cu 2.5-CHX	A	0.9301	0.08468
0Cu 4.5-CHX	A	0.8502	0.08468

Levels not connected by same letter are significantly different.

Ordered Differences Report

Level	- Level	Difference	Std Err Dif	Lower CL	Upper CL	p-Value
0Cu 1-CHX	0Cu 4.5-CHX	0.2450783	0.1197556	-0.138420	0.6285769	0.2486
0Cu 1-CHX	0Cu 2.5-CHX	0.1651337	0.1197556	-0.218365	0.5486323	0.5443
0Cu 0-CHX	0Cu 4.5-CHX	0.1498352	0.1197556	-0.233663	0.5333337	0.6150
0Cu 1-CHX	0Cu 0-CHX	0.0952431	0.1197556	-0.288255	0.4787417	0.8547
0Cu 2.5-CHX	0Cu 4.5-CHX	0.0799446	0.1197556	-0.303554	0.4634432	0.9064
0Cu 0-CHX	0Cu 2.5-CHX	0.0698906	0.1197556	-0.313608	0.4533891	0.9343

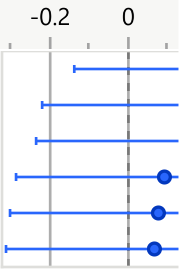

Missing Rows 8

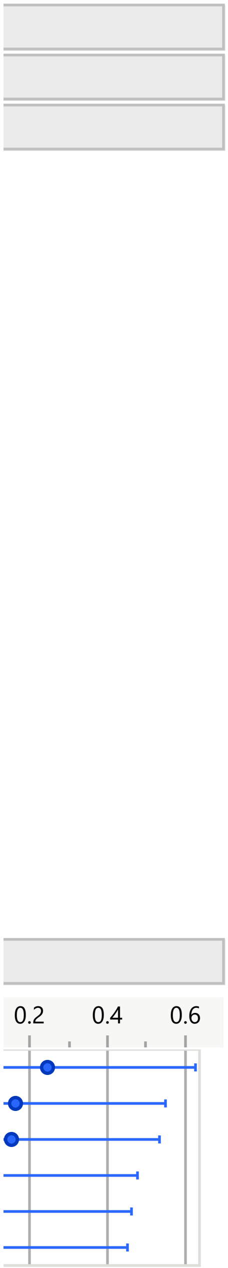
0.2
0.4
0.6

Oneway Analysis of Myc-new blots By Treatment

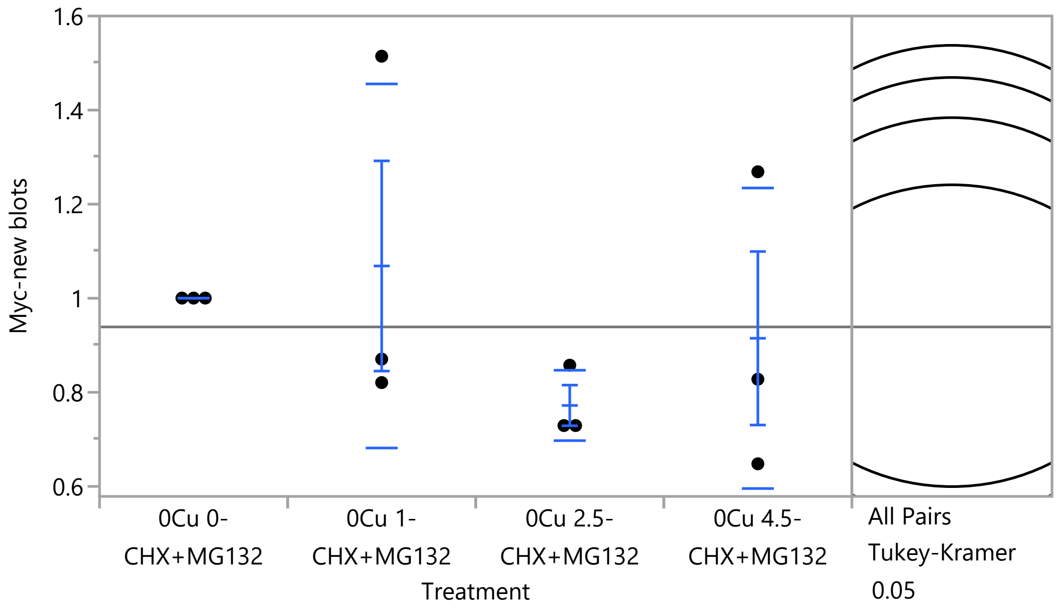

Means and Std Deviations

Level	Number	Mean	Std Dev	Std Err Mean	Lower 95%	Upper 95%
0Cu 0-CHX+MG132	3	1	0	0	1	1
0Cu 1-CHX+MG132	3	1.0683094	0.3872033	0.2235519	0.106443	2.0301757
0Cu 2.5-CHX+MG132	3	0.771651	0.0748787	0.0432312	0.5856421	0.9576599
0Cu 4.5-CHX+MG132	3	0.9145206	0.3196272	0.1845368	0.1205227	1.7085186

Oneway Analysis of Myc-new blots By Treatment

Means Comparisons

Comparisons for all pairs using Tukey-Kramer HSD

Confidence Quantile

q*	Alpha
3.20234	0.05

HSD Threshold Matrix

Abs(Dif)-HSD

	0Cu 1-CHX+MG132	0Cu 0-CHX+MG132	0Cu 4.5-CHX+MG132	0Cu 2.5-CHX+MG132
0Cu 1-CHX+MG132	-0.66366	-0.59535	-0.50987	-0.36700
0Cu 0-CHX+MG132	-0.59535	-0.66366	-0.57818	-0.43531
0Cu 4.5-CHX+MG132	-0.50987	-0.57818	-0.66366	-0.52079
0Cu 2.5-CHX+MG132	-0.36700	-0.43531	-0.52079	-0.66366

Positive values show pairs of means that are significantly different.

Connecting Letters Report

Level		Mean	Std Error
0Cu 1-CHX+MG132	A	1.0683	0.14654
0Cu 0-CHX+MG132	A	1.0000	0.14654
0Cu 4.5-CHX+MG132	A	0.9145	0.14654
0Cu 2.5-CHX+MG132	A	0.7717	0.14654

Levels not connected by same letter are significantly different.

Ordered Differences Report

Level	- Level	Difference	Std Err Dif	Lower CL	Upper CL	p-Value
0Cu 1-CHX+MG132	0Cu 2.5-CHX+MG132	0.2966584	0.2072417	-0.367001	0.9603176	0.5160
0Cu 0-CHX+MG132	0Cu 2.5-CHX+MG132	0.2283490	0.2072417	-0.435310	0.8920083	0.6984
0Cu 1-CHX+MG132	0Cu 4.5-CHX+MG132	0.1537887	0.2072417	-0.509871	0.8174480	0.8775
0Cu 4.5-CHX+MG132	0Cu 2.5-CHX+MG132	0.1428696	0.2072417	-0.520790	0.8065289	0.8983
0Cu 0-CHX+MG132	0Cu 4.5-CHX+MG132	0.0854794	0.2072417	-0.578180	0.7491386	0.9748
0Cu 1-CHX+MG132	0Cu 0-CHX+MG132	0.0683094	0.2072417	-0.595350	0.7319686	0.9867

Missing Rows 8

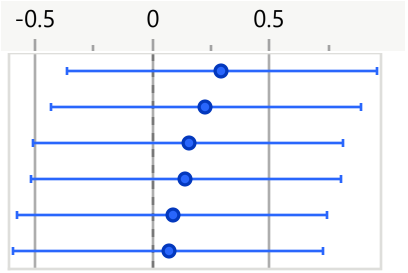
-0.5
0
0.5

Oneway Analysis of Myc By Treatment

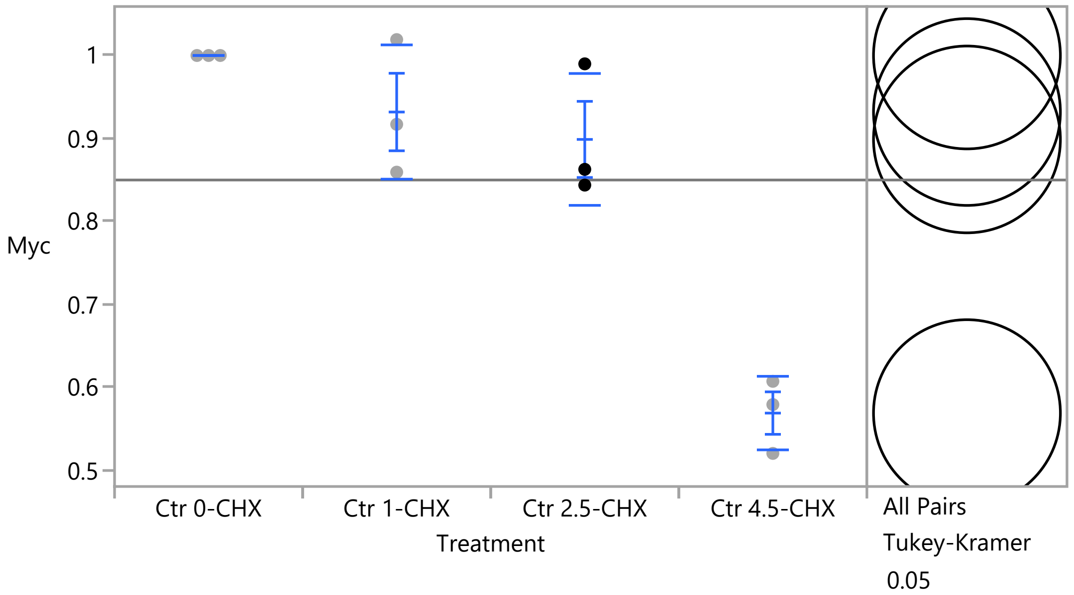

Means and Std Deviations

Level	Number	Mean	Std Dev	Std Err Mean	Lower 95%	Upper 95%
Ctr 0-CHX	3	1	0	0	1	1
Ctr 1-CHX	3	0.9317358	0.0810509	0.0467947	0.7303943	1.1330773
Ctr 2.5-CHX	3	0.8986574	0.0796107	0.0459633	0.7008934	1.0964213
Ctr 4.5-CHX	3	0.5682367	0.0444143	0.0256426	0.4579054	0.6785679

Oneway Analysis of Myc By Treatment

Means Comparisons

Comparisons for all pairs using Tukey-Kramer HSD

Confidence Quantile

q*	Alpha
3.20234	0.05

HSD Threshold Matrix

Abs(Dif)-HSD

	Ctr 0-CHX	Ctr 1-CHX	Ctr 2.5-CHX	Ctr 4.5-CHX
Ctr 0-CHX	-0.15947	-0.09121	-0.05813	0.27229
Ctr 1-CHX	-0.09121	-0.15947	-0.12640	0.20403
Ctr 2.5-CHX	-0.05813	-0.12640	-0.15947	0.17095
Ctr 4.5-CHX	0.27229	0.20403	0.17095	-0.15947

Positive values show pairs of means that are significantly different.

Connecting Letters Report

Level			Mean	Std Error
Ctr 0-CHX	A		1.0000	0.03521
Ctr 1-CHX	A		0.9317	0.03521
Ctr 2.5-CHX	A		0.8987	0.03521
Ctr 4.5-CHX		B	0.5682	0.03521

Levels not connected by same letter are significantly different.

Ordered Differences Report

Level	- Level	Difference	Std Err Dif	Lower CL	Upper CL	p-Value
Ctr 0-CHX	Ctr 4.5-CHX	0.4317633	0.0497992	0.272289	0.5912374	0.0001*
Ctr 1-CHX	Ctr 4.5-CHX	0.3634991	0.0497992	0.204025	0.5229731	0.0004*
Ctr 2.5-CHX	Ctr 4.5-CHX	0.3304207	0.0497992	0.170947	0.4898947	0.0007*
Ctr 0-CHX	Ctr 2.5-CHX	0.1013426	0.0497992	-0.058131	0.2608167	0.2523
Ctr 0-CHX	Ctr 1-CHX	0.0682642	0.0497992	-0.091210	0.2277383	0.5487
Ctr 1-CHX	Ctr 2.5-CHX	0.0330784	0.0497992	-0.126396	0.1925524	0.9076

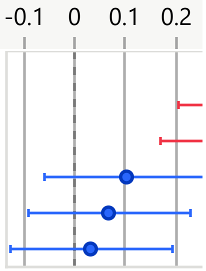

Missing Rows 8

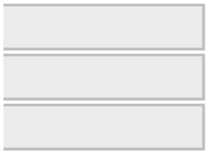

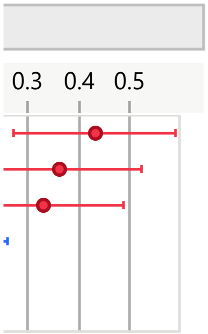
0.3
0.4
0.5

Oneway Analysis of Myc By Treatment

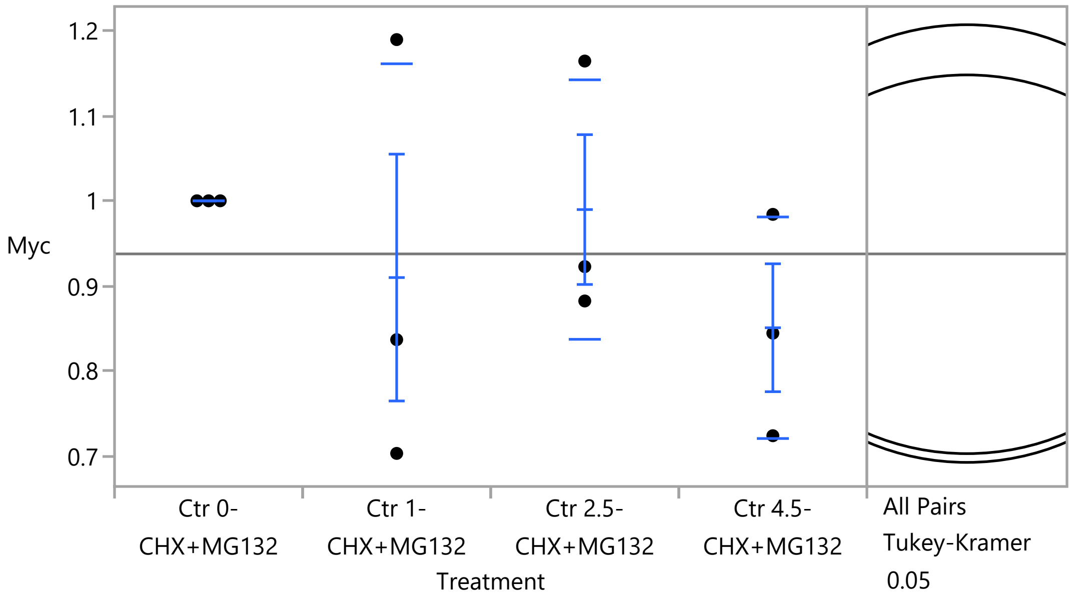

Means and Std Deviations

Level	Number	Mean	Std Dev	Std Err Mean	Lower 95%	Upper 95%
Ctr 0-CHX+MG132	3	1	0	0	1	1
Ctr 1-CHX+MG132	3	0.9097747	0.2512736	0.1450729	0.2855766	1.5339729
Ctr 2.5-CHX+MG132	3	0.9896927	0.1525069	0.0880499	0.6108447	1.3685408
Ctr 4.5-CHX+MG132	3	0.8507752	0.1301973	0.0751694	0.5273471	1.1742032

Oneway Analysis of Myc By Treatment

Means Comparisons

Comparisons for all pairs using Tukey-Kramer HSD

Confidence Quantile

q*	Alpha
3.20234	0.05

HSD Threshold Matrix

Abs(Dif)-HSD

	Ctr 0-CHX+MG132	Ctr 2.5-CHX+MG132	Ctr 1-CHX+MG132	Ctr 4.5-CHX+MG132
Ctr 0-CHX+MG132	-0.42028	-0.40998	-0.33006	-0.27106
Ctr 2.5-CHX+MG132	-0.40998	-0.42028	-0.34037	-0.28137
Ctr 1-CHX+MG132	-0.33006	-0.34037	-0.42028	-0.36129
Ctr 4.5-CHX+MG132	-0.27106	-0.28137	-0.36129	-0.42028

Positive values show pairs of means that are significantly different.

Connecting Letters Report

Level		Mean	Std Error
Ctr 0-CHX+MG132	A	1.0000	0.09280
Ctr 2.5-CHX+MG132	A	0.9897	0.09280
Ctr 1-CHX+MG132	A	0.9098	0.09280
Ctr 4.5-CHX+MG132	A	0.8508	0.09280

Levels not connected by same letter are significantly different.

Ordered Differences Report

Level	- Level	Difference	Std Err Dif	Lower CL	Upper CL	p-Value	-0.
Ctr 0-CHX+MG132	Ctr 4.5-CHX+MG132	0.1492248	0.1312428	-0.271060	0.5695095	0.6789	
Ctr 2.5-CHX+MG132	Ctr 4.5-CHX+MG132	0.1389176	0.1312428	-0.281367	0.5592023	0.7223	
Ctr 0-CHX+MG132	Ctr 1-CHX+MG132	0.0902253	0.1312428	-0.330059	0.5105100	0.8990	
Ctr 2.5-CHX+MG132	Ctr 1-CHX+MG132	0.0799180	0.1312428	-0.340367	0.5002027	0.9264	
Ctr 1-CHX+MG132	Ctr 4.5-CHX+MG132	0.0589995	0.1312428	-0.361285	0.4792843	0.9679	
Ctr 0-CHX+MG132	Ctr 2.5-CHX+MG132	0.0103073	0.1312428	-0.409977	0.4305920	0.9998	

Missing Rows 8

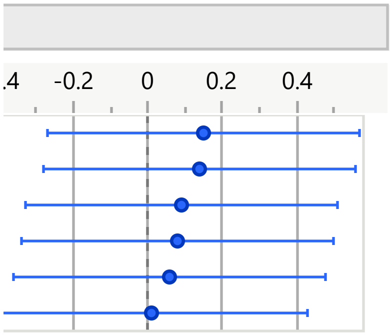
.4
-0.2
0
0.2
0.4

Oneway Analysis of myc By Treatment

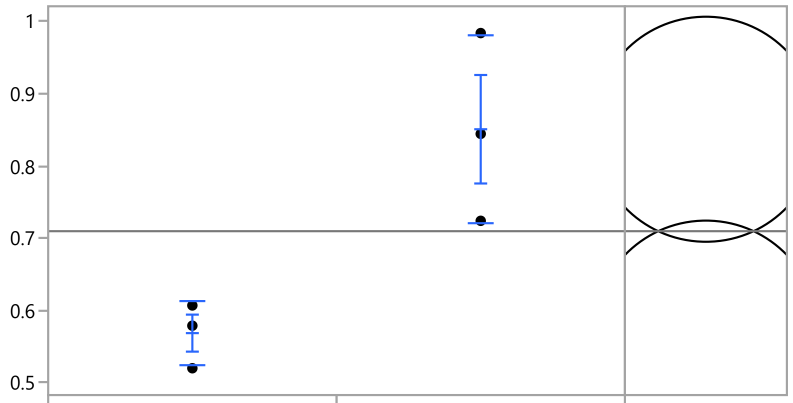

Means and Std Deviations

Level	Number	Mean	Std Dev	Std Err Mean	Lower 95%	Upper 95%
Ctr 4.5-CHX	3	0.5682367	0.0444143	0.0256426	0.4579054	0.6785679
Ctr 4.5-CHX+MG132	3	0.8507752	0.1301973	0.0751694	0.5273471	1.1742032

Means Comparisons

Comparisons for each pair using Student's t

Confidence Quantile

t	Alpha
2.77645	0.05

LSD Threshold Matrix

Abs(Dif)-LSD

	Ctr 4.5-CHX+MG132	Ctr 4.5-CHX
Ctr 4.5-CHX+MG132	-0.22051	0.06203
Ctr 4.5-CHX	0.06203	-0.22051

Positive values show pairs of means that are significantly different.

Connecting Letters Report

Level			Mean	Std Error
Ctr 4.5-CHX+MG132	A		0.85078	0.05616
Ctr 4.5-CHX		B	0.56824	0.05616

Levels not connected by same letter are significantly different.

Ordered Differences Report

Level	- Level	Difference	Std Err Dif	Lower CL	Upper CL	p-Value
Ctr 4.5-CHX+MG132	Ctr 4.5-CHX	0.2825385	0.0794229	0.0620253	0.5030517	0.0236*

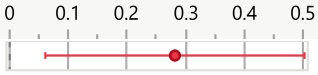

Oneway Analysis of GFP/ACT normalized by Time 0 By Treatment

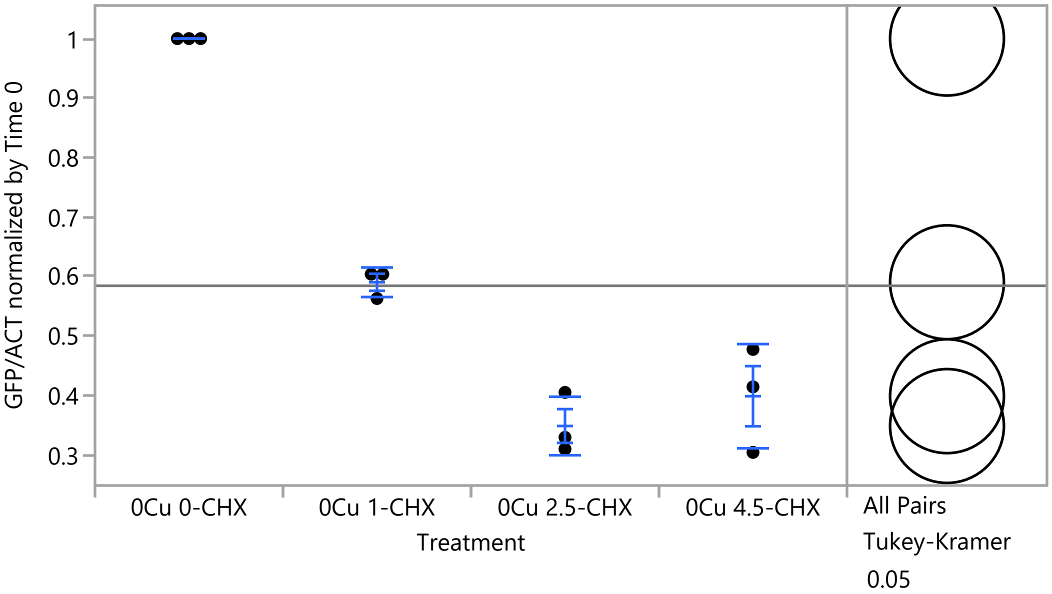

Means and Std Deviations

Level	Number	Mean	Std Dev	Std Err Mean	Lower 95%	Upper 95%
0Cu 0-CHX	3	1	0	0	1	1
0Cu 1-CHX	3	0.5902881	0.0248421	0.0143426	0.5285769	0.6519994
0Cu 2.5-CHX	3	0.3485837	0.049186	0.0283976	0.2263988	0.4707686
0Cu 4.5-CHX	3	0.3986728	0.0876549	0.0506076	0.1809259	0.6164198

Oneway Analysis of GFP/ACT normalized by Time 0 By Treatment

Means Comparisons

Comparisons for all pairs using Tukey-Kramer HSD

Confidence Quantile

q*	Alpha
3.20234	0.05

HSD Threshold Matrix

Abs(Dif)-HSD

	0Cu 0-CHX	0Cu 1-CHX	0Cu 4.5-CHX	0Cu 2.5-CHX
0Cu 0-CHX	-0.13536	0.27435	0.46597	0.51606
0Cu 1-CHX	0.27435	-0.13536	0.05626	0.10635
0Cu 4.5-CHX	0.46597	0.05626	-0.13536	-0.08527
0Cu 2.5-CHX	0.51606	0.10635	-0.08527	-0.13536

Positive values show pairs of means that are significantly different.

Connecting Letters Report

Level				Mean	Std Error
0Cu 0-CHX	A			1.0000	0.02989
0Cu 1-CHX		B		0.5903	0.02989
0Cu 4.5-CHX			C	0.3987	0.02989
0Cu 2.5-CHX			C	0.3486	0.02989

Levels not connected by same letter are significantly different.

Ordered Differences Report

Level	- Level	Difference	Std Err Dif	Lower CL	Upper CL	p-Value
0Cu 0-CHX	0Cu 2.5-CHX	0.6514163	0.0422686	0.516058	0.7867748	<.0001*
0Cu 0-CHX	0Cu 4.5-CHX	0.6013272	0.0422686	0.465969	0.7366856	<.0001*
0Cu 0-CHX	0Cu 1-CHX	0.4097119	0.0422686	0.274353	0.5450703	<.0001*
0Cu 1-CHX	0Cu 2.5-CHX	0.2417044	0.0422686	0.106346	0.3770629	0.0020*
0Cu 1-CHX	0Cu 4.5-CHX	0.1916153	0.0422686	0.056257	0.3269738	0.0083*
0Cu 4.5-CHX	0Cu 2.5-CHX	0.0500891	0.0422686	-0.085269	0.1854476	0.6520

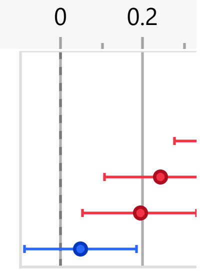

Missing Rows 55

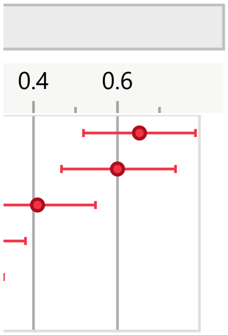
0.4
0.6

Oneway Analysis of GFP/ACT normalized by Time 0 By Treatment

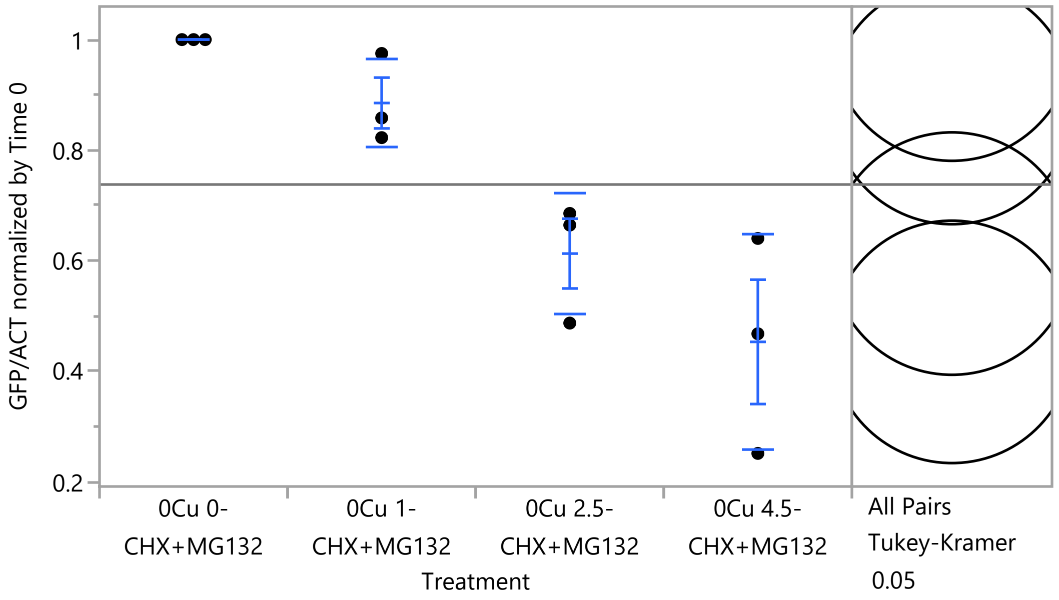

Means and Std Deviations

Level	Number	Mean	Std Dev	Std Err Mean	Lower 95%	Upper 95%
0Cu 0-CHX+MG132	3	1	0	0	1	1
0Cu 1-CHX+MG132	3	0.8853236	0.0796191	0.0459681	0.6875388	1.0831085
0Cu 2.5-CHX+MG132	3	0.6130156	0.1093091	0.0631096	0.3414767	0.8845546
0Cu 4.5-CHX+MG132	3	0.4533509	0.194805	0.1124707	-0.030572	0.9372735

Oneway Analysis of GFP/ACT normalized by Time 0 By Treatment

Means Comparisons

Comparisons for all pairs using Tukey-Kramer HSD

Confidence Quantile

q*	Alpha
3.20234	0.05

HSD Threshold Matrix

Abs(Dif)-HSD

	0Cu 0-CHX+MG132	0Cu 1-CHX+MG132	0Cu 2.5-CHX+MG132	0Cu 4.5-CHX+MG132
0Cu 0-CHX+MG132	-0.31003	-0.19535	0.07696	0.23662
0Cu 1-CHX+MG132	-0.19535	-0.31003	-0.03772	0.12194
0Cu 2.5-CHX+MG132	0.07696	-0.03772	-0.31003	-0.15036
0Cu 4.5-CHX+MG132	0.23662	0.12194	-0.15036	-0.31003

Positive values show pairs of means that are significantly different.

Connecting Letters Report

Level				Mean	Std Error
0Cu 0-CHX+MG132	A			1.0000	0.06846
0Cu 1-CHX+MG132	A	B		0.8853	0.06846
0Cu 2.5-CHX+MG132		B	C	0.6130	0.06846
0Cu 4.5-CHX+MG132			C	0.4534	0.06846

Levels not connected by same letter are significantly different.

Ordered Differences Report

Level	- Level	Difference	Std Err Dif	Lower CL	Upper CL	p-Value
0Cu 0-CHX+MG132	0Cu 4.5-CHX+MG132	0.5466491	0.0968131	0.236620	0.8566780	0.0022*
0Cu 1-CHX+MG132	0Cu 4.5-CHX+MG132	0.4319727	0.0968131	0.121944	0.7420016	0.0091*
0Cu 0-CHX+MG132	0Cu 2.5-CHX+MG132	0.3869844	0.0968131	0.076955	0.6970133	0.0167*
0Cu 1-CHX+MG132	0Cu 2.5-CHX+MG132	0.2723080	0.0968131	-0.037721	0.5823369	0.0867
0Cu 2.5-CHX+MG132	0Cu 4.5-CHX+MG132	0.1596647	0.0968131	-0.150364	0.4696937	0.4064
0Cu 0-CHX+MG132	0Cu 1-CHX+MG132	0.1146764	0.0968131	-0.195353	0.4247053	0.6523

Missing Rows 55

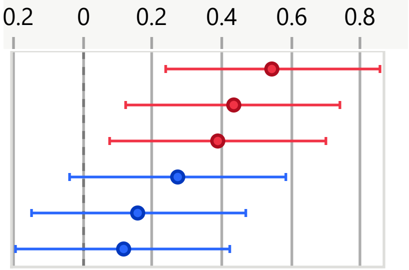
0.2
0
0.2
0.4
0.6
0.8

Oneway Analysis of GFP/ACT By Treatment

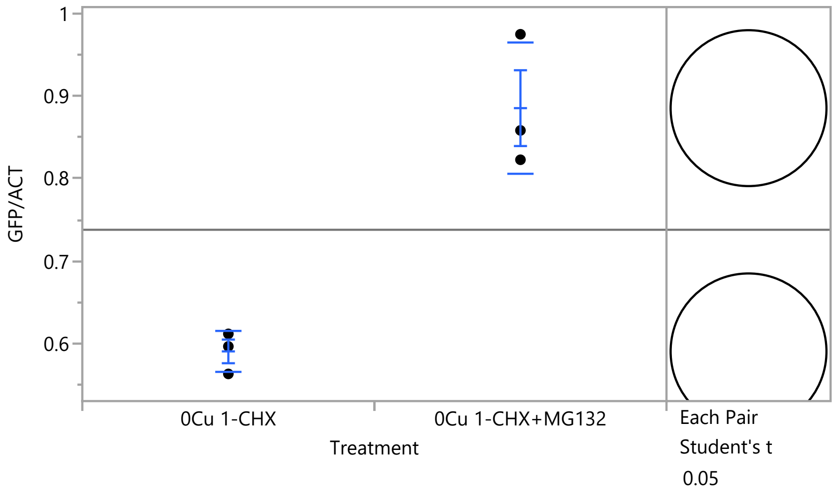

Means and Std Deviations

Level	Number	Mean	Std Dev	Std Err Mean	Lower 95%	Upper 95%
0Cu 1-CHX	3	0.5902881	0.0248421	0.0143426	0.5285769	0.6519994
0Cu 1-CHX+MG132	3	0.8853236	0.0796191	0.0459681	0.6875388	1.0831085

Means Comparisons

Comparisons for each pair using Student's t

Confidence Quantile

t	Alpha
2.77645	0.05

LSD Threshold Matrix

Abs(Dif)-LSD

	0Cu 1-CHX+MG132	0Cu 1-CHX
0Cu 1-CHX+MG132	-0.13370	0.16134
0Cu 1-CHX	0.16134	-0.13370

Positive values show pairs of means that are significantly different.

Connecting Letters Report

Level			Mean	Std Error
0Cu 1-CHX+MG132	A		0.88532	0.03405
0Cu 1-CHX		B	0.59029	0.03405

Levels not connected by same letter are significantly different.

Ordered Differences Report

Level	- Level	Difference	Std Err Dif	Lower CL	Upper CL	p-Value
0Cu 1-CHX+MG132	0Cu 1-CHX	0.2950355	0.0481537	0.1613394	0.4287316	0.0036*

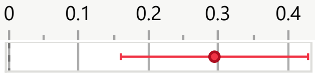

Oneway Analysis of GFP/ACT By Treatment

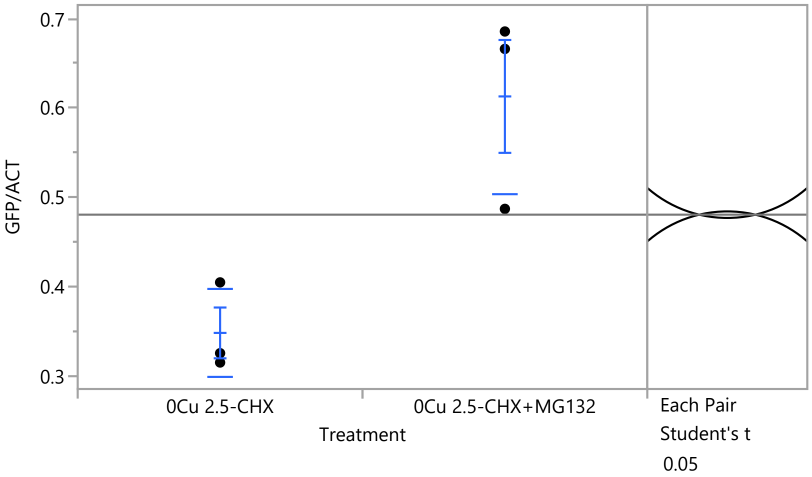

Means and Std Deviations

Level	Number	Mean	Std Dev	Std Err Mean	Lower 95%	Upper 95%
0Cu 2.5-CHX	3	0.3485837	0.049186	0.0283976	0.2263988	0.4707686
0Cu 2.5-CHX+MG132	3	0.6130156	0.1093091	0.0631096	0.3414767	0.8845546

Means Comparisons

Comparisons for each pair using Student's t

Confidence Quantile

t	Alpha
2.77645	0.05

LSD Threshold Matrix

Abs(Dif)-LSD

	0Cu 2.5-CHX+MG132	0Cu 2.5-CHX
0Cu 2.5-CHX+MG132	-0.19214	0.07229
0Cu 2.5-CHX	0.07229	-0.19214

Positive values show pairs of means that are significantly different.

Connecting Letters Report

Level			Mean	Std Error
0Cu 2.5-CHX+MG132	A		0.61302	0.04893
0Cu 2.5-CHX		B	0.34858	0.04893

Levels not connected by same letter are significantly different.

Ordered Differences Report

Level	- Level	Difference	Std Err Dif	Lower CL	Upper CL	p-Value
0Cu 2.5-CHX+MG132	0Cu 2.5-CHX	0.2644320	0.0692044	0.0722897	0.4565742	0.0188*

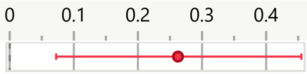

Oneway Analysis of GFP/ACT normalized by Time 0 By Treatment

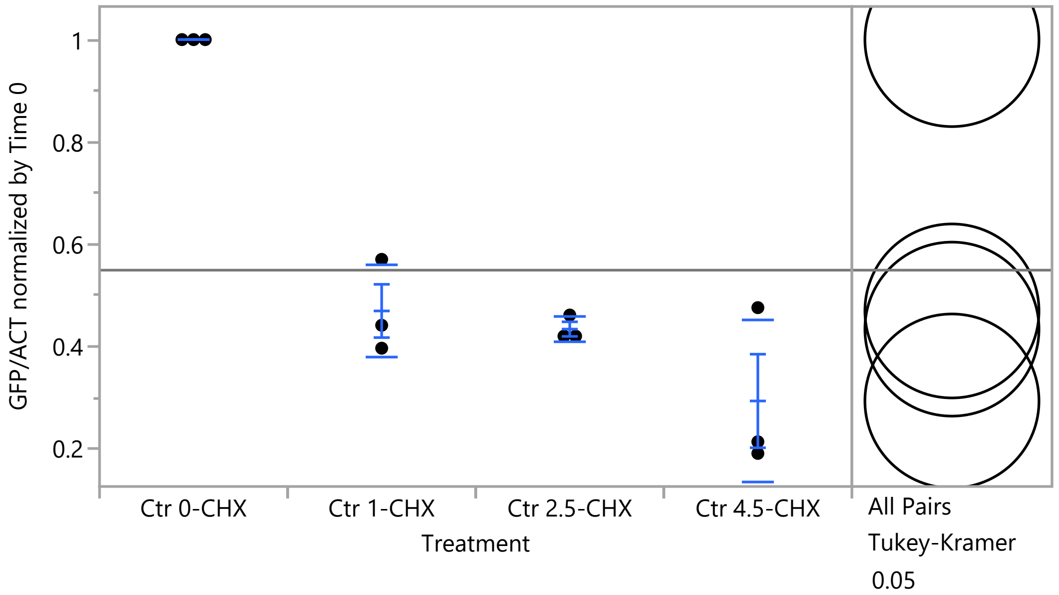

Means and Std Deviations

Level	Number	Mean	Std Dev	Std Err Mean	Lower 95%	Upper 95%
Ctr 0-CHX	3	1	0	0	1	1
Ctr 1-CHX	3	0.4697957	0.0901243	0.0520333	0.2459144	0.693677
Ctr 2.5-CHX	3	0.4343072	0.0247246	0.0142748	0.3728878	0.4957267
Ctr 4.5-CHX	3	0.2938872	0.1583284	0.0914109	-0.099422	0.6871968

Oneway Analysis of GFP/ACT normalized by Time 0 By Treatment

Means Comparisons

Comparisons for all pairs using Tukey-Kramer HSD

Confidence Quantile

q*	Alpha
3.20234	0.05

HSD Threshold Matrix

Abs(Dif)-HSD

	Ctr 0-CHX	Ctr 1-CHX	Ctr 2.5-CHX	Ctr 4.5-CHX
Ctr 0-CHX	-0.24036	0.28984	0.32533	0.46575
Ctr 1-CHX	0.28984	-0.24036	-0.20487	-0.06445
Ctr 2.5-CHX	0.32533	-0.20487	-0.24036	-0.09994
Ctr 4.5-CHX	0.46575	-0.06445	-0.09994	-0.24036

Positive values show pairs of means that are significantly different.

Connecting Letters Report

Level			Mean	Std Error
Ctr 0-CHX	A		1.0000	0.05307
Ctr 1-CHX		B	0.4698	0.05307
Ctr 2.5-CHX		B	0.4343	0.05307
Ctr 4.5-CHX		B	0.2939	0.05307

Levels not connected by same letter are significantly different.

Ordered Differences Report

Level	- Level	Difference	Std Err Dif	Lower CL	Upper CL	p-Value
Ctr 0-CHX	Ctr 4.5-CHX	0.7061128	0.0750573	0.465753	0.9464721	<.0001*
Ctr 0-CHX	Ctr 2.5-CHX	0.5656928	0.0750573	0.325333	0.8060521	0.0003*
Ctr 0-CHX	Ctr 1-CHX	0.5302043	0.0750573	0.289845	0.7705636	0.0005*
Ctr 1-CHX	Ctr 4.5-CHX	0.1759085	0.0750573	-0.064451	0.4162678	0.1668
Ctr 2.5-CHX	Ctr 4.5-CHX	0.1404200	0.0750573	-0.099939	0.3807793	0.3112
Ctr 1-CHX	Ctr 2.5-CHX	0.0354885	0.0750573	-0.204871	0.2758478	0.9630

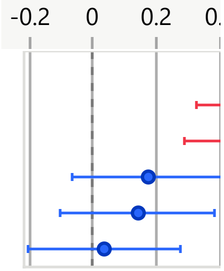

Missing Rows 55

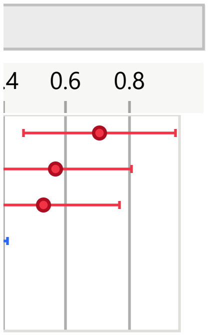
.4
0.6
0.8

Oneway Analysis of GFP/ACT normalized by Time 0 By Treatment

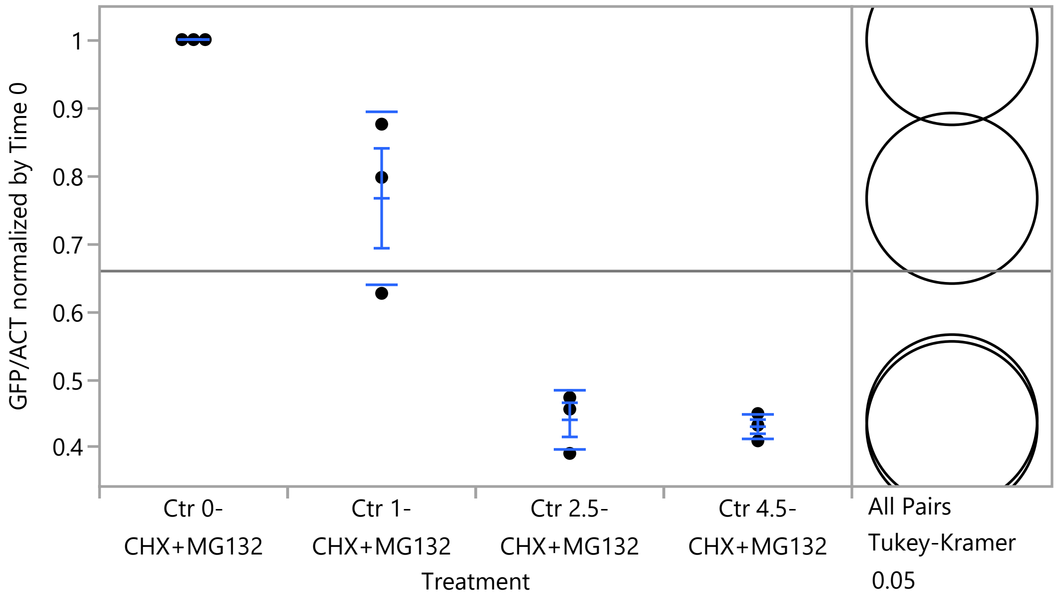

Means and Std Deviations

Level	Number	Mean	Std Dev	Std Err Mean	Lower 95%	Upper 95%
Ctr 0-CHX+MG132	3	1	0	0	1	1
Ctr 1-CHX+MG132	3	0.7665612	0.1272345	0.0734588	0.4504933	1.0826291
Ctr 2.5-CHX+MG132	3	0.4406602	0.0435149	0.0251234	0.332563	0.5487573
Ctr 4.5-CHX+MG132	3	0.4306409	0.0180281	0.0104085	0.3858566	0.4754251

Oneway Analysis of GFP/ACT normalized by Time 0 By Treatment

Means Comparisons

Comparisons for all pairs using Tukey-Kramer HSD

Confidence Quantile

q*	Alpha
3.20234	0.05

HSD Threshold Matrix

Abs(Dif)-HSD

	Ctr 0-CHX+MG132	Ctr 1-CHX+MG132	Ctr 2.5-CHX+MG132	Ctr 4.5-CHX+MG132
Ctr 0-CHX+MG132	-0.17737	0.05607	0.38197	0.39199
Ctr 1-CHX+MG132	0.05607	-0.17737	0.14853	0.15855
Ctr 2.5-CHX+MG132	0.38197	0.14853	-0.17737	-0.16735
Ctr 4.5-CHX+MG132	0.39199	0.15855	-0.16735	-0.17737

Positive values show pairs of means that are significantly different.

Connecting Letters Report

Level				Mean	Std Error
Ctr 0-CHX+MG132	A			1.0000	0.03917
Ctr 1-CHX+MG132		B		0.7666	0.03917
Ctr 2.5-CHX+MG132			C	0.4407	0.03917
Ctr 4.5-CHX+MG132			C	0.4306	0.03917

Levels not connected by same letter are significantly different.

Ordered Differences Report

Level	- Level	Difference	Std Err Dif	Lower CL	Upper CL	p-Value
Ctr 0-CHX+MG132	Ctr 4.5-CHX+MG132	0.5693591	0.0553883	0.391987	0.7467315	<.0001*
Ctr 0-CHX+MG132	Ctr 2.5-CHX+MG132	0.5593398	0.0553883	0.381968	0.7367122	<.0001*
Ctr 1-CHX+MG132	Ctr 4.5-CHX+MG132	0.3359203	0.0553883	0.158548	0.5132926	0.0014*
Ctr 1-CHX+MG132	Ctr 2.5-CHX+MG132	0.3259010	0.0553883	0.148529	0.5032734	0.0017*
Ctr 0-CHX+MG132	Ctr 1-CHX+MG132	0.2334388	0.0553883	0.056066	0.4108111	0.0125*
Ctr 2.5-CHX+MG132	Ctr 4.5-CHX+MG132	0.0100193	0.0553883	-0.167353	0.1873916	0.9977

Missing Rows 55

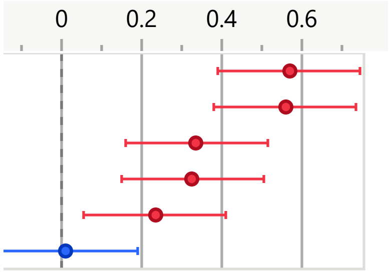
0
0.2
0.4
0.6

Oneway Analysis of GFP/ACT By Treatment

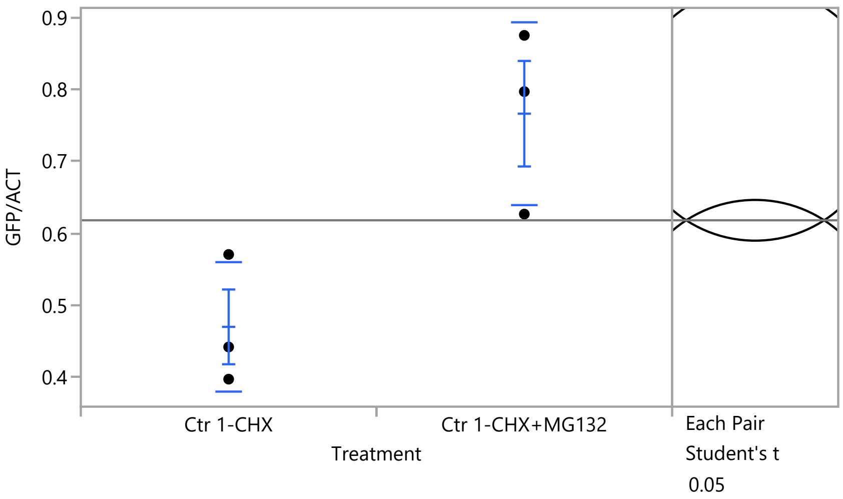

Means and Std Deviations

Level	Number	Mean	Std Dev	Std Err Mean	Lower 95%	Upper 95%
Ctr 1-CHX	3	0.4697957	0.0901243	0.0520333	0.2459144	0.693677
Ctr 1-CHX+MG132	3	0.7665612	0.1272345	0.0734588	0.4504933	1.0826291

Means Comparisons

Comparisons for each pair using Student's t

Confidence Quantile

t	Alpha
2.77645	0.05

LSD Threshold Matrix

Abs(Dif)-LSD

	Ctr 1-CHX+MG132	Ctr 1-CHX
Ctr 1-CHX+MG132	-0.24994	0.04683
Ctr 1-CHX	0.04683	-0.24994

Positive values show pairs of means that are significantly different.

Connecting Letters Report

Level			Mean	Std Error
Ctr 1-CHX+MG132	A		0.76656	0.06365
Ctr 1-CHX		B	0.46980	0.06365

Levels not connected by same letter are significantly different.

Ordered Differences Report

Level	- Level	Difference	Std Err Dif	Lower CL	Upper CL	p-Value	
Ctr 1-CHX+MG132	Ctr 1-CHX	0.2967655	0.0900204	0.0468288	0.5467021	0.0300*	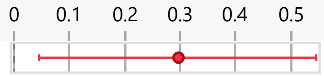

Oneway Analysis of MYC/ACT normalized by Time 0 By Treatment

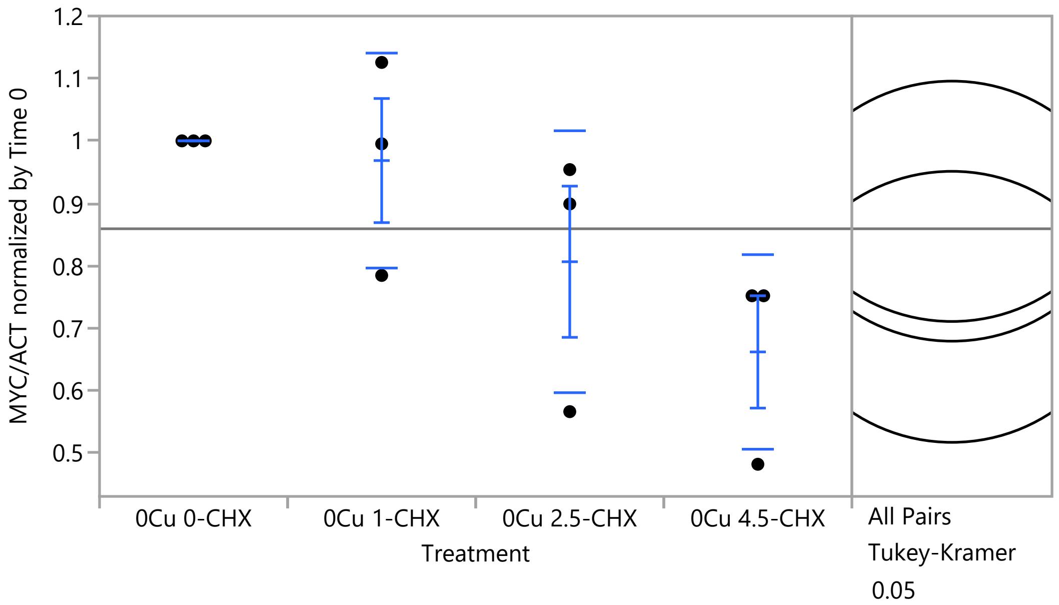

Means and Std Deviations

Level	Number	Mean	Std Dev	Std Err Mean	Lower 95%	Upper 95%
0Cu 0-CHX	3	1	0	0	1	1
0Cu 1-CHX	3	0.9684175	0.1721966	0.0994177	0.5406575	1.3961775
0Cu 2.5-CHX	3	0.8063479	0.2098111	0.1211345	0.2851482	1.3275476
0Cu 4.5-CHX	3	0.6618481	0.1558625	0.0899873	0.2746641	1.0490321

Oneway Analysis of MYC/ACT normalized by Time 0 By Treatment

Means Comparisons

Comparisons for all pairs using Tukey-Kramer HSD

Confidence Quantile

q*	Alpha
3.20234	0.05

HSD Threshold Matrix

Abs(Dif)-HSD

	0Cu 0-CHX	0Cu 1-CHX	0Cu 2.5-CHX	0Cu 4.5-CHX
0Cu 0-CHX	-0.40919	-0.37761	-0.21554	-0.07104
0Cu 1-CHX	-0.37761	-0.40919	-0.24712	-0.10262
0Cu 2.5-CHX	-0.21554	-0.24712	-0.40919	-0.26469
0Cu 4.5-CHX	-0.07104	-0.10262	-0.26469	-0.40919

Positive values show pairs of means that are significantly different.

Connecting Letters Report

Level		Mean	Std Error
0Cu 0-CHX	A	1.0000	0.09035
0Cu 1-CHX	A	0.9684	0.09035
0Cu 2.5-CHX	A	0.8063	0.09035
0Cu 4.5-CHX	A	0.6618	0.09035

Levels not connected by same letter are significantly different.

Ordered Differences Report

Level	- Level	Difference	Std Err Dif	Lower CL	Upper CL	p-Value
0Cu 0-CHX	0Cu 4.5-CHX	0.3381519	0.1277794	-0.071042	0.7473456	0.1095
0Cu 1-CHX	0Cu 4.5-CHX	0.3065695	0.1277794	-0.102624	0.7157631	0.1546
0Cu 0-CHX	0Cu 2.5-CHX	0.1936521	0.1277794	-0.215542	0.6028458	0.4721
0Cu 1-CHX	0Cu 2.5-CHX	0.1620696	0.1277794	-0.247124	0.5712633	0.6054
0Cu 2.5-CHX	0Cu 4.5-CHX	0.1444998	0.1277794	-0.264694	0.5536935	0.6823
0Cu 0-CHX	0Cu 1-CHX	0.0315825	0.1277794	-0.377611	0.4407762	0.9943

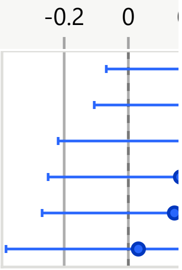

Missing Rows 4

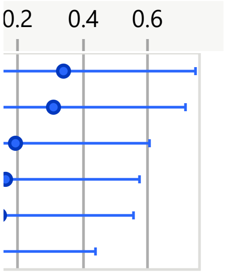
0.2
0.4
0.6

Oneway Analysis of MYC/ACT normalized by Time 0 By Treatment

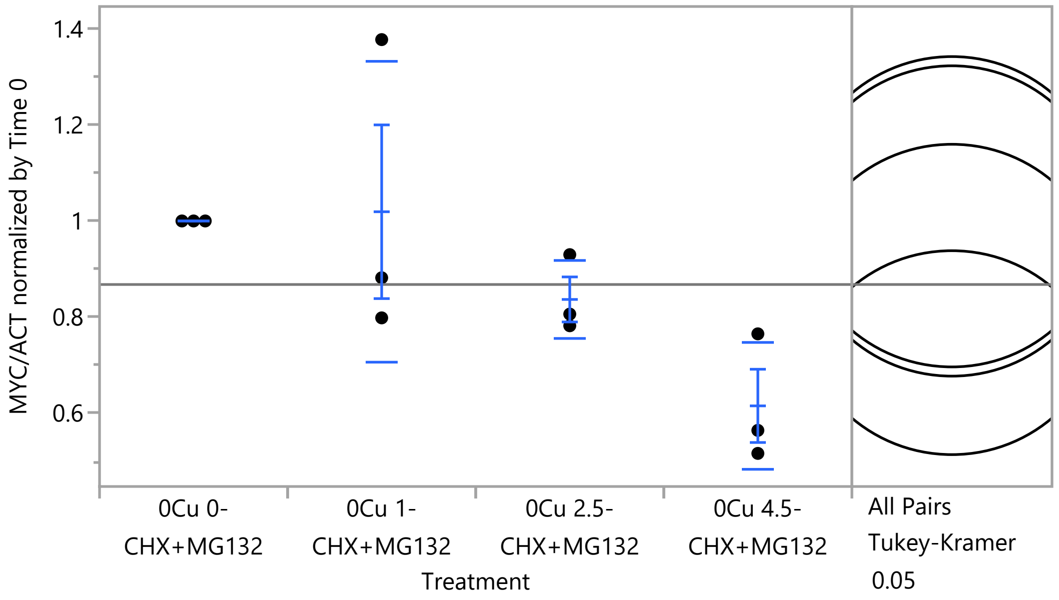

Means and Std Deviations

Level	Number	Mean	Std Dev	Std Err Mean	Lower 95%	Upper 95%
0Cu 0-CHX+MG132	3	1	0	0	1	1
0Cu 1-CHX+MG132	3	1.0191401	0.312517	0.1804318	0.2428047	1.7954754
0Cu 2.5-CHX+MG132	3	0.8369367	0.0810243	0.0467794	0.6356612	1.0382121
0Cu 4.5-CHX+MG132	3	0.6158411	0.1317878	0.0760877	0.2884621	0.94322

Oneway Analysis of MYC/ACT normalized by Time 0 By Treatment

Means Comparisons

Comparisons for all pairs using Tukey-Kramer HSD

Confidence Quantile

q*	Alpha
3.20234	0.05

HSD Threshold Matrix

Abs(Dif)-HSD

	0Cu 1-CHX+MG132	0Cu 0-CHX+MG132	0Cu 2.5-CHX+MG132	0Cu 4.5-CHX+MG132
0Cu 1-CHX+MG132	-0.45589	-0.43675	-0.27369	-0.05259
0Cu 0-CHX+MG132	-0.43675	-0.45589	-0.29283	-0.07173
0Cu 2.5-CHX+MG132	-0.27369	-0.29283	-0.45589	-0.23479
0Cu 4.5-CHX+MG132	-0.05259	-0.07173	-0.23479	-0.45589

Positive values show pairs of means that are significantly different.

Connecting Letters Report

Level		Mean	Std Error
0Cu 1-CHX+MG132	A	1.0191	0.10066
0Cu 0-CHX+MG132	A	1.0000	0.10066
0Cu 2.5-CHX+MG132	A	0.8369	0.10066
0Cu 4.5-CHX+MG132	A	0.6158	0.10066

Levels not connected by same letter are significantly different.

Ordered Differences Report

Level	- Level	Difference	Std Err Dif	Lower CL	Upper CL	p-Value
0Cu 1-CHX+MG132	0Cu 4.5-CHX+MG132	0.4032990	0.1423610	-0.052590	0.8591877	0.0842
0Cu 0-CHX+MG132	0Cu 4.5-CHX+MG132	0.3841589	0.1423610	-0.071730	0.8400477	0.1018
0Cu 2.5-CHX+MG132	0Cu 4.5-CHX+MG132	0.2210956	0.1423610	-0.234793	0.6769844	0.4531
0Cu 1-CHX+MG132	0Cu 2.5-CHX+MG132	0.1822034	0.1423610	-0.273685	0.6380922	0.5989
0Cu 0-CHX+MG132	0Cu 2.5-CHX+MG132	0.1630633	0.1423610	-0.292825	0.6189521	0.6742
0Cu 1-CHX+MG132	0Cu 0-CHX+MG132	0.0191401	0.1423610	-0.436749	0.4750288	0.9991

Missing Rows 4

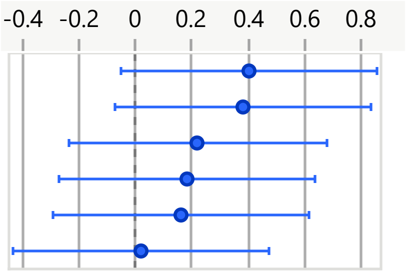
-0.4 -0.2 0 0.2 0.4 0.6 0.8

Oneway Analysis of MYC/ACT normalized by Time 0 By Treatment

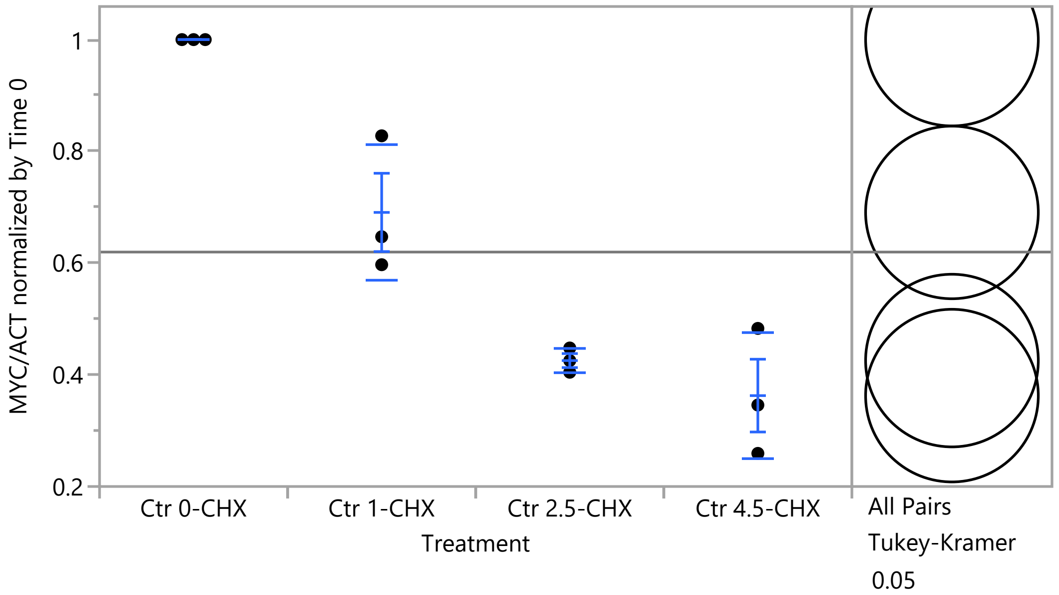

Means and Std Deviations

Level	Number	Mean	Std Dev	Std Err Mean	Lower 95%	Upper 95%
Ctr 0-CHX	3	1	0	0	1	1
Ctr 1-CHX	3	0.6904332	0.1214635	0.070127	0.3887012	0.9921651
Ctr 2.5-CHX	3	0.4250468	0.0217522	0.0125586	0.3710114	0.4790822
Ctr 4.5-CHX	3	0.362286	0.1129007	0.0651832	0.0818253	0.6427468

Oneway Analysis of MYC/ACT normalized by Time 0 By Treatment

Means Comparisons

Comparisons for all pairs using Tukey-Kramer HSD

Confidence Quantile

q*	Alpha
3.20234	0.05

HSD Threshold Matrix

Abs(Dif)-HSD

	Ctr 0-CHX	Ctr 1-CHX	Ctr 2.5-CHX	Ctr 4.5-CHX
Ctr 0-CHX	-0.21866	0.09091	0.35630	0.41906
Ctr 1-CHX	0.09091	-0.21866	0.04673	0.10949
Ctr 2.5-CHX	0.35630	0.04673	-0.21866	-0.15590
Ctr 4.5-CHX	0.41906	0.10949	-0.15590	-0.21866

Positive values show pairs of means that are significantly different.

Connecting Letters Report

Level				Mean	Std Error
Ctr 0-CHX	A			1.0000	0.04828
Ctr 1-CHX		B		0.6904	0.04828
Ctr 2.5-CHX			C	0.4250	0.04828
Ctr 4.5-CHX			C	0.3623	0.04828

Levels not connected by same letter are significantly different.

Ordered Differences Report

Level	- Level	Difference	Std Err Dif	Lower CL	Upper CL	p-Value
Ctr 0-CHX	Ctr 4.5-CHX	0.6377140	0.0682802	0.419057	0.8563706	<.0001*
Ctr 0-CHX	Ctr 2.5-CHX	0.5749532	0.0682802	0.356297	0.7936098	0.0001*
Ctr 1-CHX	Ctr 4.5-CHX	0.3281471	0.0682802	0.109491	0.5468037	0.0059*
Ctr 0-CHX	Ctr 1-CHX	0.3095668	0.0682802	0.090910	0.5282234	0.0083*
Ctr 1-CHX	Ctr 2.5-CHX	0.2653864	0.0682802	0.046730	0.4840430	0.0194*
Ctr 2.5-CHX	Ctr 4.5-CHX	0.0627608	0.0682802	-0.155896	0.2814173	0.7958

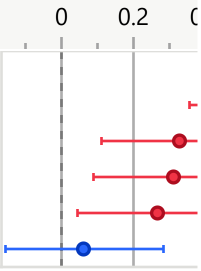

Missing Rows 4

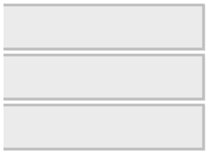

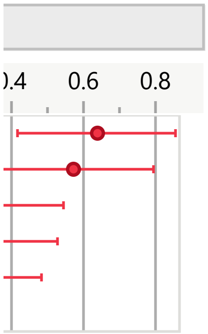
).4
0.6
0.8

Oneway Analysis of MYC/ACT normalized by Time 0 By Treatment

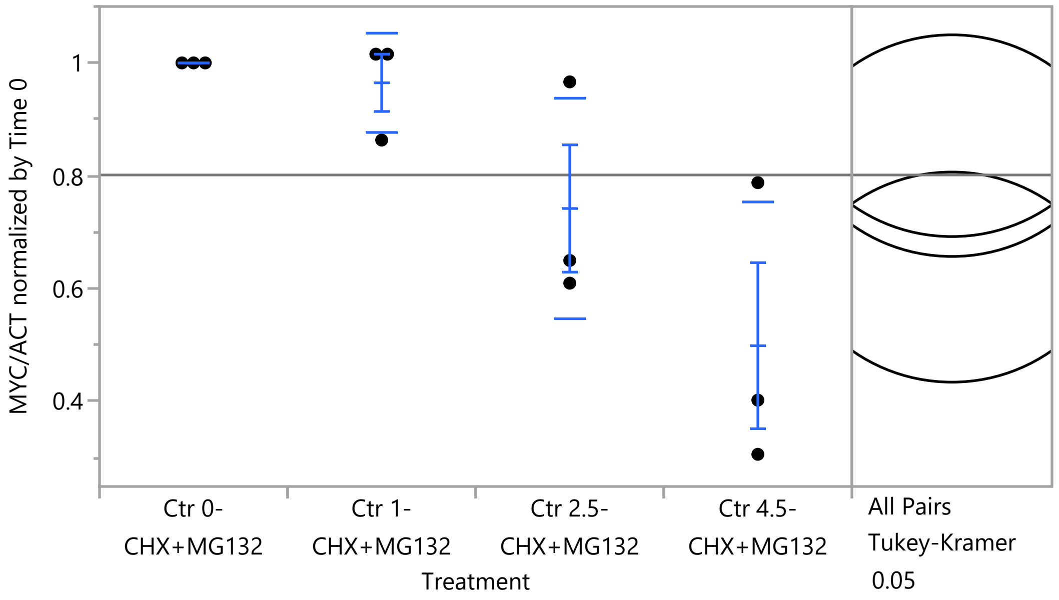

Means and Std Deviations

Level	Number	Mean	Std Dev	Std Err Mean	Lower 95%	Upper 95%
Ctr 0-CHX+MG132	3	1	0	0	1	1
Ctr 1-CHX+MG132	3	0.9647176	0.0880068	0.0508107	0.7460967	1.1833385
Ctr 2.5-CHX+MG132	3	0.7419081	0.1954488	0.1128424	0.2563864	1.2274298
Ctr 4.5-CHX+MG132	3	0.4986429	0.2548858	0.1471584	-0.134529	1.1318143

Oneway Analysis of MYC/ACT normalized by Time 0 By Treatment

Means Comparisons

Comparisons for all pairs using Tukey-Kramer HSD

Confidence Quantile

q*	Alpha
3.20234	0.05

HSD Threshold Matrix

Abs(Dif)-HSD

	Ctr 0-CHX+MG132	Ctr 1-CHX+MG132	Ctr 2.5-CHX+MG132	Ctr 4.5-CHX+MG132
Ctr 0-CHX+MG132	-0.43539	-0.40011	-0.17730	0.06596
Ctr 1-CHX+MG132	-0.40011	-0.43539	-0.21258	0.03068
Ctr 2.5-CHX+MG132	-0.17730	-0.21258	-0.43539	-0.19213
Ctr 4.5-CHX+MG132	0.06596	0.03068	-0.19213	-0.43539

Positive values show pairs of means that are significantly different.

Connecting Letters Report

Level			Mean	Std Error
Ctr 0-CHX+MG132	A		1.0000	0.09614
Ctr 1-CHX+MG132	A		0.9647	0.09614
Ctr 2.5-CHX+MG132	A	B	0.7419	0.09614
Ctr 4.5-CHX+MG132		B	0.4986	0.09614

Levels not connected by same letter are significantly different.

Ordered Differences Report

Level	- Level	Difference	Std Err Dif	Lower CL	Upper CL	p-Value
Ctr 0-CHX+MG132	Ctr 4.5-CHX+MG132	0.5013571	0.1359609	0.065964	0.9367507	0.0255*
Ctr 1-CHX+MG132	Ctr 4.5-CHX+MG132	0.4660747	0.1359609	0.030681	0.9014683	0.0364*
Ctr 0-CHX+MG132	Ctr 2.5-CHX+MG132	0.2580919	0.1359609	-0.177302	0.6934855	0.3007
Ctr 2.5-CHX+MG132	Ctr 4.5-CHX+MG132	0.2432652	0.1359609	-0.192128	0.6786588	0.3442
Ctr 1-CHX+MG132	Ctr 2.5-CHX+MG132	0.2228095	0.1359609	-0.212584	0.6582031	0.4113
Ctr 0-CHX+MG132	Ctr 1-CHX+MG132	0.0352824	0.1359609	-0.400111	0.4706760	0.9934

Missing Rows 4

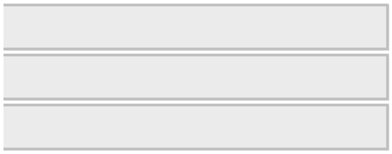

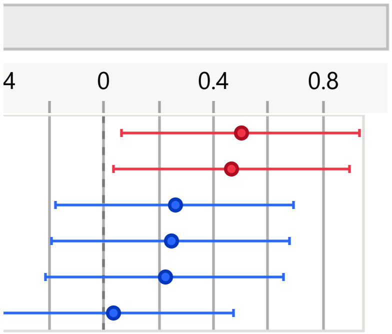
4
0
0.4
0.8

Oneway Analysis of MYC/ACT normalized by Time 0 By Treatment

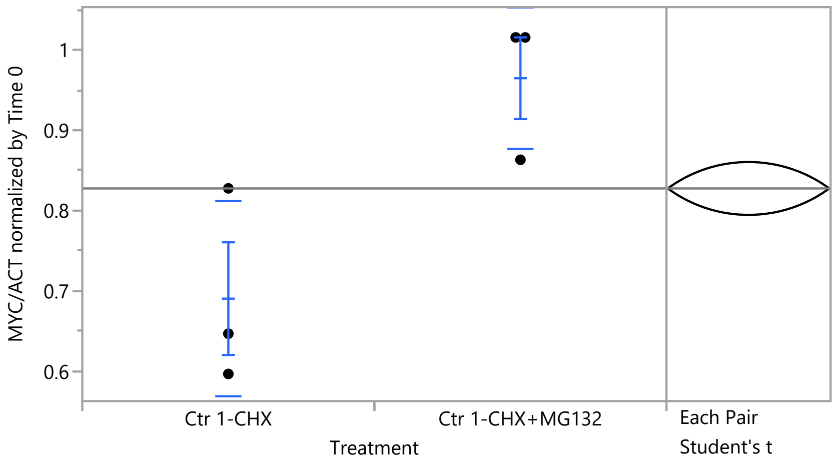

Means and Std Deviations

Level	Number	Mean	Std Dev	Std Err Mean	Lower 95%	Upper 95%
Ctr 1-CHX	3	0.6904332	0.1214635	0.070127	0.3887012	0.9921651
Ctr 1-CHX+MG132	3	0.9647176	0.0880068	0.0508107	0.7460967	1.1833385

Means Comparisons

Comparisons for each pair using Student's t

Confidence Quantile

t	Alpha
2.77645	0.05

LSD Threshold Matrix

Abs(Dif)-LSD

	Ctr 1-CHX+MG132	Ctr 1-CHX
Ctr 1-CHX+MG132	-0.24044	0.03384
Ctr 1-CHX	0.03384	-0.24044

Positive values show pairs of means that are significantly different.

Connecting Letters Report

Level			Mean	Std Error
Ctr 1-CHX+MG132	A		0.96472	0.06124
Ctr 1-CHX		B	0.69043	0.06124

Levels not connected by same letter are significantly different.

Ordered Differences Report

Level	- Level	Difference	Std Err Dif	Lower CL	Upper CL	p-Value
Ctr 1-CHX+MG132	Ctr 1-CHX	0.2742844	0.0865998	0.0338449	0.5147240	0.0339*

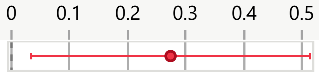

Oneway Analysis of MYC/ACT normalized by Time 0 By Treatment

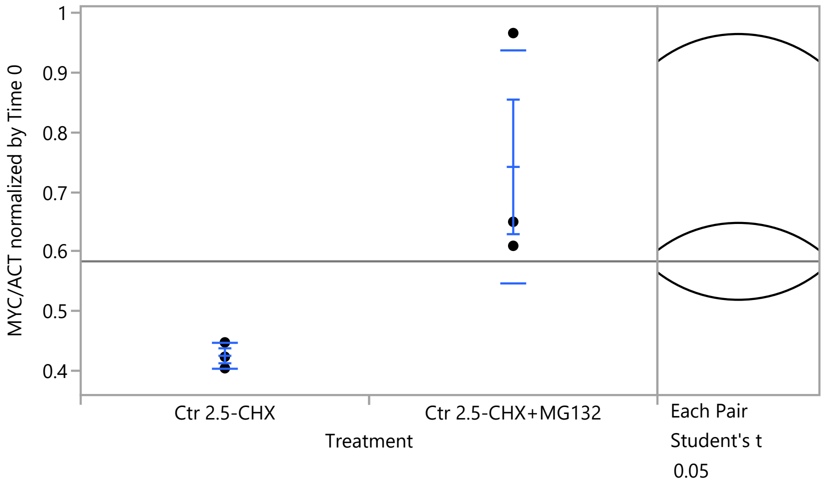

Means and Std Deviations

Level	Number	Mean	Std Dev	Std Err Mean	Lower 95%	Upper 95%
Ctr 2.5-CHX	3	0.4250468	0.0217522	0.0125586	0.3710114	0.4790822
Ctr 2.5-CHX+MG132	3	0.7419081	0.1954488	0.1128424	0.2563864	1.2274298

Means Comparisons

Comparisons for each pair using Student's t

Confidence Quantile

t	Alpha
2.77645	0.05

LSD Threshold Matrix

Abs(Dif)-LSD

	Ctr 2.5-CHX+MG132	Ctr 2.5-CHX
Ctr 2.5-CHX+MG132	-0.31524	0.00163
Ctr 2.5-CHX	0.00163	-0.31524

Positive values show pairs of means that are significantly different.

Connecting Letters Report

Level			Mean	Std Error
Ctr 2.5-CHX+MG132	A		0.74191	0.08028
Ctr 2.5-CHX		B	0.42505	0.08028

Levels not connected by same letter are significantly different.

Ordered Differences Report

Level	- Level	Difference	Std Err Dif	Lower CL	Upper CL	p-Value	
Ctr 2.5-CHX+MG132	Ctr 2.5-CHX	0.3168613	0.1135391	0.0016262	0.6320964	0.0493*	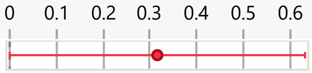

Missing Rows 1

Oneway Analysis of COPT2 By Genotype

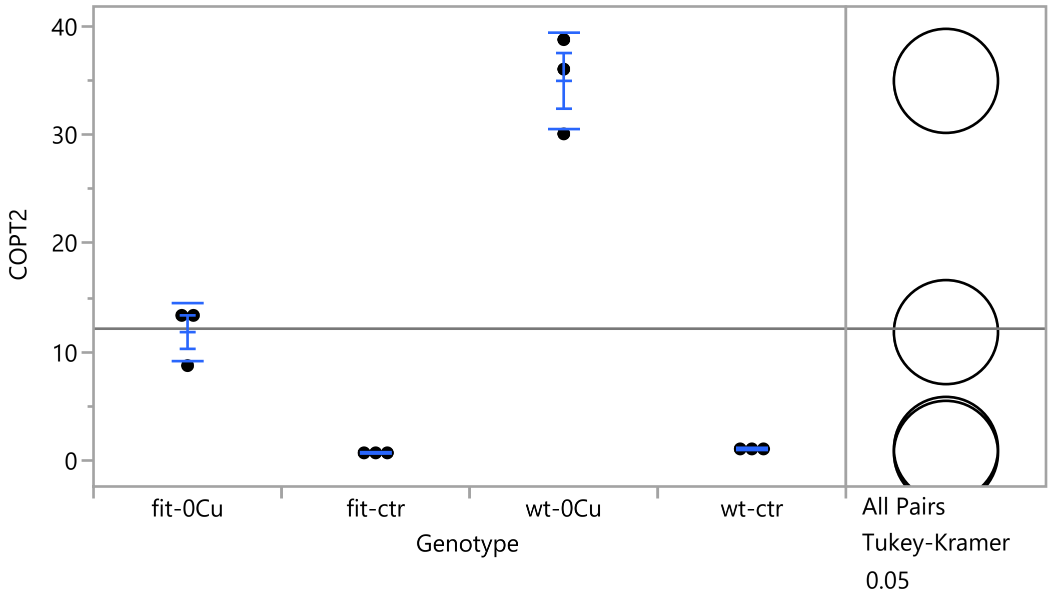

Means and Std Deviations

Level	Number	Mean	Std Dev	Std Err Mean	Lower 95%	Upper 95%
fit-0Cu	3	11.801872	2.6832575	1.5491794	5.1362904	18.467453
fit-ctr	3	0.634775	0.0642903	0.037118	0.475069	0.7944809
wt-0Cu	3	35.040551	4.4587626	2.5742678	23.96437	46.116731
wt-ctr	3	1.0000041	0.1032181	0.059593	0.743596	1.2564121

Oneway Analysis of COPT2 By Genotype

Means Comparisons

Comparisons for all pairs using Tukey-Kramer HSD

Confidence Quantile

q*	Alpha
3.20234	0.05

HSD Threshold Matrix

Abs(Dif)-HSD

	wt-0Cu	fit-0Cu	wt-ctr	fit-ctr
wt-0Cu	-6.805	16.434	27.235	27.601
fit-0Cu	16.434	-6.805	3.997	4.362
wt-ctr	27.235	3.997	-6.805	-6.440
fit-ctr	27.601	4.362	-6.440	-6.805

Positive values show pairs of means that are significantly different.

Connecting Letters Report

Level				Mean	Std Error
wt-0Cu	A			35.041	1.5026
fit-0Cu		B		11.802	1.5026
wt-ctr			C	1.000	1.5026
fit-ctr			C	0.635	1.5026

Levels not connected by same letter are significantly different.

Ordered Differences Report

Level	- Level	Difference	Std Err Dif	Lower CL	Upper CL	p-Value
wt-0Cu	fit-ctr	34.40578	2.125058	27.6006	41.21094	<.0001*
wt-0Cu	wt-ctr	34.04055	2.125058	27.2354	40.84571	<.0001*
wt-0Cu	fit-0Cu	23.23868	2.125058	16.4335	30.04385	<.0001*
fit-0Cu	fit-ctr	11.16710	2.125058	4.3619	17.97226	0.0034*
fit-0Cu	wt-ctr	10.80187	2.125058	3.9967	17.60703	0.0042*
wt-ctr	fit-ctr	0.36523	2.125058	-6.4399	7.17040	0.9980

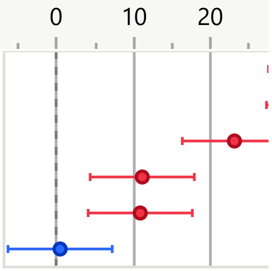

Missing Rows 2

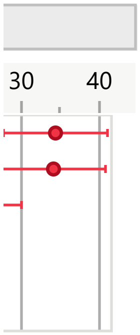
30
40

Oneway Analysis of COPT2 By Genotype

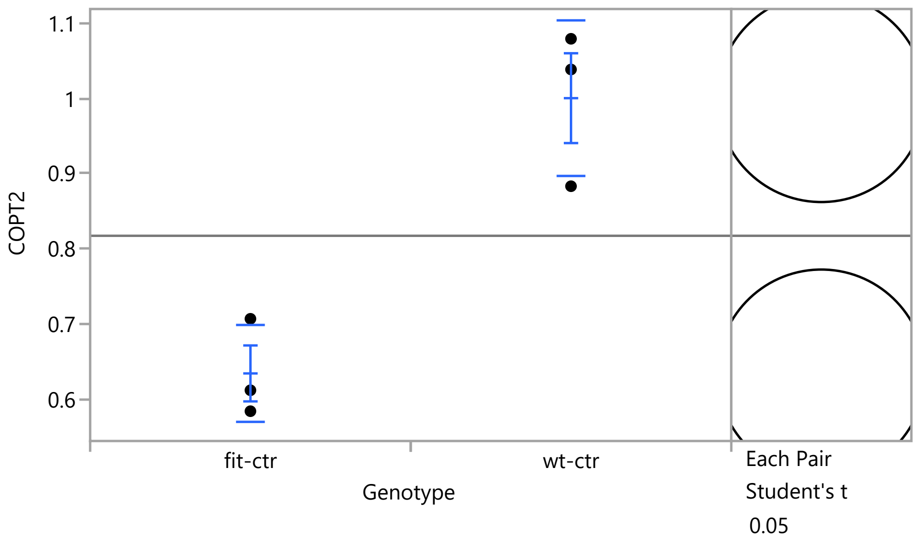

Means and Std Deviations

Level	Number	Mean	Std Dev	Std Err Mean	Lower 95%	Upper 95%
fit-ctr	3	0.634775	0.0642903	0.037118	0.475069	0.7944809
wt-ctr	3	1.0000041	0.1032181	0.059593	0.743596	1.2564121

Means Comparisons

Comparisons for each pair using Student's t

Confidence Quantile

t	Alpha
2.77645	0.05

LSD Threshold Matrix

Abs(Dif)-LSD

	wt-ctr	fit-ctr
wt-ctr	-0.19493	0.17030
fit-ctr	0.17030	-0.19493

Positive values show pairs of means that are significantly different.

Connecting Letters Report

Level			Mean	Std Error
wt-ctr	A		1.0000	0.04964
fit-ctr		B	0.6348	0.04964

Levels not connected by same letter are significantly different.

Ordered Differences Report

Level	- Level	Difference	Std Err Dif	Lower CL	Upper CL	p-Value
wt-ctr	fit-ctr	0.3652291	0.0702074	0.1703022	0.5601560	0.0065*

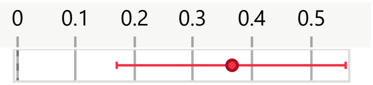

Oneway Analysis of FRO4 By Genotype

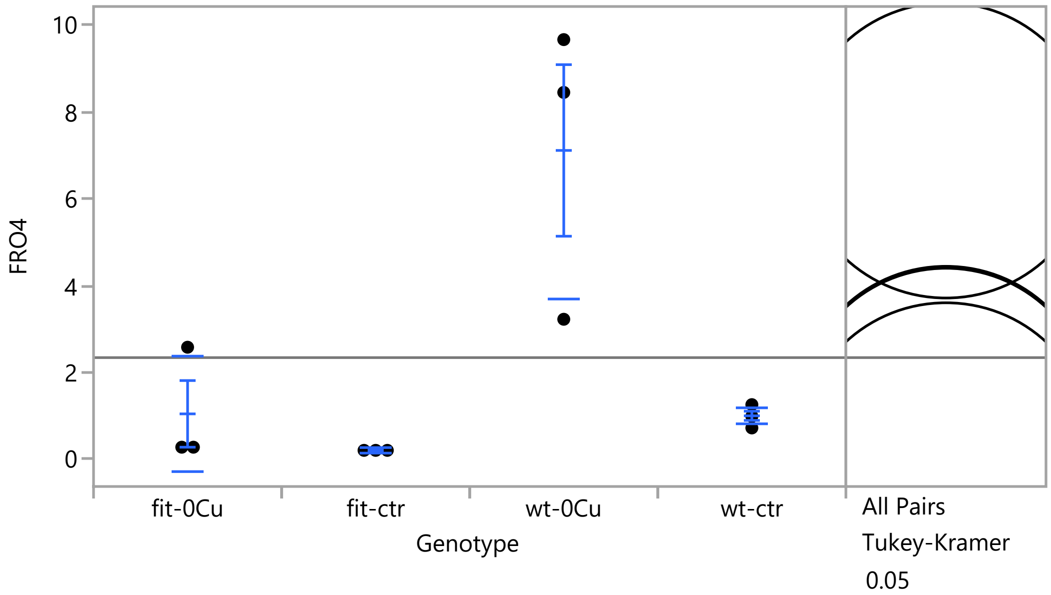

Means and Std Deviations

Level	Number	Mean	Std Dev	Std Err Mean	Lower 95%	Upper 95%
fit-0Cu	3	1.0445497	1.3297422	0.767727	-2.258713	4.3478126
fit-ctr	3	0.2043099	0.063714	0.0367853	0.0460356	0.3625841
wt-0Cu	3	7.1136931	3.4230363	1.9762909	-1.3896	15.616987
wt-ctr	3	0.9999962	0.1842132	0.1063556	0.5423851	1.4576072

Oneway Analysis of FRO4 By Genotype

Means Comparisons

Comparisons for all pairs using Tukey-Kramer HSD

Confidence Quantile

q*	Alpha
3.20234	0.05

HSD Threshold Matrix

Abs(Dif)-HSD

	wt-0Cu	fit-0Cu	wt-ctr	fit-ctr
wt-0Cu	-4.8077	1.2615	1.3060	2.1017
fit-0Cu	1.2615	-4.8077	-4.7631	-3.9674
wt-ctr	1.3060	-4.7631	-4.8077	-4.0120
fit-ctr	2.1017	-3.9674	-4.0120	-4.8077

Positive values show pairs of means that are significantly different.

Connecting Letters Report

Level			Mean	Std Error
wt-0Cu	A		7.1137	1.0616
fit-0Cu		B	1.0445	1.0616
wt-ctr		B	1.0000	1.0616
fit-ctr		B	0.2043	1.0616

Levels not connected by same letter are significantly different.

Ordered Differences Report

Level	- Level	Difference	Std Err Dif	Lower CL	Upper CL	p-Value
wt-0Cu	fit-ctr	6.909383	1.501299	2.10171	11.71706	0.0076*
wt-0Cu	wt-ctr	6.113697	1.501299	1.30602	10.92137	0.0151*
wt-0Cu	fit-0Cu	6.069143	1.501299	1.26147	10.87682	0.0157*
fit-0Cu	fit-ctr	0.840240	1.501299	-3.96744	5.64791	0.9413
wt-ctr	fit-ctr	0.795686	1.501299	-4.01199	5.60336	0.9493
fit-0Cu	wt-ctr	0.044554	1.501299	-4.76312	4.85223	1.0000

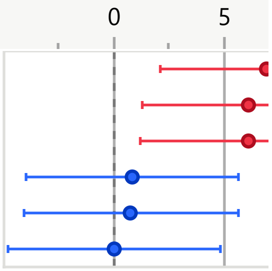

Missing Rows 2

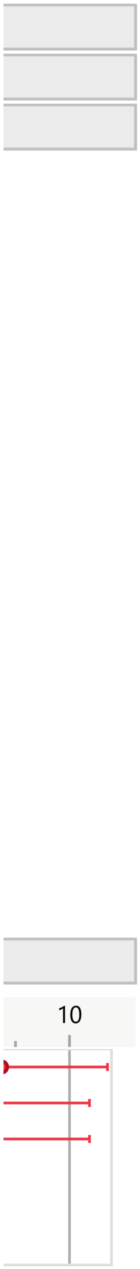
10

Oneway Analysis of FRO4 By Genotype

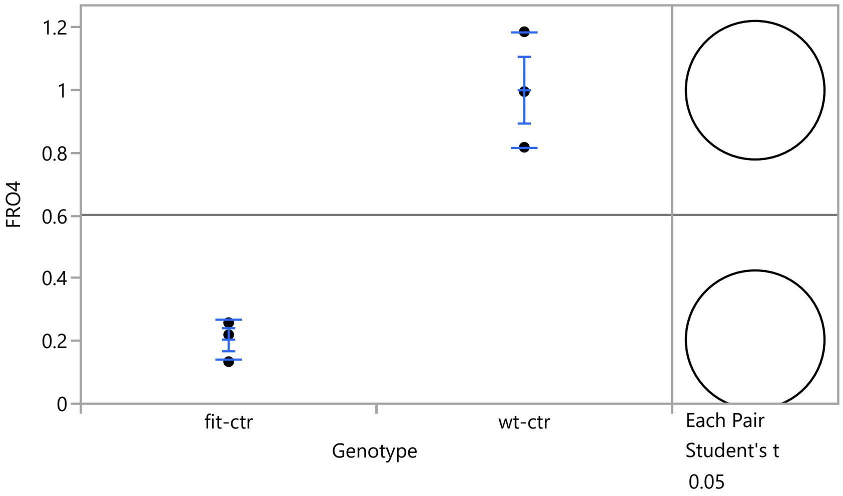

Means and Std Deviations

Level	Number	Mean	Std Dev	Std Err Mean	Lower 95%	Upper 95%
fit-ctr	3	0.2043099	0.063714	0.0367853	0.0460356	0.3625841
wt-ctr	3	0.9999962	0.1842132	0.1063556	0.5423851	1.4576072

Means Comparisons

Comparisons for each pair using Student's t

Confidence Quantile

t	Alpha
2.77645	0.05

LSD Threshold Matrix

Abs(Dif)-LSD

	wt-ctr	fit-ctr
wt-ctr	-0.31245	0.48323
fit-ctr	0.48323	-0.31245

Positive values show pairs of means that are significantly different.

Connecting Letters Report

Level			Mean	Std Error
wt-ctr	A		1.0000	0.07958
fit-ctr		B	0.2043	0.07958

Levels not connected by same letter are significantly different.

Ordered Differences Report

Level	- Level	Difference	Std Err Dif	Lower CL	Upper CL	p-Value
wt-ctr	fit-ctr	0.7956863	0.1125374	0.4832325	1.108140	0.0021*

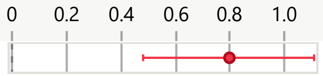

Oneway Analysis of FRO5 By Genotype

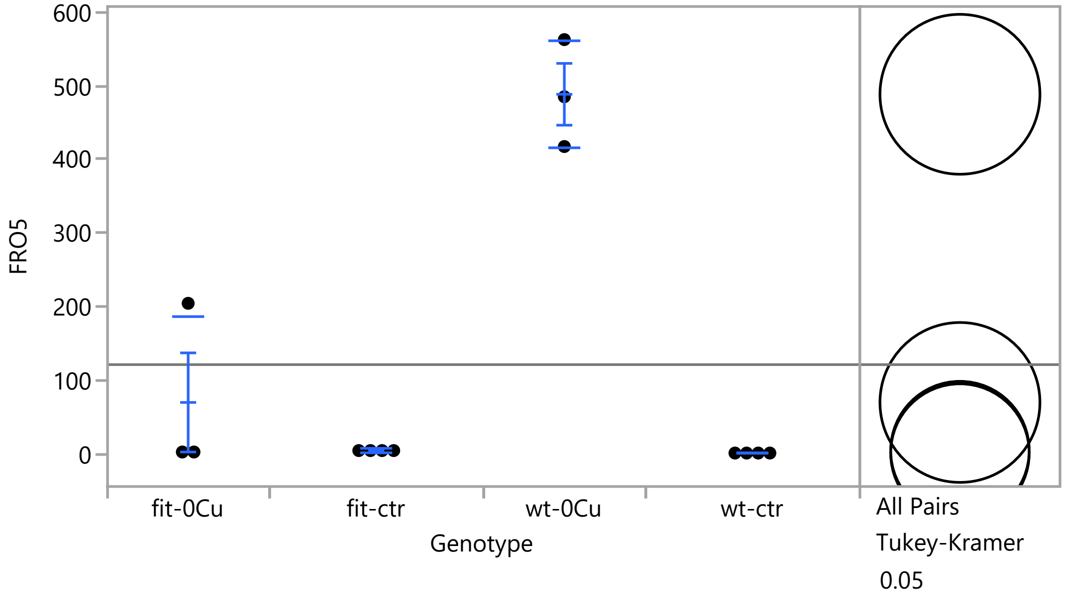

Means and Std Deviations

Level	Number	Mean	Std Dev	Std Err Mean	Lower 95%	Upper 95%
fit-0Cu	3	69.706144	116.56018	67.296053	-219.8454	359.25769
fit-ctr	4	4.3737413	3.0794762	1.5397381	-0.526393	9.2738751
wt-0Cu	3	488.0921	72.645158	41.941702	307.63152	668.55268
wt-ctr	4	1.0001096	0.2784751	0.1392376	0.5569935	1.4432257

Oneway Analysis of FRO5 By Genotype

Means Comparisons

Comparisons for all pairs using Tukey-Kramer HSD

Confidence Quantile

q*	Alpha
3.05935	0.05

HSD Threshold Matrix

Abs(Dif)-HSD

	wt-0Cu	fit-0Cu	fit-ctr	wt-ctr
wt-0Cu	-153.49	264.90	340.14	343.52
fit-0Cu	264.90	-153.49	-78.24	-74.87
fit-ctr	340.14	-78.24	-132.93	-129.55
wt-ctr	343.52	-74.87	-129.55	-132.93

Positive values show pairs of means that are significantly different.

Connecting Letters Report

Level			Mean	Std Error
wt-0Cu	A		488.09	35.476
fit-0Cu		B	69.71	35.476
fit-ctr		B	4.37	30.723
wt-ctr		B	1.00	30.723

Levels not connected by same letter are significantly different.

Ordered Differences Report

Level	- Level	Difference	Std Err Dif	Lower CL	Upper CL	p-Value
wt-0Cu	wt-ctr	487.0920	46.93001	343.517	630.6674	<.0001*
wt-0Cu	fit-ctr	483.7184	46.93001	340.143	627.2938	<.0001*
wt-0Cu	fit-0Cu	418.3860	50.17029	264.897	571.8746	<.0001*
fit-0Cu	wt-ctr	68.7060	46.93001	-74.869	212.2815	0.4917
fit-0Cu	fit-ctr	65.3324	46.93001	-78.243	208.9078	0.5312
fit-ctr	wt-ctr	3.3736	43.44875	-129.551	136.2987	0.9998

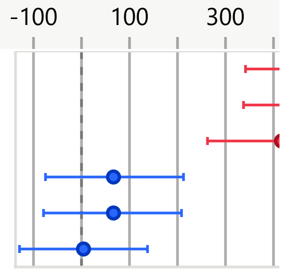

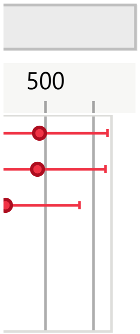
500

Oneway Analysis of COPT2 By Column 1

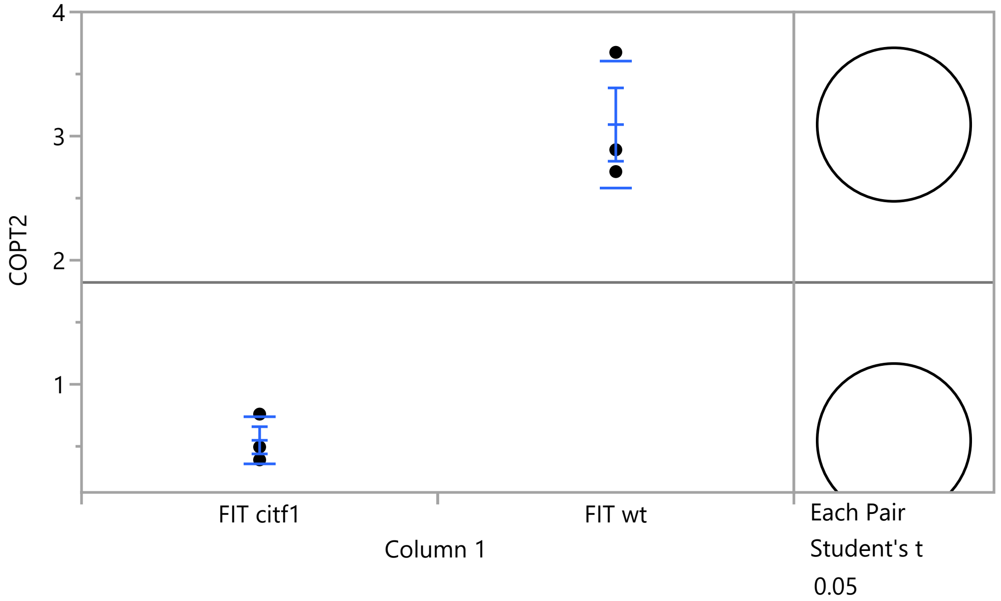

Means and Std Deviations

Level	Number	Mean	Std Dev	Std Err Mean	Lower 95%	Upper 95%
FIT citf1	3	0.5565125	0.1895027	0.1094094	0.0857618	1.0272632
FIT wt	3	3.095026	0.5104049	0.2946824	1.82711	4.3629419

Means Comparisons

Comparisons for each pair using Student's t

Confidence Quantile

t	Alpha
2.77645	0.05

LSD Threshold Matrix

Abs(Dif)-LSD

	FIT wt	FIT citf1
FIT wt	-0.8727	1.6658
FIT citf1	1.6658	-0.8727

Positive values show pairs of means that are significantly different.

Connecting Letters Report

Level			Mean	Std Error
FIT wt	A		3.0950	0.22227
FIT citf1		B	0.5565	0.22227

Levels not connected by same letter are significantly different.

Ordered Differences Report

Level	- Level	Difference	Std Err Dif	Lower CL	Upper CL	p-Value	0 1 2
FIT wt	FIT citf1	2.538513	0.3143376	1.665772	3.411255	0.0013*	

[illegible]

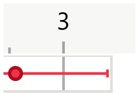
3

Fit Group

Oneway Analysis of FRO4 By Column 1

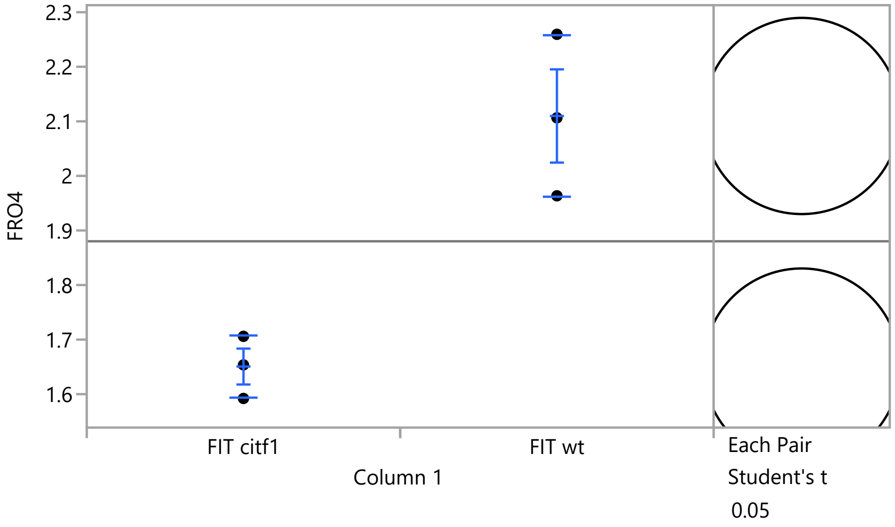

Means and Std Deviations

Level	Number	Mean	Std Dev	Std Err Mean	Lower 95%	Upper 95%
FIT citf1	3	1.6493777	0.057057	0.0329418	1.5076404	1.791115
FIT wt	3	2.1092055	0.1482057	0.0855666	1.7410422	2.4773687

Means Comparisons

Comparisons for each pair using Student's t

Confidence Quantile

t	Alpha
2.77645	0.05

LSD Threshold Matrix

Abs(Dif)-LSD

	FIT wt	FIT citf1
FIT wt	-0.25457	0.20526
FIT citf1	0.20526	-0.25457

Positive values show pairs of means that are significantly different.

Connecting Letters Report

Level			Mean	Std Error
FIT wt	A		2.1092	0.06483
FIT citf1		B	1.6494	0.06483

Levels not connected by same letter are significantly different.

Ordered Differences Report

Level	- Level	Difference	Std Err Dif	Lower CL	Upper CL	p-Value
FIT wt	FIT citf1	0.4598278	0.0916886	0.2052593	0.7143962	0.0074*

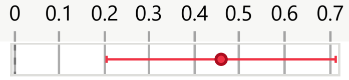

Missing Rows 70

Fit Group

Oneway Analysis of FRO5 By Column 1

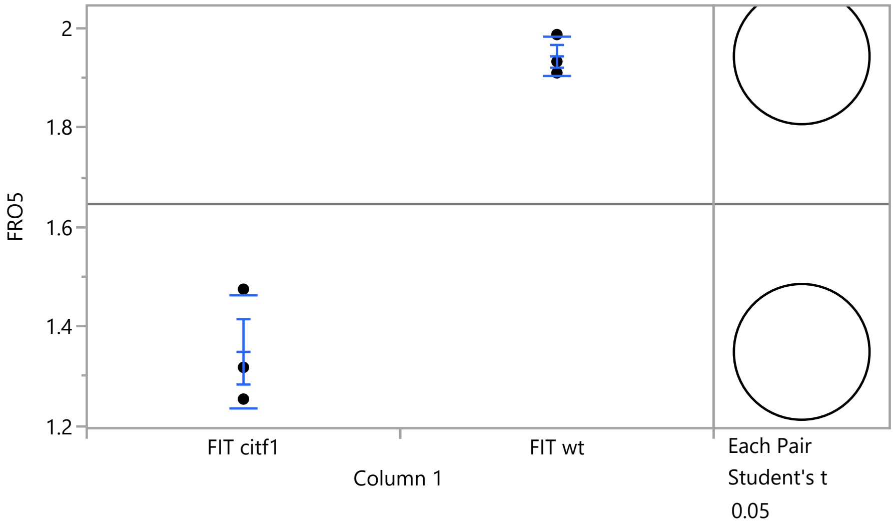

Means and Std Deviations

Level	Number	Mean	Std Dev	Std Err Mean	Lower 95%	Upper 95%
FIT citf1	3	1.348874	0.1137131	0.0656523	1.0663949	1.6313531
FIT wt	3	1.9431222	0.0395443	0.0228309	1.8448887	2.0413557

Means Comparisons

Comparisons for each pair using Student's t

Confidence Quantile

t	Alpha
2.77645	0.05

LSD Threshold Matrix

Abs(Dif)-LSD

	FIT wt	FIT citf1
FIT wt	-0.19299	0.40126
FIT citf1	0.40126	-0.19299

Positive values show pairs of means that are significantly different.

Connecting Letters Report

Level			Mean	Std Error
FIT wt	A		1.9431	0.04915
FIT citf1		B	1.3489	0.04915

Levels not connected by same letter are significantly different.

Ordered Differences Report

Level	- Level	Difference	Std Err Dif	Lower CL	Upper CL	p-Value	
FIT wt	FIT citf1	0.5942482	0.0695088	0.4012608	0.7872356	0.0010*	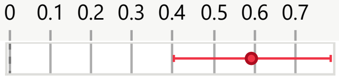

Missing Rows 70

Oneway Analysis of COPT2 RE By Sample

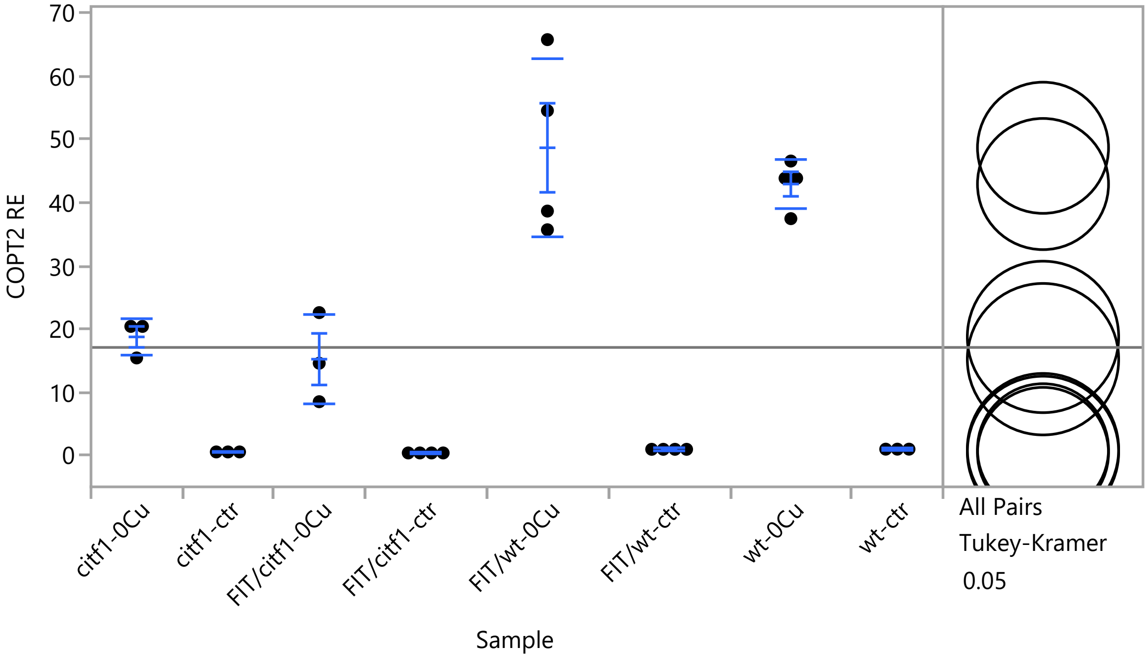

Means and Std Deviations

Level	Number	Mean	Std Dev	Std Err Mean	Lower 95%	Upper 95%
citf1-0Cu	3	18.766874	2.8827378	1.6643495	11.605756	25.927992
citf1-ctr	3	0.5616733	0.0746174	0.0430804	0.3763134	0.7470331
FIT/citf1-0Cu	3	15.242594	7.0727515	4.083455	-2.327095	32.812283
FIT/citf1-ctr	4	0.4015154	0.1419158	0.0709579	0.1756956	0.6273351
FIT/wt-0Cu	4	48.704862	14.098394	7.0491972	26.271171	71.138554
FIT/wt-ctr	4	0.9771562	0.2466122	0.1233061	0.5847411	1.3695712
wt-0Cu	4	42.964043	3.8852394	1.9426197	36.78176	49.146326
wt-ctr	3	1.0000113	0.1972539	0.1138846	0.5100055	1.4900172

Means Comparisons

Comparisons for all pairs using Tukey-Kramer HSD

Confidence Quantile

q*	Alpha
3.37119	0.05

Oneway Analysis of COPT2 RE By Sample

Means Comparisons

Comparisons for all pairs using Tukey-Kramer HSD

HSD Threshold Matrix

Abs(Dif)-HSD

	FIT/wt-0Cu	wt-0Cu	citf1-0Cu	FIT/citf1-0Cu	wt-ctr	FIT/wt-ctr	citf1-ctr	FIT/citf1-ctr
FIT/wt-0Cu	-14.681	-8.940	14.081	17.605	31.848	33.047	32.286	33.622
wt-0Cu	-8.940	-14.681	8.340	11.864	26.107	27.306	26.545	27.882
citf1-0Cu	14.081	8.340	-16.952	-13.428	0.815	1.932	1.253	2.508
FIT/citf1-0Cu	17.605	11.864	-13.428	-16.952	-2.710	-1.592	-2.271	-1.016
wt-ctr	31.848	26.107	0.815	-2.710	-16.952	-15.834	-16.514	-15.259
FIT/wt-ctr	33.047	27.306	1.932	-1.592	-15.834	-14.681	-15.442	-14.105
citf1-ctr	32.286	26.545	1.253	-2.271	-16.514	-15.442	-16.952	-15.697
FIT/citf1-ctr	33.622	27.882	2.508	-1.016	-15.259	-14.105	-15.697	-14.681

Positive values show pairs of means that are significantly different.

Connecting Letters Report

Level				Mean	Std Error
FIT/wt-0Cu	A			48.705	3.0793
wt-0Cu	A			42.964	3.0793
citf1-0Cu		B		18.767	3.5557
FIT/citf1-0Cu		B	C	15.243	3.5557
wt-ctr			C	1.000	3.5557
FIT/wt-ctr			C	0.977	3.0793
citf1-ctr			C	0.562	3.5557
FIT/citf1-ctr			C	0.402	3.0793

Levels not connected by same letter are significantly different.

Oneway Analysis of COPT2 RE By Sample

Means Comparisons

Comparisons for all pairs using Tukey-Kramer HSD

Ordered Differences Report

Level	- Level	Difference	Std Err Dif	Lower CL	Upper CL	p-Value
FIT/wt-0Cu	FIT/citf1-ctr	48.30335	4.354823	33.6224	62.98429	<.0001*
FIT/wt-0Cu	citf1-ctr	48.14319	4.703746	32.2860	64.00042	<.0001*
FIT/wt-0Cu	FIT/wt-ctr	47.72771	4.354823	33.0468	62.40865	<.0001*
FIT/wt-0Cu	wt-ctr	47.70485	4.703746	31.8476	63.56208	<.0001*
wt-0Cu	FIT/citf1-ctr	42.56253	4.354823	27.8816	57.24347	<.0001*
wt-0Cu	citf1-ctr	42.40237	4.703746	26.5451	58.25960	<.0001*
wt-0Cu	FIT/wt-ctr	41.98689	4.354823	27.3059	56.66783	<.0001*
wt-0Cu	wt-ctr	41.96403	4.703746	26.1068	57.82126	<.0001*
FIT/wt-0Cu	FIT/citf1-0Cu	33.46227	4.703746	17.6050	49.31950	<.0001*
FIT/wt-0Cu	citf1-0Cu	29.93799	4.703746	14.0808	45.79522	<.0001*
wt-0Cu	FIT/citf1-0Cu	27.72145	4.703746	11.8642	43.57868	0.0002*
wt-0Cu	citf1-0Cu	24.19717	4.703746	8.3399	40.05440	0.0011*
citf1-0Cu	FIT/citf1-ctr	18.36536	4.703746	2.5081	34.22259	0.0163*
citf1-0Cu	citf1-ctr	18.20520	5.028516	1.2531	35.15730	0.0298*
citf1-0Cu	FIT/wt-ctr	17.78972	4.703746	1.9325	33.64695	0.0212*
citf1-0Cu	wt-ctr	17.76686	5.028516	0.8148	34.71896	0.0358*
FIT/citf1-0Cu	FIT/citf1-ctr	14.84108	4.703746	-1.0162	30.69831	0.0770
FIT/citf1-0Cu	citf1-ctr	14.68092	5.028516	-2.2712	31.63302	0.1209
FIT/citf1-0Cu	FIT/wt-ctr	14.26544	4.703746	-1.5918	30.12267	0.0976
FIT/citf1-0Cu	wt-ctr	14.24258	5.028516	-2.7095	31.19468	0.1418
FIT/wt-0Cu	wt-0Cu	5.74082	4.354823	-8.9401	20.42176	0.8814
citf1-0Cu	FIT/citf1-0Cu	3.52428	5.028516	-13.4278	20.47637	0.9960
wt-ctr	FIT/citf1-ctr	0.59850	4.703746	-15.2587	16.45573	1.0000
FIT/wt-ctr	FIT/citf1-ctr	0.57564	4.354823	-14.1053	15.25659	1.0000
wt-ctr	citf1-ctr	0.43834	5.028516	-16.5138	17.39043	1.0000
FIT/wt-ctr	citf1-ctr	0.41548	4.703746	-15.4417	16.27272	1.0000
citf1-ctr	FIT/citf1-ctr	0.16016	4.703746	-15.6971	16.01739	1.0000
wt-ctr	FIT/wt-ctr	0.02286	4.703746	-15.8344	15.88009	1.0000

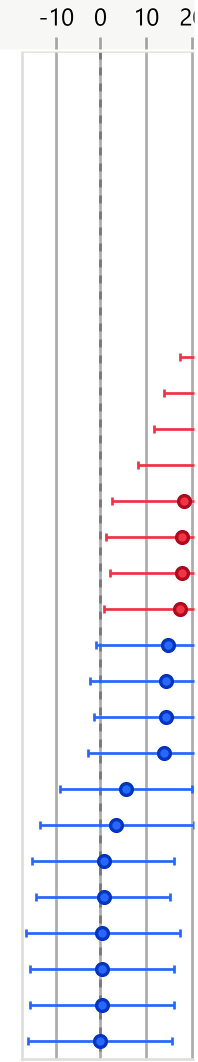

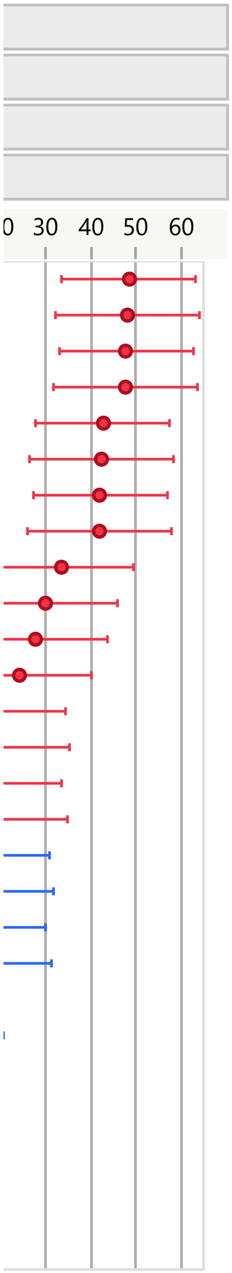
0
30
40
50
60

Oneway Analysis of COPT2 RE By Sample

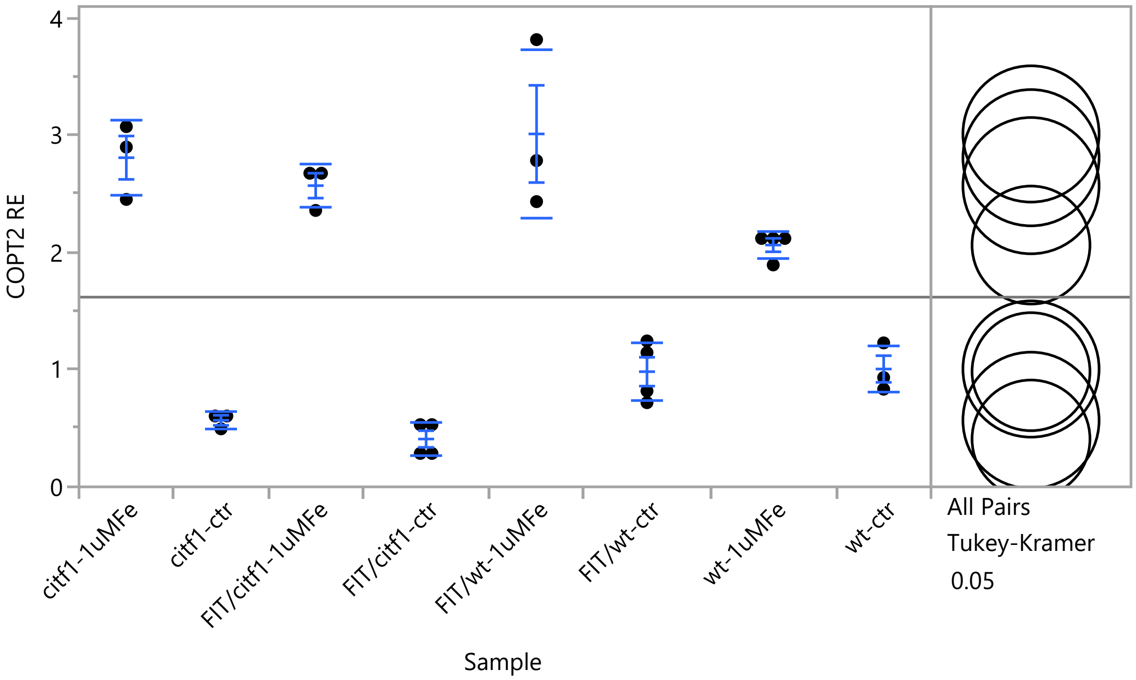

Means and Std Deviations

Level	Number	Mean	Std Dev	Std Err Mean	Lower 95%	Upper 95%
citf1-1uMFe	3	2.8060643	0.320832	0.1852324	2.0090735	3.6030552
citf1-ctr	3	0.5616733	0.0746174	0.0430804	0.3763134	0.7470331
FIT/citf1-1uMFe	3	2.5672517	0.1843715	0.1064469	2.1092476	3.0252558
FIT/citf1-ctr	4	0.4015154	0.1419158	0.0709579	0.1756956	0.6273351
FIT/wt-1uMFe	3	3.0091842	0.7197714	0.4155602	1.221173	4.7971954
FIT/wt-ctr	4	0.9771562	0.2466122	0.1233061	0.5847411	1.3695712
wt-1uMFe	4	2.0595296	0.1153028	0.0576514	1.8760571	2.2430021
wt-ctr	3	1.0000113	0.1972539	0.1138846	0.5100055	1.4900172

Means Comparisons

Comparisons for all pairs using Tukey-Kramer HSD

Confidence Quantile

q*	Alpha
3.39014	0.05

Oneway Analysis of COPT2 RE By Sample

Means Comparisons

Comparisons for all pairs using Tukey-Kramer HSD

HSD Threshold Matrix

Abs(Dif)-HSD

	FIT/wt-1uMFe	citf1-1uMFe	FIT/citf1-1uMFe	wt-1uMFe	wt-ctr	FIT/wt-ctr	citf1-ctr	FIT/citf[illegible]
FIT/wt-1uMFe	-0.8235	-0.6204	-0.3816	0.1793	1.1857	1.2617	1.6240	1.8[illegible]
citf1-1uMFe	-0.6204	-0.8235	-0.5847	-0.0238	0.9825	1.0586	1.4209	1.6[illegible]
FIT/citf1-1uMFe	-0.3816	-0.5847	-0.8235	-0.2626	0.7437	0.8198	1.1821	1.3[illegible]
wt-1uMFe	0.1793	-0.0238	-0.2626	-0.7132	0.2892	0.3692	0.7275	0.9[illegible]
wt-ctr	1.1857	0.9825	0.7437	0.2892	-0.8235	-0.7475	-0.3852	-0.1[illegible]
FIT/wt-ctr	1.2617	1.0586	0.8198	0.3692	-0.7475	-0.7132	-0.3548	-0.1[illegible]
citf1-ctr	1.6240	1.4209	1.1821	0.7275	-0.3852	-0.3548	-0.8235	-0.6[illegible]
FIT/citf1-ctr	1.8373	1.6342	1.3954	0.9448	-0.1718	-0.1375	-0.6102	-0.7[illegible]

Positive values show pairs of means that are significantly different.

Connecting Letters Report

Level				Mean	Std Error
FIT/wt-1uMFe	A			3.0092	0.17177
citf1-1uMFe	A	B		2.8061	0.17177
FIT/citf1-1uMFe	A	B		2.5673	0.17177
wt-1uMFe		B		2.0595	0.14875
wt-ctr			C	1.0000	0.17177
FIT/wt-ctr			C	0.9772	0.14875
citf1-ctr			C	0.5617	0.17177
FIT/citf1-ctr			C	0.4015	0.14875

Levels not connected by same letter are significantly different.

[illegible]1-ctr
[illegible]373
[illegible]342
[illegible]954
[illegible]448
[illegible]718
[illegible]375
[illegible]102
[illegible]132

Oneway Analysis of COPT2 RE By Sample

Means Comparisons

Comparisons for all pairs using Tukey-Kramer HSD

Ordered Differences Report

Level	- Level	Difference	Std Err Dif	Lower CL	Upper CL	p-Value
FIT/wt-1uMFe	FIT/citf1-ctr	2.607669	0.2272252	1.83734	3.377993	<.0001*
FIT/wt-1uMFe	citf1-ctr	2.447511	0.2429139	1.62400	3.271022	<.0001*
citf1-1uMFe	FIT/citf1-ctr	2.404549	0.2272252	1.63422	3.174873	<.0001*
citf1-1uMFe	citf1-ctr	2.244391	0.2429139	1.42088	3.067902	<.0001*
FIT/citf1-1uMFe	FIT/citf1-ctr	2.165736	0.2272252	1.39541	2.936060	<.0001*
FIT/wt-1uMFe	FIT/wt-ctr	2.032028	0.2272252	1.26170	2.802352	<.0001*
FIT/wt-1uMFe	wt-ctr	2.009173	0.2429139	1.18566	2.832684	<.0001*
FIT/citf1-1uMFe	citf1-ctr	2.005578	0.2429139	1.18207	2.829090	<.0001*
citf1-1uMFe	FIT/wt-ctr	1.828908	0.2272252	1.05858	2.599232	<.0001*
citf1-1uMFe	wt-ctr	1.806053	0.2429139	0.98254	2.629564	<.0001*
wt-1uMFe	FIT/citf1-ctr	1.658014	0.2103696	0.94483	2.371196	<.0001*
FIT/citf1-1uMFe	FIT/wt-ctr	1.590096	0.2272252	0.81977	2.360420	<.0001*
FIT/citf1-1uMFe	wt-ctr	1.567240	0.2429139	0.74373	2.390751	<.0001*
wt-1uMFe	citf1-ctr	1.497856	0.2272252	0.72753	2.268180	<.0001*
wt-1uMFe	FIT/wt-ctr	1.082373	0.2103696	0.36919	1.795555	0.0012*
wt-1uMFe	wt-ctr	1.059518	0.2272252	0.28919	1.829842	0.0034*
FIT/wt-1uMFe	wt-1uMFe	0.949655	0.2272252	0.17933	1.719979	0.0097*
citf1-1uMFe	wt-1uMFe	0.746535	0.2272252	-0.02379	1.516859	0.0616
wt-ctr	FIT/citf1-ctr	0.598496	0.2272252	-0.17183	1.368820	0.2036
FIT/wt-ctr	FIT/citf1-ctr	0.575641	0.2103696	-0.13754	1.288822	0.1710
FIT/citf1-1uMFe	wt-1uMFe	0.507722	0.2272252	-0.26260	1.278046	0.3756
FIT/wt-1uMFe	FIT/citf1-1uMFe	0.441933	0.2429139	-0.38158	1.265444	0.6158
wt-ctr	citf1-ctr	0.438338	0.2429139	-0.38517	1.261849	0.6247
FIT/wt-ctr	citf1-ctr	0.415483	0.2272252	-0.35484	1.185807	0.6102
citf1-1uMFe	FIT/citf1-1uMFe	0.238813	0.2429139	-0.58470	1.062324	0.9715
FIT/wt-1uMFe	citf1-1uMFe	0.203120	0.2429139	-0.62039	1.026631	0.9884
citf1-ctr	FIT/citf1-ctr	0.160158	0.2272252	-0.61017	0.930482	0.9958
wt-ctr	FIT/wt-ctr	0.022855	0.2272252	-0.74747	0.793179	1.0000

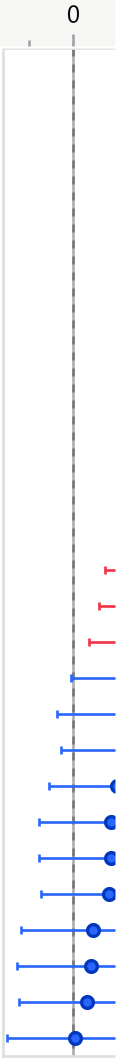

Missing Rows 10

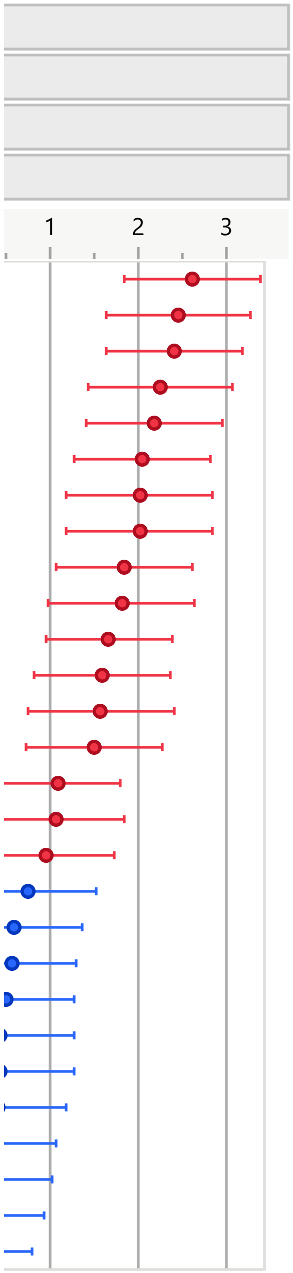
1
2
3

Oneway Analysis of COPT2 By Sample

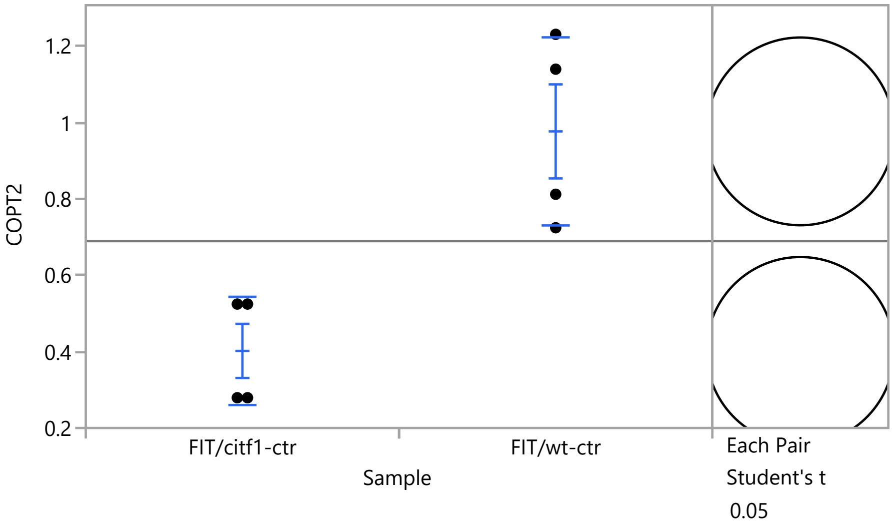

Means and Std Deviations

Level	Number	Mean	Std Dev	Std Err Mean	Lower 95%	Upper 95%
FIT/citf1-ctr	4	0.4015154	0.1419158	0.0709579	0.1756956	0.6273351
FIT/wt-ctr	4	0.9771562	0.2466122	0.1233061	0.5847411	1.3695712

Means Comparisons

Comparisons for each pair using Student's t

Confidence Quantile

t	Alpha
2.44691	0.05

LSD Threshold Matrix

Abs(Dif)-LSD

	FIT/wt-ctr	FIT/citf1-ctr
FIT/wt-ctr	-0.34811	0.22753
FIT/citf1-ctr	0.22753	-0.34811

Positive values show pairs of means that are significantly different.

Connecting Letters Report

Level			Mean	Std Error
FIT/wt-ctr	A		0.97716	0.10060
FIT/citf1-ctr		B	0.40152	0.10060

Levels not connected by same letter are significantly different.

Ordered Differences Report

Level	- Level	Difference	Std Err Dif	Lower CL	Upper CL	p-Value	
FIT/wt-ctr	FIT/citf1-ctr	0.5756408	0.1422653	0.2275301	0.9237515	0.0068*	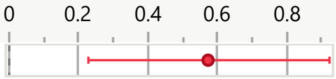

Oneway Analysis of COPT2 By Sample

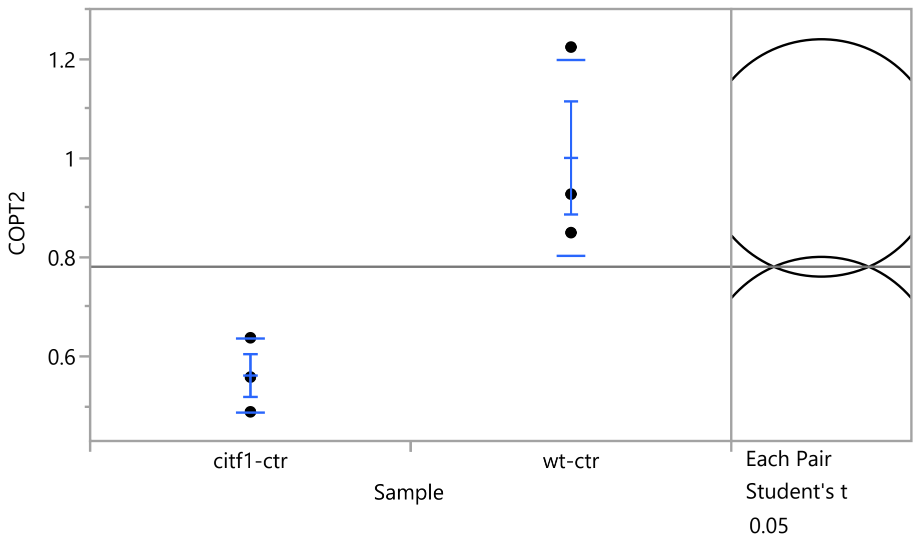

Means and Std Deviations

Level	Number	Mean	Std Dev	Std Err Mean	Lower 95%	Upper 95%
citf1-ctr	3	0.5616733	0.0746174	0.0430804	0.3763134	0.7470331
wt-ctr	3	1.0000113	0.1972539	0.1138846	0.5100055	1.4900172

Means Comparisons

Comparisons for each pair using Student's t

Confidence Quantile

t	Alpha
2.77645	0.05

LSD Threshold Matrix

Abs(Dif)-LSD

	wt-ctr	citf1-ctr
wt-ctr	-0.33806	0.10028
citf1-ctr	0.10028	-0.33806

Positive values show pairs of means that are significantly different.

Connecting Letters Report

Level			Mean	Std Error
wt-ctr	A		1.0000	0.08610
citf1-ctr		B	0.5617	0.08610

Levels not connected by same letter are significantly different.

Ordered Differences Report

Level	- Level	Difference	Std Err Dif	Lower CL	Upper CL	p-Value
wt-ctr	citf1-ctr	0.4383381	0.1217605	0.1002768	0.7763994	0.0228*

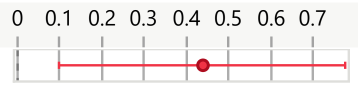

Oneway Analysis of COPT2 By Sample

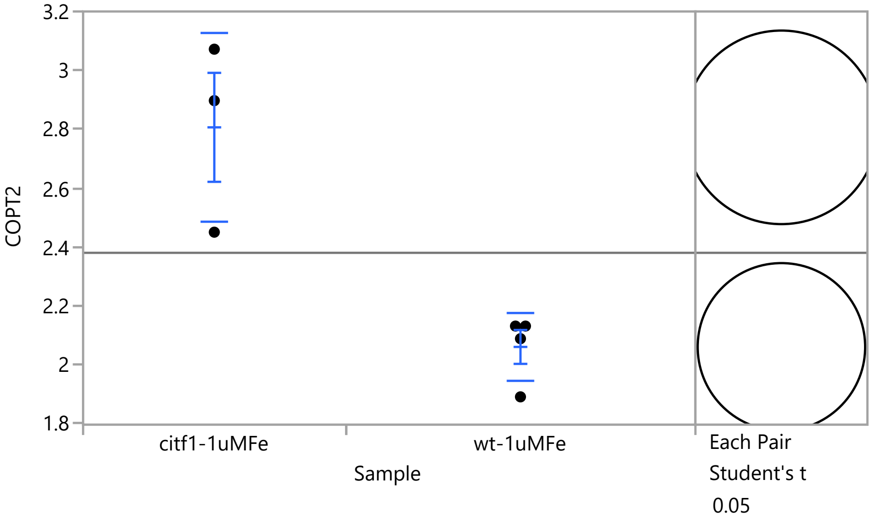

Means and Std Deviations

Level	Number	Mean	Std Dev	Std Err Mean	Lower 95%	Upper 95%
citf1-1uMFe	3	2.8060643	0.320832	0.1852324	2.0090735	3.6030552
wt-1uMFe	4	2.0595296	0.1153028	0.0576514	1.8760571	2.2430021

Means Comparisons

Comparisons for each pair using Student's t

Confidence Quantile

t	Alpha
2.57058	0.05

LSD Threshold Matrix

Abs(Dif)-LSD

	citf1-1uMFe	wt-1uMFe
citf1-1uMFe	-0.46532	0.31127
wt-1uMFe	0.31127	-0.40298

Positive values show pairs of means that are significantly different.

Connecting Letters Report

Level			Mean	Std Error
citf1-1uMFe	A		2.8061	0.12800
wt-1uMFe		B	2.0595	0.11085

Levels not connected by same letter are significantly different.

Ordered Differences Report

Level	- Level	Difference	Std Err Dif	Lower CL	Upper CL	p-Value
citf1-1uMFe	wt-1uMFe	0.7465347	0.1693248	0.3112714	1.181798	0.0070*

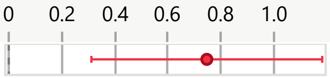

Oneway Analysis of FRO4 RE By Sample

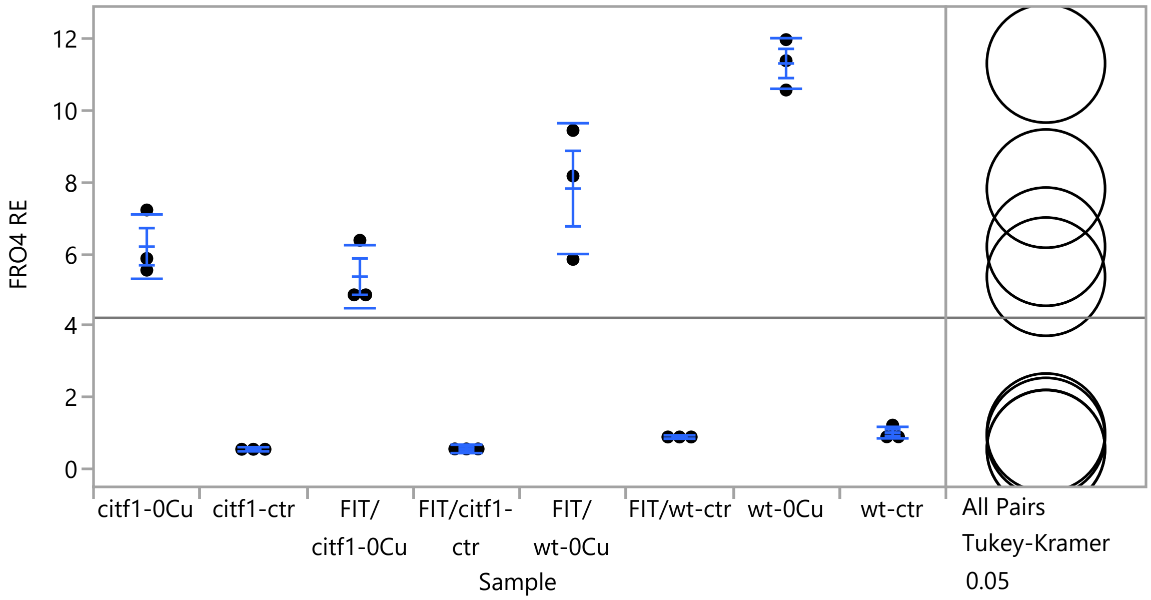

Means and Std Deviations

Level	Number	Mean	Std Dev	Std Err Mean	Lower 95%	Upper 95%
citf1-0Cu	3	6.1989435	0.8993551	0.5192429	3.9648215	8.4330654
citf1-ctr	3	0.5345854	0.0580616	0.0335219	0.3903524	0.6788185
FIT/citf1-0Cu	3	5.360222	0.8809533	0.5086186	3.1718127	7.5486314
FIT/citf1-ctr	3	0.5440741	0.1176857	0.0679459	0.2517265	0.8364217
FIT/wt-0Cu	3	7.8223256	1.8283551	1.0556013	3.2804396	12.364212
FIT/wt-ctr	3	0.8779637	0.0475898	0.027476	0.759744	0.9961834
wt-0Cu	3	11.320822	0.7074832	0.4084656	9.5633365	13.078308
wt-ctr	3	0.9999955	0.1603717	0.0925906	0.6016101	1.3983809

Means Comparisons

Comparisons for all pairs using Tukey-Kramer HSD

Confidence Quantile

q*	Alpha
3.46215	0.05

HSD Threshold Matrix

Abs(Dif)-HSD

	wt-0Cu	FIT/wt-0Cu	citf1-0Cu	FIT/citf1-0Cu	wt-ctr	FIT/wt-ctr	FIT/citf1-ctr	citf1-ctr
wt-0Cu	-2.3382	1.1603	2.7836	3.6224	7.9826	8.1046	8.4385	8.4480
FIT/wt-0Cu	1.1603	-2.3382	-0.7149	0.1239	4.4841	4.6061	4.9400	4.9495
citf1-0Cu	2.7836	-0.7149	-2.3382	-1.4995	2.8607	2.9827	3.3166	3.3261
FIT/citf1-0Cu	3.6224	0.1239	-1.4995	-2.3382	2.0220	2.1440	2.4779	2.4874
wt-ctr	7.9826	4.4841	2.8607	2.0220	-2.3382	-2.2162	-1.8823	-1.8728
FIT/wt-ctr	8.1046	4.6061	2.9827	2.1440	-2.2162	-2.3382	-2.0044	-1.9949
FIT/citf1-ctr	8.4385	4.9400	3.3166	2.4779	-1.8823	-2.0044	-2.3382	-2.3288
citf1-ctr	8.4480	4.9495	3.3261	2.4874	-1.8728	-1.9949	-2.3288	-2.3382

Positive values show pairs of means that are significantly different.

Oneway Analysis of FRO4 RE By Sample

Means Comparisons

Comparisons for all pairs using Tukey-Kramer HSD

Connecting Letters Report

Level					Mean	Std Error
wt-0Cu	A				11.321	0.47756
FIT/wt-0Cu		B			7.822	0.47756
citf1-0Cu		B	C		6.199	0.47756
FIT/citf1-0Cu			C		5.360	0.47756
wt-ctr				D	1.000	0.47756
FIT/wt-ctr				D	0.878	0.47756
FIT/citf1-ctr				D	0.544	0.47756
citf1-ctr				D	0.535	0.47756

Levels not connected by same letter are significantly different.

Ordered Differences Report

Level	- Level	Difference	Std Err Dif	Lower CL	Upper CL	p-Value
wt-0Cu	citf1-ctr	10.78624	0.6753726	8.44799	13.12448	<.0001*
wt-0Cu	FIT/citf1-ctr	10.77675	0.6753726	8.43851	13.11499	<.0001*
wt-0Cu	FIT/wt-ctr	10.44286	0.6753726	8.10462	12.78110	<.0001*
wt-0Cu	wt-ctr	10.32083	0.6753726	7.98258	12.65907	<.0001*
FIT/wt-0Cu	citf1-ctr	7.28774	0.6753726	4.94950	9.62598	<.0001*
FIT/wt-0Cu	FIT/citf1-ctr	7.27825	0.6753726	4.94001	9.61649	<.0001*
FIT/wt-0Cu	FIT/wt-ctr	6.94436	0.6753726	4.60612	9.28260	<.0001*
FIT/wt-0Cu	wt-ctr	6.82233	0.6753726	4.48409	9.16057	<.0001*
wt-0Cu	FIT/citf1-0Cu	5.96060	0.6753726	3.62236	8.29884	<.0001*
citf1-0Cu	citf1-ctr	5.66436	0.6753726	3.32612	8.00260	<.0001*
citf1-0Cu	FIT/citf1-ctr	5.65487	0.6753726	3.31663	7.99311	<.0001*
citf1-0Cu	FIT/wt-ctr	5.32098	0.6753726	2.98274	7.65922	<.0001*
citf1-0Cu	wt-ctr	5.19895	0.6753726	2.86071	7.53719	<.0001*
wt-0Cu	citf1-0Cu	5.12188	0.6753726	2.78364	7.46012	<.0001*
FIT/citf1-0Cu	citf1-ctr	4.82564	0.6753726	2.48739	7.16388	<.0001*
FIT/citf1-0Cu	FIT/citf1-ctr	4.81615	0.6753726	2.47791	7.15439	<.0001*
FIT/citf1-0Cu	FIT/wt-ctr	4.48226	0.6753726	2.14402	6.82050	0.0001*
FIT/citf1-0Cu	wt-ctr	4.36023	0.6753726	2.02198	6.69847	0.0002*
wt-0Cu	FIT/wt-0Cu	3.49850	0.6753726	1.16025	5.83674	0.0018*
FIT/wt-0Cu	FIT/citf1-0Cu	2.46210	0.6753726	0.12386	4.80035	0.0354*
FIT/wt-0Cu	citf1-0Cu	1.62338	0.6753726	-0.71486	3.96162	0.3025
citf1-0Cu	FIT/citf1-0Cu	0.83872	0.6753726	-1.49952	3.17696	0.9070
wt-ctr	citf1-ctr	0.46541	0.6753726	-1.87283	2.80365	0.9962
wt-ctr	FIT/citf1-ctr	0.45592	0.6753726	-1.88232	2.79416	0.9966
FIT/wt-ctr	citf1-ctr	0.34338	0.6753726	-1.99486	2.68162	0.9994
FIT/wt-ctr	FIT/citf1-ctr	0.33389	0.6753726	-2.00435	2.67213	0.9995
wt-ctr	FIT/wt-ctr	0.12203	0.6753726	-2.21621	2.46027	1.0000
FIT/citf1-ctr	citf1-ctr	0.00949	0.6753726	-2.32875	2.34773	1.0000

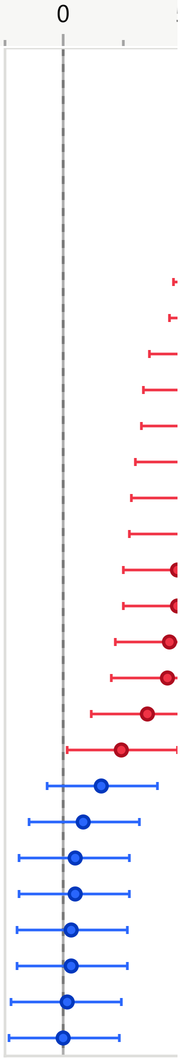

Missing Rows 17

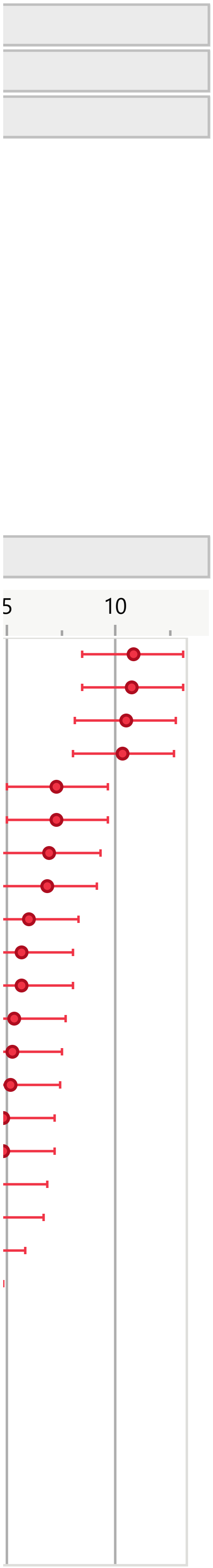
5
10

Oneway Analysis of FRO4 RE By Sample

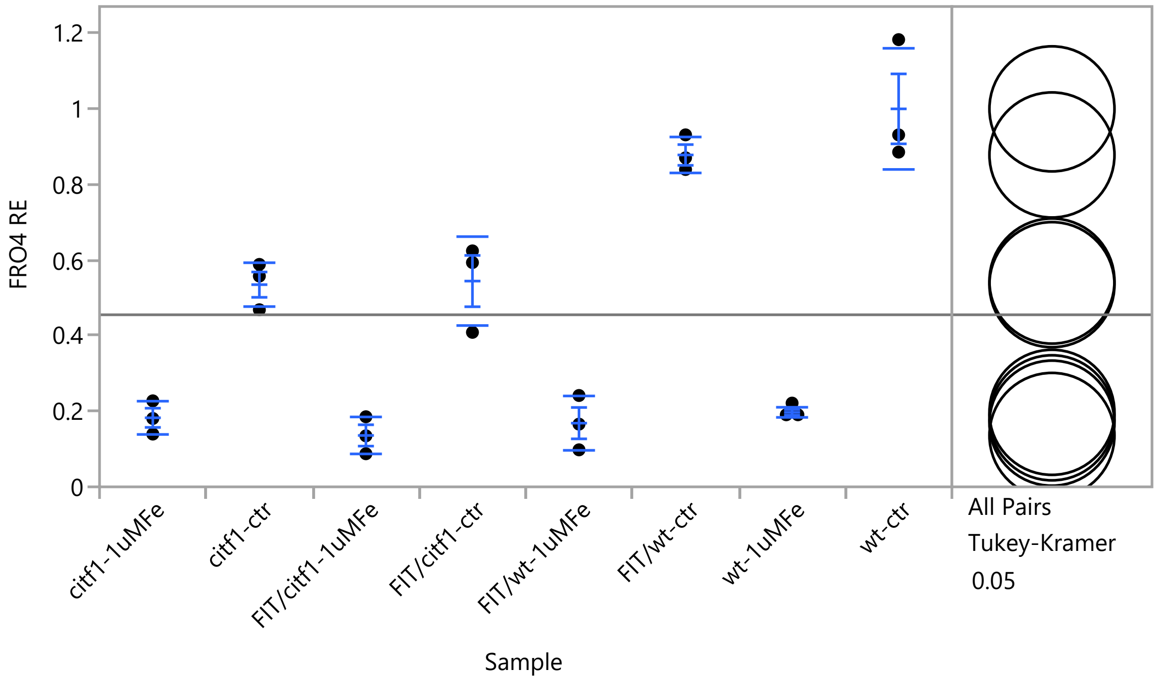

Means and Std Deviations

Level	Number	Mean	Std Dev	Std Err Mean	Lower 95%	Upper 95%
citf1-1uMFe	3	0.1820798	0.044014	0.0254115	0.0727428	0.2914167
citf1-ctr	3	0.5345854	0.0580616	0.0335219	0.3903524	0.6788185
FIT/citf1-1uMFe	3	0.1353955	0.0489434	0.0282575	0.0138133	0.2569777
FIT/citf1-ctr	3	0.5440741	0.1176857	0.0679459	0.2517265	0.8364217
FIT/wt-1uMFe	3	0.1679171	0.0719693	0.0415515	-0.010865	0.3466988
FIT/wt-ctr	3	0.8779637	0.0475898	0.027476	0.759744	0.9961834
wt-1uMFe	3	0.1965918	0.0133811	0.0077256	0.1633514	0.2298322
wt-ctr	3	0.9999955	0.1603717	0.0925906	0.6016101	1.3983809

Means Comparisons

Comparisons for all pairs using Tukey-Kramer HSD

Confidence Quantile

q*	Alpha
3.46215	0.05

Oneway Analysis of FRO4 RE By Sample

Means Comparisons

Comparisons for all pairs using Tukey-Kramer HSD

HSD Threshold Matrix

Abs(Dif)-HSD

	wt-ctr	FIT/wt-ctr	FIT/citf1-ctr	citf1-ctr	wt-1uMFe	citf1-1uMFe	FIT/wt-1uMFe	FIT/citf1-1u[illegible]
wt-ctr	-0.23417	-0.11214	0.22175	0.23124	0.56924	0.58375	0.59791	0.63[illegible]
FIT/wt-ctr	-0.11214	-0.23417	0.09972	0.10921	0.44720	0.46172	0.47588	0.50[illegible]
FIT/citf1-ctr	0.22175	0.09972	-0.23417	-0.22468	0.11331	0.12783	0.14199	0.17[illegible]
citf1-ctr	0.23124	0.10921	-0.22468	-0.23417	0.10383	0.11834	0.13250	0.16[illegible]
wt-1uMFe	0.56924	0.44720	0.11331	0.10383	-0.23417	-0.21966	-0.20549	-0.17[illegible]
citf1-1uMFe	0.58375	0.46172	0.12783	0.11834	-0.21966	-0.23417	-0.22000	-0.18[illegible]
FIT/wt-1uMFe	0.59791	0.47588	0.14199	0.13250	-0.20549	-0.22000	-0.23417	-0.20[illegible]
FIT/citf1-1uMFe	0.63043	0.50840	0.17451	0.16502	-0.17297	-0.18748	-0.20165	-0.23[illegible]

Positive values show pairs of means that are significantly different.

Connecting Letters Report

Level				Mean	Std Error
wt-ctr	A			1.0000	0.04783
FIT/wt-ctr	A			0.8780	0.04783
FIT/citf1-ctr		B		0.5441	0.04783
citf1-ctr		B		0.5346	0.04783
wt-1uMFe			C	0.1966	0.04783
citf1-1uMFe			C	0.1821	0.04783
FIT/wt-1uMFe			C	0.1679	0.04783
FIT/citf1-1uMFe			C	0.1354	0.04783

Levels not connected by same letter are significantly different.

MFe
3043
0840
7451
5502
7297
3748
0165
3417

Oneway Analysis of FRO4 RE By Sample

Means Comparisons

Comparisons for all pairs using Tukey-Kramer HSD

Ordered Differences Report

Level	- Level	Difference	Std Err Dif	Lower CL	Upper CL	p-Value
wt-ctr	FIT/citf1-1uMFe	0.8646000	0.0676365	0.630432	1.098768	<.0001*
wt-ctr	FIT/wt-1uMFe	0.8320784	0.0676365	0.597911	1.066246	<.0001*
wt-ctr	citf1-1uMFe	0.8179157	0.0676365	0.583748	1.052083	<.0001*
wt-ctr	wt-1uMFe	0.8034037	0.0676365	0.569236	1.037571	<.0001*
FIT/wt-ctr	FIT/citf1-1uMFe	0.7425682	0.0676365	0.508401	0.976736	<.0001*
FIT/wt-ctr	FIT/wt-1uMFe	0.7100466	0.0676365	0.475879	0.944214	<.0001*
FIT/wt-ctr	citf1-1uMFe	0.6958839	0.0676365	0.461716	0.930052	<.0001*
FIT/wt-ctr	wt-1uMFe	0.6813719	0.0676365	0.447204	0.915540	<.0001*
wt-ctr	citf1-ctr	0.4654101	0.0676365	0.231242	0.699578	<.0001*
wt-ctr	FIT/citf1-ctr	0.4559214	0.0676365	0.221754	0.690089	0.0001*
FIT/citf1-ctr	FIT/citf1-1uMFe	0.4086786	0.0676365	0.174511	0.642846	0.0004*
citf1-ctr	FIT/citf1-1uMFe	0.3991899	0.0676365	0.165022	0.633358	0.0005*
FIT/citf1-ctr	FIT/wt-1uMFe	0.3761570	0.0676365	0.141989	0.610325	0.0009*
citf1-ctr	FIT/wt-1uMFe	0.3666683	0.0676365	0.132501	0.600836	0.0011*
FIT/citf1-ctr	citf1-1uMFe	0.3619943	0.0676365	0.127827	0.596162	0.0013*
citf1-ctr	citf1-1uMFe	0.3525056	0.0676365	0.118338	0.586673	0.0017*
FIT/citf1-ctr	wt-1uMFe	0.3474823	0.0676365	0.113315	0.581650	0.0020*
FIT/wt-ctr	citf1-ctr	0.3433783	0.0676365	0.109211	0.577546	0.0022*
citf1-ctr	wt-1uMFe	0.3379936	0.0676365	0.103826	0.572161	0.0026*
FIT/wt-ctr	FIT/citf1-ctr	0.3338896	0.0676365	0.099722	0.568057	0.0029*
wt-ctr	FIT/wt-ctr	0.1220318	0.0676365	-0.112136	0.356199	0.6262
wt-1uMFe	FIT/citf1-1uMFe	0.0611963	0.0676365	-0.172971	0.295364	0.9812
citf1-1uMFe	FIT/citf1-1uMFe	0.0466843	0.0676365	-0.187483	0.280852	0.9961
FIT/wt-1uMFe	FIT/citf1-1uMFe	0.0325216	0.0676365	-0.201646	0.266689	0.9996
wt-1uMFe	FIT/wt-1uMFe	0.0286747	0.0676365	-0.205493	0.262842	0.9998
wt-1uMFe	citf1-1uMFe	0.0145120	0.0676365	-0.219656	0.248680	1.0000
citf1-1uMFe	FIT/wt-1uMFe	0.0141627	0.0676365	-0.220005	0.248330	1.0000
FIT/citf1-ctr	citf1-ctr	0.0094887	0.0676365	-0.224679	0.243656	1.0000

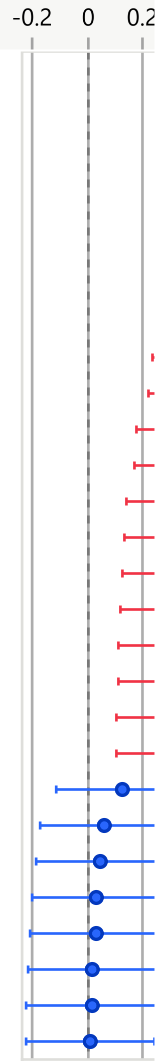

Missing Rows 4

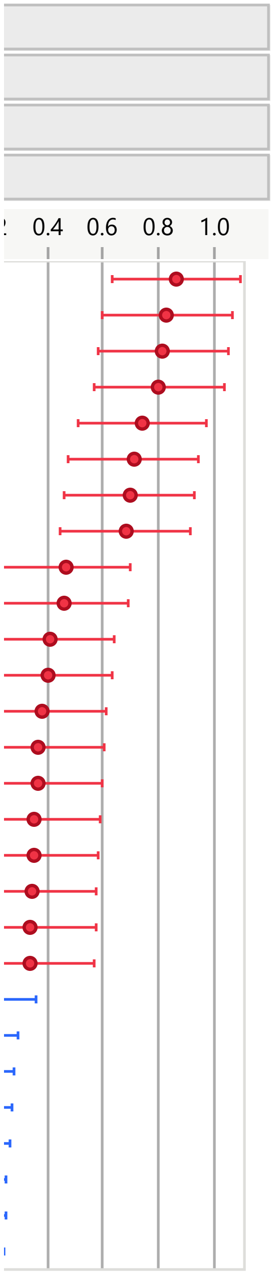
0.4
0.6
0.8
1.0

Oneway Analysis of FRO5 RE By Sample

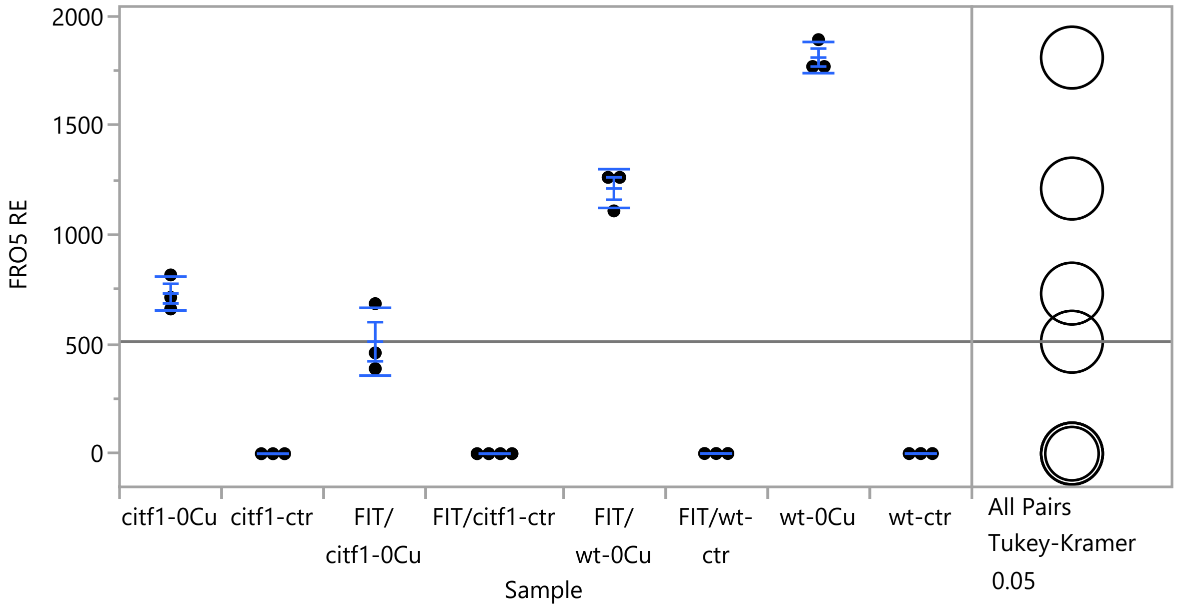

Means and Std Deviations

Level	Number	Mean	Std Dev	Std Err Mean	Lower 95%	Upper 95%
citf1-0Cu	3	731.43523	77.404635	44.689587	539.15145	923.719
citf1-ctr	3	0.418735	0.3505482	0.2023891	-0.452075	1.2895449
FIT/citf1-0Cu	3	511.74171	154.737	89.33745	127.35368	896.12973
FIT/citf1-ctr	4	0.5657653	0.5302946	0.2651473	-0.278052	1.4095823
FIT/wt-0Cu	3	1211.0411	88.509187	51.100803	991.17205	1430.9101
FIT/wt-ctr	3	1.3237067	0.0812885	0.046932	1.1217748	1.5256386
wt-0Cu	3	1809.0252	71.192164	41.102815	1632.1741	1985.8764
wt-ctr	3	0.9999758	0.3871263	0.2235075	0.0383007	1.9616509

Means Comparisons

Comparisons for all pairs using Tukey-Kramer HSD

Confidence Quantile

q*	Alpha
3.43514	0.05

HSD Threshold Matrix

Abs(Dif)-HSD

	wt-0Cu	FIT/wt-0Cu	citf1-0Cu	FIT/citf1-0Cu	FIT/wt-ctr	wt-ctr	FIT/citf1-ctr	citf1-ctr
wt-0Cu	-199.1	398.9	878.5	1098.2	1608.6	1608.9	1622.2	1609.5
FIT/wt-0Cu	398.9	-199.1	280.5	500.2	1010.6	1010.9	1024.2	1011.5
citf1-0Cu	878.5	280.5	-199.1	20.6	531.0	531.3	544.6	531.9
FIT/citf1-0Cu	1098.2	500.2	20.6	-199.1	311.3	311.6	324.9	312.2
FIT/wt-ctr	1608.6	1010.6	531.0	311.3	-199.1	-198.8	-185.5	-198.2
wt-ctr	1608.9	1010.9	531.3	311.6	-198.8	-199.1	-185.8	-198.5
FIT/citf1-ctr	1622.2	1024.2	544.6	324.9	-185.5	-185.8	-172.4	-186.1
citf1-ctr	1609.5	1011.5	531.9	312.2	-198.2	-198.5	-186.1	-199.1

Positive values show pairs of means that are significantly different.

Oneway Analysis of FRO5 RE By Sample

Means Comparisons

Comparisons for all pairs using Tukey-Kramer HSD

Connecting Letters Report

Level						Mean	Std Error
wt-0Cu	A					1809.0	40.987
FIT/wt-0Cu		B				1211.0	40.987
citf1-0Cu			C			731.4	40.987
FIT/citf1-0Cu				D		511.7	40.987
FIT/wt-ctr					E	1.3	40.987
wt-ctr					E	1.0	40.987
FIT/citf1-ctr					E	0.56577	35.496
citf1-ctr					E	0.41873	40.987

Levels not connected by same letter are significantly different.

Ordered Differences Report

Level	- Level	Difference	Std Err Dif	Lower CL	Upper CL	p-Value
wt-0Cu	citf1-ctr	1808.606	57.96410	1609.49	2007.721	<.0001*
wt-0Cu	FIT/citf1-ctr	1808.459	54.22045	1622.20	1994.714	<.0001*
wt-0Cu	wt-ctr	1808.025	57.96410	1608.91	2007.140	<.0001*
wt-0Cu	FIT/wt-ctr	1807.702	57.96410	1608.59	2006.816	<.0001*
wt-0Cu	FIT/citf1-0Cu	1297.284	57.96410	1098.17	1496.398	<.0001*
FIT/wt-0Cu	citf1-ctr	1210.622	57.96410	1011.51	1409.737	<.0001*
FIT/wt-0Cu	FIT/citf1-ctr	1210.475	54.22045	1024.22	1396.730	<.0001*
FIT/wt-0Cu	wt-ctr	1210.041	57.96410	1010.93	1409.156	<.0001*
FIT/wt-0Cu	FIT/wt-ctr	1209.717	57.96410	1010.60	1408.832	<.0001*
wt-0Cu	citf1-0Cu	1077.590	57.96410	878.48	1276.705	<.0001*
citf1-0Cu	citf1-ctr	731.016	57.96410	531.90	930.131	<.0001*
citf1-0Cu	FIT/citf1-ctr	730.869	54.22045	544.61	917.124	<.0001*
citf1-0Cu	wt-ctr	730.435	57.96410	531.32	929.550	<.0001*
citf1-0Cu	FIT/wt-ctr	730.112	57.96410	531.00	929.226	<.0001*
FIT/wt-0Cu	FIT/citf1-0Cu	699.299	57.96410	500.18	898.414	<.0001*
wt-0Cu	FIT/wt-0Cu	597.984	57.96410	398.87	797.099	<.0001*
FIT/citf1-0Cu	citf1-ctr	511.323	57.96410	312.21	710.438	<.0001*
FIT/citf1-0Cu	FIT/citf1-ctr	511.176	54.22045	324.92	697.431	<.0001*
FIT/citf1-0Cu	wt-ctr	510.742	57.96410	311.63	709.856	<.0001*
FIT/citf1-0Cu	FIT/wt-ctr	510.418	57.96410	311.30	709.533	<.0001*
FIT/wt-0Cu	citf1-0Cu	479.606	57.96410	280.49	678.721	<.0001*
citf1-0Cu	FIT/citf1-0Cu	219.694	57.96410	20.58	418.808	0.0250*
FIT/wt-ctr	citf1-ctr	0.905	57.96410	-198.21	200.020	1.0000
FIT/wt-ctr	FIT/citf1-ctr	0.758	54.22045	-185.50	187.013	1.0000
wt-ctr	citf1-ctr	0.581	57.96410	-198.53	199.696	1.0000
wt-ctr	FIT/citf1-ctr	0.434	54.22045	-185.82	186.689	1.0000
FIT/wt-ctr	wt-ctr	0.324	57.96410	-198.79	199.438	1.0000
FIT/citf1-ctr	citf1-ctr	0.147	54.22045	-186.11	186.402	1.0000

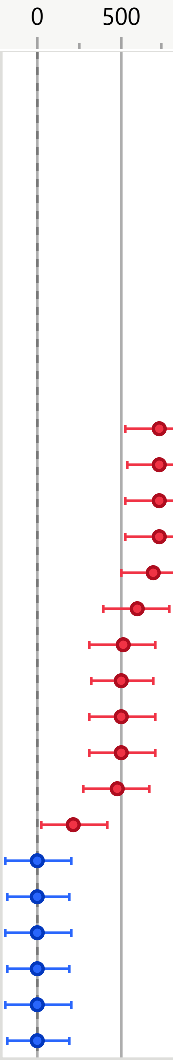

Missing Rows 16

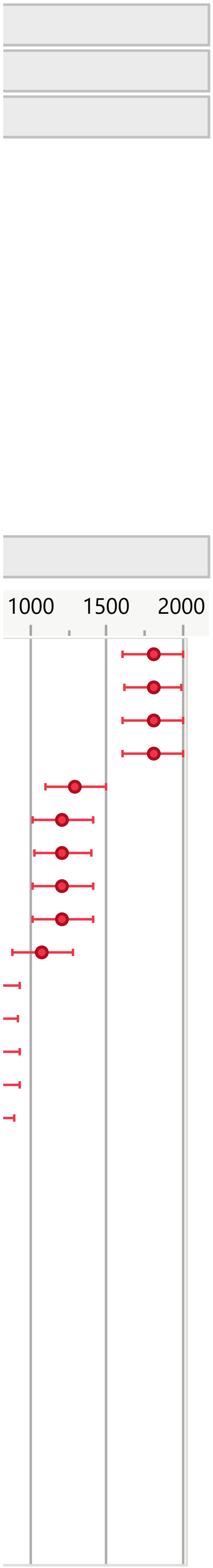
1000
1500
2000

Oneway Analysis of FRO5 RE By Sample

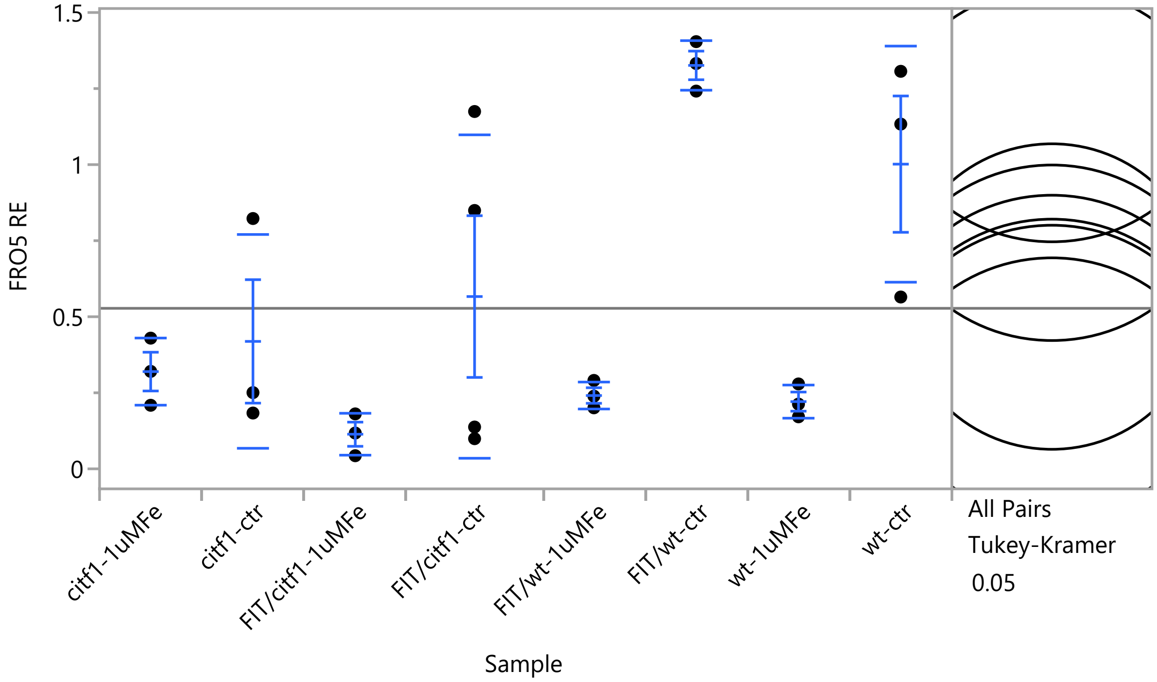

Means and Std Deviations

Level	Number	Mean	Std Dev	Std Err Mean	Lower 95%	Upper 95%
citf1-1uMFe	3	0.3196653	0.1100687	0.0635482	0.0462395	0.5930911
citf1-ctr	3	0.418735	0.3505482	0.2023891	-0.452075	1.2895449
FIT/citf1-1uMFe	3	0.1143168	0.0686727	0.0396482	-0.056276	0.2849091
FIT/citf1-ctr	4	0.5657653	0.5302946	0.2651473	-0.278052	1.4095823
FIT/wt-1uMFe	3	0.2412045	0.0441928	0.0255147	0.1314236	0.3509854
FIT/wt-ctr	3	1.3237067	0.0812885	0.046932	1.1217748	1.5256386
wt-1uMFe	3	0.2212482	0.0543373	0.0313716	0.0862669	0.3562295
wt-ctr	3	0.9999758	0.3871263	0.2235075	0.0383007	1.9616509

Means Comparisons

Comparisons for all pairs using Tukey-Kramer HSD

Confidence Quantile

q*	Alpha
3.43514	0.05

Oneway Analysis of FRO5 RE By Sample

Means Comparisons

Comparisons for all pairs using Tukey-Kramer HSD

HSD Threshold Matrix

Abs(Dif)-HSD

	FIT/wt-ctr	wt-ctr	FIT/citf1-ctr	citf1-ctr	citf1-1uMFe	FIT/wt-1uMFe	wt-1uMFe	FIT/citf1-1u
FIT/wt-ctr	-0.81796	-0.49423	-0.00719	0.08701	0.18608	0.26454	0.28450	0.39
wt-ctr	-0.49423	-0.81796	-0.33092	-0.23672	-0.13765	-0.05919	-0.03923	0.06
FIT/citf1-ctr	-0.00719	-0.33092	-0.70837	-0.61810	-0.51903	-0.44057	-0.42062	-0.3
citf1-ctr	0.08701	-0.23672	-0.61810	-0.81796	-0.71889	-0.64043	-0.62047	-0.5
citf1-1uMFe	0.18608	-0.13765	-0.51903	-0.71889	-0.81796	-0.73950	-0.71954	-0.6
FIT/wt-1uMFe	0.26454	-0.05919	-0.44057	-0.64043	-0.73950	-0.81796	-0.79800	-0.69
wt-1uMFe	0.28450	-0.03923	-0.42062	-0.62047	-0.71954	-0.79800	-0.81796	-0.7
FIT/citf1-1uMFe	0.39143	0.06770	-0.31368	-0.51354	-0.61261	-0.69107	-0.71103	-0.8

Positive values show pairs of means that are significantly different.

Connecting Letters Report

Level				Mean	Std Error
FIT/wt-ctr	A			1.3237	0.16837
wt-ctr	A	B		1.0000	0.16837
FIT/citf1-ctr	A	B	C	0.5658	0.14582
citf1-ctr		B	C	0.4187	0.16837
citf1-1uMFe		B	C	0.3197	0.16837
FIT/wt-1uMFe		B	C	0.2412	0.16837
wt-1uMFe		B	C	0.2212	0.16837
FIT/citf1-1uMFe			C	0.1143	0.16837

Levels not connected by same letter are significantly different.

MFe
9143
5770
1368
1354
1261
9107
1103
1796

Oneway Analysis of FRO5 RE By Sample

Means Comparisons

Comparisons for all pairs using Tukey-Kramer HSD

Ordered Differences Report

Level	- Level	Difference	Std Err Dif	Lower CL	Upper CL	p-Value
FIT/wt-ctr	FIT/citf1-1uMFe	1.209390	0.2381158	0.391429	2.027351	0.0019*
FIT/wt-ctr	wt-1uMFe	1.102458	0.2381158	0.284498	1.920419	0.0046*
FIT/wt-ctr	FIT/wt-1uMFe	1.082502	0.2381158	0.264541	1.900463	0.0055*
FIT/wt-ctr	citf1-1uMFe	1.004041	0.2381158	0.186081	1.822002	0.0107*
FIT/wt-ctr	citf1-ctr	0.904972	0.2381158	0.087011	1.722933	0.0245*
wt-ctr	FIT/citf1-1uMFe	0.885659	0.2381158	0.067698	1.703620	0.0287*
wt-ctr	wt-1uMFe	0.778728	0.2381158	-0.039233	1.596688	0.0684
wt-ctr	FIT/wt-1uMFe	0.758771	0.2381158	-0.059190	1.576732	0.0799
FIT/wt-ctr	FIT/citf1-ctr	0.757941	0.2227369	-0.007191	1.523074	0.0532
wt-ctr	citf1-1uMFe	0.680311	0.2381158	-0.137650	1.498271	0.1444
wt-ctr	citf1-ctr	0.581241	0.2381158	-0.236720	1.399202	0.2832
FIT/citf1-ctr	FIT/citf1-1uMFe	0.451448	0.2227369	-0.313684	1.216581	0.4937
wt-ctr	FIT/citf1-ctr	0.434211	0.2227369	-0.330922	1.199343	0.5390
FIT/citf1-ctr	wt-1uMFe	0.344517	0.2227369	-0.420615	1.109649	0.7732
FIT/citf1-ctr	FIT/wt-1uMFe	0.324561	0.2227369	-0.440572	1.089693	0.8187
FIT/wt-ctr	wt-ctr	0.323731	0.2381158	-0.494230	1.141692	0.8629
citf1-ctr	FIT/citf1-1uMFe	0.304418	0.2381158	-0.513543	1.122379	0.8948
FIT/citf1-ctr	citf1-1uMFe	0.246100	0.2227369	-0.519032	1.011232	0.9470
citf1-1uMFe	FIT/citf1-1uMFe	0.205348	0.2381158	-0.612612	1.023309	0.9858
citf1-ctr	wt-1uMFe	0.197487	0.2381158	-0.620474	1.015448	0.9887
citf1-ctr	FIT/wt-1uMFe	0.177531	0.2381158	-0.640430	0.995491	0.9939
FIT/citf1-ctr	citf1-ctr	0.147030	0.2227369	-0.618102	0.912163	0.9971
FIT/wt-1uMFe	FIT/citf1-1uMFe	0.126888	0.2381158	-0.691073	0.944849	0.9992
wt-1uMFe	FIT/citf1-1uMFe	0.106931	0.2381158	-0.711029	0.924892	0.9998
citf1-ctr	citf1-1uMFe	0.099070	0.2381158	-0.718891	0.917031	0.9999
citf1-1uMFe	wt-1uMFe	0.098417	0.2381158	-0.719544	0.916378	0.9999
citf1-1uMFe	FIT/wt-1uMFe	0.078461	0.2381158	-0.739500	0.896422	1.0000
FIT/wt-1uMFe	wt-1uMFe	0.019956	0.2381158	-0.798005	0.837917	1.0000

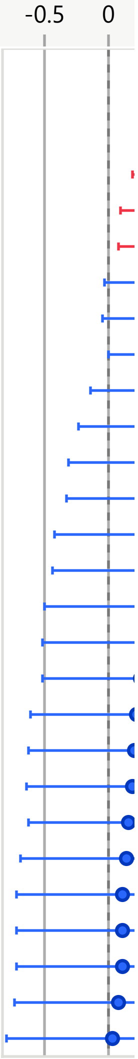

Missing Rows 11

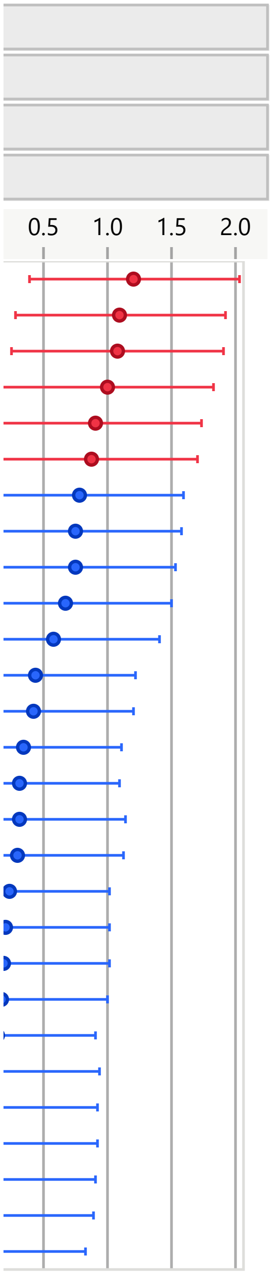
0.5
1.0
1.5
2.0

Oneway Analysis of Relative expression By Sample

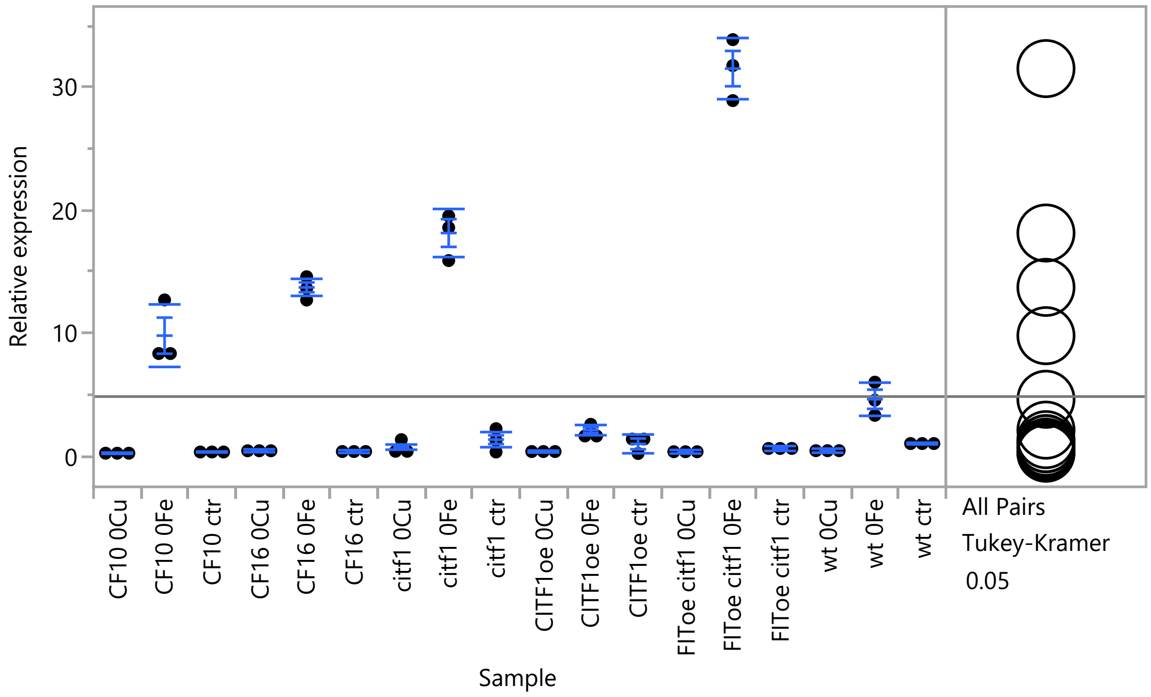

Means and Std Deviations

Level	Number	Mean	Std Dev	Std Err Mean	Lower 95%	Upper 95%
CF10 0Cu	3	0.2118804	0.0523278	0.0302115	0.0818909	0.34187
CF10 0Fe	3	9.787665	2.5477147	1.4709238	3.4587907	16.116539
CF10 ctr	3	0.3125759	0.0372855	0.0215268	0.2199535	0.4051982
CF16 0Cu	3	0.4122425	0.1181863	0.0682349	0.1186513	0.7058336
CF16 0Fe	3	13.722187	0.6938362	0.4005865	11.998603	15.445772
CF16 ctr	3	0.3493786	0.1176352	0.0679167	0.0571565	0.6416007
citf1 0Cu	3	0.7093908	0.211562	0.1221454	0.1838416	1.23494
citf1 0Fe	3	18.149142	1.9589746	1.1310145	13.282779	23.015504
citf1 ctr	3	1.3174468	0.6166739	0.3560368	-0.214456	2.8493495
CITF1oe 0Cu	3	0.3492768	0.0780337	0.0450528	0.1554302	0.5431233
CITF1oe 0Fe	3	2.0939258	0.4181484	0.2414181	1.0551877	3.1326639
CITF1oe ctr	3	0.9732111	0.7658411	0.4421585	-0.929244	2.8756657
FIToe citf1 0Cu	3	0.334162	0.1456226	0.0840752	-0.027585	0.6959086
FIToe citf1 0Fe	3	31.541861	2.4924627	1.439024	25.350241	37.733482
FIToe citf1 ctr	3	0.6004335	0.1986806	0.1147083	0.1068837	1.0939834
wt 0Cu	3	0.4196213	0.1682533	0.0971411	0.0016568	0.8375857
wt 0Fe	3	4.6077875	1.3526891	0.7809754	1.2475216	7.9680534
wt ctr	3	1.0000005	0.0694262	0.0400833	0.8275361	1.1724648

Means Comparisons

Comparisons for all pairs using Tukey-Kramer HSD

Confidence Quantile

q*	Alpha
3.75036	0.05

Oneway Analysis of Relative expression By Sample

Means Comparisons

Comparisons for all pairs using Tukey-Kramer HSD

HSD Threshold Matrix

Abs(Dif)-HSD

	FIToe citf1 0Fe	citf1 0Fe	CF16 0Fe	CF10 0Fe	wt 0Fe	CITF1oe 0Fe	citf1 ctr	wt ctr
FIToe citf1 0Fe	-3.242	10.151	14.578	18.513	23.693	26.206	26.983	27.300
citf1 0Fe	10.151	-3.242	1.185	5.120	10.300	12.814	13.590	13.908
CF16 0Fe	14.578	1.185	-3.242	0.693	5.873	8.387	9.163	9.481
CF10 0Fe	18.513	5.120	0.693	-3.242	1.938	4.452	5.229	5.546
wt 0Fe	23.693	10.300	5.873	1.938	-3.242	-0.728	0.049	0.366
CITF1oe 0Fe	26.206	12.814	8.387	4.452	-0.728	-3.242	-2.465	-2.148
citf1 ctr	26.983	13.590	9.163	5.229	0.049	-2.465	-3.242	-2.924
wt ctr	27.300	13.908	9.481	5.546	0.366	-2.148	-2.924	-3.242
CITF1oe ctr	27.327	13.934	9.507	5.573	0.393	-2.121	-2.897	-3.215
citf1 0Cu	27.591	14.198	9.771	5.837	0.657	-1.857	-2.633	-2.951
FIToe citf1 ctr	27.700	14.307	9.880	5.946	0.766	-1.748	-2.524	-2.842
wt 0Cu	27.881	14.488	10.061	6.127	0.947	-1.567	-2.344	-2.661
CF16 0Cu	27.888	14.495	10.068	6.134	0.954	-1.560	-2.336	-2.654
CF16 ctr	27.951	14.558	10.131	6.197	1.017	-1.497	-2.273	-2.591
CITF1oe 0Cu	27.951	14.558	10.131	6.197	1.017	-1.497	-2.273	-2.591
FIToe citf1 0Cu	27.966	14.573	10.147	6.212	1.032	-1.482	-2.258	-2.576
CF10 ctr	27.988	14.595	10.168	6.234	1.054	-1.460	-2.237	-2.554
CF10 0Cu	28.088	14.696	10.269	6.334	1.154	-1.359	-2.136	-2.453

Positive values show pairs of means that are significantly different.

Connecting Letters Report

Level							Mean	Std Error
FIToe citf1 0Fe	A						31.542	0.61116
citf1 0Fe		B					18.149	0.61116
CF16 0Fe			C				13.722	0.61116
CF10 0Fe				D			9.788	0.61116
wt 0Fe					E		4.608	0.61116
CITF1oe 0Fe					E	F	2.094	0.61116
citf1 ctr						F	1.317	0.61116
wt ctr						F	1.000	0.61116
CITF1oe ctr						F	0.973	0.61116
citf1 0Cu						F	0.709	0.61116
FIToe citf1 ctr						F	0.600	0.61116
wt 0Cu						F	0.420	0.61116
CF16 0Cu						F	0.412	0.61116
CF16 ctr						F	0.349	0.61116
CITF1oe 0Cu						F	0.349	0.61116
FIToe citf1 0Cu						F	0.334	0.61116
CF10 ctr						F	0.313	0.61116
CF10 0Cu						F	0.212	0.61116

Levels not connected by same letter are significantly different.

CITF1oe ctr	citf1 0Cu	FIToe citf1 ctr	wt 0Cu	CF16 0Cu	CF16 ctr	CITF1oe 0Cu	FIToe citf1 0Cu	CF10 ctr	CF10 0Cu
27.327	27.591	27.700	27.881	27.888	27.951	27.951	27.966	27.988	28.088
13.934	14.198	14.307	14.488	14.495	14.558	14.558	14.573	14.595	14.696
9.507	9.771	9.880	10.061	10.068	10.131	10.131	10.147	10.168	10.269
5.573	5.837	5.946	6.127	6.134	6.197	6.197	6.212	6.234	6.334
0.393	0.657	0.766	0.947	0.954	1.017	1.017	1.032	1.054	1.154
-2.121	-1.857	-1.748	-1.567	-1.560	-1.497	-1.497	-1.482	-1.460	-1.359
-2.897	-2.633	-2.524	-2.344	-2.336	-2.273	-2.273	-2.258	-2.237	-2.136
-3.215	-2.951	-2.842	-2.661	-2.654	-2.591	-2.591	-2.576	-2.554	-2.453
-3.242	-2.978	-2.869	-2.688	-2.681	-2.618	-2.618	-2.602	-2.581	-2.480
-2.978	-3.242	-3.133	-2.952	-2.944	-2.881	-2.881	-2.866	-2.845	-2.744
-2.869	-3.133	-3.242	-3.061	-3.053	-2.990	-2.990	-2.975	-2.954	-2.853
-2.688	-2.952	-3.061	-3.242	-3.234	-3.171	-3.171	-3.156	-3.134	-3.034
-2.681	-2.944	-3.053	-3.234	-3.242	-3.179	-3.179	-3.163	-3.142	-3.041
-2.618	-2.881	-2.990	-3.171	-3.179	-3.242	-3.241	-3.226	-3.205	-3.104
-2.618	-2.881	-2.990	-3.171	-3.179	-3.241	-3.242	-3.226	-3.205	-3.104
-2.602	-2.866	-2.975	-3.156	-3.163	-3.226	-3.226	-3.242	-3.220	-3.119
-2.581	-2.845	-2.954	-3.134	-3.142	-3.205	-3.205	-3.220	-3.242	-3.141
-2.480	-2.744	-2.853	-3.034	-3.041	-3.104	-3.104	-3.119	-3.141	-3.242

Oneway Analysis of Relative expression By Sample

Means Comparisons

Comparisons for all pairs using Tukey-Kramer HSD

Ordered Differences Report

Level	- Level	Difference	Std Err Dif	Lower CL	Upper CL	p-Value
FITοe citf1 0Fe	CF10 0Cu	31.32998	0.8643177	28.0885	34.57148	<.0001*
FIToe citf1 0Fe	CF10 ctr	31.22929	0.8643177	27.9878	34.47079	<.0001*
FIToe citf1 0Fe	FIToe citf1 0Cu	31.20770	0.8643177	27.9662	34.44920	<.0001*
FIToe citf1 0Fe	CITF1oe 0Cu	31.19258	0.8643177	27.9511	34.43409	<.0001*
FIToe citf1 0Fe	CF16 ctr	31.19248	0.8643177	27.9510	34.43399	<.0001*
FIToe citf1 0Fe	CF16 0Cu	31.12962	0.8643177	27.8881	34.37112	<.0001*
FIToe citf1 0Fe	wt 0Cu	31.12224	0.8643177	27.8807	34.36374	<.0001*
FIToe citf1 0Fe	FIToe citf1 ctr	30.94143	0.8643177	27.6999	34.18293	<.0001*
FIToe citf1 0Fe	citf1 0Cu	30.83247	0.8643177	27.5910	34.07397	<.0001*
FIToe citf1 0Fe	CITF1oe ctr	30.56865	0.8643177	27.3271	33.81015	<.0001*
FIToe citf1 0Fe	wt ctr	30.54186	0.8643177	27.3004	33.78336	<.0001*
FIToe citf1 0Fe	citf1 ctr	30.22441	0.8643177	26.9829	33.46592	<.0001*
FIToe citf1 0Fe	CITF1oe 0Fe	29.44794	0.8643177	26.2064	32.68944	<.0001*
FIToe citf1 0Fe	wt 0Fe	26.93407	0.8643177	23.6926	30.17558	<.0001*
FIToe citf1 0Fe	CF10 0Fe	21.75420	0.8643177	18.5127	24.99570	<.0001*
citf1 0Fe	CF10 0Cu	17.93726	0.8643177	14.6958	21.17876	<.0001*
citf1 0Fe	CF10 ctr	17.83657	0.8643177	14.5951	21.07807	<.0001*
FIToe citf1 0Fe	CF16 0Fe	17.81967	0.8643177	14.5782	21.06118	<.0001*
citf1 0Fe	FIToe citf1 0Cu	17.81498	0.8643177	14.5735	21.05648	<.0001*
citf1 0Fe	CITF1oe 0Cu	17.79987	0.8643177	14.5584	21.04137	<.0001*
citf1 0Fe	CF16 ctr	17.79976	0.8643177	14.5583	21.04127	<.0001*
citf1 0Fe	CF16 0Cu	17.73690	0.8643177	14.4954	20.97840	<.0001*
citf1 0Fe	wt 0Cu	17.72952	0.8643177	14.4880	20.97102	<.0001*
citf1 0Fe	FIToe citf1 ctr	17.54871	0.8643177	14.3072	20.79021	<.0001*
citf1 0Fe	citf1 0Cu	17.43975	0.8643177	14.1982	20.68125	<.0001*
citf1 0Fe	CITF1oe ctr	17.17593	0.8643177	13.9344	20.41743	<.0001*
citf1 0Fe	wt ctr	17.14914	0.8643177	13.9076	20.39064	<.0001*
citf1 0Fe	citf1 ctr	16.83170	0.8643177	13.5902	20.07320	<.0001*
citf1 0Fe	CITF1oe 0Fe	16.05522	0.8643177	12.8137	19.29672	<.0001*
citf1 0Fe	wt 0Fe	13.54135	0.8643177	10.2999	16.78286	<.0001*
CF16 0Fe	CF10 0Cu	13.51031	0.8643177	10.2688	16.75181	<.0001*
CF16 0Fe	CF10 ctr	13.40961	0.8643177	10.1681	16.65111	<.0001*
FIToe citf1 0Fe	citf1 0Fe	13.39272	0.8643177	10.1512	16.63422	<.0001*
CF16 0Fe	FIToe citf1 0Cu	13.38803	0.8643177	10.1465	16.62953	<.0001*
CF16 0Fe	CITF1oe 0Cu	13.37291	0.8643177	10.1314	16.61441	<.0001*
CF16 0Fe	CF16 ctr	13.37281	0.8643177	10.1313	16.61431	<.0001*
CF16 0Fe	CF16 0Cu	13.30994	0.8643177	10.0684	16.55145	<.0001*
CF16 0Fe	wt 0Cu	13.30257	0.8643177	10.0611	16.54407	<.0001*
CF16 0Fe	FIToe citf1 ctr	13.12175	0.8643177	9.8803	16.36326	<.0001*
CF16 0Fe	citf1 0Cu	13.01280	0.8643177	9.7713	16.25430	<.0001*
CF16 0Fe	CITF1oe ctr	12.74898	0.8643177	9.5075	15.99048	<.0001*
CF16 0Fe	wt ctr	12.72219	0.8643177	9.4807	15.96369	<.0001*
CF16 0Fe	citf1 ctr	12.40474	0.8643177	9.1632	15.64624	<.0001*

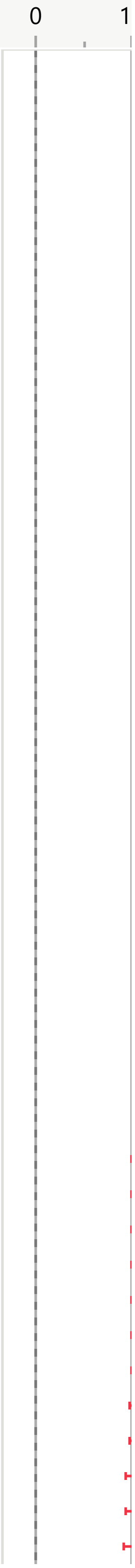

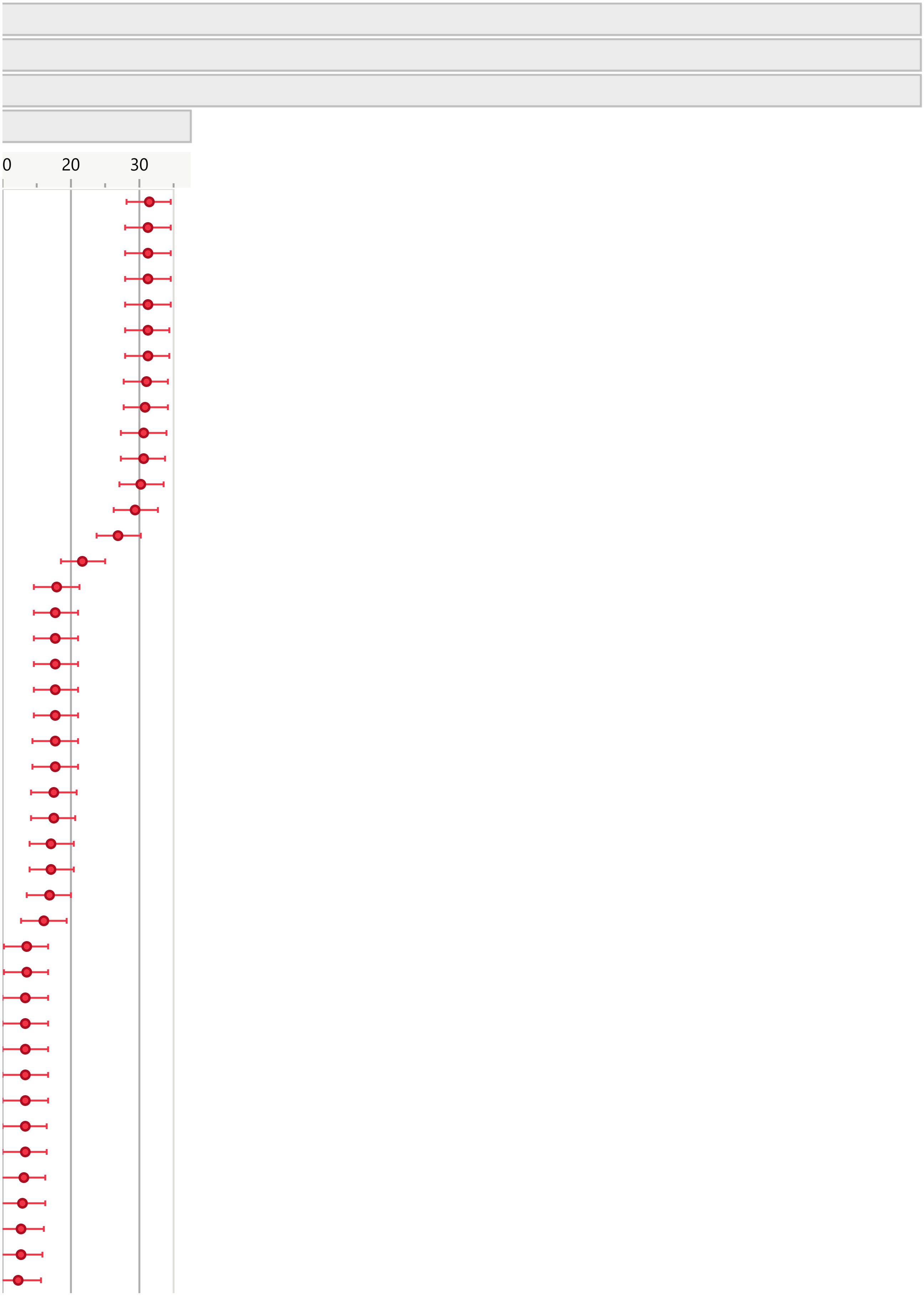
0
20
30

Oneway Analysis of Relative expression By Sample

Means Comparisons

Comparisons for all pairs using Tukey-Kramer HSD

Ordered Differences Report

Level	- Level	Difference	Std Err Dif	Lower CL	Upper CL	p-Value
CF16 0Fe	CITF1oe 0Fe	11.62826	0.8643177	8.3868	14.86976	<.0001*
CF10 0Fe	CF10 0Cu	9.57578	0.8643177	6.3343	12.81729	<.0001*
CF10 0Fe	CF10 ctr	9.47509	0.8643177	6.2336	12.71659	<.0001*
CF10 0Fe	FIToe citf1 0Cu	9.45350	0.8643177	6.2120	12.69501	<.0001*
CF10 0Fe	CITF1oe 0Cu	9.43839	0.8643177	6.1969	12.67989	<.0001*
CF10 0Fe	CF16 ctr	9.43829	0.8643177	6.1968	12.67979	<.0001*
CF10 0Fe	CF16 0Cu	9.37542	0.8643177	6.1339	12.61692	<.0001*
CF10 0Fe	wt 0Cu	9.36804	0.8643177	6.1265	12.60955	<.0001*
CF10 0Fe	FIToe citf1 ctr	9.18723	0.8643177	5.9457	12.42873	<.0001*
CF16 0Fe	wt 0Fe	9.11440	0.8643177	5.8729	12.35590	<.0001*
CF10 0Fe	citf1 0Cu	9.07827	0.8643177	5.8368	12.31978	<.0001*
CF10 0Fe	CITF1oe ctr	8.81445	0.8643177	5.5730	12.05596	<.0001*
CF10 0Fe	wt ctr	8.78766	0.8643177	5.5462	12.02917	<.0001*
CF10 0Fe	citf1 ctr	8.47022	0.8643177	5.2287	11.71172	<.0001*
citf1 0Fe	CF10 0Fe	8.36148	0.8643177	5.1200	11.60298	<.0001*
CF10 0Fe	CITF1oe 0Fe	7.69374	0.8643177	4.4522	10.93524	<.0001*
CF10 0Fe	wt 0Fe	5.17988	0.8643177	1.9384	8.42138	<.0001*
citf1 0Fe	CF16 0Fe	4.42695	0.8643177	1.1855	7.66846	0.0012*
wt 0Fe	CF10 0Cu	4.39591	0.8643177	1.1544	7.63741	0.0013*
wt 0Fe	CF10 ctr	4.29521	0.8643177	1.0537	7.53671	0.0019*
wt 0Fe	FIToe citf1 0Cu	4.27363	0.8643177	1.0321	7.51513	0.0020*
wt 0Fe	CITF1oe 0Cu	4.25851	0.8643177	1.0170	7.50001	0.0021*
wt 0Fe	CF16 ctr	4.25841	0.8643177	1.0169	7.49991	0.0021*
wt 0Fe	CF16 0Cu	4.19555	0.8643177	0.9540	7.43705	0.0026*
wt 0Fe	wt 0Cu	4.18817	0.8643177	0.9467	7.42967	0.0027*
wt 0Fe	FIToe citf1 ctr	4.00735	0.8643177	0.7659	7.24886	0.0048*
CF16 0Fe	CF10 0Fe	3.93452	0.8643177	0.6930	7.17602	0.0061*
wt 0Fe	citf1 0Cu	3.89840	0.8643177	0.6569	7.13990	0.0068*
wt 0Fe	CITF1oe ctr	3.63458	0.8643177	0.3931	6.87608	0.0156*
wt 0Fe	wt ctr	3.60779	0.8643177	0.3663	6.84929	0.0170*
wt 0Fe	citf1 ctr	3.29034	0.8643177	0.0488	6.53184	0.0435*
wt 0Fe	CITF1oe 0Fe	2.51386	0.8643177	-0.7276	5.75536	0.2967
CITF1oe 0Fe	CF10 0Cu	1.88205	0.8643177	-1.3595	5.12355	0.7547
CITF1oe 0Fe	CF10 ctr	1.78135	0.8643177	-1.4602	5.02285	0.8200
CITF1oe 0Fe	FIToe citf1 0Cu	1.75976	0.8643177	-1.4817	5.00127	0.8328
CITF1oe 0Fe	CITF1oe 0Cu	1.74465	0.8643177	-1.4969	4.98615	0.8415
CITF1oe 0Fe	CF16 ctr	1.74455	0.8643177	-1.4970	4.98605	0.8415
CITF1oe 0Fe	CF16 0Cu	1.68168	0.8643177	-1.5598	4.92319	0.8749
CITF1oe 0Fe	wt 0Cu	1.67430	0.8643177	-1.5672	4.91581	0.8785
CITF1oe 0Fe	FIToe citf1 ctr	1.49349	0.8643177	-1.7480	4.73499	0.9478
CITF1oe 0Fe	citf1 0Cu	1.38454	0.8643177	-1.8570	4.62604	0.9725
CITF1oe 0Fe	CITF1oe ctr	1.12071	0.8643177	-2.1208	4.36222	0.9967
citf1 ctr	CF10 0Cu	1.10557	0.8643177	-2.1359	4.34707	0.9972

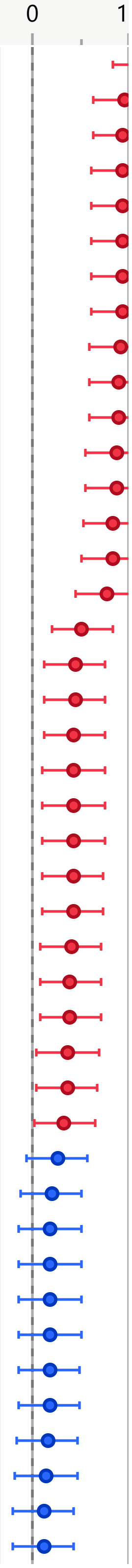

Oneway Analysis of Relative expression By Sample

Means Comparisons

Comparisons for all pairs using Tukey-Kramer HSD

Ordered Differences Report

Level	- Level	Difference	Std Err Dif	Lower CL	Upper CL	p-Value
CITF1oe 0Fe	wt ctr	1.09393	0.8643177	-2.1476	4.33543	0.9975
citf1 ctr	CF10 ctr	1.00487	0.8643177	-2.2366	4.24637	0.9991
citf1 ctr	FITоe citf1 0Cu	0.98328	0.8643177	-2.2582	4.22479	0.9993
citf1 ctr	CITF1oe 0Cu	0.96817	0.8643177	-2.2733	4.20967	0.9994
citf1 ctr	CF16 ctr	0.96807	0.8643177	-2.2734	4.20957	0.9994
citf1 ctr	CF16 0Cu	0.90520	0.8643177	-2.3363	4.14671	0.9997
citf1 ctr	wt 0Cu	0.89783	0.8643177	-2.3437	4.13933	0.9998
wt ctr	CF10 0Cu	0.78812	0.8643177	-2.4534	4.02962	1.0000
CITF1oe 0Fe	citf1 ctr	0.77648	0.8643177	-2.4650	4.01798	1.0000
CITF1oe ctr	CF10 0Cu	0.76133	0.8643177	-2.4802	4.00283	1.0000
citf1 ctr	FIToe citf1 ctr	0.71701	0.8643177	-2.5245	3.95852	1.0000
wt ctr	CF10 ctr	0.68742	0.8643177	-2.5541	3.92893	1.0000
wt ctr	FIToe citf1 0Cu	0.66584	0.8643177	-2.5757	3.90734	1.0000
CITF1oe ctr	CF10 ctr	0.66064	0.8643177	-2.5809	3.90214	1.0000
wt ctr	CITF1oe 0Cu	0.65072	0.8643177	-2.5908	3.89223	1.0000
wt ctr	CF16 ctr	0.65062	0.8643177	-2.5909	3.89212	1.0000
CITF1oe ctr	FIToe citf1 0Cu	0.63905	0.8643177	-2.6025	3.88055	1.0000
CITF1oe ctr	CITF1oe 0Cu	0.62393	0.8643177	-2.6176	3.86544	1.0000
CITF1oe ctr	CF16 ctr	0.62383	0.8643177	-2.6177	3.86533	1.0000
citf1 ctr	citf1 0Cu	0.60806	0.8643177	-2.6334	3.84956	1.0000
wt ctr	CF16 0Cu	0.58776	0.8643177	-2.6537	3.82926	1.0000
wt ctr	wt 0Cu	0.58038	0.8643177	-2.6611	3.82188	1.0000
CITF1oe ctr	CF16 0Cu	0.56097	0.8643177	-2.6805	3.80247	1.0000
CITF1oe ctr	wt 0Cu	0.55359	0.8643177	-2.6879	3.79509	1.0000
citf1 0Cu	CF10 0Cu	0.49751	0.8643177	-2.7440	3.73901	1.0000
wt ctr	FIToe citf1 ctr	0.39957	0.8643177	-2.8419	3.64107	1.0000
citf1 0Cu	CF10 ctr	0.39681	0.8643177	-2.8447	3.63832	1.0000
FIToe citf1 ctr	CF10 0Cu	0.38855	0.8643177	-2.8529	3.63006	1.0000
citf1 0Cu	FIToe citf1 0Cu	0.37523	0.8643177	-2.8663	3.61673	1.0000
CITF1oe ctr	FIToe citf1 ctr	0.37278	0.8643177	-2.8687	3.61428	1.0000
citf1 0Cu	CITF1oe 0Cu	0.36011	0.8643177	-2.8814	3.60162	1.0000
citf1 0Cu	CF16 ctr	0.36001	0.8643177	-2.8815	3.60151	1.0000
citf1 ctr	CITF1oe ctr	0.34424	0.8643177	-2.8973	3.58574	1.0000
citf1 ctr	wt ctr	0.31745	0.8643177	-2.9241	3.55895	1.0000
citf1 0Cu	CF16 0Cu	0.29715	0.8643177	-2.9444	3.53865	1.0000
wt ctr	citf1 0Cu	0.29061	0.8643177	-2.9509	3.53211	1.0000
citf1 0Cu	wt 0Cu	0.28977	0.8643177	-2.9517	3.53127	1.0000
FIToe citf1 ctr	CF10 ctr	0.28786	0.8643177	-2.9536	3.52936	1.0000
FIToe citf1 ctr	FIToe citf1 0Cu	0.26627	0.8643177	-2.9752	3.50777	1.0000
CITF1oe ctr	citf1 0Cu	0.26382	0.8643177	-2.9777	3.50532	1.0000
FIToe citf1 ctr	CITF1oe 0Cu	0.25116	0.8643177	-2.9903	3.49266	1.0000
FIToe citf1 ctr	CF16 ctr	0.25105	0.8643177	-2.9904	3.49256	1.0000
wt 0Cu	CF10 0Cu	0.20774	0.8643177	-3.0338	3.44924	1.0000

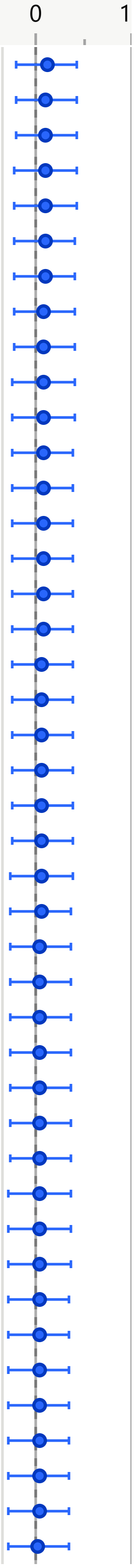

Oneway Analysis of Relative expression By Sample

Means Comparisons

Comparisons for all pairs using Tukey-Kramer HSD

Ordered Differences Report

Level	- Level	Difference	Std Err Dif	Lower CL	Upper CL	p-Value
CF16 0Cu	CF10 0Cu	0.20036	0.8643177	-3.0411	3.44186	1.0000
FIToe citf1 ctr	CF16 0Cu	0.18819	0.8643177	-3.0533	3.42969	1.0000
FIToe citf1 ctr	wt 0Cu	0.18081	0.8643177	-3.0607	3.42231	1.0000
CF16 ctr	CF10 0Cu	0.13750	0.8643177	-3.1040	3.37900	1.0000
CITF1oe 0Cu	CF10 0Cu	0.13740	0.8643177	-3.1041	3.37890	1.0000
FIToe citf1 0Cu	CF10 0Cu	0.12228	0.8643177	-3.1192	3.36378	1.0000
citf1 0Cu	FIToe citf1 ctr	0.10896	0.8643177	-3.1325	3.35046	1.0000
wt 0Cu	CF10 ctr	0.10705	0.8643177	-3.1345	3.34855	1.0000
CF10 ctr	CF10 0Cu	0.10070	0.8643177	-3.1408	3.34220	1.0000
CF16 0Cu	CF10 ctr	0.09967	0.8643177	-3.1418	3.34117	1.0000
wt 0Cu	FIToe citf1 0Cu	0.08546	0.8643177	-3.1560	3.32696	1.0000
CF16 0Cu	FIToe citf1 0Cu	0.07808	0.8643177	-3.1634	3.31958	1.0000
wt 0Cu	CITF1oe 0Cu	0.07034	0.8643177	-3.1712	3.31185	1.0000
wt 0Cu	CF16 ctr	0.07024	0.8643177	-3.1713	3.31175	1.0000
CF16 0Cu	CITF1oe 0Cu	0.06297	0.8643177	-3.1785	3.30447	1.0000
CF16 0Cu	CF16 ctr	0.06286	0.8643177	-3.1786	3.30437	1.0000
CF16 ctr	CF10 ctr	0.03680	0.8643177	-3.2047	3.27831	1.0000
CITF1oe 0Cu	CF10 ctr	0.03670	0.8643177	-3.2048	3.27820	1.0000
wt ctr	CITF1oe ctr	0.02679	0.8643177	-3.2147	3.26829	1.0000
FIToe citf1 0Cu	CF10 ctr	0.02159	0.8643177	-3.2199	3.26309	1.0000
CF16 ctr	FIToe citf1 0Cu	0.01522	0.8643177	-3.2263	3.25672	1.0000
CITF1oe 0Cu	FIToe citf1 0Cu	0.01511	0.8643177	-3.2264	3.25662	1.0000
wt 0Cu	CF16 0Cu	0.00738	0.8643177	-3.2341	3.24888	1.0000
CF16 ctr	CITF1oe 0Cu	0.00010	0.8643177	-3.2414	3.24160	1.0000

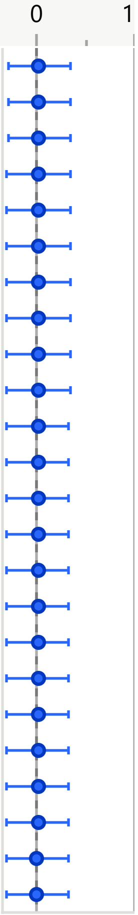

Missing Rows 54

Oneway Analysis of Relative expression By Sample

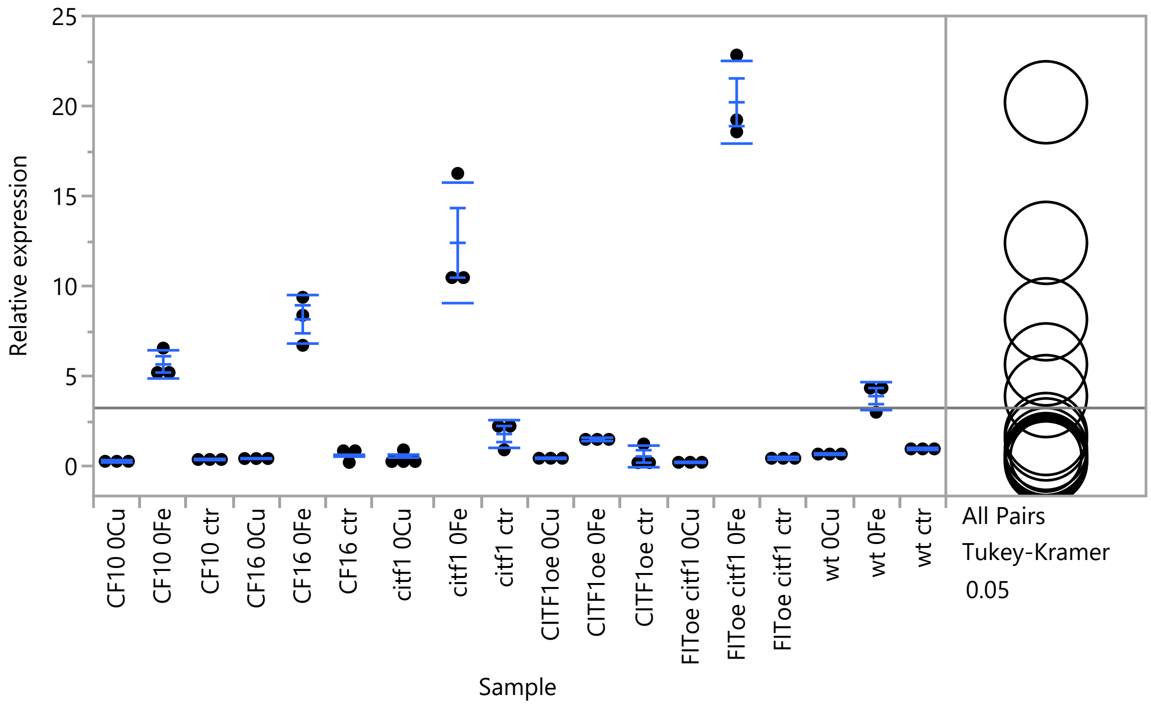

Means and Std Deviations

Level	Number	Mean	Std Dev	Std Err Mean	Lower 95%	Upper 95%
CF10 0Cu	3	0.3108204	0.0684479	0.0395184	0.1407864	0.4808544
CF10 0Fe	3	5.6842691	0.7824028	0.4517205	3.7406729	7.6278654
CF10 ctr	3	0.4129316	0.0351492	0.0202934	0.3256161	0.5002471
CF16 0Cu	3	0.4645446	0.0204658	0.0118159	0.4137048	0.5153844
CF16 0Fe	3	8.184058	1.3453208	0.7767213	4.8420958	11.52602
CF16 ctr	3	0.6219342	0.0518912	0.0299594	0.4930294	0.7508389
citf1 0Cu	4	0.5989988	0.0850839	0.0425419	0.4636114	0.7343863
citf1 0Fe	3	12.422226	3.3408256	1.9288265	4.1231547	20.721296
citf1 ctr	3	1.8240086	0.7702968	0.4447311	-0.089515	3.737532
CITF1oe 0Cu	3	0.4806393	0.035535	0.0205162	0.3923654	0.5689132
CITF1oe 0Fe	3	1.5263011	0.0935488	0.0540104	1.293913	1.7586892
CITF1oe ctr	3	0.5810946	0.6000753	0.3464537	-0.909575	2.0717644
FIToe citf1 0Cu	3	0.254519	0.0333398	0.0192487	0.1716984	0.3373397
FIToe citf1 0Fe	3	20.217151	2.2907609	1.3225714	14.526585	25.907717
FIToe citf1 ctr	3	0.478808	0.0786712	0.0454208	0.2833779	0.6742381
wt 0Cu	3	0.7116645	0.0451612	0.0260739	0.5994777	0.8238512
wt 0Fe	3	3.9253167	0.7748523	0.4473612	2.0004769	5.8501565
wt ctr	3	1.0000002	0.061054	0.0352496	0.8483336	1.1516669

Means Comparisons

Comparisons for all pairs using Tukey-Kramer HSD

Confidence Quantile

q*	Alpha
3.74317	0.05

Oneway Analysis of Relative expression By Sample

Means Comparisons

Comparisons for all pairs using Tukey-Kramer HSD

HSD Threshold Matrix

Abs(Dif)-HSD

	FITоe citf1 0Fe	citf1 0Fe	CF16 0Fe	CF10 0Fe	wt 0Fe	citf1 ctr	CITF1oe 0Fe	wt ctr
FITоe citf1 0Fe	-3.212	4.583	8.822	11.321	13.080	15.182	15.479	16.006
citf1 0Fe	4.583	-3.212	1.027	3.526	5.285	7.387	7.684	8.211
CF16 0Fe	8.822	1.027	-3.212	-0.712	1.047	3.148	3.446	3.972
CF10 0Fe	11.321	3.526	-0.712	-3.212	-1.453	0.649	0.946	1.473
wt 0Fe	13.080	5.285	1.047	-1.453	-3.212	-1.110	-0.813	-0.286
citf1 ctr	15.182	7.387	3.148	0.649	-1.110	-3.212	-2.914	-2.388
CITF1oe 0Fe	15.479	7.684	3.446	0.946	-0.813	-2.914	-3.212	-2.685
wt ctr	16.006	8.211	3.972	1.473	-0.286	-2.388	-2.685	-3.212
wt 0Cu	16.294	8.499	4.261	1.761	0.002	-2.099	-2.397	-2.923
CF16 ctr	16.384	8.589	4.351	1.851	0.092	-2.009	-2.307	-2.833
citf1 0Cu	16.614	8.819	4.581	2.081	0.322	-1.779	-2.077	-2.603
CITF1oe ctr	16.424	8.630	4.391	1.892	0.133	-1.969	-2.266	-2.793
CITF1oe 0Cu	16.525	8.730	4.492	1.992	0.233	-1.868	-2.166	-2.692
FITоe citf1 ctr	16.527	8.732	4.494	1.994	0.235	-1.866	-2.164	-2.690
CF16 0Cu	16.541	8.746	4.508	2.008	0.249	-1.852	-2.150	-2.676
CF10 ctr	16.593	8.798	4.560	2.060	0.301	-1.800	-2.098	-2.624
CF10 0Cu	16.695	8.900	4.662	2.162	0.403	-1.698	-1.996	-2.522
FITоe citf1 0Cu	16.751	8.956	4.718	2.218	0.459	-1.642	-1.940	-2.466

Positive values show pairs of means that are significantly different.

Connecting Letters Report

Level							Mean	Std Error
FITоe citf1 0Fe	A						20.217	0.60668
citf1 0Fe		B					12.422	0.60668
CF16 0Fe			C				8.184	0.60668
CF10 0Fe			C	D			5.684	0.60668
wt 0Fe				D	E		3.925	0.60668
citf1 ctr					E	F	1.824	0.60668
CITF1oe 0Fe					E	F	1.526	0.60668
wt ctr					E	F	1.000	0.60668
wt 0Cu						F	0.712	0.60668
CF16 ctr						F	0.622	0.60668
citf1 0Cu						F	0.599	0.52540
CITF1oe ctr						F	0.581	0.60668
CITF1oe 0Cu						F	0.481	0.60668
FITоe citf1 ctr						F	0.479	0.60668
CF16 0Cu						F	0.465	0.60668
CF10 ctr						F	0.413	0.60668
CF10 0Cu						F	0.311	0.60668
FITоe citf1 0Cu						F	0.255	0.60668

Levels not connected by same letter are significantly different.

wt 0Cu	CF16 ctr	citf1 0Cu	CITF1oe ctr	CITF1oe 0Cu	FITоe citf1 ctr	CF16 0Cu	CF10 ctr	CF10 0Cu	FITоe citf1 0Cu
16.294	16.384	16.614	16.424	16.525	16.527	16.541	16.593	16.695	16.751
8.499	8.589	8.819	8.630	8.730	8.732	8.746	8.798	8.900	8.956
4.261	4.351	4.581	4.391	4.492	4.494	4.508	4.560	4.662	4.718
1.761	1.851	2.081	1.892	1.992	1.994	2.008	2.060	2.162	2.218
0.002	0.092	0.322	0.133	0.233	0.235	0.249	0.301	0.403	0.459
-2.099	-2.009	-1.779	-1.969	-1.868	-1.866	-1.852	-1.800	-1.698	-1.642
-2.397	-2.307	-2.077	-2.266	-2.166	-2.164	-2.150	-2.098	-1.996	-1.940
-2.923	-2.833	-2.603	-2.793	-2.692	-2.690	-2.676	-2.624	-2.522	-2.466
-3.212	-3.122	-2.891	-3.081	-2.981	-2.979	-2.964	-2.913	-2.811	-2.754
-3.122	-3.212	-2.981	-3.171	-3.070	-3.068	-3.054	-3.003	-2.900	-2.844
-2.891	-2.981	-2.781	-2.986	-2.886	-2.884	-2.870	-2.818	-2.716	-2.660
-3.081	-3.171	-2.986	-3.212	-3.111	-3.109	-3.095	-3.043	-2.941	-2.885
-2.981	-3.070	-2.886	-3.111	-3.212	-3.210	-3.195	-3.144	-3.042	-2.985
-2.979	-3.068	-2.884	-3.109	-3.210	-3.212	-3.197	-3.146	-3.044	-2.987
-2.964	-3.054	-2.870	-3.095	-3.195	-3.197	-3.212	-3.160	-3.058	-3.002
-2.913	-3.003	-2.818	-3.043	-3.144	-3.146	-3.160	-3.212	-3.109	-3.053
-2.811	-2.900	-2.716	-2.941	-3.042	-3.044	-3.058	-3.109	-3.212	-3.155
-2.754	-2.844	-2.660	-2.885	-2.985	-2.987	-3.002	-3.053	-3.155	-3.212

Oneway Analysis of Relative expression By Sample

Means Comparisons

Comparisons for all pairs using Tukey-Kramer HSD

Ordered Differences Report

Level	- Level	Difference	Std Err Dif	Lower CL	Upper CL	p-Value
FIToe citf1 0Fe	FIToe citf1 0Cu	19.96263	0.8579792	16.7511	23.17419	<.0001*
FIToe citf1 0Fe	CF10 0Cu	19.90633	0.8579792	16.6948	23.11789	<.0001*
FIToe citf1 0Fe	CF10 ctr	19.80422	0.8579792	16.5927	23.01578	<.0001*
FIToe citf1 0Fe	CF16 0Cu	19.75261	0.8579792	16.5410	22.96417	<.0001*
FIToe citf1 0Fe	FIToe citf1 ctr	19.73834	0.8579792	16.5268	22.94990	<.0001*
FIToe citf1 0Fe	CITF1oe 0Cu	19.73651	0.8579792	16.5250	22.94807	<.0001*
FIToe citf1 0Fe	CITF1oe ctr	19.63606	0.8579792	16.4245	22.84762	<.0001*
FIToe citf1 0Fe	citf1 0Cu	19.61815	0.8025660	16.6140	22.62229	<.0001*
FIToe citf1 0Fe	CF16 ctr	19.59522	0.8579792	16.3837	22.80678	<.0001*
FIToe citf1 0Fe	wt 0Cu	19.50549	0.8579792	16.2939	22.71705	<.0001*
FIToe citf1 0Fe	wt ctr	19.21715	0.8579792	16.0056	22.42871	<.0001*
FIToe citf1 0Fe	CITF1oe 0Fe	18.69085	0.8579792	15.4793	21.90241	<.0001*
FIToe citf1 0Fe	citf1 ctr	18.39314	0.8579792	15.1816	21.60470	<.0001*
FIToe citf1 0Fe	wt 0Fe	16.29183	0.8579792	13.0803	19.50339	<.0001*
FIToe citf1 0Fe	CF10 0Fe	14.53288	0.8579792	11.3213	17.74444	<.0001*
citf1 0Fe	FIToe citf1 0Cu	12.16771	0.8579792	8.9561	15.37927	<.0001*
citf1 0Fe	CF10 0Cu	12.11141	0.8579792	8.8998	15.32297	<.0001*
FIToe citf1 0Fe	CF16 0Fe	12.03309	0.8579792	8.8215	15.24465	<.0001*
citf1 0Fe	CF10 ctr	12.00929	0.8579792	8.7977	15.22085	<.0001*
citf1 0Fe	CF16 0Cu	11.95768	0.8579792	8.7461	15.16924	<.0001*
citf1 0Fe	FIToe citf1 ctr	11.94342	0.8579792	8.7319	15.15498	<.0001*
citf1 0Fe	CITF1oe 0Cu	11.94159	0.8579792	8.7300	15.15315	<.0001*
citf1 0Fe	CITF1oe ctr	11.84113	0.8579792	8.6296	15.05269	<.0001*
citf1 0Fe	citf1 0Cu	11.82323	0.8025660	8.8191	14.82737	<.0001*
citf1 0Fe	CF16 ctr	11.80029	0.8579792	8.5887	15.01185	<.0001*
citf1 0Fe	wt 0Cu	11.71056	0.8579792	8.4990	14.92212	<.0001*
citf1 0Fe	wt ctr	11.42223	0.8579792	8.2107	14.63379	<.0001*
citf1 0Fe	CITF1oe 0Fe	10.89592	0.8579792	7.6844	14.10748	<.0001*
citf1 0Fe	citf1 ctr	10.59822	0.8579792	7.3867	13.80978	<.0001*
citf1 0Fe	wt 0Fe	8.49691	0.8579792	5.2853	11.70847	<.0001*
CF16 0Fe	FIToe citf1 0Cu	7.92954	0.8579792	4.7180	11.14110	<.0001*
CF16 0Fe	CF10 0Cu	7.87324	0.8579792	4.6617	11.08480	<.0001*
FIToe citf1 0Fe	citf1 0Fe	7.79493	0.8579792	4.5834	11.00649	<.0001*
CF16 0Fe	CF10 ctr	7.77113	0.8579792	4.5596	10.98269	<.0001*
CF16 0Fe	CF16 0Cu	7.71951	0.8579792	4.5080	10.93107	<.0001*
CF16 0Fe	FIToe citf1 ctr	7.70525	0.8579792	4.4937	10.91681	<.0001*
CF16 0Fe	CITF1oe 0Cu	7.70342	0.8579792	4.4919	10.91498	<.0001*
CF16 0Fe	CITF1oe ctr	7.60296	0.8579792	4.3914	10.81452	<.0001*
CF16 0Fe	citf1 0Cu	7.58506	0.8025660	4.5809	10.58920	<.0001*
CF16 0Fe	CF16 ctr	7.56212	0.8579792	4.3506	10.77368	<.0001*
CF16 0Fe	wt 0Cu	7.47239	0.8579792	4.2608	10.68395	<.0001*
CF16 0Fe	wt ctr	7.18406	0.8579792	3.9725	10.39562	<.0001*
citf1 0Fe	CF10 0Fe	6.73796	0.8579792	3.5264	9.94952	<.0001*

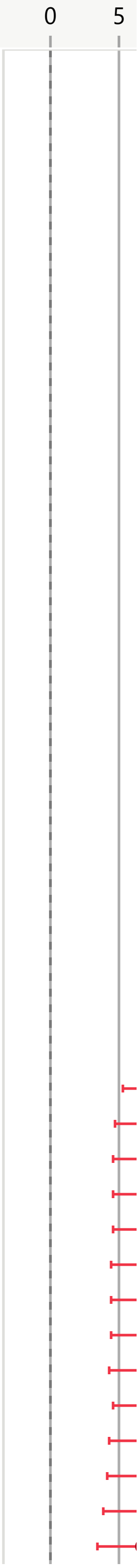

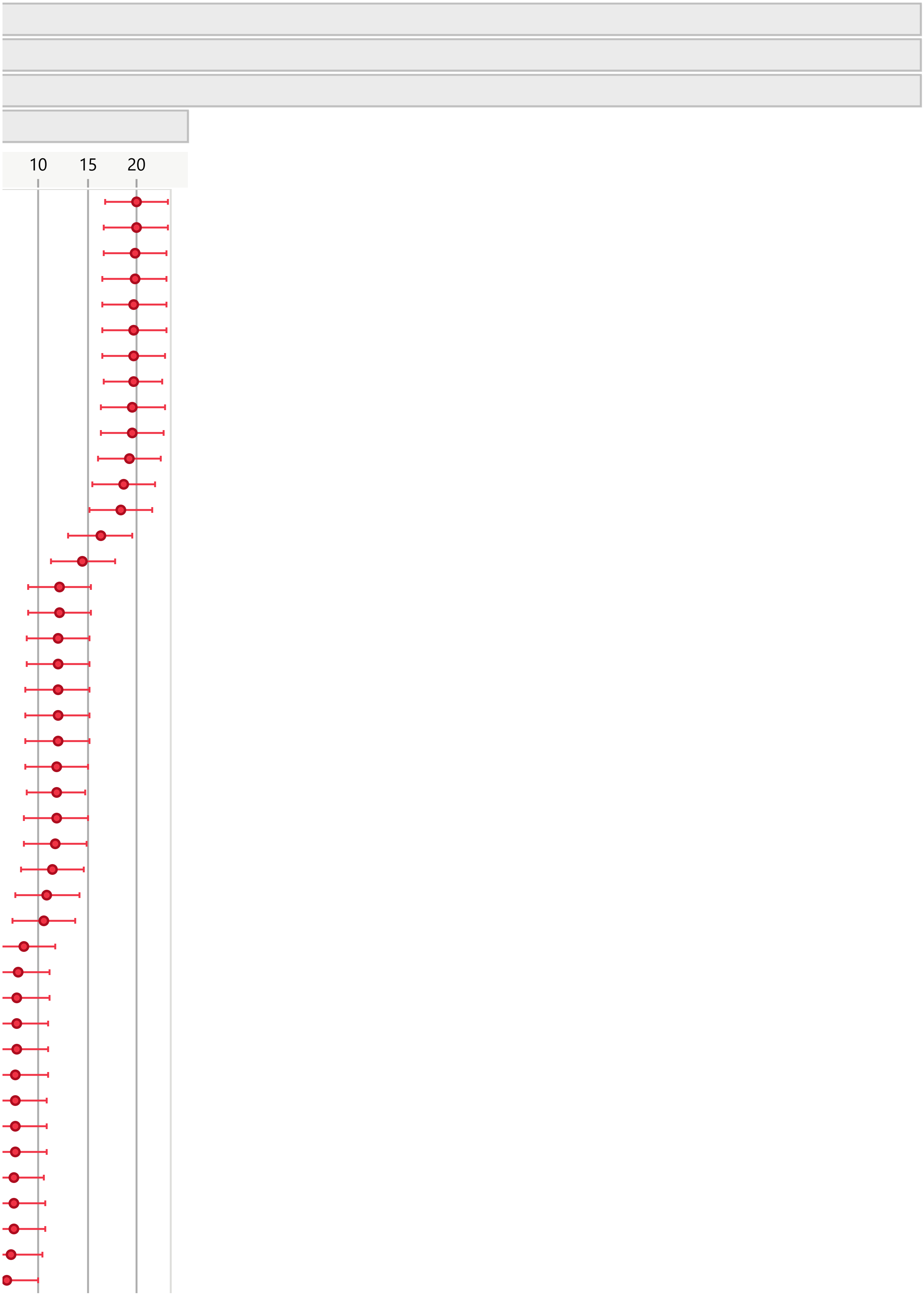
10
15
20

Oneway Analysis of Relative expression By Sample

Means Comparisons

Comparisons for all pairs using Tukey-Kramer HSD

Ordered Differences Report

Level	- Level	Difference	Std Err Dif	Lower CL	Upper CL	p-Value
CF16 0Fe	CITF1oe 0Fe	6.65776	0.8579792	3.4462	9.86932	<.0001*
CF16 0Fe	citf1 ctr	6.36005	0.8579792	3.1485	9.57161	<.0001*
CF10 0Fe	FIToe citf1 0Cu	5.42975	0.8579792	2.2182	8.64131	<.0001*
CF10 0Fe	CF10 0Cu	5.37345	0.8579792	2.1619	8.58501	<.0001*
CF10 0Fe	CF10 ctr	5.27134	0.8579792	2.0598	8.48290	<.0001*
CF10 0Fe	CF16 0Cu	5.21972	0.8579792	2.0082	8.43128	<.0001*
CF10 0Fe	FIToe citf1 ctr	5.20546	0.8579792	1.9939	8.41702	<.0001*
CF10 0Fe	CITF1oe 0Cu	5.20363	0.8579792	1.9921	8.41519	<.0001*
CF10 0Fe	CITF1oe ctr	5.10317	0.8579792	1.8916	8.31473	<.0001*
CF10 0Fe	citf1 0Cu	5.08527	0.8025660	2.0811	8.08941	<.0001*
CF10 0Fe	CF16 ctr	5.06233	0.8579792	1.8508	8.27390	0.0001*
CF10 0Fe	wt 0Cu	4.97260	0.8579792	1.7610	8.18416	0.0001*
CF10 0Fe	wt ctr	4.68427	0.8579792	1.4727	7.89583	0.0004*
CF16 0Fe	wt 0Fe	4.25874	0.8579792	1.0472	7.47030	0.0018*
citf1 0Fe	CF16 0Fe	4.23817	0.8579792	1.0266	7.44973	0.0019*
CF10 0Fe	CITF1oe 0Fe	4.15797	0.8579792	0.9464	7.36953	0.0025*
CF10 0Fe	citf1 ctr	3.86026	0.8579792	0.6487	7.07182	0.0068*
wt 0Fe	FIToe citf1 0Cu	3.67080	0.8579792	0.4592	6.88236	0.0125*
wt 0Fe	CF10 0Cu	3.61450	0.8579792	0.4029	6.82606	0.0149*
wt 0Fe	CF10 ctr	3.51239	0.8579792	0.3008	6.72395	0.0205*
wt 0Fe	CF16 0Cu	3.46077	0.8579792	0.2492	6.67233	0.0240*
wt 0Fe	FIToe citf1 ctr	3.44651	0.8579792	0.2349	6.65807	0.0250*
wt 0Fe	CITF1oe 0Cu	3.44468	0.8579792	0.2331	6.65624	0.0252*
wt 0Fe	CITF1oe ctr	3.34422	0.8579792	0.1327	6.55578	0.0340*
wt 0Fe	citf1 0Cu	3.32632	0.8025660	0.3222	6.33046	0.0179*
wt 0Fe	CF16 ctr	3.30338	0.8579792	0.0918	6.51494	0.0383*
wt 0Fe	wt 0Cu	3.21365	0.8579792	0.0021	6.42521	0.0497*
wt 0Fe	wt ctr	2.92532	0.8579792	-0.2862	6.13688	0.1091
CF16 0Fe	CF10 0Fe	2.49979	0.8579792	-0.7118	5.71135	0.2930
wt 0Fe	CITF1oe 0Fe	2.39902	0.8579792	-0.8125	5.61058	0.3568
wt 0Fe	citf1 ctr	2.10131	0.8579792	-1.1103	5.31287	0.5787
CF10 0Fe	wt 0Fe	1.75895	0.8579792	-1.4526	4.97051	0.8261
citf1 ctr	FIToe citf1 0Cu	1.56949	0.8579792	-1.6421	4.78105	0.9193
citf1 ctr	CF10 0Cu	1.51319	0.8579792	-1.6984	4.72475	0.9389
citf1 ctr	CF10 ctr	1.41108	0.8579792	-1.8005	4.62264	0.9657
citf1 ctr	CF16 0Cu	1.35946	0.8579792	-1.8521	4.57102	0.9753
citf1 ctr	FIToe citf1 ctr	1.34520	0.8579792	-1.8664	4.55676	0.9776
citf1 ctr	CITF1oe 0Cu	1.34337	0.8579792	-1.8682	4.55493	0.9779
CITF1oe 0Fe	FIToe citf1 0Cu	1.27178	0.8579792	-1.9398	4.48334	0.9868
citf1 ctr	CITF1oe ctr	1.24291	0.8579792	-1.9686	4.45447	0.9895
citf1 ctr	citf1 0Cu	1.22501	0.8025660	-1.7791	4.22915	0.9825
CITF1oe 0Fe	CF10 0Cu	1.21548	0.8579792	-1.9961	4.42704	0.9916
citf1 ctr	CF16 ctr	1.20207	0.8579792	-2.0095	4.41363	0.9925

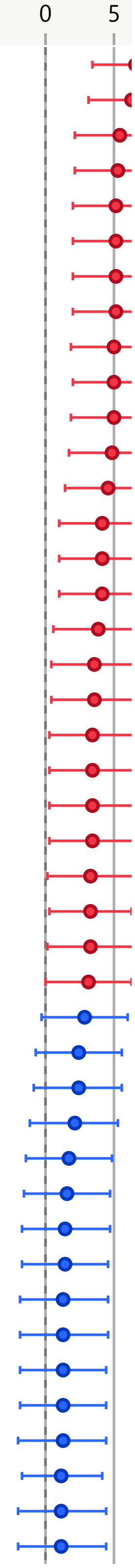

Oneway Analysis of Relative expression By Sample

Means Comparisons

Comparisons for all pairs using Tukey-Kramer HSD

Ordered Differences Report

Level	- Level	Difference	Std Err Dif	Lower CL	Upper CL	p-Value
CITF1oe 0Fe	CF10 ctr	1.11337	0.8579792	-2.0982	4.32493	0.9967
citf1 ctr	wt 0Cu	1.11234	0.8579792	-2.0992	4.32390	0.9967
CITF1oe 0Fe	CF16 0Cu	1.06176	0.8579792	-2.1498	4.27332	0.9981
CITF1oe 0Fe	FIToe citf1 ctr	1.04749	0.8579792	-2.1641	4.25905	0.9984
CITF1oe 0Fe	CITF1oe 0Cu	1.04566	0.8579792	-2.1659	4.25722	0.9984
CITF1oe 0Fe	CITF1oe ctr	0.94521	0.8579792	-2.2664	4.15677	0.9995
CITF1oe 0Fe	citf1 0Cu	0.92730	0.8025660	-2.0768	3.93144	0.9991
CITF1oe 0Fe	CF16 ctr	0.90437	0.8579792	-2.3072	4.11593	0.9997
citf1 ctr	wt ctr	0.82401	0.8579792	-2.3876	4.03557	0.9999
CITF1oe 0Fe	wt 0Cu	0.81464	0.8579792	-2.3969	4.02620	0.9999
wt ctr	FIToe citf1 0Cu	0.74548	0.8579792	-2.4661	3.95704	1.0000
wt ctr	CF10 0Cu	0.68918	0.8579792	-2.5224	3.90074	1.0000
wt ctr	CF10 ctr	0.58707	0.8579792	-2.6245	3.79863	1.0000
wt ctr	CF16 0Cu	0.53546	0.8579792	-2.6761	3.74702	1.0000
CITF1oe 0Fe	wt ctr	0.52630	0.8579792	-2.6853	3.73786	1.0000
wt ctr	FIToe citf1 ctr	0.52119	0.8579792	-2.6904	3.73275	1.0000
wt ctr	CITF1oe 0Cu	0.51936	0.8579792	-2.6922	3.73092	1.0000
wt 0Cu	FIToe citf1 0Cu	0.45715	0.8579792	-2.7544	3.66871	1.0000
wt ctr	CITF1oe ctr	0.41891	0.8579792	-2.7927	3.63047	1.0000
wt ctr	citf1 0Cu	0.40100	0.8025660	-2.6031	3.40514	1.0000
wt 0Cu	CF10 0Cu	0.40084	0.8579792	-2.8107	3.61240	1.0000
wt ctr	CF16 ctr	0.37807	0.8579792	-2.8335	3.58963	1.0000
CF16 ctr	FIToe citf1 0Cu	0.36742	0.8579792	-2.8441	3.57898	1.0000
citf1 0Cu	FIToe citf1 0Cu	0.34448	0.8025660	-2.6597	3.34862	1.0000
CITF1oe ctr	FIToe citf1 0Cu	0.32658	0.8579792	-2.8850	3.53814	1.0000
CF16 ctr	CF10 0Cu	0.31111	0.8579792	-2.9004	3.52267	1.0000
wt 0Cu	CF10 ctr	0.29873	0.8579792	-2.9128	3.51029	1.0000
citf1 ctr	CITF1oe 0Fe	0.29771	0.8579792	-2.9139	3.50927	1.0000
wt ctr	wt 0Cu	0.28834	0.8579792	-2.9232	3.49990	1.0000
citf1 0Cu	CF10 0Cu	0.28818	0.8025660	-2.7160	3.29232	1.0000
CITF1oe ctr	CF10 0Cu	0.27027	0.8579792	-2.9413	3.48183	1.0000
wt 0Cu	CF16 0Cu	0.24712	0.8579792	-2.9644	3.45868	1.0000
wt 0Cu	FIToe citf1 ctr	0.23286	0.8579792	-2.9787	3.44442	1.0000
wt 0Cu	CITF1oe 0Cu	0.23103	0.8579792	-2.9805	3.44259	1.0000
CITF1oe 0Cu	FIToe citf1 0Cu	0.22612	0.8579792	-2.9854	3.43768	1.0000
FIToe citf1 ctr	FIToe citf1 0Cu	0.22429	0.8579792	-2.9873	3.43585	1.0000
CF16 0Cu	FIToe citf1 0Cu	0.21003	0.8579792	-3.0015	3.42159	1.0000
CF16 ctr	CF10 ctr	0.20900	0.8579792	-3.0026	3.42056	1.0000
citf1 0Cu	CF10 ctr	0.18607	0.8025660	-2.8181	3.19021	1.0000
CITF1oe 0Cu	CF10 0Cu	0.16982	0.8579792	-3.0417	3.38138	1.0000
CITF1oe ctr	CF10 ctr	0.16816	0.8579792	-3.0434	3.37972	1.0000
FIToe citf1 ctr	CF10 0Cu	0.16799	0.8579792	-3.0436	3.37955	1.0000
CF10 ctr	FIToe citf1 0Cu	0.15841	0.8579792	-3.0531	3.36997	1.0000

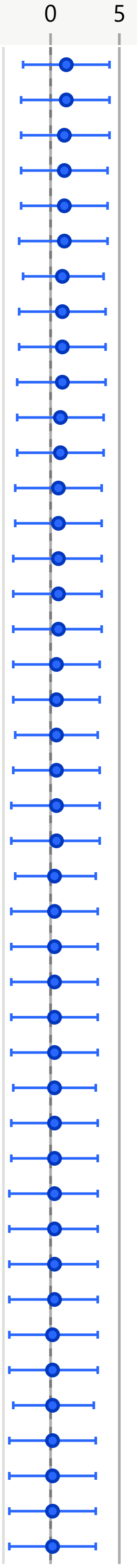

Oneway Analysis of Relative expression By Sample

Means Comparisons

Comparisons for all pairs using Tukey-Kramer HSD

Ordered Differences Report

Level	- Level	Difference	Std Err Dif	Lower CL	Upper CL	p-Value
CF16 ctr	CF16 0Cu	0.15739	0.8579792	-3.0542	3.36895	1.0000
CF16 0Cu	CF10 0Cu	0.15372	0.8579792	-3.0578	3.36528	1.0000
CF16 ctr	FITоe citf1 ctr	0.14313	0.8579792	-3.0684	3.35469	1.0000
CF16 ctr	CITF1oe 0Cu	0.14129	0.8579792	-3.0703	3.35285	1.0000
citf1 0Cu	CF16 0Cu	0.13445	0.8025660	-2.8697	3.13859	1.0000
wt 0Cu	CITF1oe ctr	0.13057	0.8579792	-3.0810	3.34213	1.0000
citf1 0Cu	FIToe citf1 ctr	0.12019	0.8025660	-2.8839	3.12433	1.0000
citf1 0Cu	CITF1oe 0Cu	0.11836	0.8025660	-2.8858	3.12250	1.0000
CITF1oe ctr	CF16 0Cu	0.11655	0.8579792	-3.0950	3.32811	1.0000
wt 0Cu	citf1 0Cu	0.11267	0.8025660	-2.8915	3.11680	1.0000
CITF1oe ctr	FIToe citf1 ctr	0.10229	0.8579792	-3.1093	3.31385	1.0000
CF10 ctr	CF10 0Cu	0.10211	0.8579792	-3.1094	3.31367	1.0000
CITF1oe ctr	CITF1oe 0Cu	0.10046	0.8579792	-3.1111	3.31202	1.0000
wt 0Cu	CF16 ctr	0.08973	0.8579792	-3.1218	3.30129	1.0000
CITF1oe 0Cu	CF10 ctr	0.06771	0.8579792	-3.1439	3.27927	1.0000
FIToe citf1 ctr	CF10 ctr	0.06588	0.8579792	-3.1457	3.27744	1.0000
CF10 0Cu	FIToe citf1 0Cu	0.05630	0.8579792	-3.1553	3.26786	1.0000
CF16 0Cu	CF10 ctr	0.05161	0.8579792	-3.1599	3.26317	1.0000
CF16 ctr	CITF1oe ctr	0.04084	0.8579792	-3.1707	3.25240	1.0000
CF16 ctr	citf1 0Cu	0.02294	0.8025660	-2.9812	3.02707	1.0000
citf1 0Cu	CITF1oe ctr	0.01790	0.8025660	-2.9862	3.02204	1.0000
CITF1oe 0Cu	CF16 0Cu	0.01609	0.8579792	-3.1955	3.22765	1.0000
FIToe citf1 ctr	CF16 0Cu	0.01426	0.8579792	-3.1973	3.22582	1.0000
CITF1oe 0Cu	FIToe citf1 ctr	0.00183	0.8579792	-3.2097	3.21339	1.0000

Missing Rows 89

Oneway Analysis of FRO2 By Sample

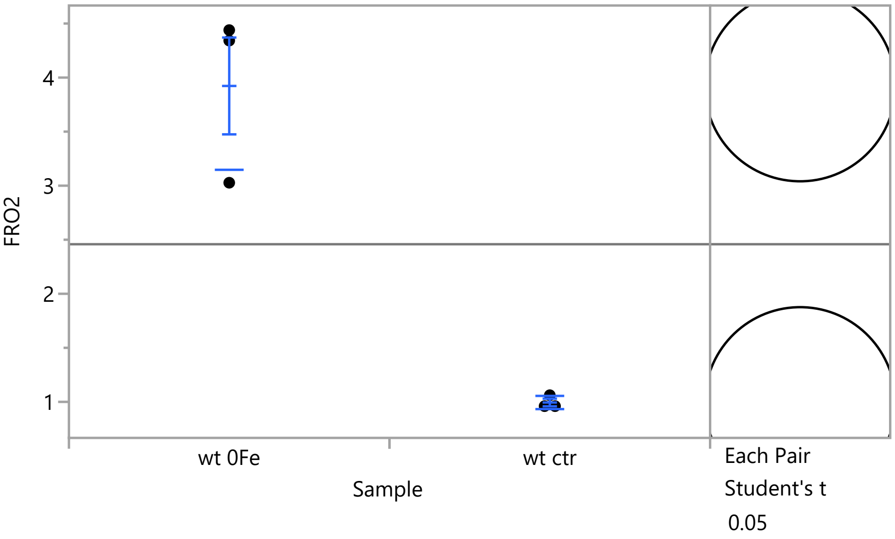

Means and Std Deviations

Level	Number	Mean	Std Dev	Std Err Mean	Lower 95%	Upper 95%
wt 0Fe	3	3.9253167	0.7748523	0.4473612	2.0004769	5.8501565
wt ctr	3	1.0000002	0.061054	0.0352496	0.8483336	1.1516669

Means Comparisons

Comparisons for each pair using Student's t

Confidence Quantile

t	Alpha
2.77645	0.05

LSD Threshold Matrix

Abs(Dif)-LSD

	wt 0Fe	wt ctr
wt 0Fe	-1.2459	1.6794
wt ctr	1.6794	-1.2459

Positive values show pairs of means that are significantly different.

Connecting Letters Report

Level			Mean	Std Error
wt 0Fe	A		3.9253	0.31731
wt ctr		B	1.0000	0.31731

Levels not connected by same letter are significantly different.

Ordered Differences Report

Level	- Level	Difference	Std Err Dif	Lower CL	Upper CL	p-Value
wt 0Fe	wt ctr	2.925316	0.4487478	1.679393	4.171240	0.0029*

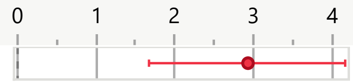

Oneway Analysis of length By Name

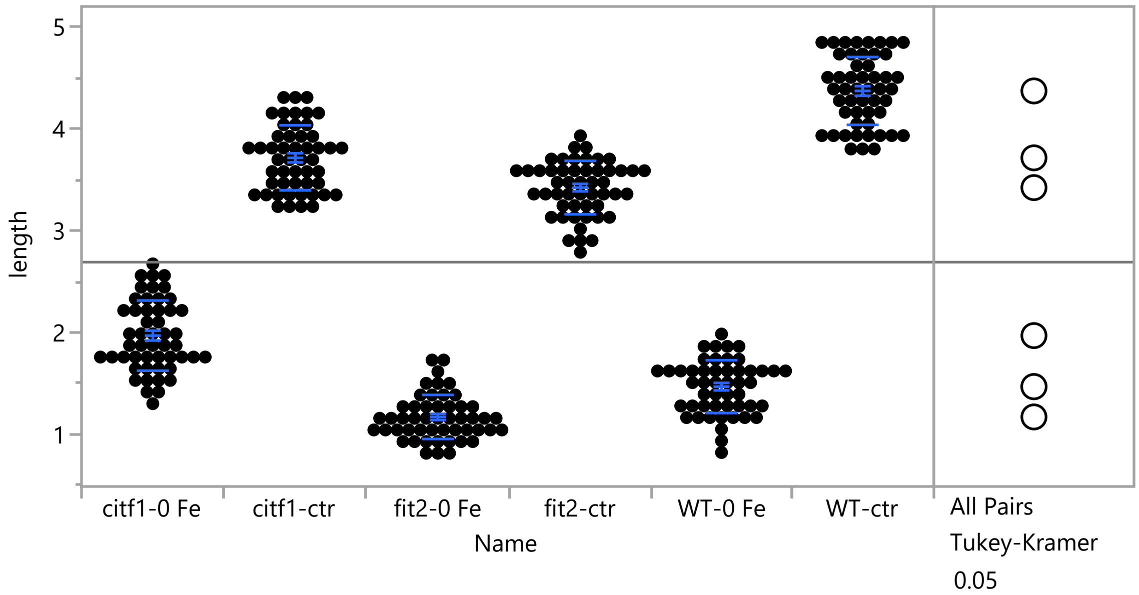

Means and Std Deviations

Level	Number	Mean	Std Dev	Std Err Mean	Lower 95%	Upper 95%
citf1-0 Fe	50	1.9698576	0.345015	0.0487925	1.8718055	2.0679098
citf1-ctr	50	3.7178819	0.3198056	0.0452273	3.6269942	3.8087697
fit2-0 Fe	50	1.1700928	0.2166133	0.0306337	1.108532	1.2316536
fit2-ctr	50	3.4252524	0.263167	0.0372174	3.3504612	3.5000437
WT-0 Fe	49	1.4680706	0.2595763	0.0370823	1.3935116	1.5426296
WT-ctr	50	4.3758332	0.3335384	0.0471695	4.2810426	4.4706238

Oneway Analysis of length By Name

Means Comparisons

Comparisons for all pairs using Tukey-Kramer HSD

Confidence Quantile

q*	Alpha
2.86868	0.05

HSD Threshold Matrix

Abs(Dif)-HSD

	WT-ctr	citf1-ctr	fit2-ctr	citf1-0 Fe	WT-0 Fe	fit2-0 Fe
WT-ctr	-0.1683	0.4896	0.7822	2.2376	2.7386	3.0374
citf1-ctr	0.4896	-0.1683	0.1243	1.5797	2.0806	2.3795
fit2-ctr	0.7822	0.1243	-0.1683	1.2871	1.7880	2.0868
citf1-0 Fe	2.2376	1.5797	1.2871	-0.1683	0.3326	0.6314
WT-0 Fe	2.7386	2.0806	1.7880	0.3326	-0.1700	0.1288
fit2-0 Fe	3.0374	2.3795	2.0868	0.6314	0.1288	-0.1683

Positive values show pairs of means that are significantly different.

Connecting Letters Report

Level							Mean	Std Error
WT-ctr	A						4.3758	0.04149
citf1-ctr		B					3.7179	0.04149
fit2-ctr			C				3.4253	0.04149
citf1-0 Fe				D			1.9699	0.04149
WT-0 Fe					E		1.4681	0.04191
fit2-0 Fe						F	1.1701	0.04149

Levels not connected by same letter are significantly different.

Ordered Differences Report

Level	- Level	Difference	Std Err Dif	Lower CL	Upper CL	p-Value
WT-ctr	fit2-0 Fe	3.205740	0.0586798	3.037407	3.374074	<.0001*
WT-ctr	WT-0 Fe	2.907763	0.0589785	2.738572	3.076953	<.0001*
citf1-ctr	fit2-0 Fe	2.547789	0.0586798	2.379455	2.716123	<.0001*
WT-ctr	citf1-0 Fe	2.405976	0.0586798	2.237642	2.574309	<.0001*
fit2-ctr	fit2-0 Fe	2.255160	0.0586798	2.086826	2.423494	<.0001*
citf1-ctr	WT-0 Fe	2.249811	0.0589785	2.080621	2.419002	<.0001*
fit2-ctr	WT-0 Fe	1.957182	0.0589785	1.787991	2.126372	<.0001*
citf1-ctr	citf1-0 Fe	1.748024	0.0586798	1.579690	1.916358	<.0001*
fit2-ctr	citf1-0 Fe	1.455395	0.0586798	1.287061	1.623729	<.0001*
WT-ctr	fit2-ctr	0.950581	0.0586798	0.782247	1.118915	<.0001*
citf1-0 Fe	fit2-0 Fe	0.799765	0.0586798	0.631431	0.968099	<.0001*
WT-ctr	citf1-ctr	0.657951	0.0586798	0.489617	0.826285	<.0001*
citf1-0 Fe	WT-0 Fe	0.501787	0.0589785	0.332596	0.670978	<.0001*
WT-0 Fe	fit2-0 Fe	0.297978	0.0589785	0.128787	0.467168	<.0001*
citf1-ctr	fit2-ctr	0.292630	0.0586798	0.124296	0.460963	<.0001*

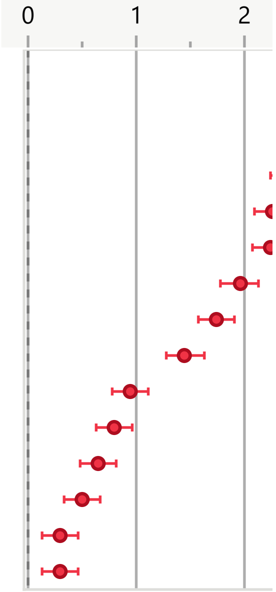

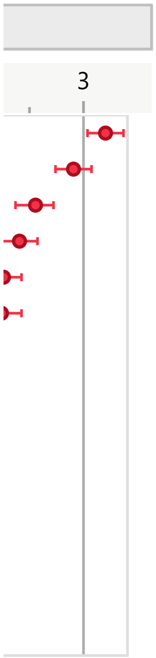
3

Oneway Analysis of length By name

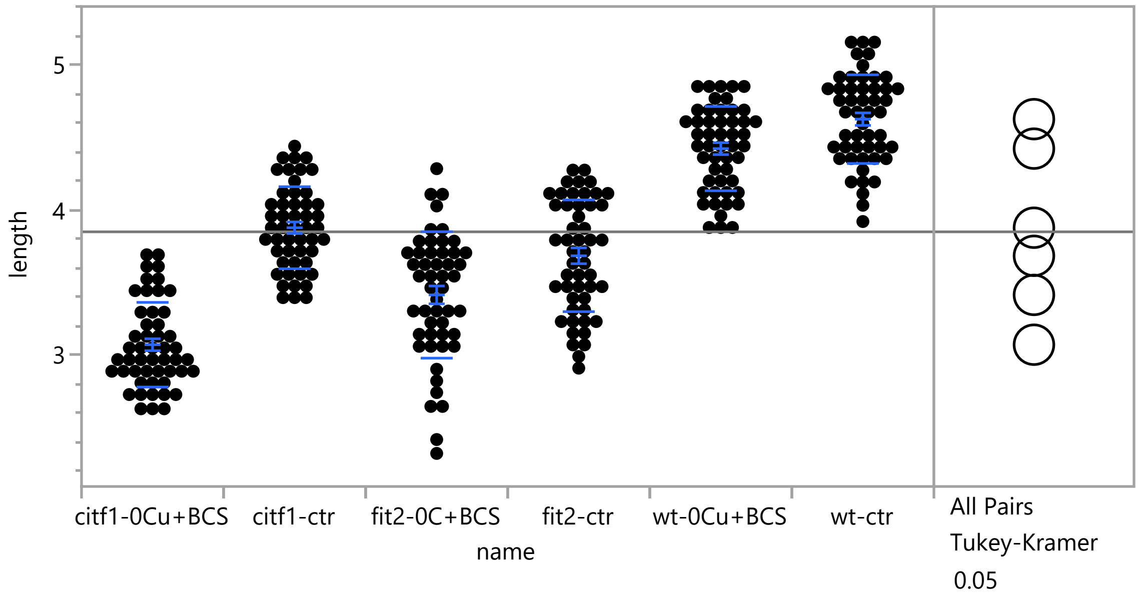

Means and Std Deviations

Level	Number	Mean	Std Dev	Std Err Mean	Lower 95%	Upper 95%
citf1-0Cu+BCS	50	3.0691949	0.2916616	0.0412472	2.9863056	3.1520842
citf1-ctr	50	3.8754761	0.2826192	0.0399684	3.7951567	3.9557956
fit2-0C+BCS	50	3.4124593	0.4356926	0.0616162	3.2886368	3.5362818
fit2-ctr	50	3.6820089	0.3849951	0.0544465	3.5725945	3.7914233
wt-0Cu+BCS	50	4.4205127	0.2903862	0.0410668	4.3379858	4.5030395
wt-ctr	50	4.6236761	0.305593	0.0432174	4.5368275	4.7105246

Oneway Analysis of length By name

Means Comparisons

Comparisons for all pairs using Tukey-Kramer HSD

Confidence Quantile

q*	Alpha
2.86862	0.05

HSD Threshold Matrix

Abs(Dif)-HSD

	wt-ctr	wt-0Cu+BCS	citf1-ctr	fit2-ctr	fit2-0C+BCS	citf1-0Cu+BCS
wt-ctr	-0.1932	0.0099	0.5550	0.7484	1.0180	1.3612
wt-0Cu+BCS	0.0099	-0.1932	0.3518	0.5453	0.8148	1.1581
citf1-ctr	0.5550	0.3518	-0.1932	0.0002	0.2698	0.6130
fit2-ctr	0.7484	0.5453	0.0002	-0.1932	0.0763	0.4196
fit2-0C+BCS	1.0180	0.8148	0.2698	0.0763	-0.1932	0.1500
citf1-0Cu+BCS	1.3612	1.1581	0.6130	0.4196	0.1500	-0.1932

Positive values show pairs of means that are significantly different.

Connecting Letters Report

Level							Mean	Std Error
wt-ctr	A						4.6237	0.04763
wt-0Cu+BCS		B					4.4205	0.04763
citf1-ctr			C				3.8755	0.04763
fit2-ctr				D			3.6820	0.04763
fit2-0C+BCS					E		3.4125	0.04763
citf1-0Cu+BCS						F	3.0692	0.04763

Levels not connected by same letter are significantly different.

Ordered Differences Report

Level	- Level	Difference	Std Err Dif	Lower CL	Upper CL	p-Value
wt-ctr	citf1-0Cu+BCS	1.554481	0.0673647	1.361237	1.747725	<.0001*
wt-0Cu+BCS	citf1-0Cu+BCS	1.351318	0.0673647	1.158074	1.544561	<.0001*
wt-ctr	fit2-0C+BCS	1.211217	0.0673647	1.017973	1.404460	<.0001*
wt-0Cu+BCS	fit2-0C+BCS	1.008053	0.0673647	0.814810	1.201297	<.0001*
wt-ctr	fit2-ctr	0.941667	0.0673647	0.748424	1.134911	<.0001*
citf1-ctr	citf1-0Cu+BCS	0.806281	0.0673647	0.613038	0.999525	<.0001*
wt-ctr	citf1-ctr	0.748200	0.0673647	0.554956	0.941444	<.0001*
wt-0Cu+BCS	fit2-ctr	0.738504	0.0673647	0.545260	0.931747	<.0001*
fit2-ctr	citf1-0Cu+BCS	0.612814	0.0673647	0.419570	0.806058	<.0001*
wt-0Cu+BCS	citf1-ctr	0.545037	0.0673647	0.351793	0.738280	<.0001*
citf1-ctr	fit2-0C+BCS	0.463017	0.0673647	0.269773	0.656260	<.0001*
fit2-0C+BCS	citf1-0Cu+BCS	0.343264	0.0673647	0.150021	0.536508	<.0001*
fit2-ctr	fit2-0C+BCS	0.269550	0.0673647	0.076306	0.462793	0.0011*
wt-ctr	wt-0Cu+BCS	0.203163	0.0673647	0.009920	0.396407	0.0329*
citf1-ctr	fit2-ctr	0.193467	0.0673647	0.000224	0.386711	0.0495*

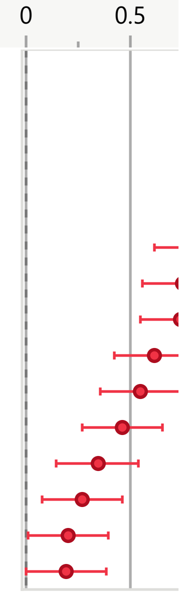

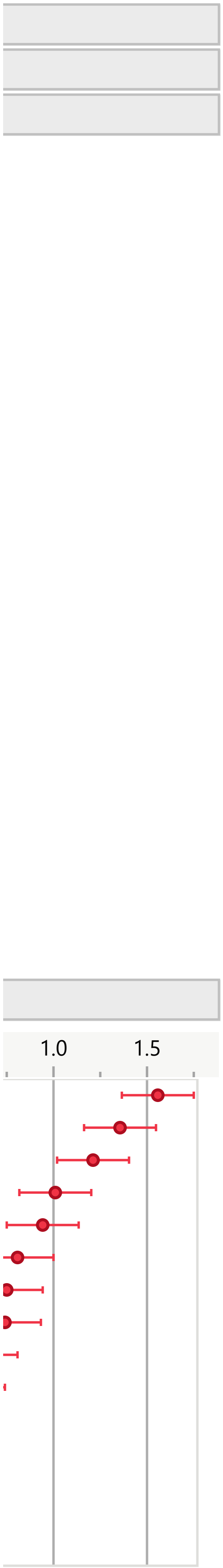
1.0
1.5

Oneway Analysis of Ctr root length By sample

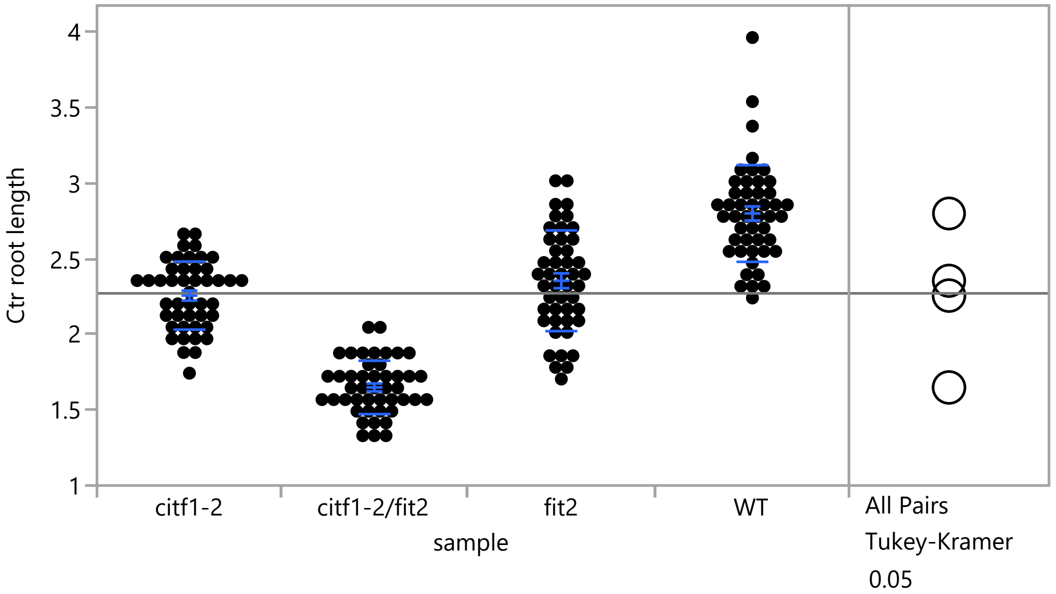

Means and Std Deviations

Level	Number	Mean	Std Dev	Std Err Mean	Lower 95%	Upper 95%
citf1-2	45	2.2559721	0.2249352	0.0335314	2.188394	2.3235501
citf1-2/fit2	45	1.648542	0.1764455	0.0263029	1.5955319	1.7015521
fit2	46	2.354024	0.3330505	0.0491056	2.2551202	2.4529278
WT	47	2.7990215	0.3198526	0.0466553	2.7051093	2.8929338

Oneway Analysis of Ctr root length By sample

Means Comparisons

Comparisons for all pairs using Tukey-Kramer HSD

Confidence Quantile

q*	Alpha
2.59334	0.05

HSD Threshold Matrix

Abs(Dif)-HSD

	WT	fit2	citf1-2	citf1-2/fit2
WT	-0.1458	0.2984	0.3957	1.0031
fit2	0.2984	-0.1474	-0.0501	0.5573
citf1-2	0.3957	-0.0501	-0.1490	0.4584
citf1-2/fit2	1.0031	0.5573	0.4584	-0.1490

Positive values show pairs of means that are significantly different.

Connecting Letters Report

Level				Mean	Std Error
WT	A			2.7990	0.03975
fit2		B		2.3540	0.04018
citf1-2		B		2.2560	0.04062
citf1-2/fit2			C	1.6485	0.04062

Levels not connected by same letter are significantly different.

Ordered Differences Report

Level	- Level	Difference	Std Err Dif	Lower CL	Upper CL	p-Value
WT	citf1-2/fit2	1.150480	0.0568374	1.00308	1.297878	<.0001*
fit2	citf1-2/fit2	0.705482	0.0571388	0.55730	0.853662	<.0001*
citf1-2	citf1-2/fit2	0.607430	0.0574519	0.45844	0.756423	<.0001*
WT	citf1-2	0.543049	0.0568374	0.39565	0.690448	<.0001*
WT	fit2	0.444998	0.0565209	0.29842	0.591576	<.0001*
fit2	citf1-2	0.098052	0.0571388	-0.05013	0.246232	0.3184

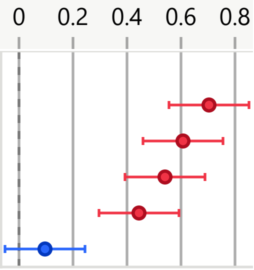

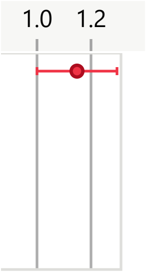
1.0
1.2

Oneway Analysis of 0Cu root length By sample

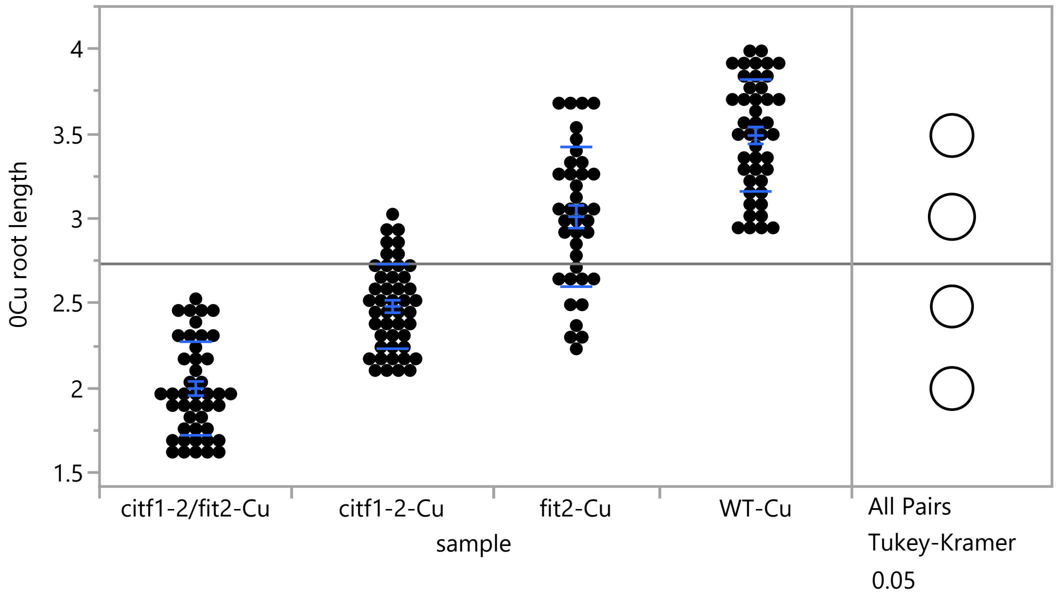

Means and Std Deviations

Level	Number	Mean	Std Dev	Std Err Mean	Lower 95%	Upper 95%
citf1-2/fit2-Cu	44	1.9972652	0.2757745	0.0415746	1.9134221	2.0811084
citf1-2-Cu	46	2.4810989	0.2491191	0.0367306	2.4071196	2.5550781
fit2-Cu	38	3.0098374	0.4117937	0.0668018	2.8744842	3.1451906
WT-Cu	44	3.4893562	0.3299194	0.0497372	3.3890515	3.5896608

Oneway Analysis of 0Cu root length By sample

Means Comparisons

Comparisons for all pairs using Tukey-Kramer HSD

Confidence Quantile

q*	Alpha
2.59494	0.05

HSD Threshold Matrix

Abs(Dif)-HSD

	WT-Cu	fit2-Cu	citf1-2-Cu	citf1-2/fit2-Cu
WT-Cu	-0.1761	0.2966	0.8341	1.3160
fit2-Cu	0.2966	-0.1895	0.3477	0.8297
citf1-2-Cu	0.8341	0.3477	-0.1722	0.3097
citf1-2/fit2-Cu	1.3160	0.8297	0.3097	-0.1761

Positive values show pairs of means that are significantly different.

Connecting Letters Report

Level					Mean	Std Error
WT-Cu	A				3.4894	0.04798
fit2-Cu		B			3.0098	0.05163
citf1-2-Cu			C		2.4811	0.04693
citf1-2/fit2-Cu				D	1.9973	0.04798

Levels not connected by same letter are significantly different.

Ordered Differences Report

Level	- Level	Difference	Std Err Dif	Lower CL	Upper CL	p-Value
WT-Cu	citf1-2/fit2-Cu	1.492091	0.0678552	1.316011	1.668171	<.0001*
fit2-Cu	citf1-2/fit2-Cu	1.012572	0.0704828	0.829673	1.195471	<.0001*
WT-Cu	citf1-2-Cu	1.008257	0.0671136	0.834101	1.182413	<.0001*
fit2-Cu	citf1-2-Cu	0.528739	0.0697691	0.347692	0.709785	<.0001*
citf1-2-Cu	citf1-2/fit2-Cu	0.483834	0.0671136	0.309678	0.657990	<.0001*
WT-Cu	fit2-Cu	0.479519	0.0704828	0.296620	0.662418	<.0001*

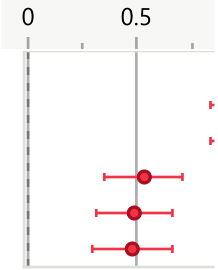

Missing Rows 182

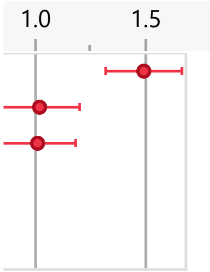
1.0
1.5

Oneway Analysis of 0Fe root length By sample

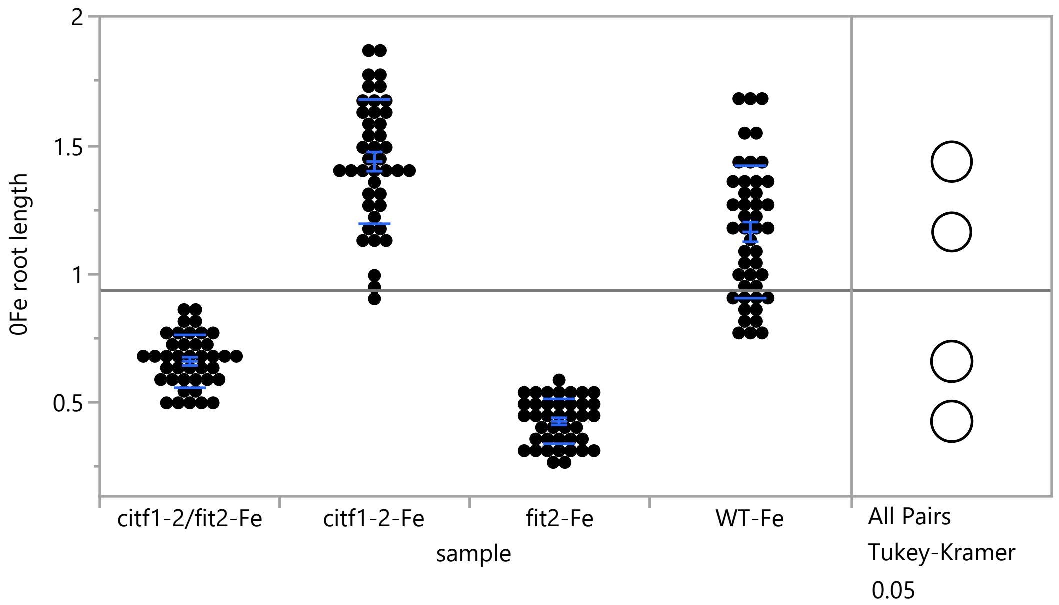

Means and Std Deviations

Level	Number	Mean	Std Dev	Std Err Mean	Lower 95%	Upper 95%
citf1-2/fit2-Fe	40	0.6600565	0.1026956	0.0162376	0.6272129	0.6929002
citf1-2-Fe	42	1.4364542	0.2415198	0.0372673	1.3611913	1.5117171
fit2-Fe	40	0.4268896	0.0870626	0.0137658	0.3990456	0.4547335
WT-Fe	45	1.1630988	0.2574088	0.0383722	1.0857647	1.240433

Oneway Analysis of 0Fe root length By sample

Means Comparisons

Comparisons for all pairs using Tukey-Kramer HSD

Confidence Quantile

q*	Alpha
2.59574	0.05

HSD Threshold Matrix

Abs(Dif)-HSD

	citf1-2-Fe	WT-Fe	citf1-2/fit2-Fe	fit2-Fe
citf1-2-Fe	-0.10880	0.16638	0.66624	0.89941
WT-Fe	0.16638	-0.10511	0.39469	0.62786
citf1-2/fit2-Fe	0.66624	0.39469	-0.11149	0.12168
fit2-Fe	0.89941	0.62786	0.12168	-0.11149

Positive values show pairs of means that are significantly different.

Connecting Letters Report

Level					Mean	Std Error
citf1-2-Fe	A				1.4365	0.02964
WT-Fe		B			1.1631	0.02863
citf1-2/fit2-Fe			C		0.6601	0.03037
fit2-Fe				D	0.4269	0.03037

Levels not connected by same letter are significantly different.

Ordered Differences Report

Level	- Level	Difference	Std Err Dif	Lower CL	Upper CL	p-Value
citf1-2-Fe	fit2-Fe	1.009565	0.0424363	0.8994108	1.119718	<.0001*
citf1-2-Fe	citf1-2/fit2-Fe	0.776398	0.0424363	0.6662438	0.886552	<.0001*
WT-Fe	fit2-Fe	0.736209	0.0417406	0.6278613	0.844557	<.0001*
WT-Fe	citf1-2/fit2-Fe	0.503042	0.0417406	0.3946944	0.611390	<.0001*
citf1-2-Fe	WT-Fe	0.273355	0.0412111	0.1663819	0.380329	<.0001*
citf1-2/fit2-Fe	fit2-Fe	0.233167	0.0429507	0.1216779	0.344656	<.0001*

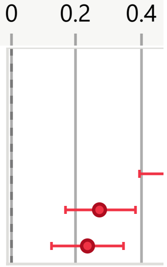

Missing Rows 182

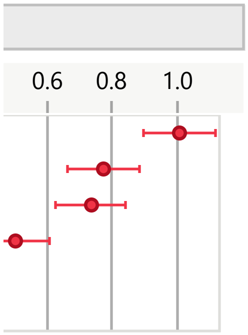
0.6
0.8
1.0

Oneway Analysis of Fe in roots By Treatment

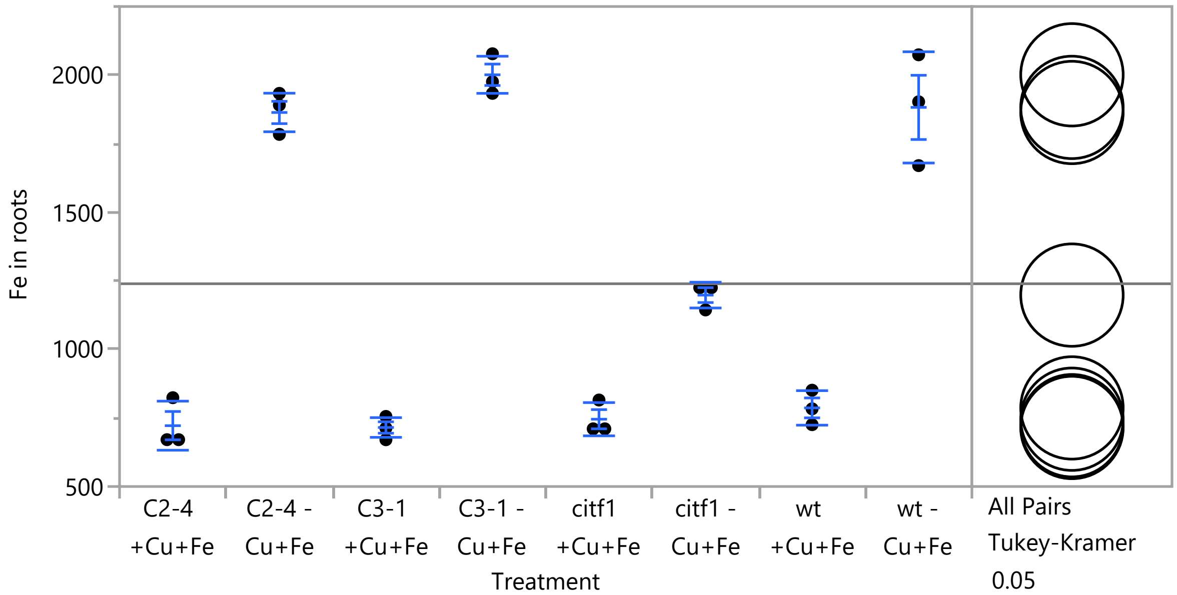

Means and Std Deviations

Level	Number	Mean	Std Dev	Std Err Mean	Lower 95%	Upper 95%
C2-4 +Cu+Fe	3	721.76255	89.458039	51.648623	499.53646	943.98864
C2-4 -Cu+Fe	3	1863.5998	70.306981	40.591754	1688.9476	2038.2521
C3-1 +Cu+Fe	3	715.13313	35.945846	20.753344	625.8387	804.42756
C3-1 -Cu+Fe	3	2001.0198	67.660193	39.063631	1832.9426	2169.097
citf1 +Cu+Fe	3	745.32575	60.607884	34.991978	594.76742	895.88408
citf1 -Cu+Fe	3	1197.836	46.985131	27.126878	1081.1184	1314.5535
wt +Cu+Fe	3	786.54826	62.883075	36.30556	630.33804	942.75848
wt -Cu+Fe	3	1882.1623	202.7718	117.07035	1378.4492	2385.8754

Means Comparisons

Comparisons for all pairs using Tukey-Kramer HSD

Confidence Quantile

q*	Alpha
3.46215	0.05

HSD Threshold Matrix

Abs(Dif)-HSD

	C3-1 -Cu+Fe	wt -Cu+Fe	C2-4 -Cu+Fe	citf1 -Cu+Fe	wt +Cu+Fe	citf1 +Cu+Fe	C2-4 +Cu+Fe	C3-1 +Cu[illegible]
C3-1 -Cu+Fe	-264.0	-145.1	-126.6	539.2	950.5	991.7	1015.3	10[illegible]
wt -Cu+Fe	-145.1	-264.0	-245.4	420.3	831.6	872.9	896.4	9[illegible]
C2-4 -Cu+Fe	-126.6	-245.4	-264.0	401.8	813.1	854.3	877.9	8[illegible]
citf1 -Cu+Fe	539.2	420.3	401.8	-264.0	147.3	188.5	212.1	2[illegible]
wt +Cu+Fe	950.5	831.6	813.1	147.3	-264.0	-222.8	-199.2	-1[illegible]
citf1 +Cu+Fe	991.7	872.9	854.3	188.5	-222.8	-264.0	-240.4	-2[illegible]
C2-4 +Cu+Fe	1015.3	896.4	877.9	212.1	-199.2	-240.4	-264.0	-2[illegible]
C3-1 +Cu+Fe	1021.9	903.0	884.5	218.7	-192.6	-233.8	-257.4	-2[illegible]

Positive values show pairs of means that are significantly different.

[illegible]+Fe
[illegible]21.9
[illegible]03.0
[illegible]84.5
[illegible]18.7
[illegible]92.6
[illegible]33.8
[illegible]57.4
[illegible]64.0

Oneway Analysis of Fe in roots By Treatment

Means Comparisons

Comparisons for all pairs using Tukey-Kramer HSD

Connecting Letters Report

Level				Mean	Std Error
C3-1 -Cu+Fe	A			2001.0	53.916
wt -Cu+Fe	A			1882.2	53.916
C2-4 -Cu+Fe	A			1863.6	53.916
citf1 -Cu+Fe		B		1197.8	53.916
wt +Cu+Fe			C	786.5	53.916
citf1 +Cu+Fe			C	745.3	53.916
C2-4 +Cu+Fe			C	721.8	53.916
C3-1 +Cu+Fe			C	715.1	53.916

Levels not connected by same letter are significantly different.

Ordered Differences Report

Level	- Level	Difference	Std Err Dif	Lower CL	Upper CL	p-Value
C3-1 -Cu+Fe	C3-1 +Cu+Fe	1285.887	76.24927	1021.90	1549.873	<.0001*
C3-1 -Cu+Fe	C2-4 +Cu+Fe	1279.257	76.24927	1015.27	1543.244	<.0001*
C3-1 -Cu+Fe	citf1 +Cu+Fe	1255.694	76.24927	991.71	1519.681	<.0001*
C3-1 -Cu+Fe	wt +Cu+Fe	1214.472	76.24927	950.49	1478.458	<.0001*
wt -Cu+Fe	C3-1 +Cu+Fe	1167.029	76.24927	903.04	1431.016	<.0001*
wt -Cu+Fe	C2-4 +Cu+Fe	1160.400	76.24927	896.41	1424.386	<.0001*
C2-4 -Cu+Fe	C3-1 +Cu+Fe	1148.467	76.24927	884.48	1412.453	<.0001*
C2-4 -Cu+Fe	C2-4 +Cu+Fe	1141.837	76.24927	877.85	1405.824	<.0001*
wt -Cu+Fe	citf1 +Cu+Fe	1136.837	76.24927	872.85	1400.823	<.0001*
C2-4 -Cu+Fe	citf1 +Cu+Fe	1118.274	76.24927	854.29	1382.261	<.0001*
wt -Cu+Fe	wt +Cu+Fe	1095.614	76.24927	831.63	1359.601	<.0001*
C2-4 -Cu+Fe	wt +Cu+Fe	1077.052	76.24927	813.07	1341.038	<.0001*
C3-1 -Cu+Fe	citf1 -Cu+Fe	803.184	76.24927	539.20	1067.170	<.0001*
wt -Cu+Fe	citf1 -Cu+Fe	684.326	76.24927	420.34	948.313	<.0001*
C2-4 -Cu+Fe	citf1 -Cu+Fe	665.764	76.24927	401.78	929.750	<.0001*
citf1 -Cu+Fe	C3-1 +Cu+Fe	482.703	76.24927	218.72	746.689	0.0002*
citf1 -Cu+Fe	C2-4 +Cu+Fe	476.073	76.24927	212.09	740.060	0.0002*
citf1 -Cu+Fe	citf1 +Cu+Fe	452.510	76.24927	188.52	716.497	0.0004*
citf1 -Cu+Fe	wt +Cu+Fe	411.288	76.24927	147.30	675.274	0.0012*
C3-1 -Cu+Fe	C2-4 -Cu+Fe	137.420	76.24927	-126.57	401.406	0.6274
C3-1 -Cu+Fe	wt -Cu+Fe	118.858	76.24927	-145.13	382.844	0.7664
wt +Cu+Fe	C3-1 +Cu+Fe	71.415	76.24927	-192.57	335.402	0.9773
wt +Cu+Fe	C2-4 +Cu+Fe	64.786	76.24927	-199.20	328.772	0.9868
wt +Cu+Fe	citf1 +Cu+Fe	41.223	76.24927	-222.76	305.209	0.9992
citf1 +Cu+Fe	C3-1 +Cu+Fe	30.193	76.24927	-233.79	294.179	0.9999
citf1 +Cu+Fe	C2-4 +Cu+Fe	23.563	76.24927	-240.42	287.550	1.0000
wt -Cu+Fe	C2-4 -Cu+Fe	18.562	76.24927	-245.42	282.549	1.0000
C2-4 +Cu+Fe	C3-1 +Cu+Fe	6.629	76.24927	-257.36	270.616	1.0000

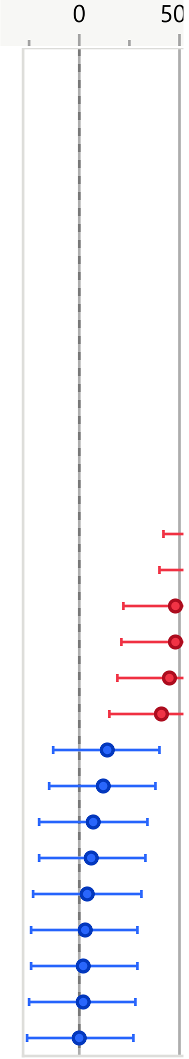

Missing Rows 24

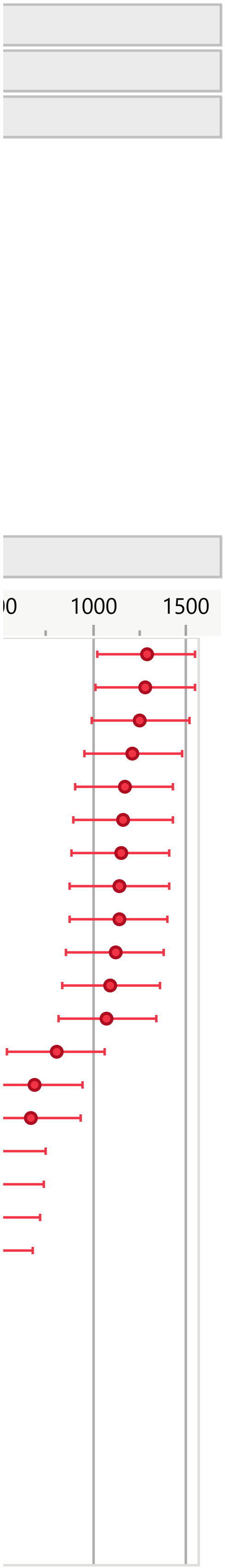
1000
1500

Oneway Analysis of Fe shoots By Treatment

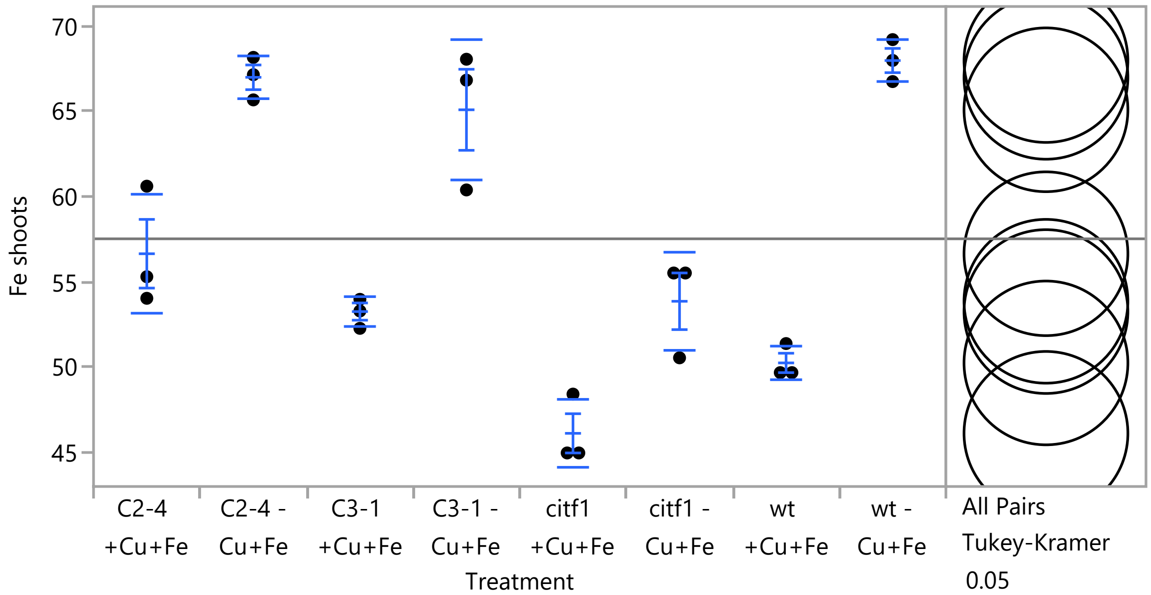

Means and Std Deviations

Level	Number	Mean	Std Dev	Std Err Mean	Lower 95%	Upper 95%
citf1 +Cu+Fe	3	46.117129	1.991819	1.1499772	41.169176	51.065082
citf1 -Cu+Fe	3	53.859087	2.8759735	1.6604441	46.714773	61.003401
wt +Cu+Fe	3	50.245034	0.9849442	0.5686578	47.798296	52.691771
C2-4 +Cu+Fe	3	56.646083	3.4872483	2.0133637	47.983278	65.308888
C3-1 +Cu+Fe	3	53.256161	0.8747302	0.5050257	51.083211	55.429111
C3-1 -Cu+Fe	3	65.085244	4.114789	2.3756745	54.863542	75.306947
C2-4 -Cu+Fe	3	66.989006	1.2529188	0.723373	63.876583	70.101429
wt -Cu+Fe	3	67.97295	1.230088	0.7101916	64.917242	71.028658

Means Comparisons

Comparisons for all pairs using Tukey-Kramer HSD

Confidence Quantile

q*	Alpha
3.46215	0.05

HSD Threshold Matrix

Abs(Dif)-HSD

	wt -Cu+Fe	C2-4 -Cu+Fe	C3-1 -Cu+Fe	C2-4 +Cu+Fe	citf1 -Cu+Fe	C3-1 +Cu+Fe	wt +Cu+Fe	citf1 +Cu
wt -Cu+Fe	-6.789	-5.806	-3.902	4.537	7.324	7.927	10.938	15
C2-4 -Cu+Fe	-5.806	-6.789	-4.886	3.553	6.340	6.943	9.954	14
C3-1 -Cu+Fe	-3.902	-4.886	-6.789	1.650	4.437	5.040	8.051	12
C2-4 +Cu+Fe	4.537	3.553	1.650	-6.789	-4.002	-3.400	-0.388	3
citf1 -Cu+Fe	7.324	6.340	4.437	-4.002	-6.789	-6.187	-3.175	0
C3-1 +Cu+Fe	7.927	6.943	5.040	-3.400	-6.187	-6.789	-3.778	0
wt +Cu+Fe	10.938	9.954	8.051	-0.388	-3.175	-3.778	-6.789	-2
citf1 +Cu+Fe	15.066	14.082	12.179	3.739	0.952	0.350	-2.662	-6

Positive values show pairs of means that are significantly different.

[illegible]+Fe
[illegible].066
[illegible].082
[illegible].179
[illegible].739
[illegible].952
[illegible].350
[illegible].662
[illegible].789

Oneway Analysis of Fe shoots By Treatment

Means Comparisons

Comparisons for all pairs using Tukey-Kramer HSD

Connecting Letters Report

Level			Mean	Std Error
wt -Cu+Fe	A		67.973	1.3867
C2-4 -Cu+Fe	A		66.989	1.3867
C3-1 -Cu+Fe	A		65.085	1.3867
C2-4 +Cu+Fe	B		56.646	1.3867
citf1 -Cu+Fe	B		53.859	1.3867
C3-1 +Cu+Fe	B		53.256	1.3867
wt +Cu+Fe	B	C	50.245	1.3867
citf1 +Cu+Fe		C	46.117	1.3867

Levels not connected by same letter are significantly different.

Ordered Differences Report

Level	- Level	Difference	Std Err Dif	Lower CL	Upper CL	p-Value
wt -Cu+Fe	citf1 +Cu+Fe	21.85582	1.961061	15.0663	28.64531	<.0001*
C2-4 -Cu+Fe	citf1 +Cu+Fe	20.87188	1.961061	14.0824	27.66137	<.0001*
C3-1 -Cu+Fe	citf1 +Cu+Fe	18.96812	1.961061	12.1786	25.75761	<.0001*
wt -Cu+Fe	wt +Cu+Fe	17.72792	1.961061	10.9384	24.51741	<.0001*
C2-4 -Cu+Fe	wt +Cu+Fe	16.74397	1.961061	9.9545	23.53346	<.0001*
C3-1 -Cu+Fe	wt +Cu+Fe	14.84021	1.961061	8.0507	21.62970	<.0001*
wt -Cu+Fe	C3-1 +Cu+Fe	14.71679	1.961061	7.9273	21.50628	<.0001*
wt -Cu+Fe	citf1 -Cu+Fe	14.11386	1.961061	7.3244	20.90335	<.0001*
C2-4 -Cu+Fe	C3-1 +Cu+Fe	13.73285	1.961061	6.9434	20.52234	<.0001*
C2-4 -Cu+Fe	citf1 -Cu+Fe	13.12992	1.961061	6.3404	19.91941	0.0001*
C3-1 -Cu+Fe	C3-1 +Cu+Fe	11.82908	1.961061	5.0396	18.61857	0.0004*
wt -Cu+Fe	C2-4 +Cu+Fe	11.32687	1.961061	4.5374	18.11636	0.0006*
C3-1 -Cu+Fe	citf1 -Cu+Fe	11.22616	1.961061	4.4367	18.01565	0.0006*
C2-4 +Cu+Fe	citf1 +Cu+Fe	10.52895	1.961061	3.7395	17.31844	0.0013*
C2-4 -Cu+Fe	C2-4 +Cu+Fe	10.34292	1.961061	3.5534	17.13241	0.0015*
C3-1 -Cu+Fe	C2-4 +Cu+Fe	8.43916	1.961061	1.6497	15.22865	0.0099*
citf1 -Cu+Fe	citf1 +Cu+Fe	7.74196	1.961061	0.9525	14.53145	0.0198*
C3-1 +Cu+Fe	citf1 +Cu+Fe	7.13903	1.961061	0.3495	13.92852	0.0357*
C2-4 +Cu+Fe	wt +Cu+Fe	6.40105	1.961061	-0.3884	13.19054	0.0721
wt +Cu+Fe	citf1 +Cu+Fe	4.12790	1.961061	-2.6616	10.91740	0.4513
citf1 -Cu+Fe	wt +Cu+Fe	3.61405	1.961061	-3.1754	10.40354	0.6033
C2-4 +Cu+Fe	C3-1 +Cu+Fe	3.38992	1.961061	-3.3996	10.17941	0.6709
C3-1 +Cu+Fe	wt +Cu+Fe	3.01113	1.961061	-3.7784	9.80062	0.7788
wt -Cu+Fe	C3-1 -Cu+Fe	2.88771	1.961061	-3.9018	9.67720	0.8107
C2-4 +Cu+Fe	citf1 -Cu+Fe	2.78700	1.961061	-4.0025	9.57649	0.8351
C2-4 -Cu+Fe	C3-1 -Cu+Fe	1.90376	1.961061	-4.8857	8.69325	0.9725
wt -Cu+Fe	C2-4 -Cu+Fe	0.98394	1.961061	-5.8055	7.77343	0.9995
citf1 -Cu+Fe	C3-1 +Cu+Fe	0.60293	1.961061	-6.1866	7.39242	1.0000

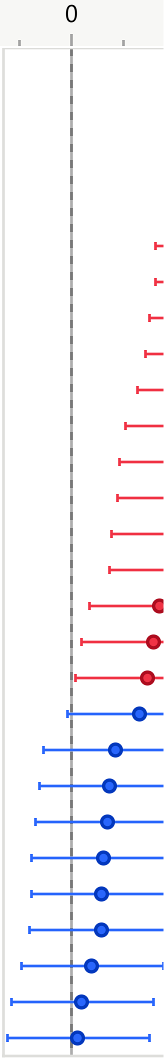

Missing Rows 24

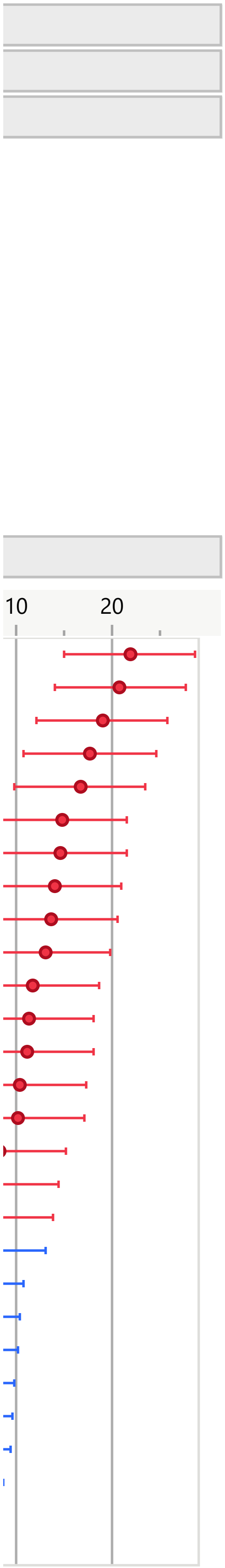
10
20

Oneway Analysis of Relative expression (set wt-ctr as 1) By Treatment

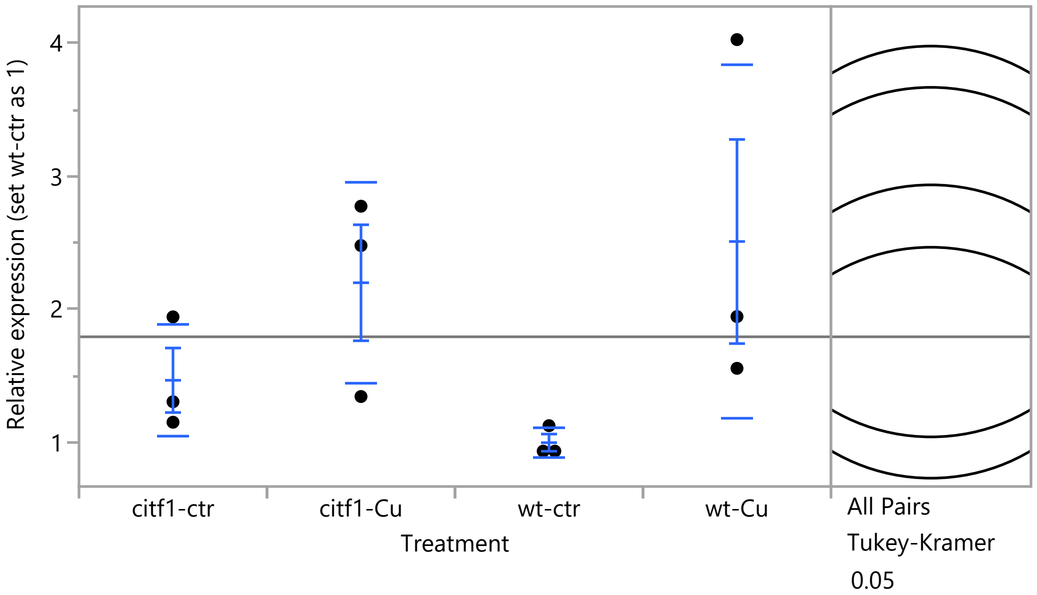

Means and Std Deviations

Level	Number	Mean	Std Dev	Std Err Mean	Lower 95%	Upper 95%
citf1-ctr	3	1.4683913	0.4195476	0.2422259	0.4261772	2.5106054
citf1-Cu	3	2.2006099	0.7543804	0.4355417	0.3266252	4.0745947
wt-ctr	3	1	0.1117261	0.0645051	0.7224571	1.2775429
wt-Cu	3	2.5104608	1.3273055	0.7663202	-0.786749	5.8076704

Oneway Analysis of Relative expression (set wt-ctr as 1) By Treatment

Means Comparisons

Comparisons for all pairs using Tukey-Kramer HSD

Confidence Quantile

q*	Alpha
3.20234	0.05

HSD Threshold Matrix

Abs(Dif)-HSD

	wt-Cu	citf1-Cu	citf1-ctr	wt-ctr
wt-Cu	-2.0751	-1.7652	-1.0330	-0.5646
citf1-Cu	-1.7652	-2.0751	-1.3429	-0.8745
citf1-ctr	-1.0330	-1.3429	-2.0751	-1.6067
wt-ctr	-0.5646	-0.8745	-1.6067	-2.0751

Positive values show pairs of means that are significantly different.

Connecting Letters Report

Level		Mean	Std Error
wt-Cu	A	2.5105	0.45820
citf1-Cu	A	2.2006	0.45820
citf1-ctr	A	1.4684	0.45820
wt-ctr	A	1.0000	0.45820

Levels not connected by same letter are significantly different.

Ordered Differences Report

Level	- Level	Difference	Std Err Dif	Lower CL	Upper CL	p-Value
wt-Cu	wt-ctr	1.510461	0.6479882	-0.56462	3.585542	0.1698
citf1-Cu	wt-ctr	1.200610	0.6479882	-0.87447	3.275691	0.3183
wt-Cu	citf1-ctr	1.042069	0.6479882	-1.03301	3.117151	0.4260
citf1-Cu	citf1-ctr	0.732219	0.6479882	-1.34286	2.807300	0.6828
citf1-ctr	wt-ctr	0.468391	0.6479882	-1.60669	2.543473	0.8853
wt-Cu	citf1-Cu	0.309851	0.6479882	-1.76523	2.384932	0.9618

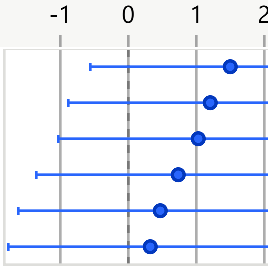

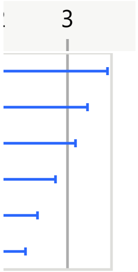
3

Oneway Analysis of EXPRESSION By Sample

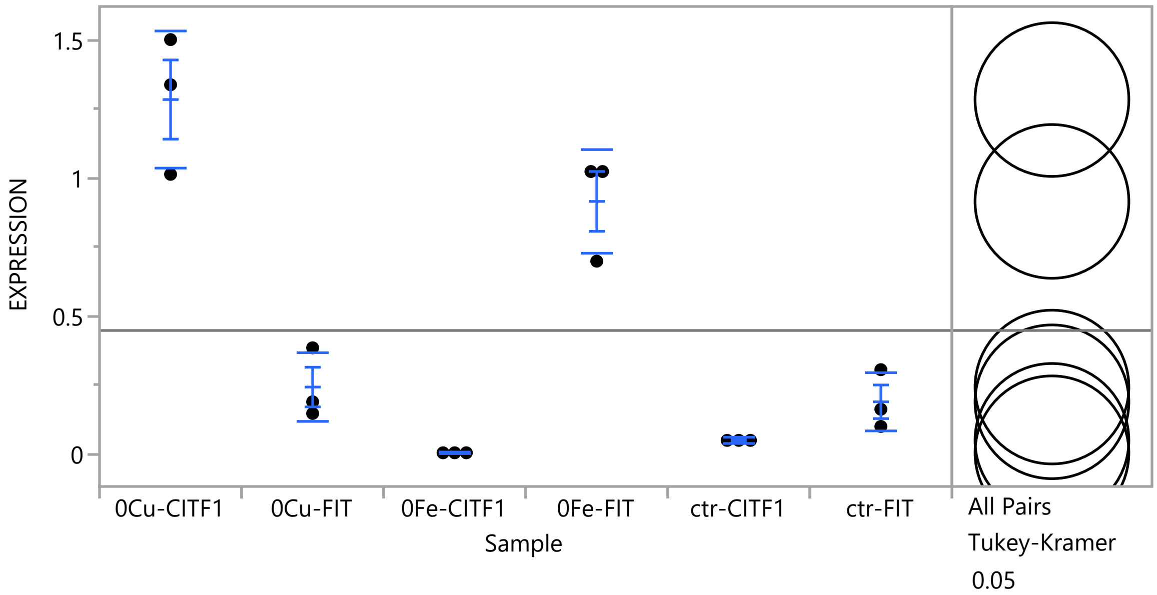

Means and Std Deviations

Level	Number	Mean	Std Dev	Std Err Mean	Lower 95%	Upper 95%
0Cu-CITF1	3	1.28252	0.2483891	0.1434075	0.6654872	1.8995528
0Cu-FIT	3	0.2410533	0.1244465	0.0718492	-0.068089	0.5501955
0Fe-CITF1	3	0.0029567	0.0028691	0.0016565	-0.004171	0.0100839
0Fe-FIT	3	0.9135533	0.1877145	0.108377	0.4472447	1.379862
ctr-CITF1	3	0.0479533	0.0113036	0.0065262	0.0198735	0.0760331
ctr-FIT	3	0.1876833	0.1054712	0.0608938	-0.074322	0.4496882

Oneway Analysis of EXPRESSION By Sample

Means Comparisons

Comparisons for all pairs using Tukey-Kramer HSD

Confidence Quantile

q*	Alpha
3.35886	0.05

HSD Threshold Matrix

Abs(Dif)-HSD

	0Cu-CITF1	0Fe-FIT	0Cu-FIT	ctr-FIT	ctr-CITF1	0Fe-CITF1
0Cu-CITF1	-0.39375	-0.02479	0.64771	0.70108	0.84081	0.88581
0Fe-FIT	-0.02479	-0.39375	0.27875	0.33212	0.47185	0.51684
0Cu-FIT	0.64771	0.27875	-0.39375	-0.34038	-0.20065	-0.15566
ctr-FIT	0.70108	0.33212	-0.34038	-0.39375	-0.25402	-0.20903
ctr-CITF1	0.84081	0.47185	-0.20065	-0.25402	-0.39375	-0.34876
0Fe-CITF1	0.88581	0.51684	-0.15566	-0.20903	-0.34876	-0.39375

Positive values show pairs of means that are significantly different.

Connecting Letters Report

Level			Mean	Std Error
0Cu-CITF1	A		1.2825	0.08289
0Fe-FIT	A		0.9136	0.08289
0Cu-FIT		B	0.2411	0.08289
ctr-FIT		B	0.1877	0.08289
ctr-CITF1		B	0.0480	0.08289
0Fe-CITF1		B	0.0030	0.08289

Levels not connected by same letter are significantly different.

Ordered Differences Report

Level	- Level	Difference	Std Err Dif	Lower CL	Upper CL	p-Value
0Cu-CITF1	0Fe-CITF1	1.279563	0.1172277	0.885811	1.673315	<.0001*
0Cu-CITF1	ctr-CITF1	1.234567	0.1172277	0.840815	1.628319	<.0001*
0Cu-CITF1	ctr-FIT	1.094837	0.1172277	0.701085	1.488589	<.0001*
0Cu-CITF1	0Cu-FIT	1.041467	0.1172277	0.647715	1.435219	<.0001*
0Fe-FIT	0Fe-CITF1	0.910597	0.1172277	0.516845	1.304349	<.0001*
0Fe-FIT	ctr-CITF1	0.865600	0.1172277	0.471848	1.259352	<.0001*
0Fe-FIT	ctr-FIT	0.725870	0.1172277	0.332118	1.119622	0.0005*
0Fe-FIT	0Cu-FIT	0.672500	0.1172277	0.278748	1.066252	0.0010*
0Cu-CITF1	0Fe-FIT	0.368967	0.1172277	-0.024785	0.762719	0.0711
0Cu-FIT	0Fe-CITF1	0.238097	0.1172277	-0.155655	0.631849	0.3804
0Cu-FIT	ctr-CITF1	0.193100	0.1172277	-0.200652	0.586852	0.5863
ctr-FIT	0Fe-CITF1	0.184727	0.1172277	-0.209025	0.578479	0.6274
ctr-FIT	ctr-CITF1	0.139730	0.1172277	-0.254022	0.533482	0.8326
0Cu-FIT	ctr-FIT	0.053370	0.1172277	-0.340382	0.447122	0.9969
ctr-CITF1	0Fe-CITF1	0.044997	0.1172277	-0.348755	0.438749	0.9986

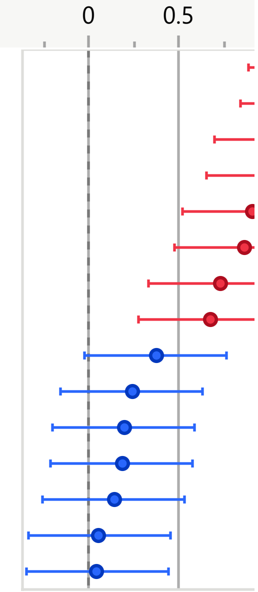

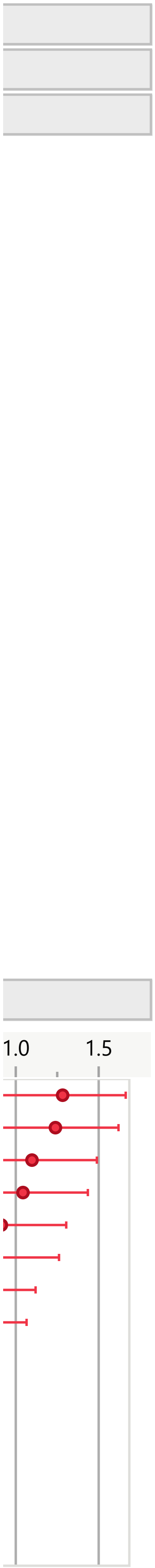
1.0
1.5

Oneway Analysis of CITF1 By Sample

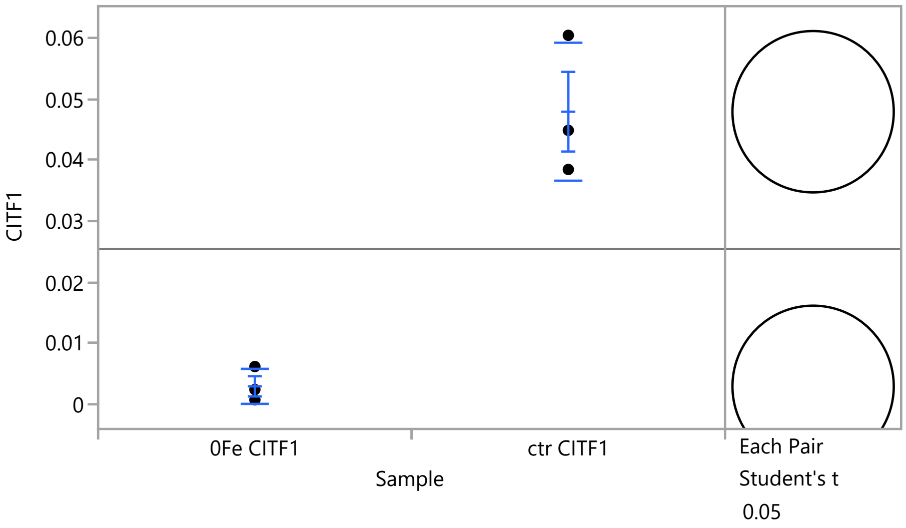

Means and Std Deviations

Level	Number	Mean	Std Dev	Std Err Mean	Lower 95%	Upper 95%
0Fe CITF1	3	0.0029567	0.0028691	0.0016565	-0.004171	0.0100839
ctr CITF1	3	0.0479533	0.0113036	0.0065262	0.0198735	0.0760331

Means Comparisons

Comparisons for each pair using Student's t

Confidence Quantile

t	Alpha
2.77645	0.05

LSD Threshold Matrix

Abs(Dif)-LSD

	ctr CITF1	0Fe CITF1
ctr CITF1	-0.01869	0.02630
0Fe CITF1	0.02630	-0.01869

Positive values show pairs of means that are significantly different.

Connecting Letters Report

Level			Mean	Std Error
ctr CITF1	A		0.04795	0.00476
0Fe CITF1		B	0.00296	0.00476

Levels not connected by same letter are significantly different.

Ordered Differences Report

Level	- Level	Difference	Std Err Dif	Lower CL	Upper CL	p-Value	
ctr CITF1	0Fe CITF1	0.0449967	0.0067331	0.0263026	0.0636907	0.0026*	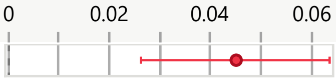

Oneway Analysis of Relative expression By Sample

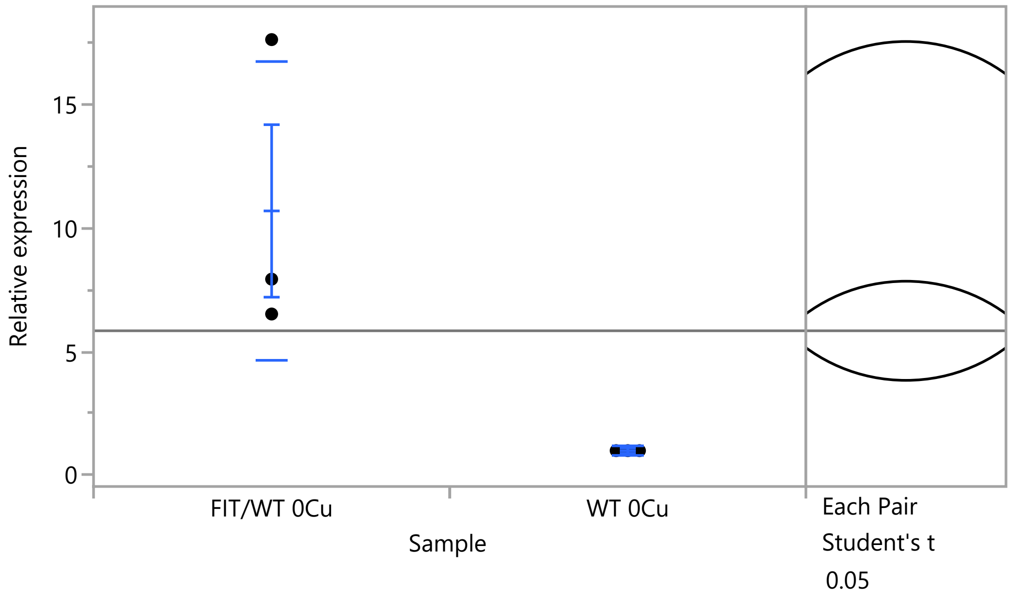

Means and Std Deviations

Level	Number	Mean	Std Dev	Std Err Mean	Lower 95%	Upper 95%
FIT/WT 0Cu	3	10.705876	6.0525346	3.4944325	-4.329453	25.741205
WT 0Cu	3	0.9941171	0.193582	0.1117646	0.5132326	1.4750015

Means Comparisons

Comparisons for each pair using Student's t

Confidence Quantile

t	Alpha
2.77645	0.05

LSD Threshold Matrix

Abs(Dif)-LSD

	FIT/WT 0Cu	WT 0Cu
FIT/WT 0Cu	-9.7071	0.0047
WT 0Cu	0.0047	-9.7071

Positive values show pairs of means that are significantly different.

Connecting Letters Report

Level			Mean	Std Error
FIT/WT 0Cu	A		10.706	2.4722
WT 0Cu		B	0.994	2.4722

Levels not connected by same letter are significantly different.

Ordered Differences Report

Level	- Level	Difference	Std Err Dif	Lower CL	Upper CL	p-Value
FIT/WT 0Cu	WT 0Cu	9.711759	3.496219	0.0046979	19.41882	0.0499*

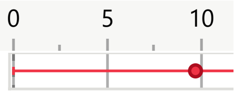

[illegible]

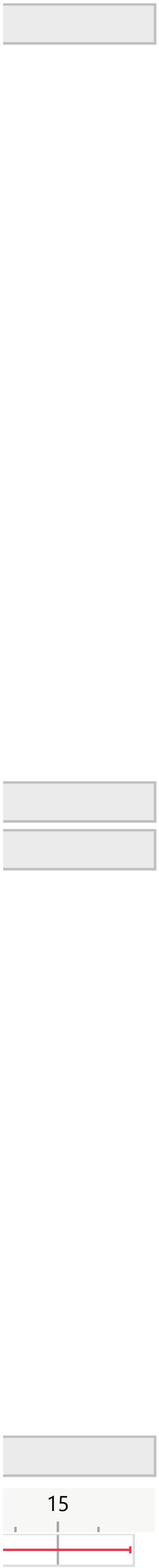

Oneway Analysis of Rosette size (mm) By Treatment

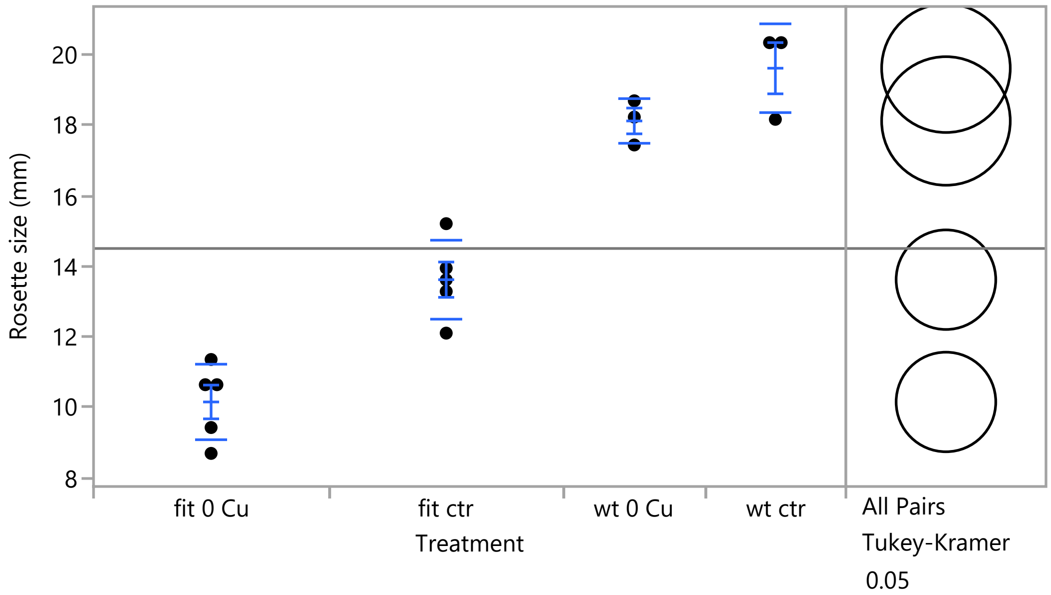

Means and Std Deviations

Level	Number	Mean	Std Dev	Std Err Mean	Lower 95%	Upper 95%
fit 0 Cu	5	10.1542	1.0712018	0.479056	8.8241273	11.484273
fit ctr	5	13.6234	1.1202305	0.5009823	12.23245	15.01435
wt 0 Cu	3	18.127	0.6335716	0.3657927	16.553121	19.700879
wt ctr	3	19.623333	1.2590005	0.7268843	16.495803	22.750864

Oneway Analysis of Rosette size (mm) By Treatment

Means Comparisons

Comparisons for all pairs using Tukey-Kramer HSD

Confidence Quantile

q*	Alpha
2.96880	0.05

HSD Threshold Matrix

Abs(Dif)-HSD

	wt ctr	wt 0 Cu	fit ctr	fit 0 Cu
wt ctr	-2.5789	-1.0826	3.6933	7.1625
wt 0 Cu	-1.0826	-2.5789	2.1970	5.6662
fit ctr	3.6933	2.1970	-1.9976	1.4716
fit 0 Cu	7.1625	5.6662	1.4716	-1.9976

Positive values show pairs of means that are significantly different.

Connecting Letters Report

Level				Mean	Std Error
wt ctr	A			19.623	0.61424
wt 0 Cu	A			18.127	0.61424
fit ctr		B		13.623	0.47579
fit 0 Cu			C	10.154	0.47579

Levels not connected by same letter are significantly different.

Ordered Differences Report

Level	- Level	Difference	Std Err Dif	Lower CL	Upper CL	p-Value
wt ctr	fit 0 Cu	9.469133	0.7769613	7.16249	11.77578	<.0001*
wt 0 Cu	fit 0 Cu	7.972800	0.7769613	5.66615	10.27945	<.0001*
wt ctr	fit ctr	5.999933	0.7769613	3.69329	8.30658	<.0001*
wt 0 Cu	fit ctr	4.503600	0.7769613	2.19695	6.81025	0.0004*
fit ctr	fit 0 Cu	3.469200	0.6728682	1.47159	5.46681	0.0012*
wt ctr	wt 0 Cu	1.496333	0.8686691	-1.08257	4.07524	0.3547

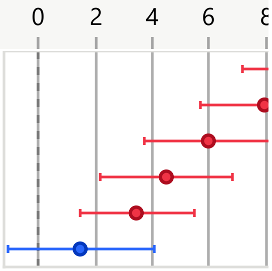

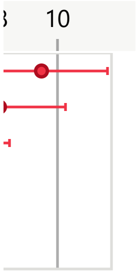
10
