# Supplementary material for: CITF1 interacts with FIT and regulates copper–iron crosstalk in Arabidopsis
Source: Plant Cell. 2026 Apr 16;38(5):koag114. doi: 10.1093/plcell/koag114 (PMC13143221; doi:10.1093/plcell/koag114)
Supplement: koag114_Supplementary_Data [file koag114_supplementary_data.zip › Supplemental Information.pdf]

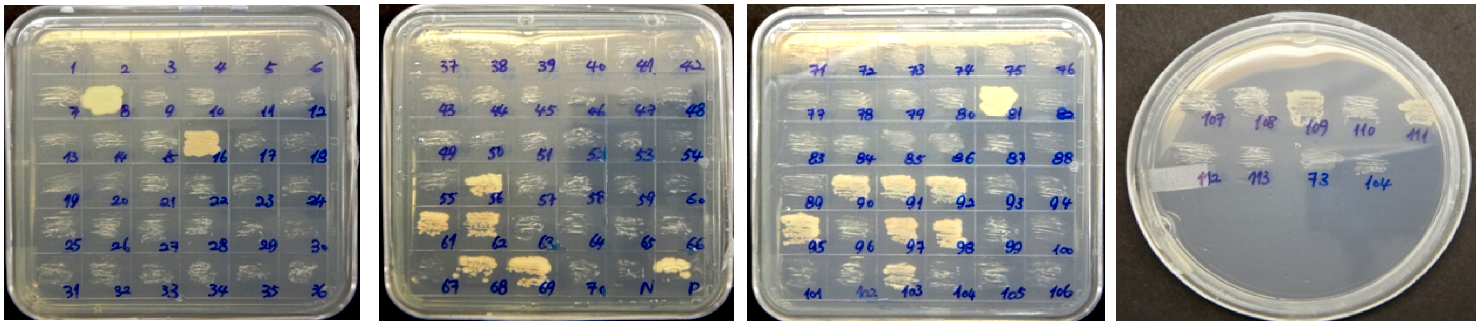

Figure S1. The Identification of CITF1-interacting partners using Yeast-Two-Hybrid assay (supports Figure 1). The Y2H assays were carried out using the Matchmaker Gold Yeast 2-Hybrid System. The Y2HGold yeast strain carrying the *CITF1* cDNA in the pGBKT7 bait vector was mated with the Y187 yeast strain carrying the yeast-two-hybrid cDNA library of *A. thaliana*. Protein interactions were selected on solid SD/–Ade/–His/–Leu/–Trp dropout medium. Numbers indicate individual colonies. N and P represent negative and positive controls, respectively, as in **Figure 1**.

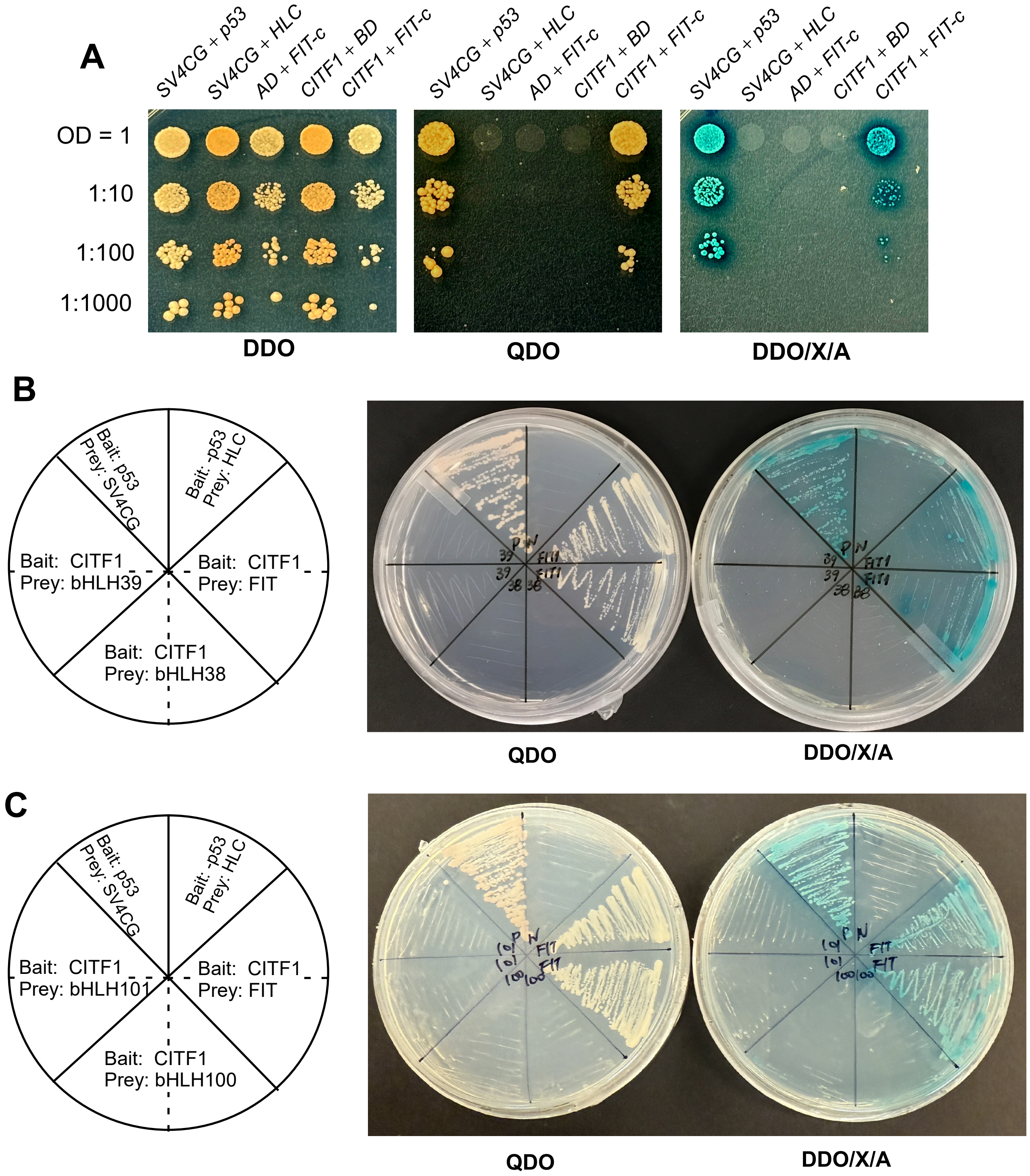

Figure S2. **CITF1 interacts with FIT but not bHLH38, bHLH39, bHLH100 or bHLH101 (supports Figure 1).** In **(A)**, *CITF1* cDNA was fused to the GAL4 activation domain (AD) of the prey vector, pGADT7, while the C-terminal part of FIT (FIT-C) was fused to the GAL4 DNA-binding domain (BD) of the bait vector, pGBKT7. In **(B** and **C)**, *CITF1* cDNA was cloned into the bait vector pGBKT7 and expressed as a fusion to the GAL4 DNA-binding domain. *FIT*, *bHLH38* or *bHLH39* cDNAs (**B**) or bHLH100 or bHLH101 (**C**) were cloned individually into the prey vector pGADT7 and expressed as fusions to the GAL4 activation domain. Combinations of bait and prey-expressing cells, along with their positions on the Petri plates, are shown on the left. Protein-protein interactions are evidenced by the ability of cells to grow on a quadruple dropout medium lacking Leu/Trp/His/Ade (QDO) and visualized on the double dropout medium lacking Leu/Trp containing 40 µg/mL X-a-Gal and 0.2 µg/mL Aureobasidin A (DDO/X/A). The bait vector expressing p53 and the prey vector expressing SV40 large T antigen (SV4CG) were used as positive control, and the prey vector expressing human lamin C (HLC) was used as a negative control. The data shown are representative of 3 independent experiments.

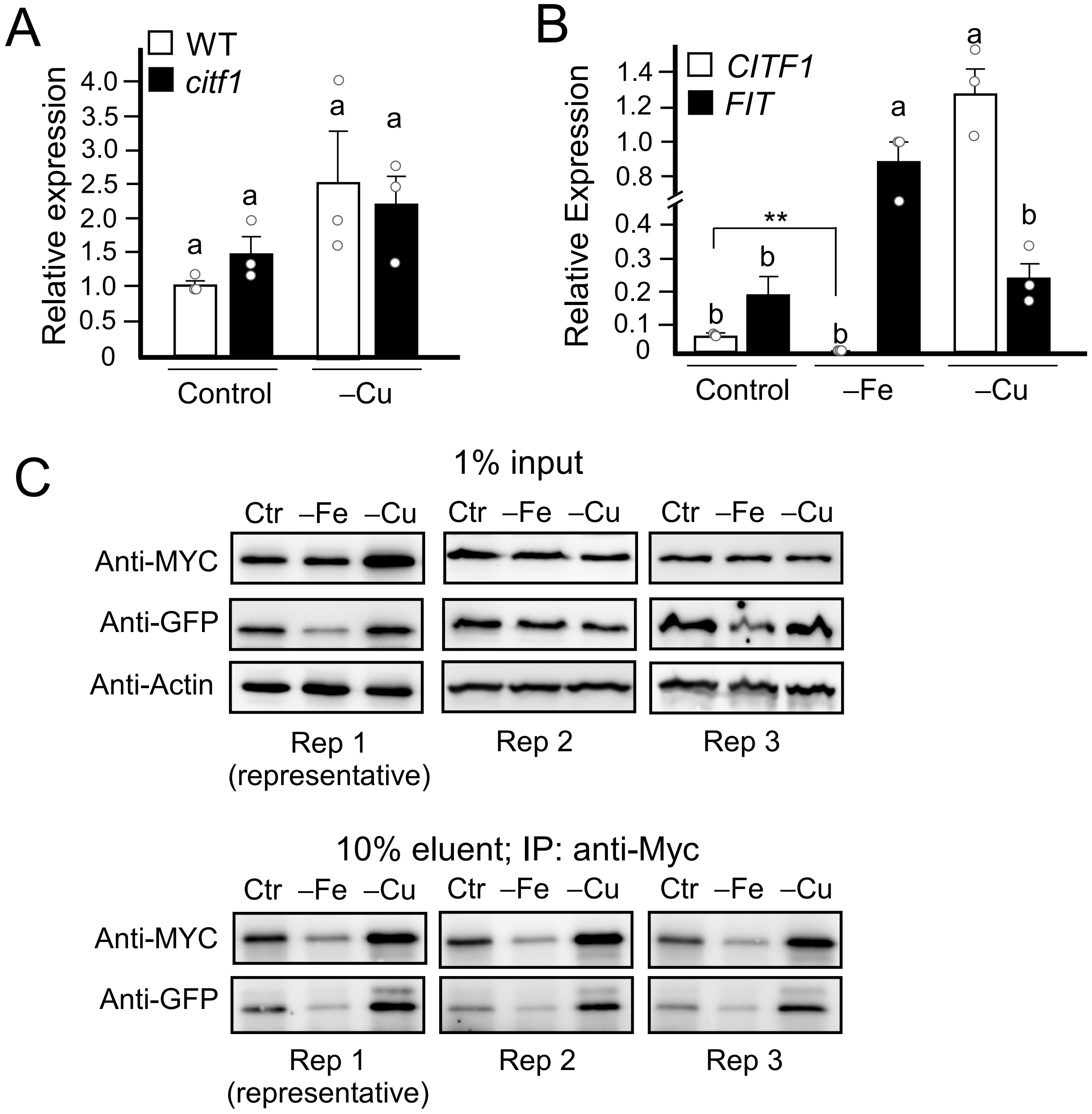

Figure S3. Transcriptional response of *FIT* to copper deficiency and *CITF1* to copper and iron deficiencies (supports Figure 2). (A) Transcriptional abundance of *FIT* in roots of wild-type and the *citf1* mutant grown under control or Cu-depleted conditions. Plants were grown hydroponically with or without 250 nM $CuSO_4$ (control and -Cu, respectively) for 4 weeks. In **(B),** wild-type plants were grown hydroponically with 10 µM Fe-HBED and 250 nM Cu (**Control**) or without Cu (-**Cu**) for four weeks. For Fe-deficiency treatment (-Fe), plants were grown in a control hydroponic solution for 3 weeks before being transferred to a medium without added Fe and grown for 1 week. In (**A**) and (**B**), roots were collected for RT-qPCR analysis. *CITF1* and *FIT* expression were normalized to *ACT2*. Values are mean ± SE (n = 3 independent experiments with samples pooled from 4 plants per experiment). Levels not connected by the same letter are statistically different (ANOVA, followed by Tukey HSD, JMP Pro 17 software package). Asterisks indicate statistically significant differences of planned comparisons between groups ($P < 0.01$, Student's *t*-test). (**C**) The CITF1-FIT protein complex was co-immunoprecipitated from five-day-old *citf1-1* mutant seedlings co-expressing CITF1-MYC and FIT-GFP constructs. Plants were grown in control or iron-, or copper-depleted hydroponic solutions for 5 days before tissue collection, protein extractions, co-IP with anti-MYC antibody, and western blot analysis using the anti-GFP and anti-MYC antibodies. Rep. 1 to 3 indicate results from three independent experiments used for quantification in **Figure 2** of the main manuscript. Rep. 1 is a representative result shown in **Figure 2A** of the main manuscript.

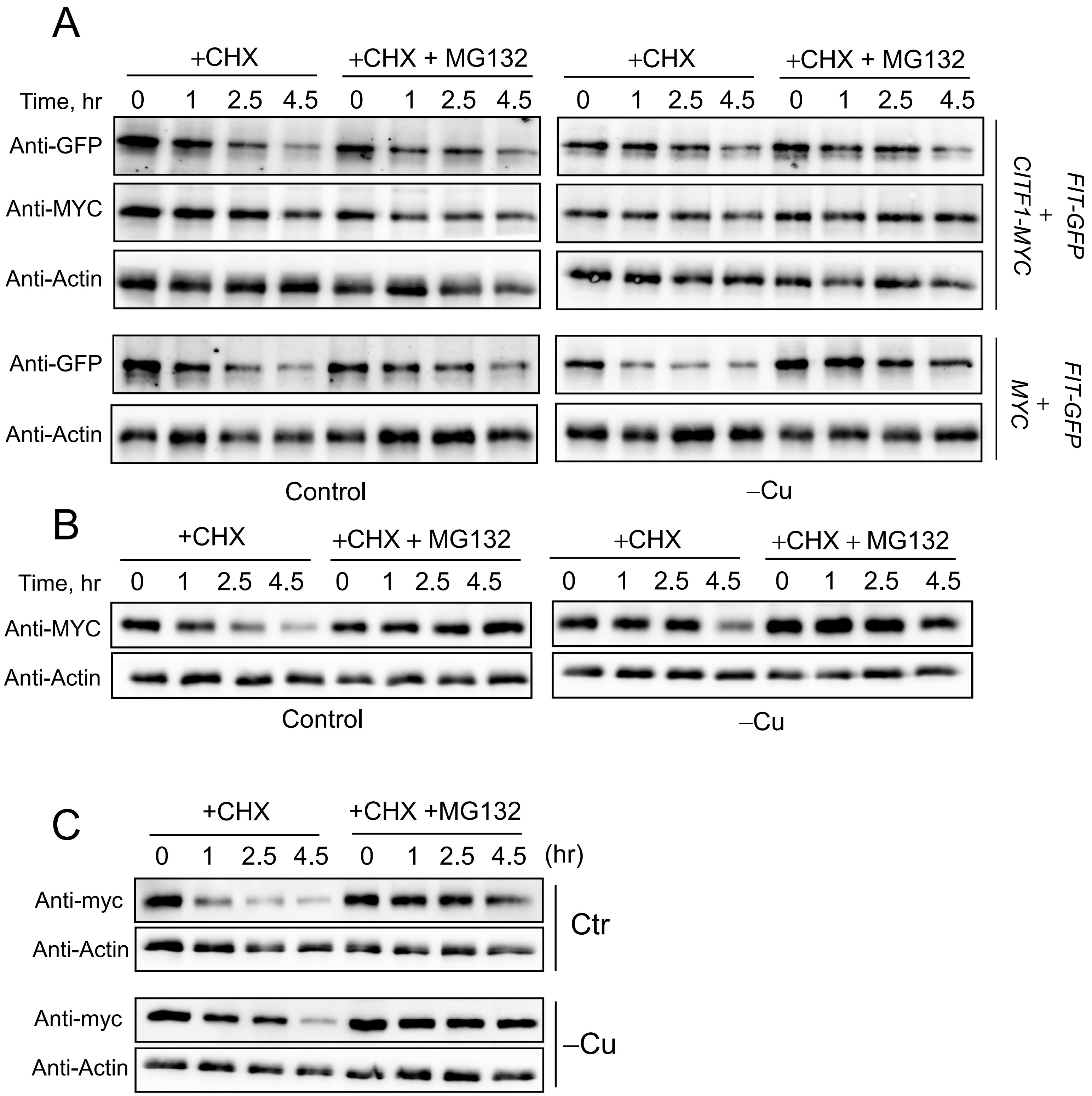

Figure S4. Copper deficiency stabilizes CITF1 and FIT proteins (supports Figure 3C, E). Western blot analyses of FIT-GFP and CITF1-MYC in *35S*$_{pro}$*:FIT-GFP* + *35S*$_{pro}$*:CITF1-MYC* and *35S*$_{pro}$*:FIT-GFP* + *35S*$_{pro}$*:MYC* (**A**), *35S*$_{pro}$*:CITF1-MYC* (Line 1) (**B**), and *35S*$_{pro}$*:CITF1-MYC* (Line 2) (**C**), all expressed in the *citf1* mutant background. Plants were grown hydroponically for 5 days with or without 250 nM $CuSO_4$ (Control and – Cu, respectively) before being treated with 355 µM CHX or 355 µM CHX + 100 µM MG-132 (CHX or CHX+MG132, respectively) for the indicated time. The CITF1 protein level was determined by Western blots using the anti-MYC antibody; the FIT protein level was determined with the anti-GFP antibody. Western blots of anti-Actin represent the loading control. CITF1-MYC, FIT-GFP, and Actin migrated on the SDS-PAGE as expected for their predicted molecular weights (~63 kDa for CITF1-MYC (TAP), 63-72 kDa for FIT-GFP, and ~42 kDa for Actin) as estimated using a pre-stained protein standards (BioRad).

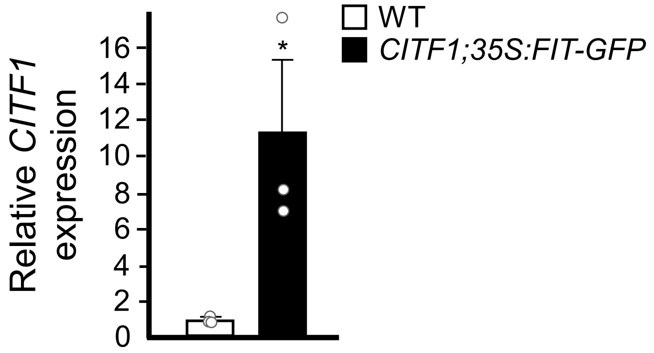

Figure S5. **The expression of *CITF1* in wild-type plants and the transgenic wild-type line that expresses *35S$_{pro}$:FIT-GFP* that were used for ChIP assays (supports Figure 3 and 4D, E).** The transcript abundance of *CITF1* in 5-day-old seedlings of wild-type and *35S$_{pro}$:FIT-GFP* plants grown hydroponically without Cu. Values are means ± SE (n = 3 independent experiments with the roots of four plants pooled together per experiment). Asterisks indicate statistically significant differences between groups ($P < 0.05$, Student's *t-test*).

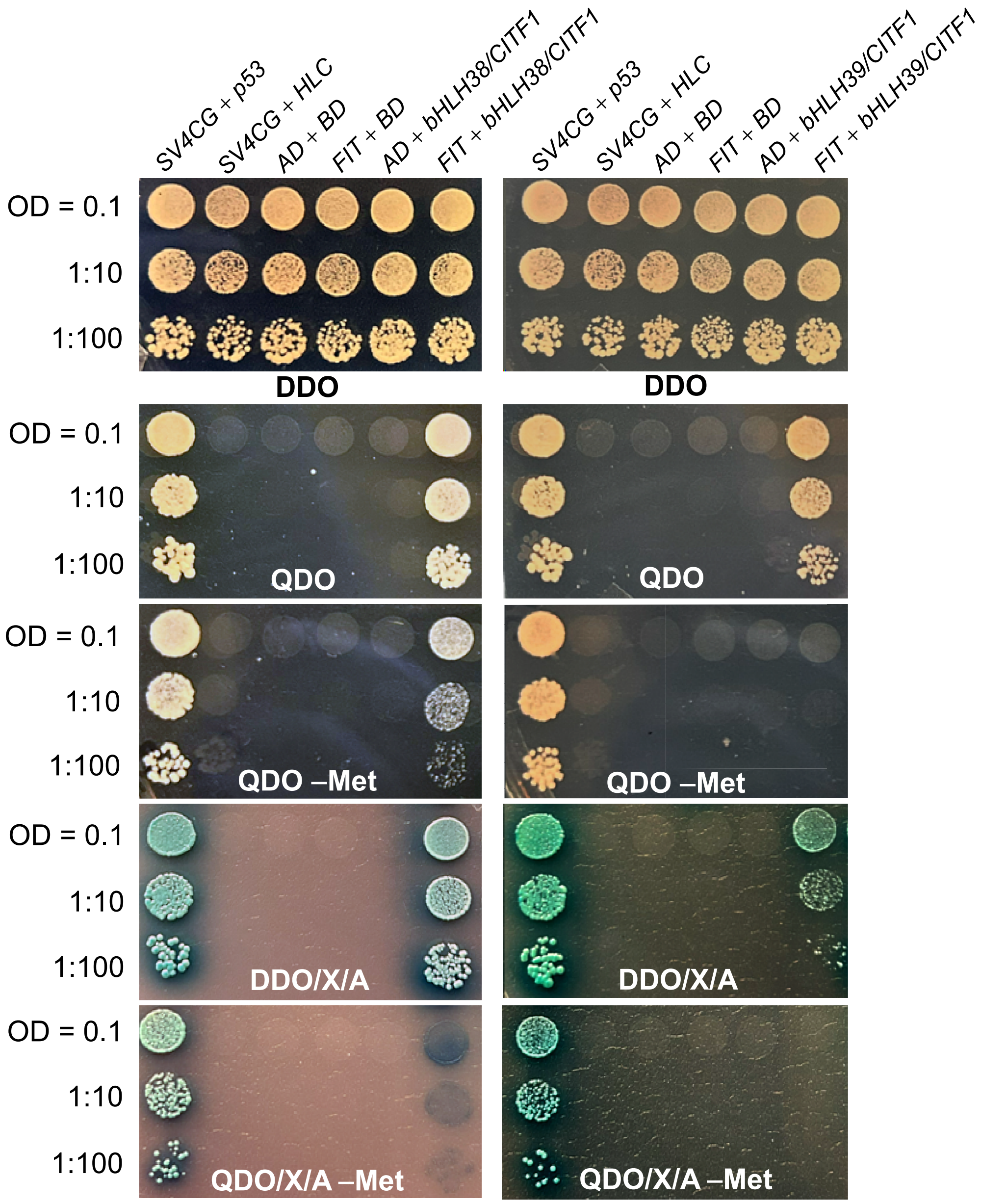

Supplemental Figure 6. CITF1 disrupts FIT-bHLH38 and FIT-bHLH39 interactions (supports Figure 6). The yeast cells harboring pGADT7-FIT and pBridge-bHLH38-CITF1 or pBridge-bHLH39-CITF1 were grown overnight to an OD_{600nm} = 1.0 in a liquid dropout medium lacking Leu/Trp (DDO). The overnight cultures were diluted to an OD_{600nm} = 0.1, then serially 10-time diluted as indicated on the left and spotted either on the solid DDO medium or on a quadruple dropout medium lacking Leu/Trp/His/Ade with or without methionine (QDO and QDO –Met, respectively) to select for protein-protein interactions. Protein-protein interactions were also visualized on DDO plates in the presence of 40 µg/mL X-α-Gal and 0.2 µg/mL Aureobasidin A (DDO/X/A and DDO/X/A –Met, respectively). The prey vector expressing SV40 large T antigen (*SV4CG*) and probed with the bait vector expressing *p53* or human lamin C (*HLC*) were used as positive and negative controls, respectively.

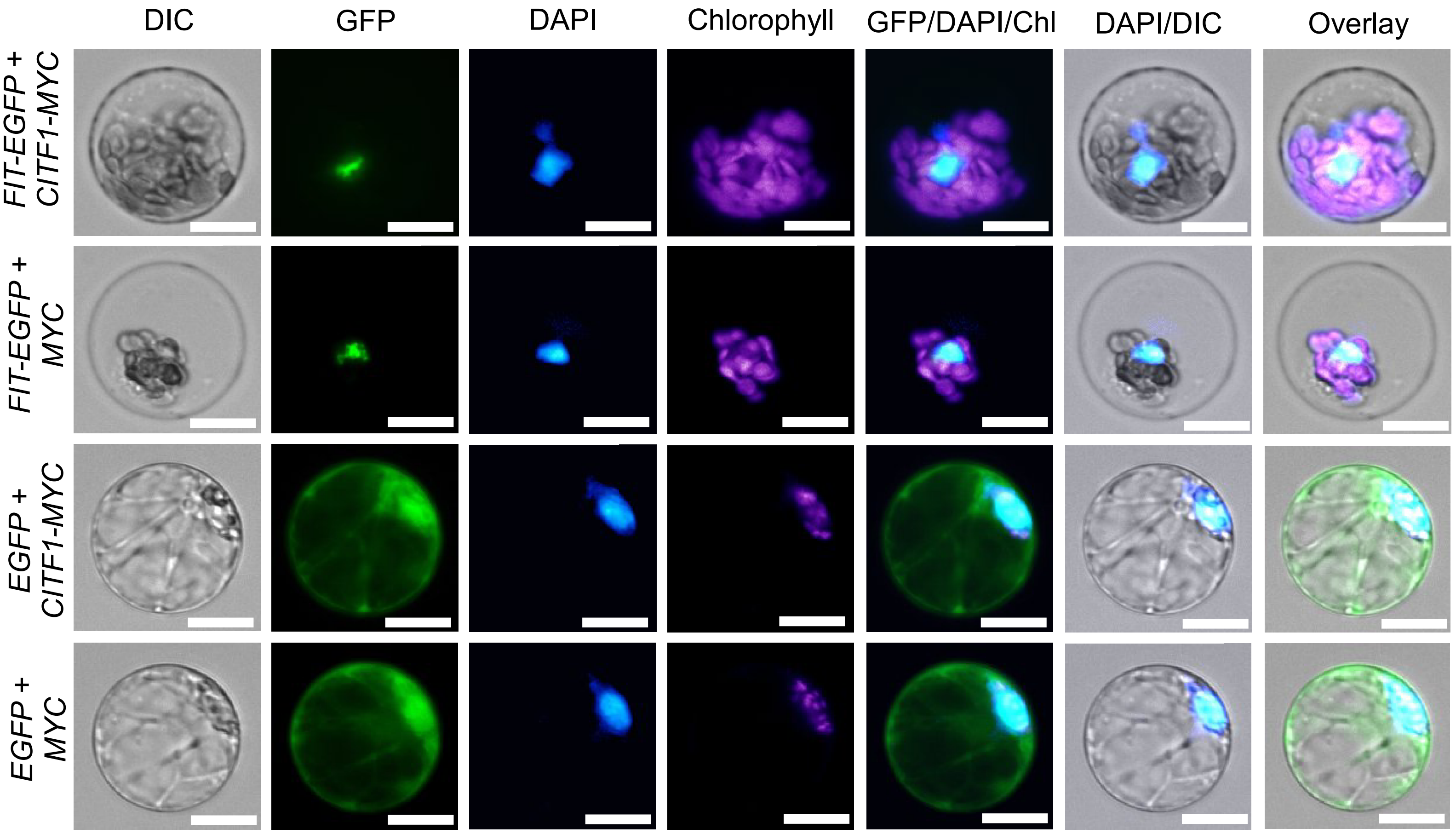

Figure 7. CITF1 and FIT co-localize to the nucleus in *A. thaliana* protoplasts (supports Figure 7). *A. thaliana* leaf protoplasts were isolated from the *citf1* mutant expressing *35S$_{pro}$:CITF1-MYC* or the empty vector (*citf1;CITF1-MYC* and *citf1;MYC*, respectively; constructs discussed in **Figure 1B**) were transfected with the vector expressing EGFP, or the vector expressing FIT fused with EGFP at the C terminus (FIT-EGFP). GFP- and DAPI-mediated fluorescence and chlorophyll autofluorescence were visualized separately. Superimposed images of chlorophyll autofluorescence and GFP-mediated fluorescence (Overlay) were created to demonstrate that green fluorescence was derived from GFP. Bar = 20 µm.

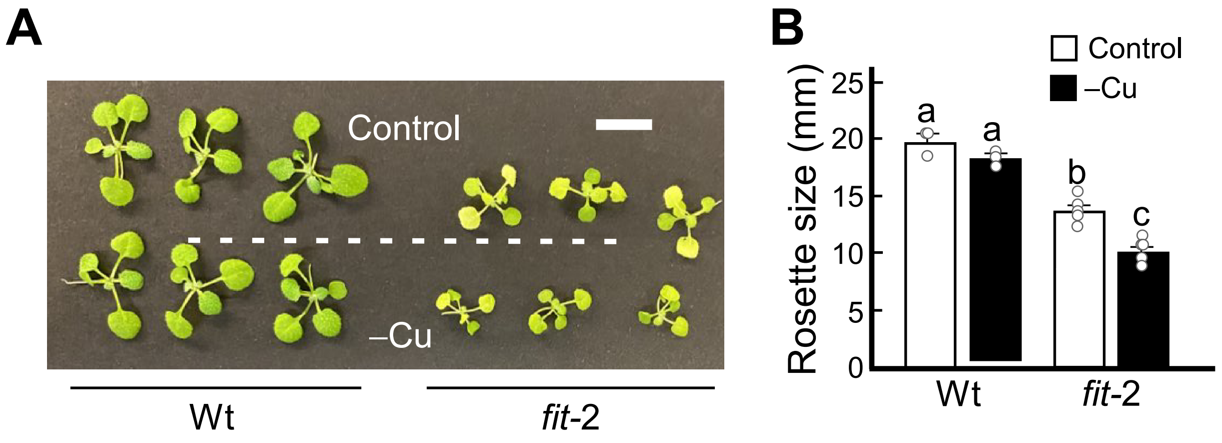

Figure S8. The *fit*-2 mutant is more sensitive to Cu deficiency than the wild-type (supports Figure 8). (**A**) The phenotype of the three-week-old wild-type and the *fit*-2 mutant, grown hydroponically with 75 µM Fe-HBED and with or without 250 nM $CuSO_4$. A representative result from three independent experiments is shown. Scale bar = 10 mm. (**B**) The rosette size of wild-type and *fit*-2 mutant grown as described in (**A**). The rosette size was determined by measuring the maximum distance between two points on the rosette boundary (Camargo *et al.*, 2014). Values are mean ± SE (n = 3-5 plants per experiment from three independent experiments). Different lowercase letters indicate significant differences ($P < 0.05$; ANOVA, followed by Tukey HSD, JMP Pro 17 software package).

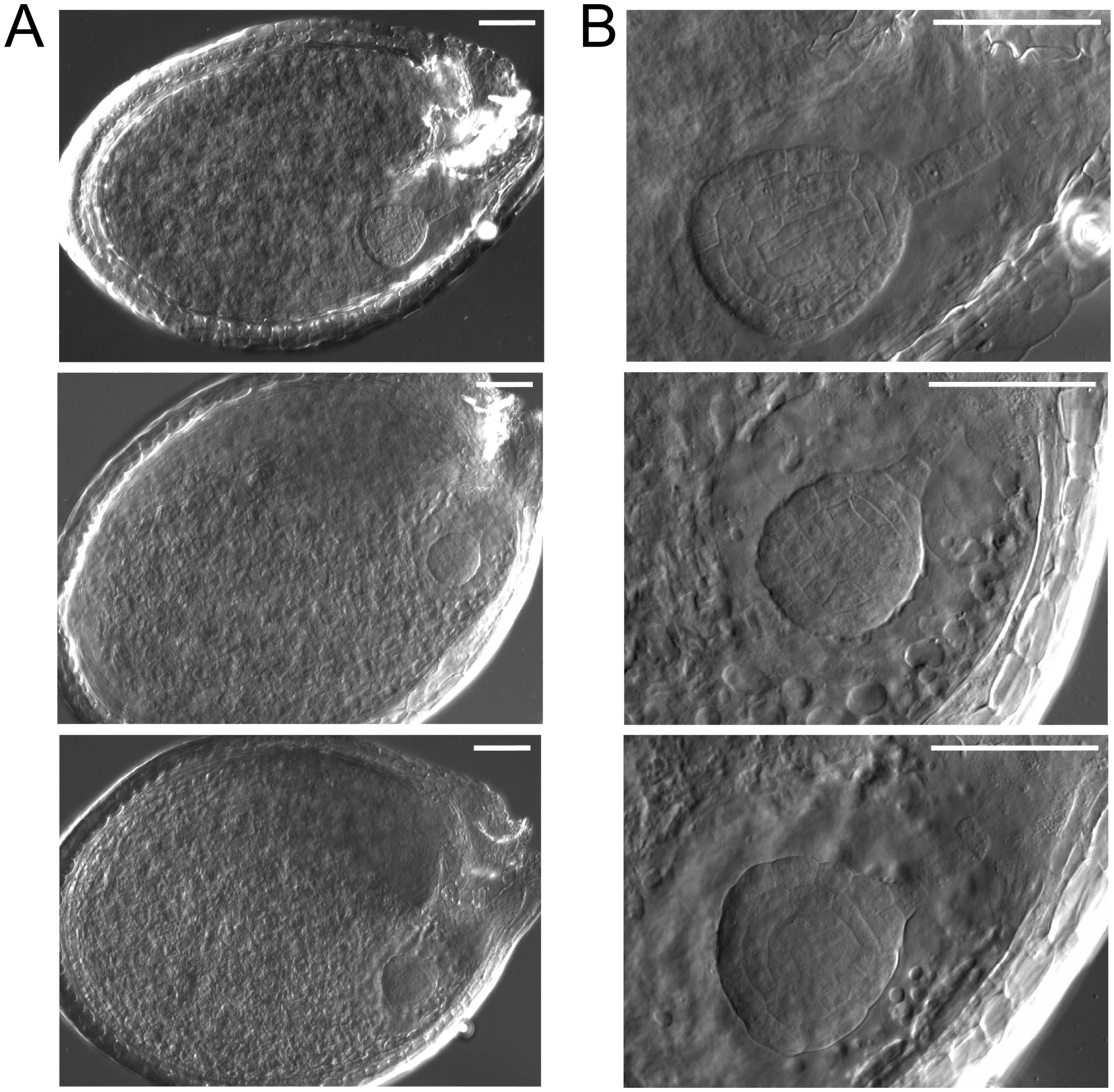

Figure S9. Embryolethality of the *citf1*-1 *fit*-2 double mutant (supports Figure 9). Three representative DIC images showing the milky-white embryos, arrested at the early heart stage of the development (**A**) and their close-ups (**B**) from the silique of the *CITF1*$^{+/-}$ *fit*-2$^{-/-}$ mutant. Scale bars = 50 µm.

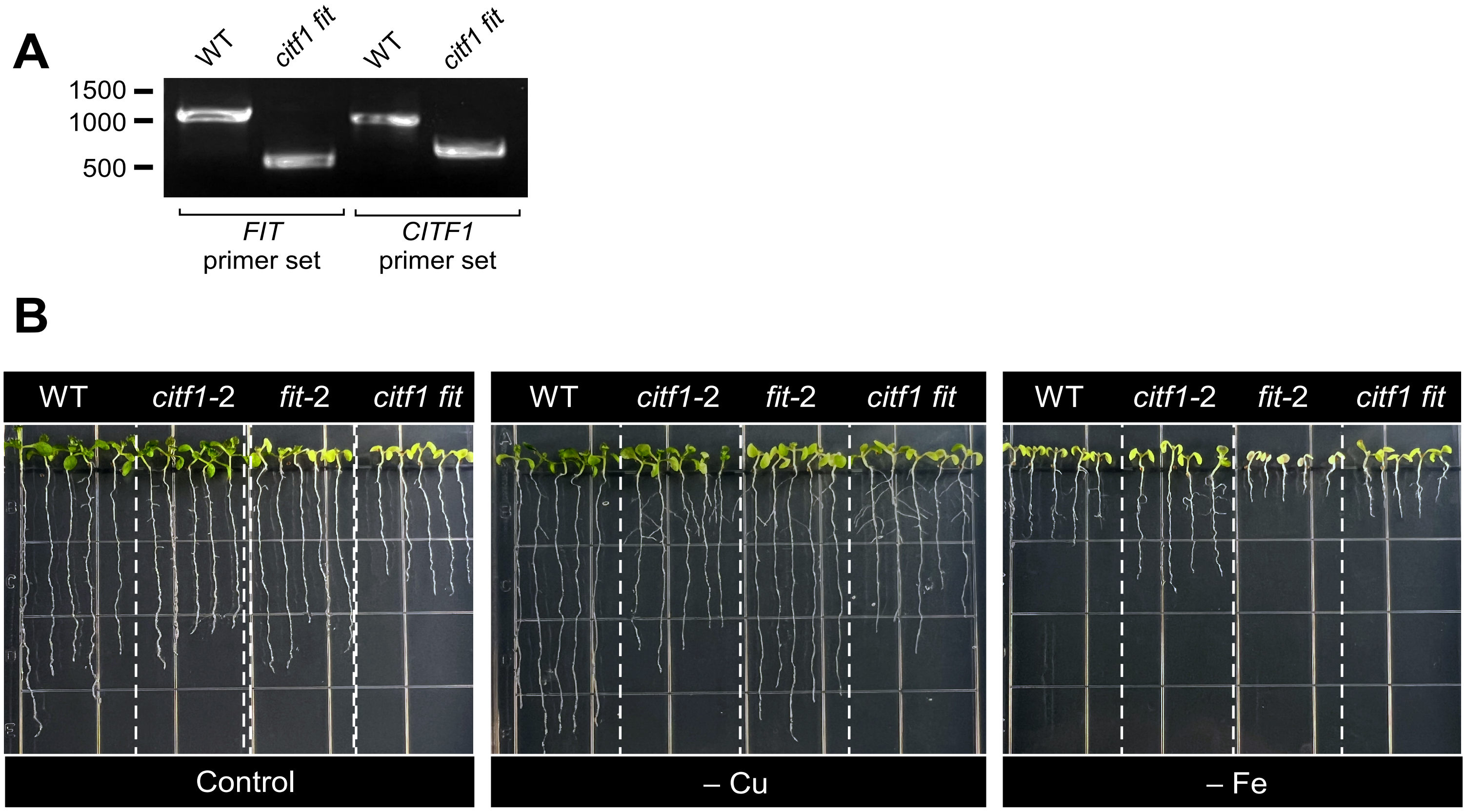

Figure S10. The *citf1-2 fit-2* double mutant shows reduced growth under control conditions and distinct sensitivity to copper and iron deficiency compared to each of the single mutants and the wild-type (supports Figure 10). (A) PCR-based genotyping to identify a homozygous *citf1*-2 *fit* double mutant. For each locus, two primer sets were used: a *CITF1* or *FIT*-specific primer pair amplifies the WT allele for both genes with the expected size of ~1,000 bp. A T-DNA border primer, in combination with a *CITF1* or *FIT*-specific primer, amplifies the expected ~ 500 bp band for each of the mutant loci, consistent with the homozygous *citf1*-2 *fit* double mutant. (**B**) Representative images of 10-day-old seedlings grown vertically on 1/2 MS agar plates under **control** conditions containing 50 μM $FeSO_4$-EDTA and 5 μM $CuSO_4$ or under Cu deficiency (–Cu), induced by replacing Cu with 100 μM BCS, or under Fe deficiency (–Fe), achieved by omitting Fe.

Supplemental Table 1. The list of CITF1-interacting proteins identified by yeast-two hybrid library screening.

*All positive clones were first selected on QDO plates followed by placing single colonies on DDO/X/A twice. The clones that showed bright blue colors when growing on DDO/X/A plates for three days were defined as strong interaction, and the clones that showed light to no blue colors or slow growth after three days of incubation were defined as weak interactions.

Accession No	Gene name	Number of positive clones	Interaction type*
AT2G28160	FIT (bHLH29)	14	Strong
AT4G19140	Unknown protein, exopolysaccharide production negative regulator	7	Weak
AT2G22230	Thioesterase superfamily protein	5	Weak
AT4G23660	PPT1, Encodes para-hydroxy benzoate polyprenyl diphosphate transferase	3	Weak
AT3G09580	FAD/NAD(P)-binding oxidoreductase family protein	2	Weak
AT5G57150	bHLH35	1	Strong
AT1G80650	RTL1, RNAse THREE-like protein 1	1	Strong
AT4G29060	EMB2726, elongation factor Ts family protein	1	Strong
AT5G26700	RmlC-like cupins superfamily protein	1	Weak
AT3G38370	AT14A-LIKE1, unknown function	1	Weak
